# Pavlovian Conditioning of Larval *Drosophila*: An Illustrated, Multilingual, Hands-On Manual for Odor-Taste Associative Learning in Maggots

**DOI:** 10.3389/fnbeh.2017.00045

**Published:** 2017-04-19

**Authors:** Birgit Michels, Timo Saumweber, Roland Biernacki, Jeanette Thum, Rupert D. V. Glasgow, Michael Schleyer, Yi-chun Chen, Claire Eschbach, Reinhard F. Stocker, Naoko Toshima, Teiichi Tanimura, Matthieu Louis, Gonzalo Arias-Gil, Manuela Marescotti, Fabio Benfenati, Bertram Gerber

**Affiliations:** ^1^Department of Genetics, Leibniz Institute for NeurobiologyMagdeburg, Germany; ^2^Department Neurobiology and Genetics, Julius Maximilians UniversityWürzburg, Germany; ^3^HHMI Janelia Research CampusAshburn, VA, USA; ^4^Department of Biology, Université de FribourgFribourg, Switzerland; ^5^Department of Biology, Kyushu UniversityFukuoka, Japan; ^6^Department of Molecular, Cellular, and Developmental Biology, University of California, Santa BarbaraSanta Barbara, CA, USA; ^7^Department Systems Physiology, Leibniz Institute for Neurobiology MagdeburgMagdeburg, Germany; ^8^Brainwave Discovery Ltd.Edinburgh, UK; ^9^Italian Institute of Technology, Center for Synaptic Neuroscience and TechnologyGenova, Italy; ^10^Institute of Biology, Otto von Guericke UniversityMagdeburg, Germany; ^11^Center for Behavioral Brain SciencesMagdeburg, Germany

**Keywords:** olfaction, taste, cognition, memory, reinforcement, association

## Abstract

Larval *Drosophila* offer a study case for behavioral neurogenetics that is simple enough to be experimentally tractable, yet complex enough to be worth the effort. We provide a detailed, hands-on manual for Pavlovian odor-reward learning in these animals. Given the versatility of *Drosophila* for genetic analyses, combined with the evolutionarily shared genetic heritage with humans, the paradigm has utility not only in behavioral neurogenetics and experimental psychology, but for translational biomedicine as well. Together with the upcoming total synaptic connectome of the *Drosophila* nervous system and the possibilities of single-cell-specific transgene expression, it offers enticing opportunities for research. Indeed, the paradigm has already been adopted by a number of labs and is robust enough to be used for teaching in classroom settings. This has given rise to a demand for a detailed, hands-on manual directed at newcomers and/or at laboratory novices, and this is what we here provide.

The paradigm and the present manual have a unique set of features:

The paradigm is cheap, easy, and robust;

The manual is detailed enough for newcomers or laboratory novices;

It briefly covers the essential scientific context;

It includes sheets for scoring, data analysis, and display;

It is multilingual: in addition to an English version we provide German, French, Japanese, Spanish and Italian language versions as well.

The present manual can thus foster science education at an earlier age and enable research by a broader community than has been the case to date.

Predictive, associative learning enables animals to decipher many aspects of the causal structure of the world and to behave accordingly (Dickinson, 2001). It is therefore a ubiquitous faculty across the animal kingdom. Indeed, following the pioneering work of Ebbinghaus, Pavlov, and Thorndike, research has uncovered remarkable conservation in the mechanisms of learning and memory (Kandel et al., 2014). Because of the feasibility of genetic screens combined with robust behavioral protocols, *Drosophila* has been one of the workhorses for these endeavors (Benzer, 1967; Dudai et al., 1976; Heisenberg et al., 1985; Tully and Quinn, 1985; reviews include Heisenberg, 2003; Gerber et al., 2014; Guven-Ozkan and Davis, 2014; Harris and Littleton, 2015; Owald and Waddell, 2015; Gerber and Aso, in press). The field received a further boost when versatile methods for transgene expression were introduced (Rubin and Spradling, 1982; O'Kane and Gehring, 1987; Brand and Perrimon, 1993), opening up the possibility for experimental manipulation with cellular specificity at the single-neuron level (Pfeiffer et al., 2010; Jenett et al., 2012; Aso et al., 2014a,b). These and related techniques (reviews include Venken et al., 2011; Sivanantharajah and Zhang, 2015) now make it relatively straightforward to express any transgene, in any cell or group of cells, at any time. Thus, *Drosophila* has become a model system for understanding learning and memory not “only” at the molecular level, but also for understanding the function of molecules within behaviorally meaningful circuitry—as envisioned by Hotta and Benzer (1970).

With a slight delay (befitting their shuffling gait, as we hesitate to add), *Drosophila* larvae entered the stage as the subjects of behavioral neurogenetics (e.g., Aceves-Piña and Quinn, 1979; Rodrigues, 1980), receiving renewed attention since the mid-1990s (Stocker, 1994; Cobb, 1999; Sokolowski, 2001; Gerber and Stocker, 2007; Gomez-Marin and Louis, 2012; Keene and Sprecher, 2012; Diegelmann et al., 2013). Larvae possess 10 times fewer neurons than adult flies, and in many cases appear to lack cellular redundancy altogether. Even so, they feature fundamental adult-like circuit motifs (e.g., in the olfactory pathways: Vosshall and Stocker, 2007; Stocker, 2008) and exhibit fundamental faculties of behavior, including learning and memory (see below). Last but not least, a synapse-by-synapse connectome of the larval nervous system seems within reach, and driver strains for transgenic manipulation can now be established to cover the neurons of the larva, one at a time (Li et al., 2014; Ohyama et al., 2015; Berck et al., 2016; Fushiki et al., 2016; Jovanic et al., 2016; Schlegel et al., 2016; Schneider-Mizell et al., 2016; Zwart et al., 2016). Taken together, the possibilities for research into the behavioral neurogenetics of larval *Drosophila* appear enticing, given the combination of analytical power, ease, elegance, and completeness.

The current contribution deals with Pavlovian odor-reward learning in larval *Drosophila* (Scherer et al., 2003; Neuser et al., 2005; Figure 1). In brief, the larvae are free to move about an agarose-filled Petri dish; the agarose substrate can either be supplemented with sugar reward, or can be used as plain substrate, not containing reward. An odor A (gray cloud in Figure 1) is presented together with a reward-supplemented substrate (+; indicated by green color in Figure 1). Then the larvae are transferred to a second Petri dish, this time with the plain substrate, and exposed to a different odor B (white cloud in Figure 1). After repeating this A+/B procedure two more times, the animals are transferred to a test Petri dish and are offered a choice between the two odors. A second set of larvae is trained reciprocally (A/B+) and is likewise tested for its preference between the two odors. If the larvae systematically prefer the previously rewarded odor relative to the previously non-rewarded odor, the conclusion is that an odor-sugar associative memory has been formed. In other words, the odor-reward association established in training guides the larvae's search for reward during the test (Gerber and Hendel, 2006; Schleyer et al., 2011, 2015a,b).

**Figure 1:**
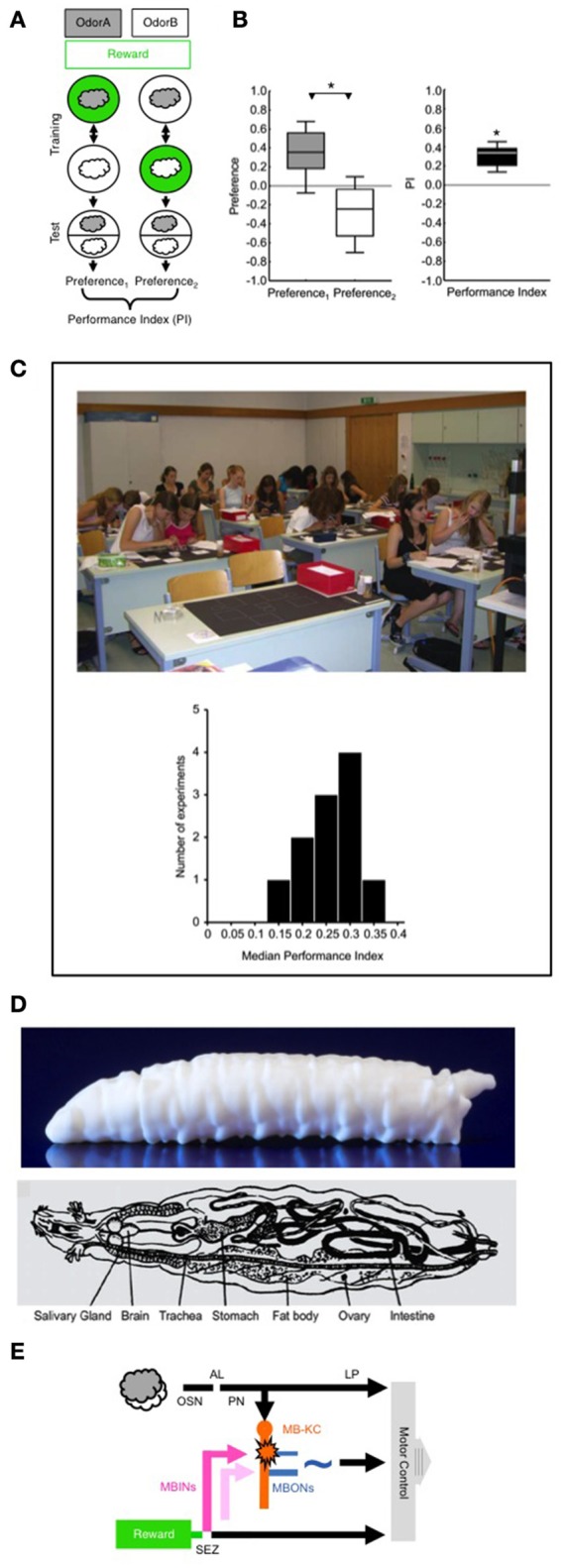
**(A)** Principle of the behavioral paradigm. In a Petri-dish assay, different groups of larvae receive odor A (gray cloud) paired with a sugar reward (green circle), alternated with presentations of another odor B (white cloud) without a reward (A+/B training); a second group of larvae is trained reciprocally (A/B+). Then, for both groups the preference of the animals between odors A and B is measured as the number of animals located on the A-side minus the number of animals located on the B-side, divided by the total number of animals (including the ones located on the middle stripe). The Performance Index is calculated as the difference in preference between the A+/B versus A/B+ trained groups of larvae (divided by 2 to yield scores between –1 and 1). The Performance Index thus represents associative memory, averaging-out effects of innate odor preference, odor exposure, reward exposure, or handling. Note that the sequence of trials is alternated across repetitions of the experiment (i.e., B/A+ and B+/A). Various sugars, aspartic acid, or low-concentration salt can alternatively be used as a taste reward; as taste punishment, quinine, or high-concentration salt can be used. **(B)** Example data from a non-academic setting. For the Preference scores (left) and the associative Performance Indices (right) the box plots show the median as the middle line, the 25/75% quantiles as box boundaries, and the 10/90% quantiles as whiskers. For the Preference scores ^*^refers to *P* < 0.05 in a Mann-Whitney *U*-test (*N* = 16, 16); for the associative Performance Indices based on these Preference scores ^*^refers to *P* < 0.05 in a one-sample sign-test. If the odor pairs, or the concentrations of the odors in a pair, are chosen such that one of them is more strongly attractive than the other, the Preference scores of both reciprocally trained groups will be shifted along the *y*-axis, i.e., will be “asymmetrically” different from zero. This does not affect the interpretation of the Performance Index as reflecting associative memory, however, because the Performance Index is based on the *difference* in Preference scores between the reciprocally trained groups (for more detail see Supplemental Material 1). **(C)** A class of 8th grade high school students performing odor-reward learning in larval *Drosophila* in a 1-day course at the Gymnasium Stettensches Institut, Augsburg, Germany. The histogram at the bottom shows the median Performance Indices from 11 such experiments in various non-academic settings, with sample sizes in the range of *N* = 12–20 each. **(D)** Side-view of a 3D print of the larval body (top; image courtesy of R. Blumenstein, LIN) and schematic overview of the internal organs of a larva (bottom; modified from Demerec and Kaufmann, 1972). **(E)** Simplified circuit diagram showing the processing of odor and taste reward. AL, antennal lobe; MBINs, mushroom body input neurons; LP, lateral protocerebrum; MB-KC, mushroom body Kenyon cells; MBONs, mushroom body output neurons; OSN, olfactory sensory neurons; PN, projection neurons. SEZ, subesophageal zone. The pink color indicates an MBIN activated by reward; the light pink color indicates an MBIN activated by punishment. The star indicates presynaptic plasticity in the MB-KC to MBON connection; the ~symbol indicates that the pathway from the MBONs toward motor control is susceptible to modulation, including modulation by the testing situation. For more details, see text. Images taken from Gerber et al. (2010) **(C)** and Demerec and Kaufmann (1972) **(D)**. The following copyright holders kindly granted permission to use these figures: Cold Spring Harbor Laboratory Press **(C)** and The Carnegie Institution **(D)**.

The working hypothesis as to how this type of learning comes about has recently been reviewed (Diegelmann et al., 2013) and is largely concordant with what has been suggested for adult flies (Heisenberg, 2003; Gerber et al., 2014; Guven-Ozkan and Davis, 2014; Harris and Littleton, 2015; Owald and Waddell, 2015; Gerber and Aso, in press) and other insects such as the honey bee (Tedjakumala and Giurfa, 2013; Menzel, 2014). In brief, larval olfactory sensory neurons are located in the dorsal organ and project to the antennal lobe. Downstream of the antennal lobe, the olfactory processing stream splits: one collateral of the projection neurons targets the lateral protocerebrum, which features premotor centers for innate olfactory behavior. The other collateral takes a “detour” to the mushroom bodies. According to the ligand profiles of the olfactory sensory neurons, the cellular properties and the connectivity within this system, including local circuitry within the antennal lobe, odors can thus be coded across these ascending olfactory pathways.

Gustatory pathways originate from multiple larval cephalic sense organs, bypass the brain, and target the subesophageal zone and premotor centers (Apostolopoulou et al., 2015). Taste pathways are thus linked relatively closely to the motor system. Notably, a “detour” branch also splits off from the gustatory pathway. From the subesophageal zone this sends information about the reinforcing value of the food toward the brain. Through an as yet unknown number of synaptic steps, this activates octopaminergic as well as dopaminergic input neurons signaling toward the Kenyon cells of the mushroom body (Schroll et al., 2006; Rohwedder et al., 2016; regarding adult *Drosophila*, reviews include Heisenberg, 2003; Gerber et al., 2014; Guven-Ozkan and Davis, 2014; Owald and Waddell, 2015; Gerber and Aso, in press; see also Hammer, 1993; Kreissl et al., 1994 on the bee).

Within the mushroom body Kenyon cells, a coincidence can thus be detected between olfactory input in terms of an odor-specific subset of activated Kenyon cells, and an internal aminergic reinforcement signal. This coincidence modulates the synapse between the odor-activated set of mushroom body Kenyon cells and their output neurons, by processes taking place presynaptically within the respective Kenyon cells. If a trained odor is subsequently encountered, it is via this odor-specific set of modulated synapses that the balance is shifted between mushroom body output neurons favoring approach and mushroom body output neurons mediating avoidance. By analogy with what has been observed in adult *Drosophila* (for reviews see Owald and Waddell, 2015; Gerber and Aso, in press), learned approach may come about by a weakening of synapses from Kenyon cells to those output neurons that are sufficient for avoidance, resulting in net relative attraction. Note that the pathway from the mushroom body output neurons carrying learned valence signals toward motor control comprises an as yet unknown number of synaptic steps and is susceptible to modulation, including modulation by the testing situation (Gerber and Hendel, 2006; Schleyer et al., 2011, 2015a,b).

Since its introduction this paradigm has made significant advances possible, including the first application of Channelrhodopsin-2 in a brain (Schroll et al., 2006), and the discovery of memories specific to the kind of reward (fructose vs. amino acid) and the kind of punishment (quinine versus high-concentration salt; Schleyer et al., 2015a). It has been adopted by a number of labs, including new groups entering the field of learning and memory. Indeed, the paradigm is robust enough to be routinely used for undergraduate teaching and in classroom settings. This has given rise to a demand for a detailed, hands-on manual directed at newcomers in the field of behavioral science and/or at laboratory novices, and this is what we here provide (Supplemental Materials 1–16). The paradigm and the presented manual have a unique set of features:

- The paradigm is cheap and easy to carry out, and can be performed in classroom settings under “degraded” experimental conditions;
- The manual is richly illustrated and detailed enough to allow newcomers or laboratory novices, even at high school level, to perform the experiment;
- It features brief “introduction” and “outlook” sections covering the scientific context and guidelines for the display and the analysis of the data;
- It includes data sheets for scoring, and customized excel sheets for data analysis and display;
- Possibly most importantly for use in schools, we provide not only an English version (Supplemental Materials 1–3), but German (Supplemental Materials 4–6), French (Supplemental Materials 7–9), Japanese (Supplemental Material 10) Spanish (Supplemental Materials 11–13), and Italian (Supplemental Materials 14–16) language versions as well.

The current contribution can thus foster science education at an earlier age and enable research by a broader community than has been the case to date (Gerber et al., 2010, 2013; Apostolopoulou et al., 2013). The paradigm allows experimental access to a fascinating aspect of nervous system function: the adaptive balance between robustness and flexibility of behavior. Given the versatility of *Drosophila* for genetic analyses, combined with their evolutionarily shared genetic heritage with humans, the paradigm has utility not only in behavioral science, genetics, neurobiology, and experimental psychology, but for translational biomedicine as well.

## Ethics statement

Procedures comply with applicable law for experimentation with invertebrates of the State of Sachsen-Anhalt and the Federal Republic of Germany.

## Author contributions

BG: Authored manuscript, co-authored Supplement 1–6. BM, TS, RB, JT, RG, MS, YC: Authored Supplement 1–6, co-authored manuscript. CE, RS, ML: Authored Supplement 7–9, co-authored manuscript. NT, TT: Authored Supplement 10. GA, RG: Authored Supplement 11–13. MM, FB: Authored Supplements 14–16.

### Conflict of interest statement

The authors declare that the research was conducted in the absence of any commercial or financial relationships that could be construed as a potential conflict of interest.

## Supplementary material

Supplemental Materials 1–3 **A manual for odor-reward learning in larval *Drosophila* (Supplemental Material 1), example of a table for data analysis (Supplemental Material 2), and an empty table for entering and analyzing one's own data (Supplemental Material 3), in the English language**. Versions of this manual in the German, French, Japanese, Spanish, and Italian languages can be found in Supplemental Materials 4–6, 7–9, 10, 11–13, 14–16, respectively.

Supplemental Materials 4–6 **A manual for odor-reward learning in larval *Drosophila* (Supplemental Material 4), example of a table for data analysis (Supplemental Material 5), and an empty table for entering and analyzing one's own data (Supplemental Material 6), in the German language**. Versions of this manual in the English, French, Japanese, Spanish, and Italian languages can be found in Supplemental Materials 1–3, 7–9, 10, 11–13, 14–16, respectively.

Supplemental Materials 7–9 **A manual for odor-reward learning in larval *Drosophila* (Supplemental Material 7), example of a table for data analysis (Supplemental Material 8), and an empty table for entering and analyzing one's own data (Supplemental Material 9), in the French language**. Versions of this manual in the English, German, Japanese, Spanish, and Italian languages can be found in Supplemental Materials 1–3, 4–6, 10, 11–13, 14–16, respectively.

Supplemental Material 10 **A manual for odor-reward learning in larval *Drosophila* in the Japanese language**. Versions of this manual in the English, German, French, Spanish, and Italian languages can be found in Supplemental Materials 1–3, 4–6, 7–9, 11–13, 14–16, respectively. For a table for data analysis and an empty table for entering and analyzing one's own data, please use the document in the English language (Supplemental Materials 2, 3, respectively).

Supplemental Materials 11–13 **A manual for odor-reward learning in larval *Drosophila* (Supplemental Material 11), example of a table for data analysis (Supplemental Material 12), and an empty table for entering and analyzing one's own data (Supplemental Material 13), in the Spanish language**. Versions of this manual in the English, German, French, Japanese, and Italian languages can be found in Supplemental Materials 1–3, 4–6, 7–9, 10, 14–16, respectively.

Supplemental Materials 14–16 **A manual for odor-reward learning in larval *Drosophila* (Supplemental Material 14), example of a table for data analysis (Supplemental Material 15), and an empty table for entering and analyzing one's own data (Supplemental Material 16), in the Italian language**. Versions of this manual in the English, German, French, Japanese, and Spanish languages can be found in Supplemental Materials 1–3, 4–6, 7–9, 10, and 11–13, respectively.

## References

[B1] Aceves-Piña E. O. Quinn W. G. (1979). Learning in normal and mutant Drosophila larvae. Science 206, 93–96. 10.1126/science.206.4414.93 17812455

[B2] Apostolopoulou A. A. Rist A. Thum A. S. (2015). Taste processing in Drosophila larvae. Front. Integr. Neurosci. 9:50. 10.3389/fnint.2015.00050 26528147 PMC4602287

[B3] Apostolopoulou A. A. Widmann A. Rohwedder A. Pfitzenmaier J. E. Thum A. S. (2013). Appetitive associative olfactory learning in Drosophila larvae. J. Vis. Exp. 72:4334 10.3791/4334 PMC3601210 23438816

[B4] Aso Y. Hattori D. Yu Y. Johnston R. M. Iyer N. A. Ngo T. T.-B.. (2014a). The neuronal architecture of the mushroom body provides a logic for associative learning. eLife 3:e04577. 10.7554/eLife.04577 25535793 PMC4273437

[B5] Aso Y. Sitaraman D. Ichinose T. Kaun K. R. Vogt K. Belliart-Guérin G.. (2014b). Mushroom body output neurons encode valence and guide memory-based action selection in *Drosophila*. eLife 3:e04580. 10.7554/eLife.04580 25535794 PMC4273436

[B6] Benzer S. (1967). Behavioral mutants of Drosophila isolated by countercurrent distribution. Proc. Natl. Acad. Sci. U.S.A. 58, 1112–1119. 10.1073/pnas.58.3.1112 16578662 PMC335755

[B7] Berck M. E. Khandelwal A. Claus L. Hernandez-Nunez L. Si G. Tabone C. J.. (2016). The wiring diagram of a glomerular olfactory system. eLife 5:e14859. 10.7554/eLife.14859 27177418 PMC4930330

[B8] Brand A. H. Perrimon N. (1993). Targeted gene expression as a means of altering cell fates and generating dominant phenotypes. Development 118, 401–415. 8223268 10.1242/dev.118.2.401

[B9] Cobb M. (1999). What and how do maggots smell? Biol. Rev. 74, 425–459.

[B10] Demerec M. Kaufmann B. P. (1972). Drosophila Guide: Introduction to the Genetics and Cytology of Drosophila Melanogaster. Washington, DC: Carnegie Institution of Washington.

[B11] Dickinson A. (2001). The 28th Bartlett Memorial Lecture. Causal learning: an associative analysis. Q. J. Exp. Psychol. B 54, 3–25. 10.1080/02724990042000010 11216300

[B12] Diegelmann S. Klagges B. Michels B. Schleyer M. Gerber B. (2013). Maggot learning and Synapsin function. J. Exp. Biol. 216, 939–951. 10.1242/jeb.076208 23447663

[B13] Dudai Y. Jan Y. N. Byers D. Quinn W. G. Benzer S. (1976). dunce, a mutant of Drosophila deficient in learning. Proc. Natl. Acad. Sci. U.S.A. 73, 1684–1688. 10.1073/pnas.73.5.1684 818641 PMC430364

[B14] Fushiki A. Zwart M. F. Kohsaka H. Fetter R. D. Cardona A. Nose A. (2016). A circuit mechanism for the propagation of waves of muscle contraction in *Drosophila*. eLife 5:e13253. 10.7554/eLife.13253 26880545 PMC4829418

[B15] Gerber B. Aso Y. (in press). Localization, diversity behavioral expression of associative engrams in Drosophila, in Learning Theory Behavior, ed Menzel R. (Oxford: Elsevier).

[B16] Gerber B. Hendel T. (2006). Outcome expectations drive learned behaviour in larval Drosophila. Proc. Biol. Sci. 273, 2965–2968. 10.1098/rspb.2006.3673 17015355 PMC1639518

[B17] Gerber B. Stocker R. F. (2007). The Drosophila larva as a model for studying chemosensation and chemosensory learning: a review. Chem. Senses 32, 65–89. 10.1093/chemse/bjl030 17071942

[B18] Gerber B. Biernacki R. Thum J. (2010). Odor–taste learning in larval Drosophila, in Drosophila Neurobiology: A Laboratory Manual, eds Zhang B. Freeman M. R. Waddell S. (Cold Spring Harbor, NY: Cold Spring Harbor Laboratory Press), 443–455.

[B19] Gerber B. Biernacki R. Thum J. (2013). Odor-taste learning assays in Drosophila larvae. Cold Spring Harb. Protoc. 2013:pdb.prot071639. 10.1101/pdb.prot071639 23457337

[B20] Gerber B. Yarali A. Diegelmann S. Wotjak C. T. Pauli P. Fendt M. (2014). Pain-relief learning in flies, rats, and man: basic research and applied perspectives. Learn. Mem. 21, 232–252. 10.1101/lm.032995.113 24643725 PMC3966540

[B21] Gomez-Marin A. Louis M. (2012). Active sensation during orientation behavior in the Drosophila larva: more sense than luck. Curr. Opin. Neurobiol. 22, 208–215. 10.1016/j.conb.2011.11.008 22169055

[B22] Guven-Ozkan T. Davis R. L. (2014). Functional neuroanatomy of Drosophila olfactory memory formation. Learn. Mem. 21, 519–526. 10.1101/lm.034363.114 25225297 PMC4175493

[B23] Hammer M. (1993). An identified neuron mediates the unconditioned stimulus in associative olfactory learning in honeybees. Nature 366, 59–63. 10.1038/366059a0 24308080

[B24] Harris K. P. Littleton J. T. (2015). Transmission, development, and plasticity of synapses. Genetics 201, 345–375. 10.1534/genetics.115.176529 26447126 PMC4596655

[B25] Heisenberg M. (2003). Mushroom body memoir: from maps to models. Nat. Rev. Neurosci. 4, 266–275. 10.1038/nrn1074 12671643

[B26] Heisenberg M. Borst A. Wagner S. Byers D. (1985). Drosophila mushroom body mutants are deficient in olfactory learning. J. Neurogenet. 2, 1–30. 10.3109/01677068509100140 4020527

[B27] Hotta Y. Benzer S. (1970). Genetic dissection of the Drosophila nervous system by means of mosaics. Proc. Natl. Acad. Sci. U.S.A. 67, 1156–1163. 10.1073/pnas.67.3.1156 5274445 PMC283331

[B28] Jenett A. Rubin G. M. Ngo T. T. Shepherd D. Murphy C. Dionne H.. (2012). A GAL4-driver line resource for Drosophila neurobiology. Cell Rep. 2, 991–1001. 10.1016/j.celrep.2012.09.011 23063364 PMC3515021

[B29] Jovanic T. Schneider-Mizell C. M. Shao M. Masson J. B. Denisov G. Fetter R. D.. (2016). Competitive disinhibition mediates behavioral choice and sequences in Drosophila. Cell 167, 858–870. 10.1016/j.cell.2016.09.009 27720450

[B30] Kandel E. R. Dudai Y. Mayford M. R. (2014). The molecular and systems biology of memory. Cell 157, 163–186. 10.1016/j.cell.2014.03.001 24679534

[B31] Keene A. C. Sprecher S. G. (2012). Seeing the light: photobehavior in fruit fly larvae. Trends Neurosci. 35, 104–110. 10.1016/j.tins.2011.11.003 22222349

[B32] Kreissl S. Eichmüller S. Bicker G. Rapus J. Eckert M. (1994). Octopamine-like immunoreactivity in the brain and subesophageal ganglion of the honeybee. J. Comp. Neurol. 348, 583–595. 10.1002/cne.903480408 7530730

[B33] Li H. H. Kroll J. R. Lennox S. M. Ogundeyi O. Jeter J. Depasquale G.. (2014). A GAL4 driver resource for developmental and behavioral studies on the larval CNS of Drosophila. Cell Rep. 8, 897–908. 10.1016/j.celrep.2014.06.065 25088417

[B34] Menzel R. (2014). The insect mushroom body, an experience-dependent recoding device. J. Physiol. Paris 108, 84–95. 10.1016/j.jphysparis.2014.07.004 25092259

[B35] Neuser K. Husse J. Stock P. Gerber B. (2005). Appetitive olfactory learning in Drosophila larvae: effects of repetition, reward strength, age, gender, assay type and memory span. Anim. Behav. 69, 891–898. 10.1016/j.anbehav.2004.06.013

[B36] O'Kane C. J. Gehring W. J. (1987). Detection in situ of genomic regulatory elements in Drosophila. Proc. Natl. Acad. Sci. U.S.A. 84, 9123–9127. 10.1073/pnas.84.24.9123 2827169 PMC299704

[B37] Ohyama T. Schneider-Mizell C. M. Fetter R. D. Aleman J. V. Franconville R. Rivera-Alba M.. (2015). A multilevel multimodal circuit enhances action selection in Drosophila. Nature 520, 633–639. 10.1038/nature14297 25896325

[B38] Owald D. Waddell S. (2015). Olfactory learning skews mushroom body output pathways to steer behavioral choice in Drosophila. Curr. Opin. Neurobiol. 35, 178–184. 10.1016/j.conb.2015.10.002 26496148 PMC4835525

[B39] Pfeiffer B. D. Ngo T. T. Hibbard K. L. Murphy C. Jenett A. Truman J. W.. (2010). Refinement of tools for targeted gene expression in Drosophila. Genetics 186, 735–755. 10.1534/genetics.110.119917 20697123 PMC2942869

[B40] Rodrigues V. (1980). Olfactory behavior of *Drosophila melanogaster*. Basic Life Sci. 16, 361–371. 10.1007/978-1-4684-7968-3_26 6779801

[B41] Rohwedder A. Wenz N. L. Stehle B. Huser A. Yamagata N. Zlatic M.. (2016). Four individually identified paired dopamine neurons signal reward in larval Drosophila. Curr. Biol. 26, 661–669. 10.1016/j.cub.2016.01.012 26877086

[B42] Rubin G. M. Spradling A. C. (1982). Genetic transformation of Drosophila with transposable element vectors. Science 218, 348–353. 10.1126/science.6289436 6289436

[B43] Scherer S. Stocker R. F. Gerber B. (2003). Olfactory learning in individually assayed Drosophila larvae. Learn. Mem. 10, 217–225. 10.1101/lm.57903 12773586 PMC202312

[B44] Schlegel P. Texada M. J. Miroschnikow A. Schoofs A. Hückesfeld S. Peters M.. (2016). Synaptic transmission parallels neuromodulation in a central food-intake circuit. eLife 5:e16799. 10.7554/eLife.16799 27845623 PMC5182061

[B45] Schleyer M. Miura D. Tanimura T. Gerber B. (2015a). Learning the specific quality of taste reinforcement in larval *Drosophila*. eLife 4:e04711. 10.7554/eLife.04711 25622533 PMC4302267

[B46] Schleyer M. Reid S. F. Pamir E. Saumweber T. Paisios E. Davies A.. (2015b). The impact of odor-reward memory on chemotaxis in larval Drosophila. Learn. Mem. 22, 267–277. 10.1101/lm.037978.114 25887280 PMC4408773

[B47] Schleyer M. Saumweber T. Nahrendorf W. Fischer B. von Alpen D. Pauls D.. (2011). A behavior-based circuit model of how outcome expectations organize learned behavior in larval Drosophila. Learn. Mem. 18, 639–653. 10.1101/lm.2163411 21946956

[B48] Schneider-Mizell C. M. Gerhard S. Longair M. Kazimiers T. Li F. Zwart M. F.. (2016). Quantitative neuroanatomy for connectomics in *Drosophila*. eLife 5:e12059. 10.7554/eLife.12059 26990779 PMC4811773

[B49] Schroll C. Riemensperger T. Bucher D. Ehmer J. Voller T. Erbguth K. (2006). Light-induced activation of distinct modulatory neurons triggers appetitive or aversive learning in Drosophila larvae. Curr. Biol. 16, 1741–1747. 10.1016/j.cub.2006.07.023 16950113

[B50] Sivanantharajah L. Zhang B. (2015). Current techniques for high-resolution mapping of behavioral circuits in Drosophila. J. Comp. Physiol. A Neuroethol. Sens. Neural Behav. Physiol. 201, 895–909. 10.1007/s00359-015-1010-y 25925433

[B51] Sokolowski M. B. (2001). Drosophila: genetics meets behaviour. Nat. Rev. Genet. 2, 879–890. 10.1038/35098592 11715043

[B52] Stocker R. F. (1994). The organization of the chemosensory system in *Drosophila melanogaster*: a review. Cell Tissue Res. 275, 3–26. 10.1007/BF00305372 8118845

[B53] Stocker R. F. (2008). Design of the larval chemosensory system. Adv. Exp. Med. Biol. 628, 69–81. 10.1007/978-0-387-78261-4_5 18683639

[B54] Tedjakumala S. R. Giurfa M. (2013). Rules and mechanisms of punishment learning in honey bees: the aversive conditioning of the sting extension response. J. Exp. Biol. 216, 2985–2997. 10.1242/jeb.086629 23885086

[B55] Tully T. Quinn W. G. (1985). Classical conditioning and retention in normal and mutant *Drosophila melanogaster*. J. Comp. Physiol. A 157, 263–277. 10.1007/BF01350033 3939242

[B56] Venken K. J. Simpson J. H. Bellen H. J. (2011). Genetic manipulation of genes and cells in the nervous system of the fruit fly. Neuron 72, 202–230. 10.1016/j.neuron.2011.09.021 22017985 PMC3232021

[B57] Vosshall L. B. Stocker R. F. (2007). Molecular architecture of smell and taste in Drosophila. Annu. Rev. Neurosci. 30, 505–533. 10.1146/annurev.neuro.30.051606.094306 17506643

[B58] Zwart M. F. Pulver S. R. Truman J. W. Fushiki A. Fetter R. D. Cardona A. (2016). Selective inhibition mediates the sequential recruitment of motor pools. Neuron 91, 615–628. 10.1016/j.neuron.2016.06.031 27427461 PMC4980426

