# Supplementary material for: Pavlovian Conditioning of Larval Drosophila: An Illustrated, Multilingual, Hands-On Manual for Odor-Taste Associative Learning in Maggots
Source: Front Behav Neurosci. 2017 Apr 19;11:45. doi: 10.3389/fnbeh.2017.00045 (PMC5395560; doi:10.3389/fnbeh.2017.00045)
Supplement: Supplemental Materials 1–3 — A manual for odor-reward learning in larval Drosophila (Supplemental Material 1), example of a table for data analysis (Supplemental Material 2), and an empty table for entering and analyzing one's own data (Supplemental Material 3), in the English language. Versions of this manual in the German, French, Japanese, Spanish, and Italian languages can be found in Supplemental Materials 4–6, 7–9, 10, 11–13, 14–16, respectively. [file SupplementalMaterial1.pptx]

## Slide 1
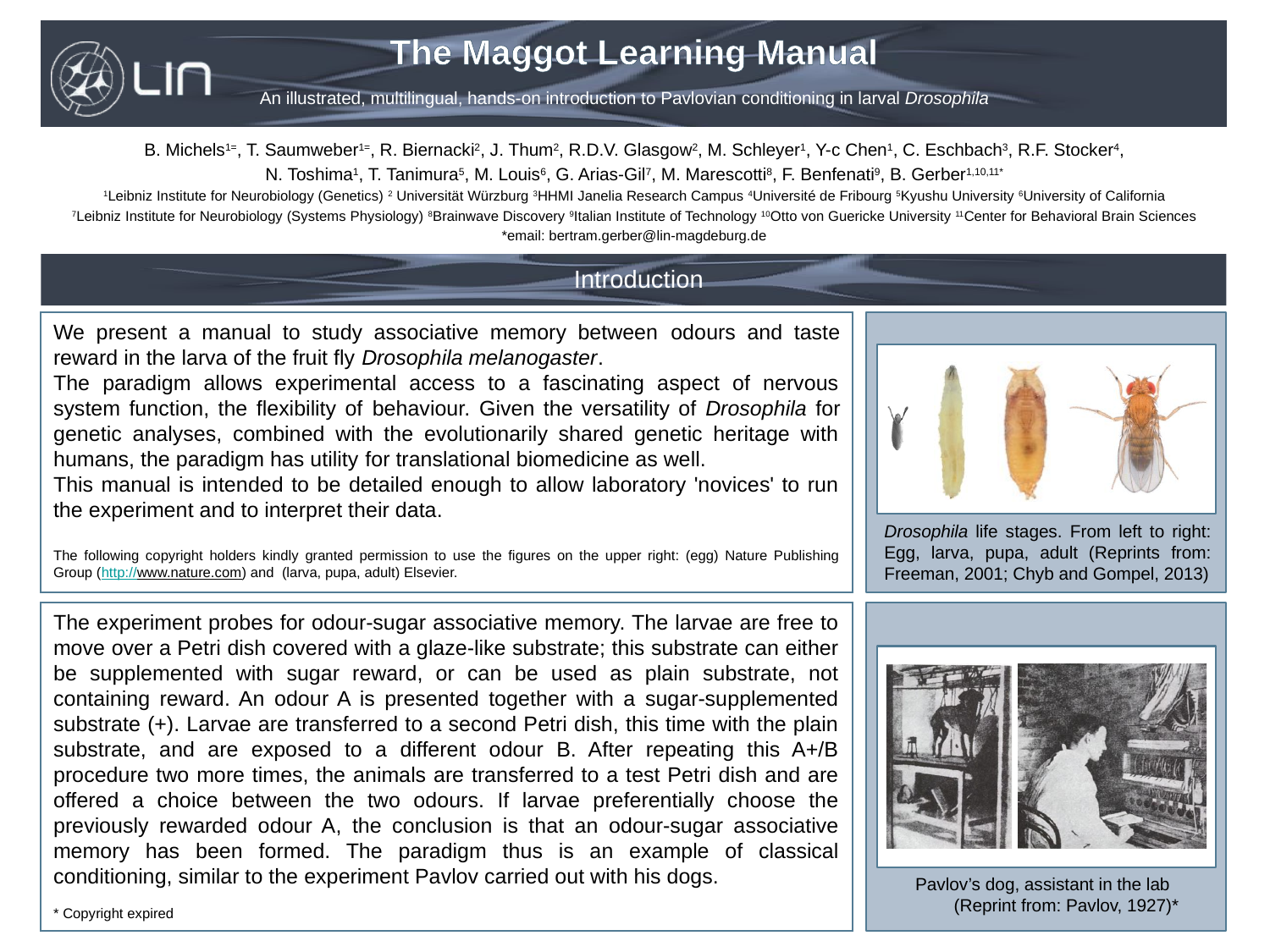

# An illustrated, multilingual, hands-on introduction to Pavlovian conditioning in larval Drosophila
The Maggot Learning Manual
B. Michels1=, T. Saumweber1=, R. Biernacki2, J. Thum2, R.D.V. Glasgow2, M. Schleyer1, Y-c Chen1, C. Eschbach3, R.F. Stocker4,
N. Toshima1, T. Tanimura5, M. Louis6, G. Arias-Gil7, M. Marescotti8, F. Benfenati9, B. Gerber1,10,11*
1Leibniz Institute for Neurobiology (Genetics) 2 Universität Würzburg 3HHMI Janelia Research Campus 4Université de Fribourg 5Kyushu University 6University of California
7Leibniz Institute for Neurobiology (Systems Physiology) 8Brainwave Discovery 9Italian Institute of Technology 10Otto von Guericke University 11Center for Behavioral Brain Sciences
*email: bertram.gerber@lin-magdeburg.de
Introduction
We present a manual to study associative memory between odours and taste reward in the larva of the fruit fly Drosophila melanogaster.
The paradigm allows experimental access to a fascinating aspect of nervous system function, the flexibility of behaviour. Given the versatility of Drosophila for genetic analyses, combined with the evolutionarily shared genetic heritage with humans, the paradigm has utility for translational biomedicine as well.
This manual is intended to be detailed enough to allow laboratory 'novices' to run the experiment and to interpret their data.
The following copyright holders kindly granted permission to use the figures on the upper right: (egg) Nature Publishing Group (http://www.nature.com) and (larva, pupa, adult) Elsevier.
Drosophila life stages. From left to right: Egg, larva, pupa, adult (Reprints from: Freeman, 2001; Chyb and Gompel, 2013)
The experiment probes for odour-sugar associative memory. The larvae are free to move over a Petri dish covered with a glaze-like substrate; this substrate can either be supplemented with sugar reward, or can be used as plain substrate, not containing reward. An odour A is presented together with a sugar-supplemented substrate (+). Larvae are transferred to a second Petri dish, this time with the plain substrate, and are exposed to a different odour B. After repeating this A+/B procedure two more times, the animals are transferred to a test Petri dish and are offered a choice between the two odours. If larvae preferentially choose the previously rewarded odour A, the conclusion is that an odour-sugar associative memory has been formed. The paradigm thus is an example of classical conditioning, similar to the experiment Pavlov carried out with his dogs.
* Copyright expired
Pavlov’s dog, assistant in the lab (Reprint from: Pavlov, 1927)*

## Slide 2
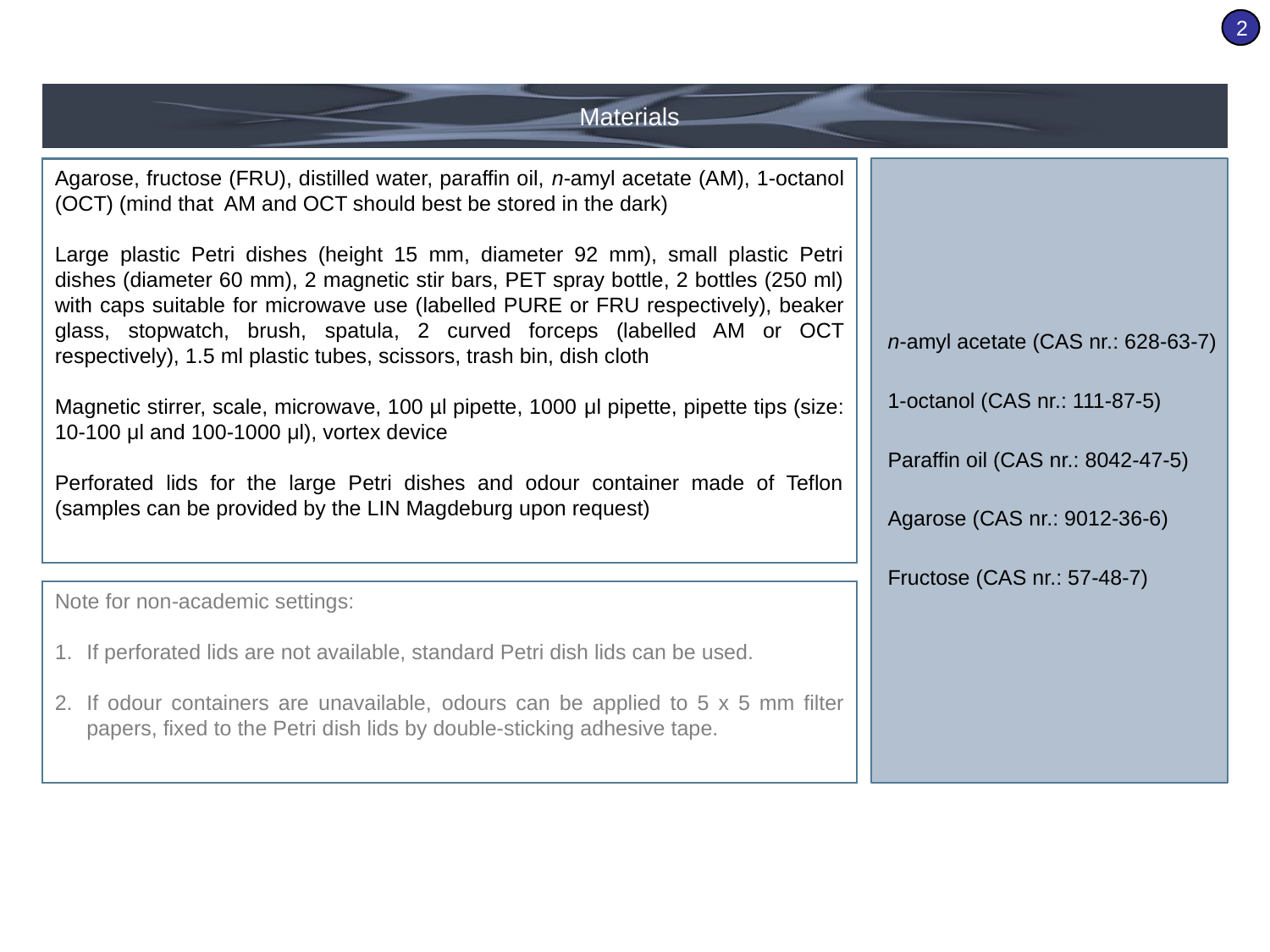

2
Materials
Agarose, fructose (FRU), distilled water, paraffin oil, n-amyl acetate (AM), 1-octanol (OCT) (mind that AM and OCT should best be stored in the dark)
Large plastic Petri dishes (height 15 mm, diameter 92 mm), small plastic Petri dishes (diameter 60 mm), 2 magnetic stir bars, PET spray bottle, 2 bottles (250 ml) with caps suitable for microwave use (labelled PURE or FRU respectively), beaker glass, stopwatch, brush, spatula, 2 curved forceps (labelled AM or OCT respectively), 1.5 ml plastic tubes, scissors, trash bin, dish cloth
Magnetic stirrer, scale, microwave, 100 µl pipette, 1000 μl pipette, pipette tips (size: 10-100 μl and 100-1000 μl), vortex device
Perforated lids for the large Petri dishes and odour container made of Teflon (samples can be provided by the LIN Magdeburg upon request)
n-amyl acetate (CAS nr.: 628-63-7)
1-octanol (CAS nr.: 111-87-5)
Paraffin oil (CAS nr.: 8042-47-5)
Agarose (CAS nr.: 9012-36-6)
Fructose (CAS nr.: 57-48-7)
Note for non-academic settings:
If perforated lids are not available, standard Petri dish lids can be used.
If odour containers are unavailable, odours can be applied to 5 x 5 mm filter papers, fixed to the Petri dish lids by double-sticking adhesive tape.

## Slide 3
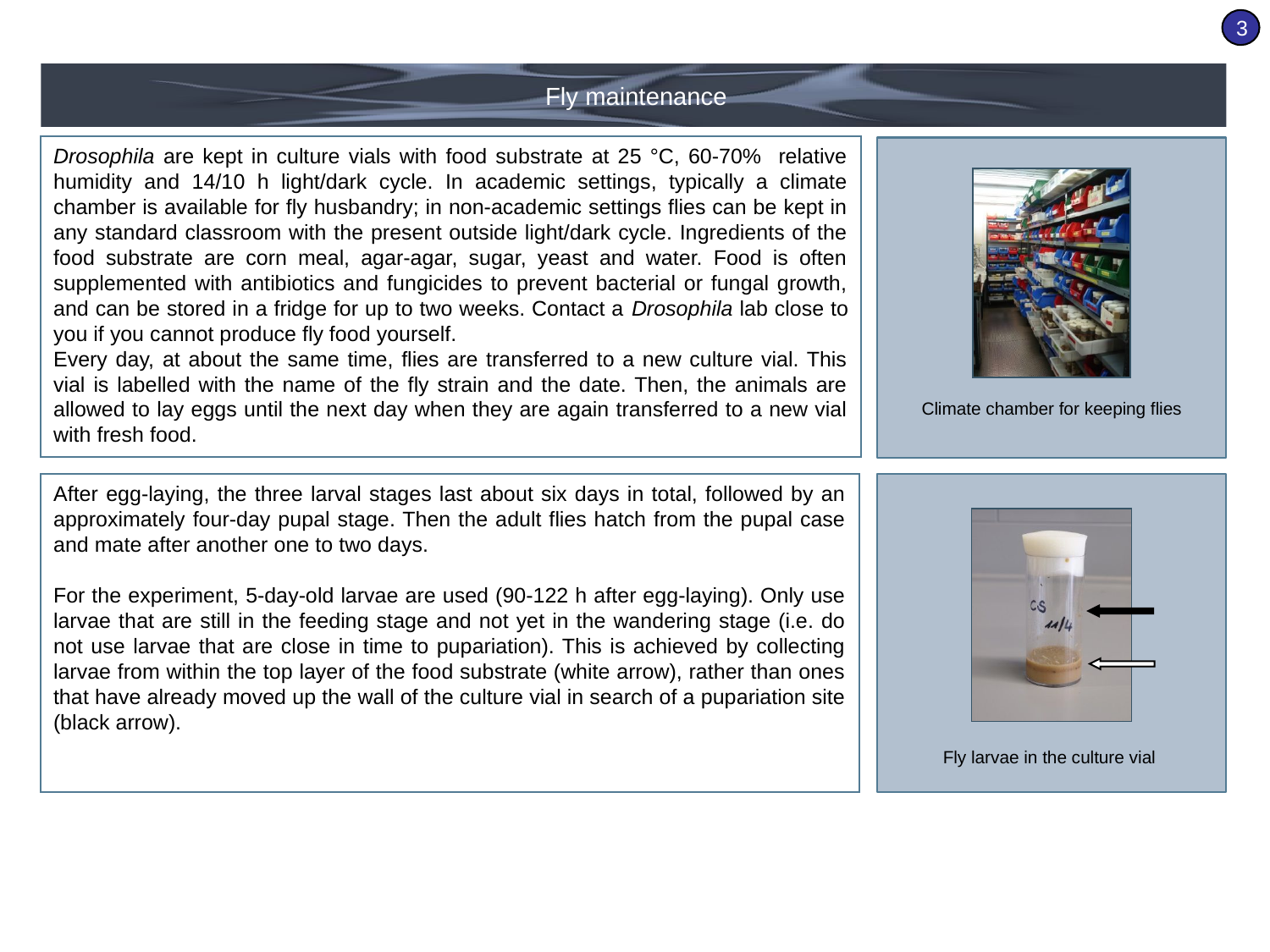

3
# Fly maintenance
Drosophila are kept in culture vials with food substrate at 25 °C, 60-70% relative humidity and 14/10 h light/dark cycle. In academic settings, typically a climate chamber is available for fly husbandry; in non-academic settings flies can be kept in any standard classroom with the present outside light/dark cycle. Ingredients of the food substrate are corn meal, agar-agar, sugar, yeast and water. Food is often supplemented with antibiotics and fungicides to prevent bacterial or fungal growth, and can be stored in a fridge for up to two weeks. Contact a Drosophila lab close to you if you cannot produce fly food yourself.
Every day, at about the same time, flies are transferred to a new culture vial. This vial is labelled with the name of the fly strain and the date. Then, the animals are allowed to lay eggs until the next day when they are again transferred to a new vial with fresh food.
Climate chamber for keeping flies
After egg-laying, the three larval stages last about six days in total, followed by an approximately four-day pupal stage. Then the adult flies hatch from the pupal case and mate after another one to two days.
For the experiment, 5-day-old larvae are used (90-122 h after egg-laying). Only use larvae that are still in the feeding stage and not yet in the wandering stage (i.e. do not use larvae that are close in time to pupariation). This is achieved by collecting larvae from within the top layer of the food substrate (white arrow), rather than ones that have already moved up the wall of the culture vial in search of a pupariation site (black arrow).
Fly larvae in the culture vial

## Slide 4
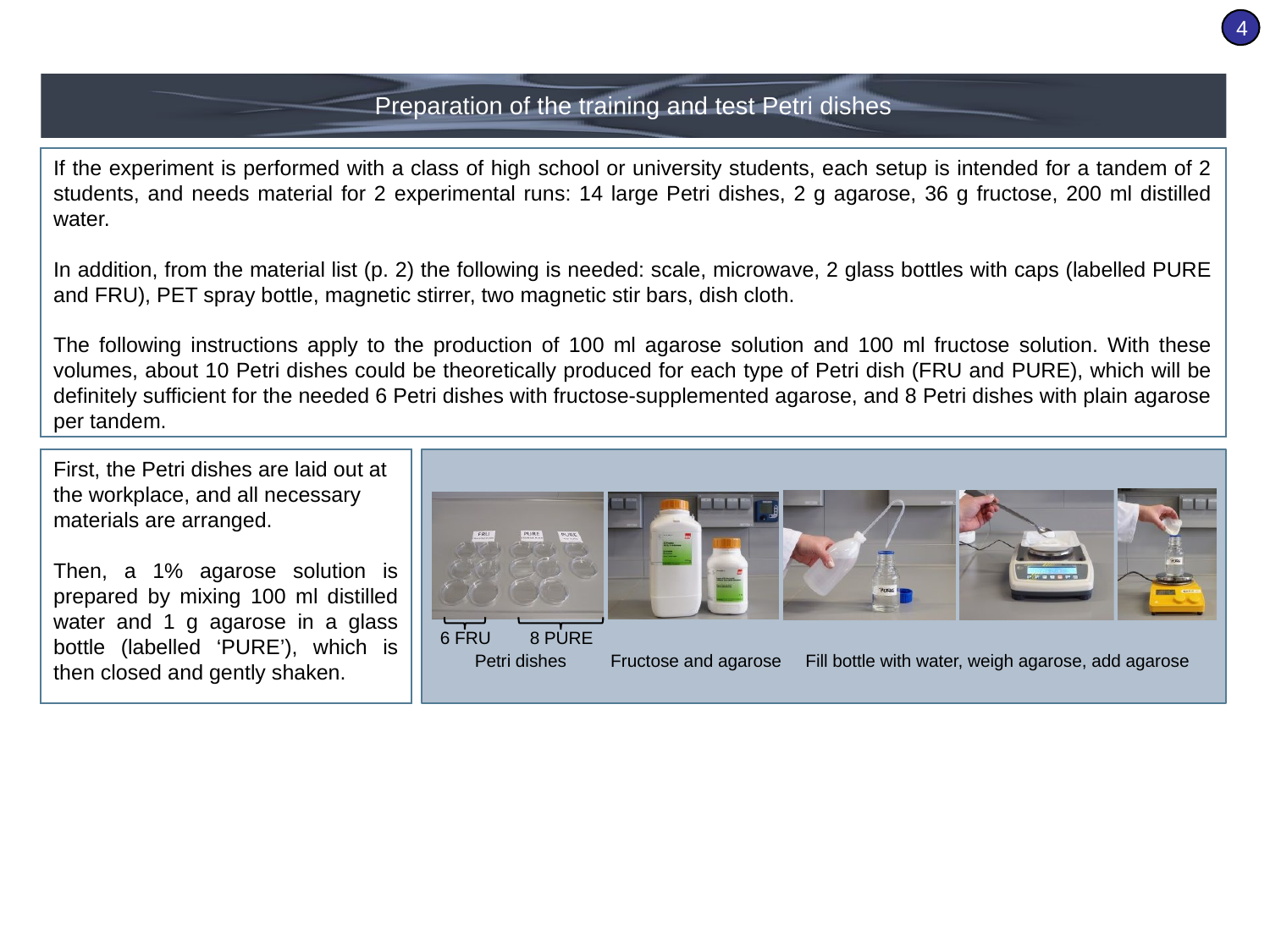

4
# Preparation of the training and test Petri dishes
If the experiment is performed with a class of high school or university students, each setup is intended for a tandem of 2 students, and needs material for 2 experimental runs: 14 large Petri dishes, 2 g agarose, 36 g fructose, 200 ml distilled water.
In addition, from the material list (p. 2) the following is needed: scale, microwave, 2 glass bottles with caps (labelled PURE and FRU), PET spray bottle, magnetic stirrer, two magnetic stir bars, dish cloth.
The following instructions apply to the production of 100 ml agarose solution and 100 ml fructose solution. With these volumes, about 10 Petri dishes could be theoretically produced for each type of Petri dish (FRU and PURE), which will be definitely sufficient for the needed 6 Petri dishes with fructose-supplemented agarose, and 8 Petri dishes with plain agarose per tandem.
First, the Petri dishes are laid out at the workplace, and all necessary materials are arranged.
Then, a 1% agarose solution is prepared by mixing 100 ml distilled water and 1 g agarose in a glass bottle (labelled ‘PURE’), which is then closed and gently shaken.
6 FRU 8 PURE
Fructose and agarose
Petri dishes
Fill bottle with water, weigh agarose, add agarose

## Slide 5
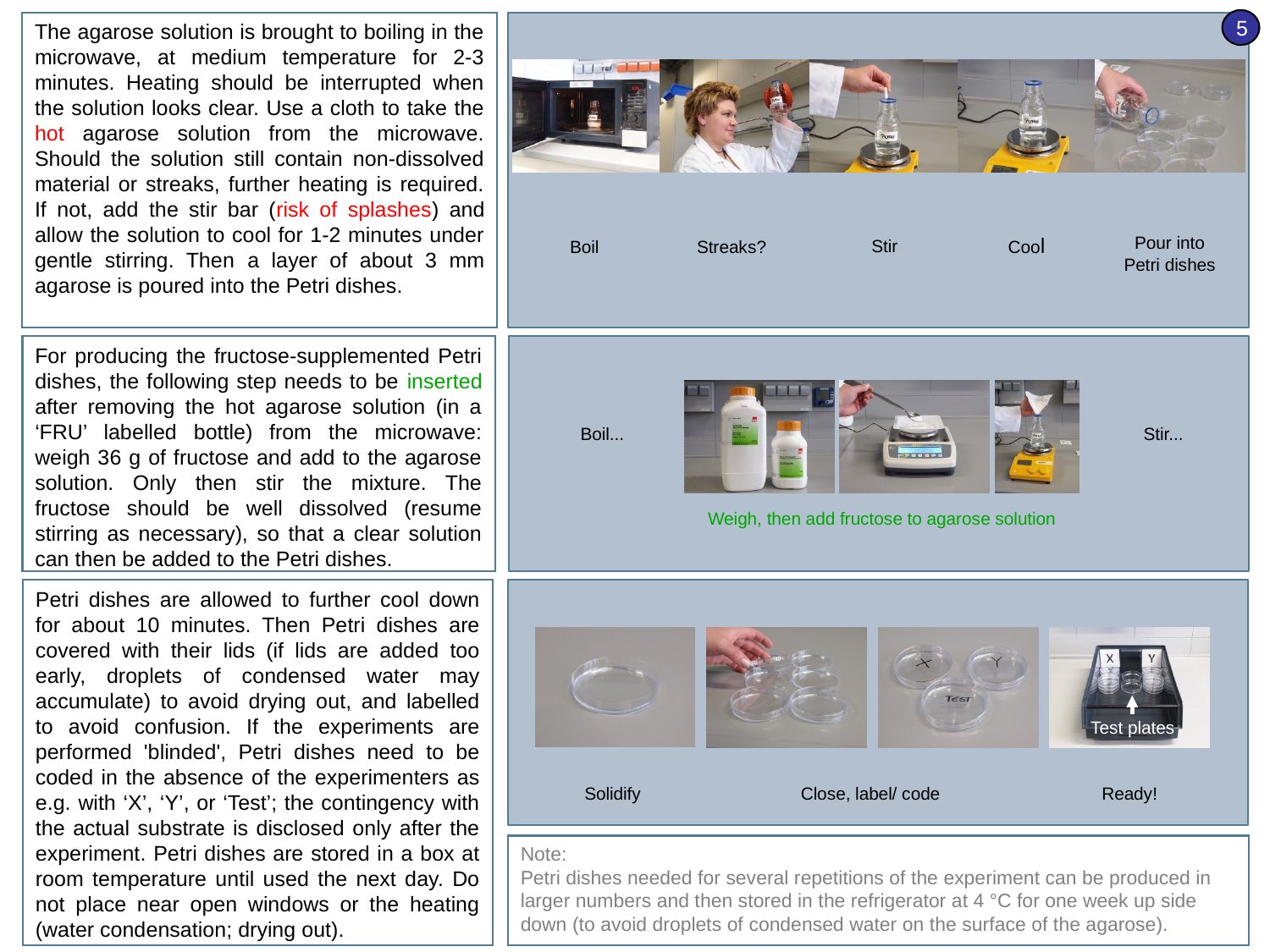

5
The agarose solution is brought to boiling in the microwave, at medium temperature for 2-3 minutes. Heating should be interrupted when the solution looks clear. Use a cloth to take the hot agarose solution from the microwave. Should the solution still contain non-dissolved material or streaks, further heating is required. If not, add the stir bar (risk of splashes) and allow the solution to cool for 1-2 minutes under gentle stirring. Then a layer of about 3 mm agarose is poured into the Petri dishes.
Pour into
Petri dishes
Streaks?
Cool
Stir
Boil
For producing the fructose-supplemented Petri dishes, the following step needs to be inserted after removing the hot agarose solution (in a ‘FRU’ labelled bottle) from the microwave: weigh 36 g of fructose and add to the agarose solution. Only then stir the mixture. The fructose should be well dissolved (resume stirring as necessary), so that a clear solution can then be added to the Petri dishes.
Boil...
Stir...
Weigh, then add fructose to agarose solution
Petri dishes are allowed to further cool down for about 10 minutes. Then Petri dishes are covered with their lids (if lids are added too early, droplets of condensed water may accumulate) to avoid drying out, and labelled to avoid confusion. If the experiments are performed 'blinded', Petri dishes need to be coded in the absence of the experimenters as e.g. with ‘X’, ‘Y’, or ‘Test’; the contingency with the actual substrate is disclosed only after the experiment. Petri dishes are stored in a box at room temperature until used the next day. Do not place near open windows or the heating (water condensation; drying out).
Test plates
Solidify
Close, label/ code
Ready!
Note:
Petri dishes needed for several repetitions of the experiment can be produced in larger numbers and then stored in the refrigerator at 4 °C for one week up side down (to avoid droplets of condensed water on the surface of the agarose).

## Slide 6
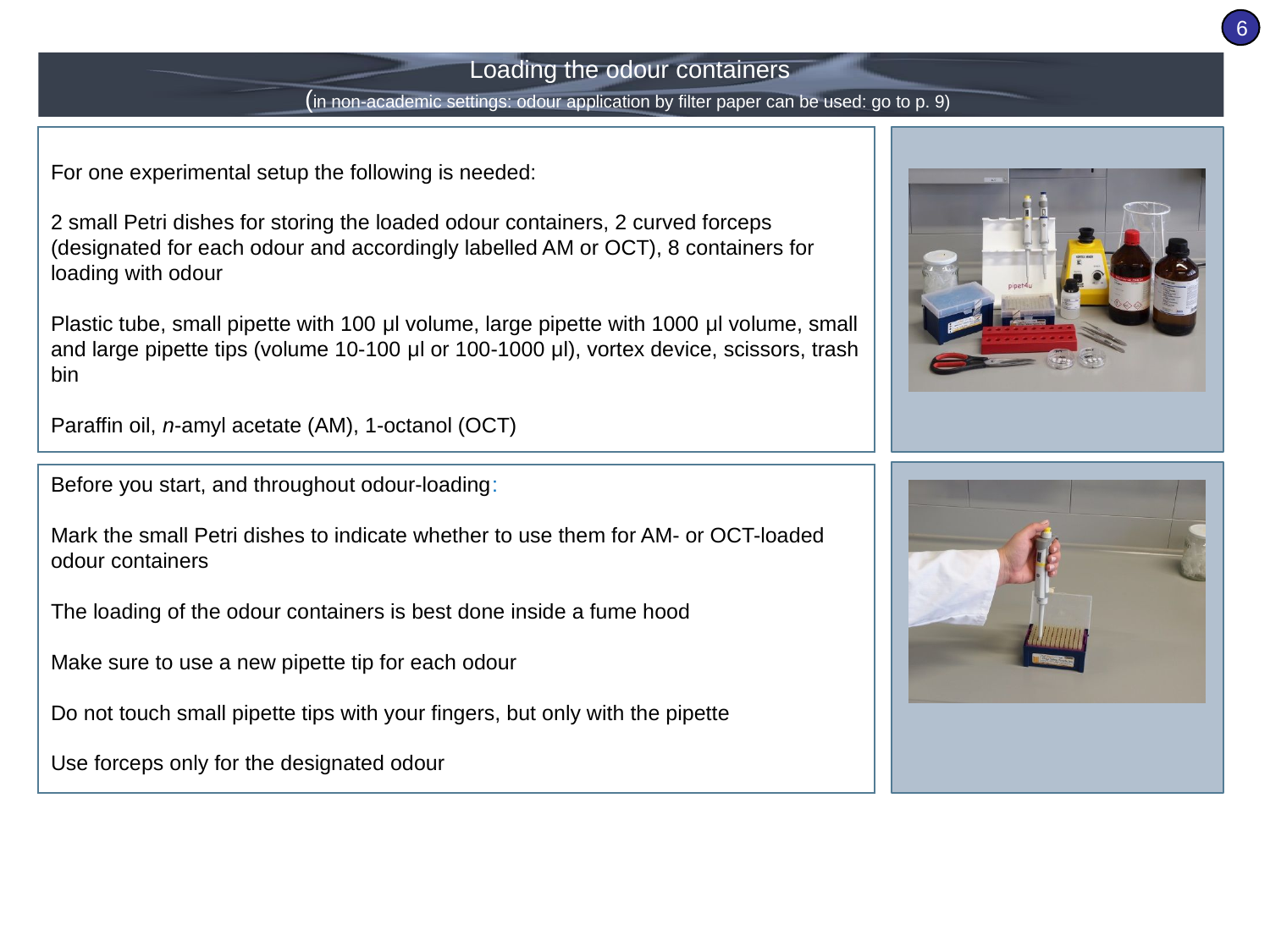

6
# Loading the odour containers (in non-academic settings: odour application by filter paper can be used: go to p. 9)
For one experimental setup the following is needed:
2 small Petri dishes for storing the loaded odour containers, 2 curved forceps (designated for each odour and accordingly labelled AM or OCT), 8 containers for loading with odour
Plastic tube, small pipette with 100 μl volume, large pipette with 1000 μl volume, small and large pipette tips (volume 10-100 μl or 100-1000 μl), vortex device, scissors, trash bin
 
Paraffin oil, n-amyl acetate (AM), 1-octanol (OCT)
Before you start, and throughout odour-loading:
Mark the small Petri dishes to indicate whether to use them for AM- or OCT-loaded odour containers
The loading of the odour containers is best done inside a fume hood
Make sure to use a new pipette tip for each odour
Do not touch small pipette tips with your fingers, but only with the pipette
Use forceps only for the designated odour

## Slide 7
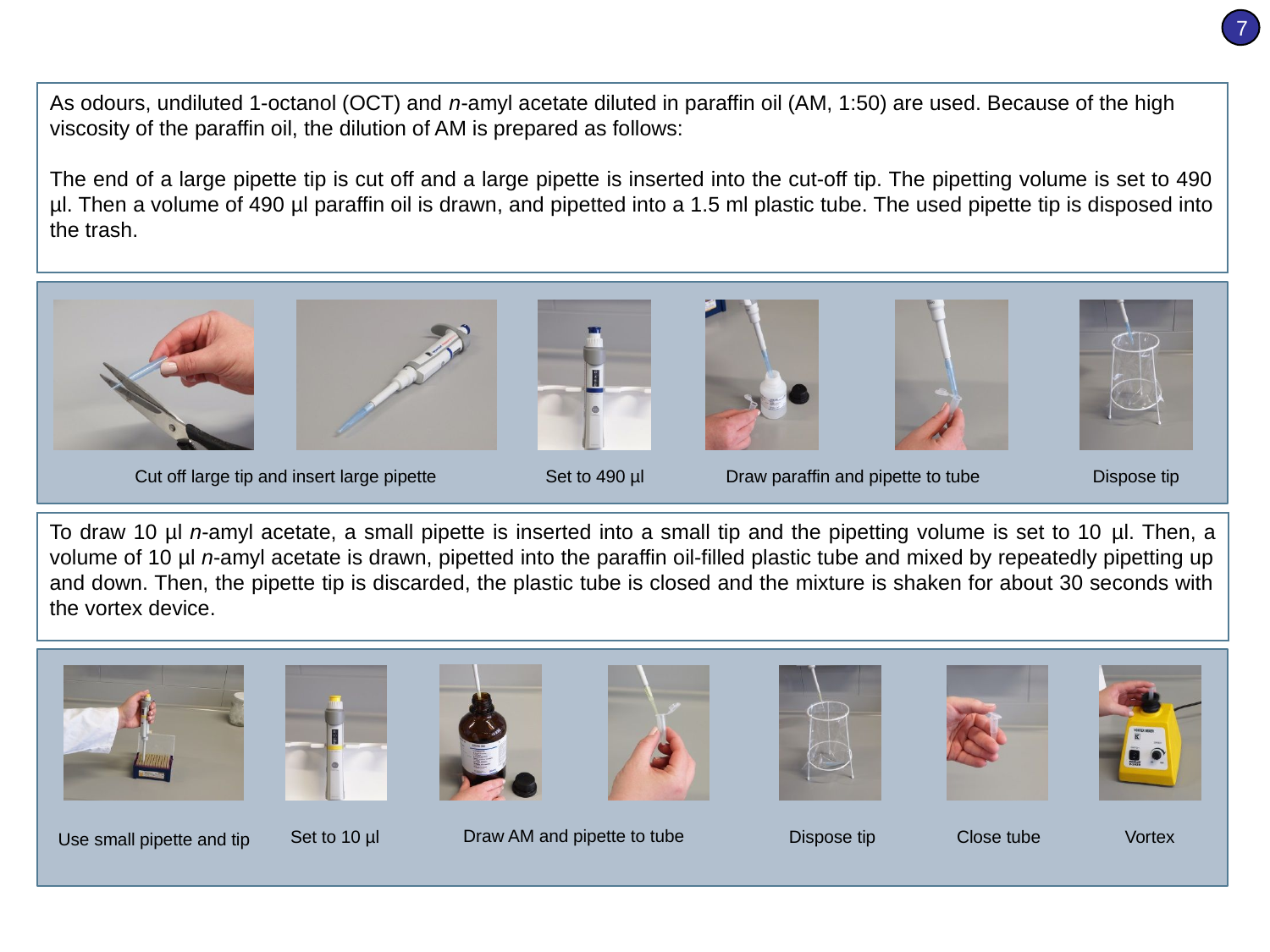

7
As odours, undiluted 1-octanol (OCT) and n-amyl acetate diluted in paraffin oil (AM, 1:50) are used. Because of the high viscosity of the paraffin oil, the dilution of AM is prepared as follows:
The end of a large pipette tip is cut off and a large pipette is inserted into the cut-off tip. The pipetting volume is set to 490 µl. Then a volume of 490 µl paraffin oil is drawn, and pipetted into a 1.5 ml plastic tube. The used pipette tip is disposed into the trash.
Cut off large tip and insert large pipette
Set to 490 µl
Draw paraffin and pipette to tube
Dispose tip
To draw 10 µl n-amyl acetate, a small pipette is inserted into a small tip and the pipetting volume is set to 10 µl. Then, a volume of 10 µl n-amyl acetate is drawn, pipetted into the paraffin oil-filled plastic tube and mixed by repeatedly pipetting up and down. Then, the pipette tip is discarded, the plastic tube is closed and the mixture is shaken for about 30 seconds with the vortex device.
 Use small pipette and tip
Set to 10 µl
Draw AM and pipette to tube
Dispose tip
Close tube
Vortex

## Slide 8
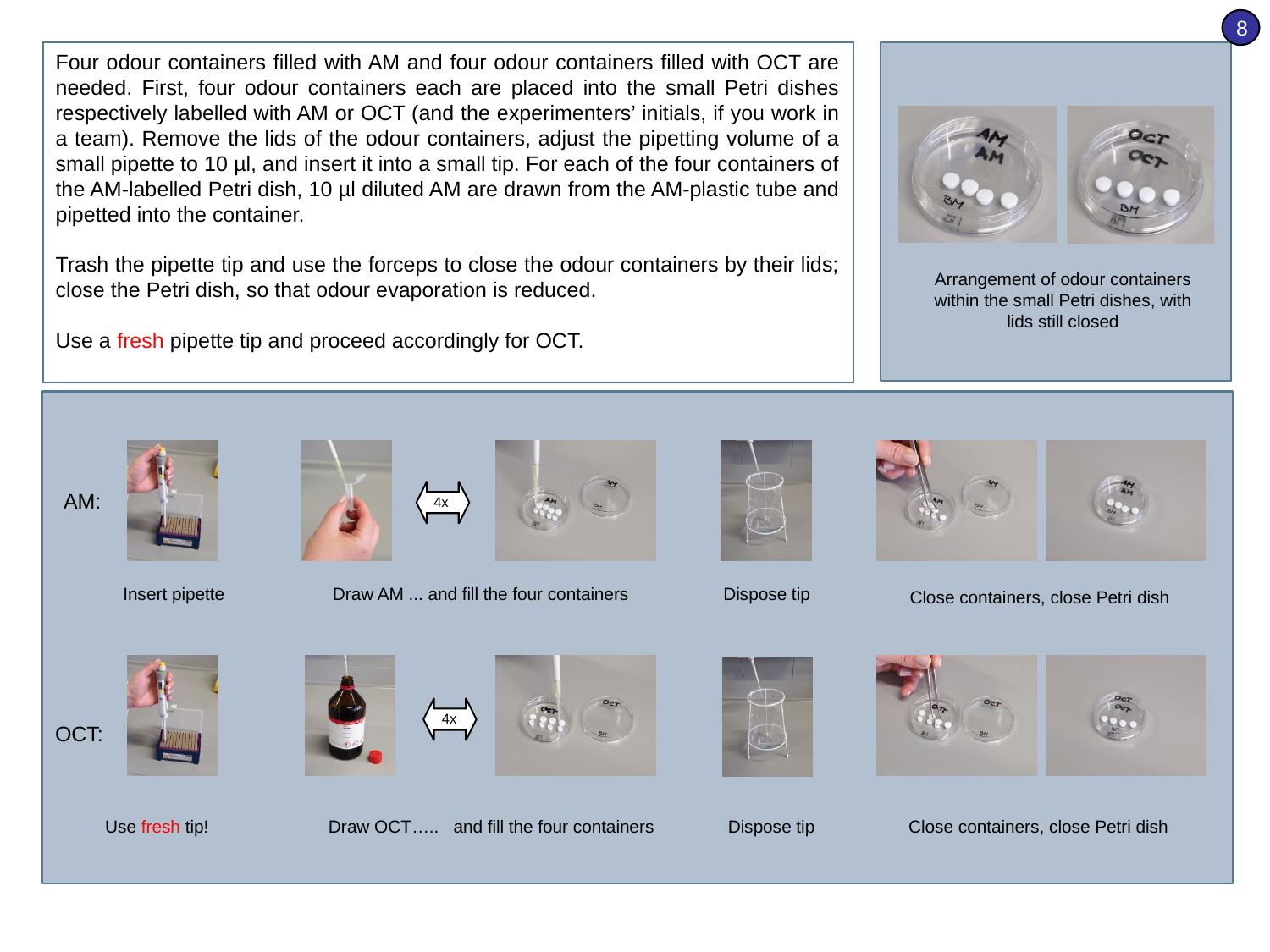

8
Four odour containers filled with AM and four odour containers filled with OCT are needed. First, four odour containers each are placed into the small Petri dishes respectively labelled with AM or OCT (and the experimenters’ initials, if you work in a team). Remove the lids of the odour containers, adjust the pipetting volume of a small pipette to 10 µl, and insert it into a small tip. For each of the four containers of the AM-labelled Petri dish, 10 µl diluted AM are drawn from the AM-plastic tube and pipetted into the container.
Trash the pipette tip and use the forceps to close the odour containers by their lids; close the Petri dish, so that odour evaporation is reduced.
Use a fresh pipette tip and proceed accordingly for OCT.
Arrangement of odour containers within the small Petri dishes, with lids still closed
4x
AM:
Insert pipette
Draw AM ... and fill the four containers
Dispose tip
Close containers, close Petri dish
4x
OCT:
Use fresh tip!
Draw OCT….. and fill the four containers
Dispose tip
Close containers, close Petri dish

## Slide 9
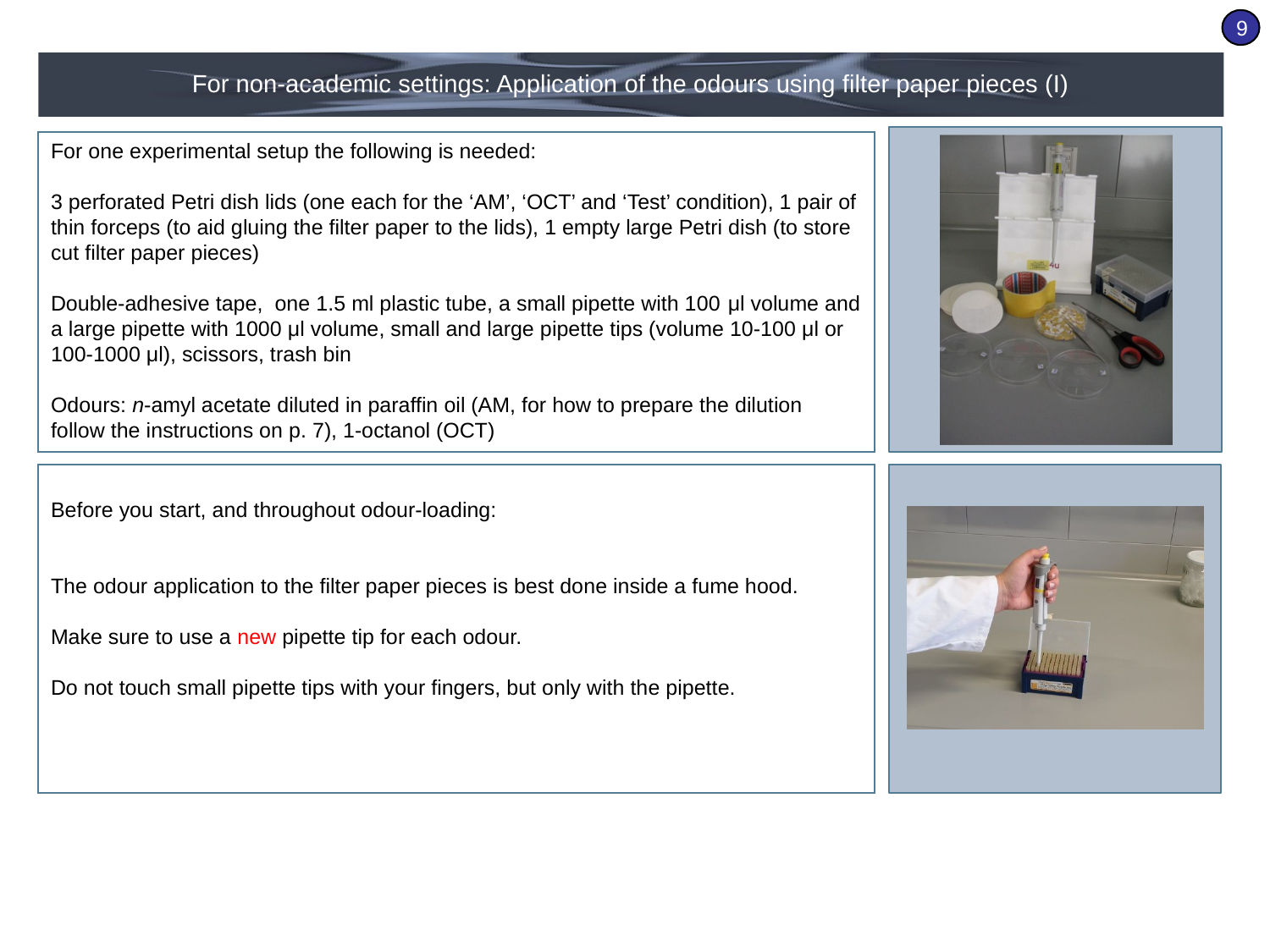

9
# For non-academic settings: Application of the odours using filter paper pieces (I)
For one experimental setup the following is needed:
3 perforated Petri dish lids (one each for the ‘AM’, ‘OCT’ and ‘Test’ condition), 1 pair of thin forceps (to aid gluing the filter paper to the lids), 1 empty large Petri dish (to store cut filter paper pieces)
Double-adhesive tape, one 1.5 ml plastic tube, a small pipette with 100 μl volume and a large pipette with 1000 μl volume, small and large pipette tips (volume 10-100 μl or 100-1000 μl), scissors, trash bin
 
Odours: n-amyl acetate diluted in paraffin oil (AM, for how to prepare the dilution follow the instructions on p. 7), 1-octanol (OCT)
Before you start, and throughout odour-loading:
The odour application to the filter paper pieces is best done inside a fume hood.
Make sure to use a new pipette tip for each odour.
Do not touch small pipette tips with your fingers, but only with the pipette.

## Slide 10
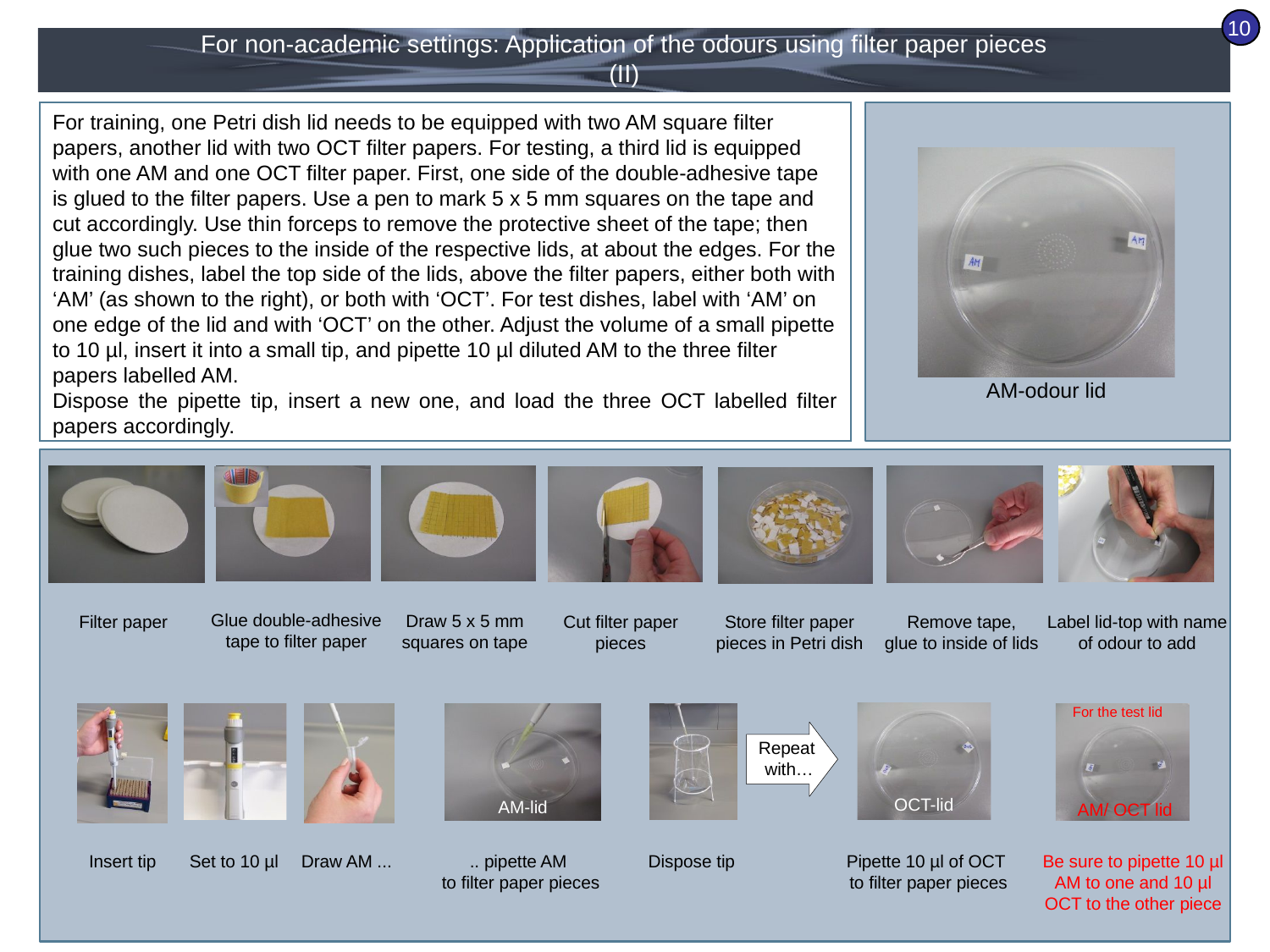

10
# For non-academic settings: Application of the odours using filter paper pieces (II)
For training, one Petri dish lid needs to be equipped with two AM square filter papers, another lid with two OCT filter papers. For testing, a third lid is equipped with one AM and one OCT filter paper. First, one side of the double-adhesive tape is glued to the filter papers. Use a pen to mark 5 x 5 mm squares on the tape and cut accordingly. Use thin forceps to remove the protective sheet of the tape; then glue two such pieces to the inside of the respective lids, at about the edges. For the training dishes, label the top side of the lids, above the filter papers, either both with ‘AM’ (as shown to the right), or both with ‘OCT’. For test dishes, label with ‘AM’ on one edge of the lid and with ‘OCT’ on the other. Adjust the volume of a small pipette to 10 µl, insert it into a small tip, and pipette 10 µl diluted AM to the three filter papers labelled AM.
Dispose the pipette tip, insert a new one, and load the three OCT labelled filter papers accordingly.
AM-odour lid
Glue double-adhesive
tape to filter paper
Draw 5 x 5 mm
squares on tape
Filter paper
Cut filter paper pieces
Store filter paper pieces in Petri dish
Remove tape,
glue to inside of lids
Label lid-top with name of odour to add
For the test lid
Repeat
with…
OCT-lid
AM-lid
AM/ OCT lid
Insert tip
Set to 10 µl
Draw AM ...
.. pipette AM
 to filter paper pieces
Dispose tip
Pipette 10 µl of OCT
 to filter paper pieces
Be sure to pipette 10 µl AM to one and 10 µl OCT to the other piece

## Slide 11
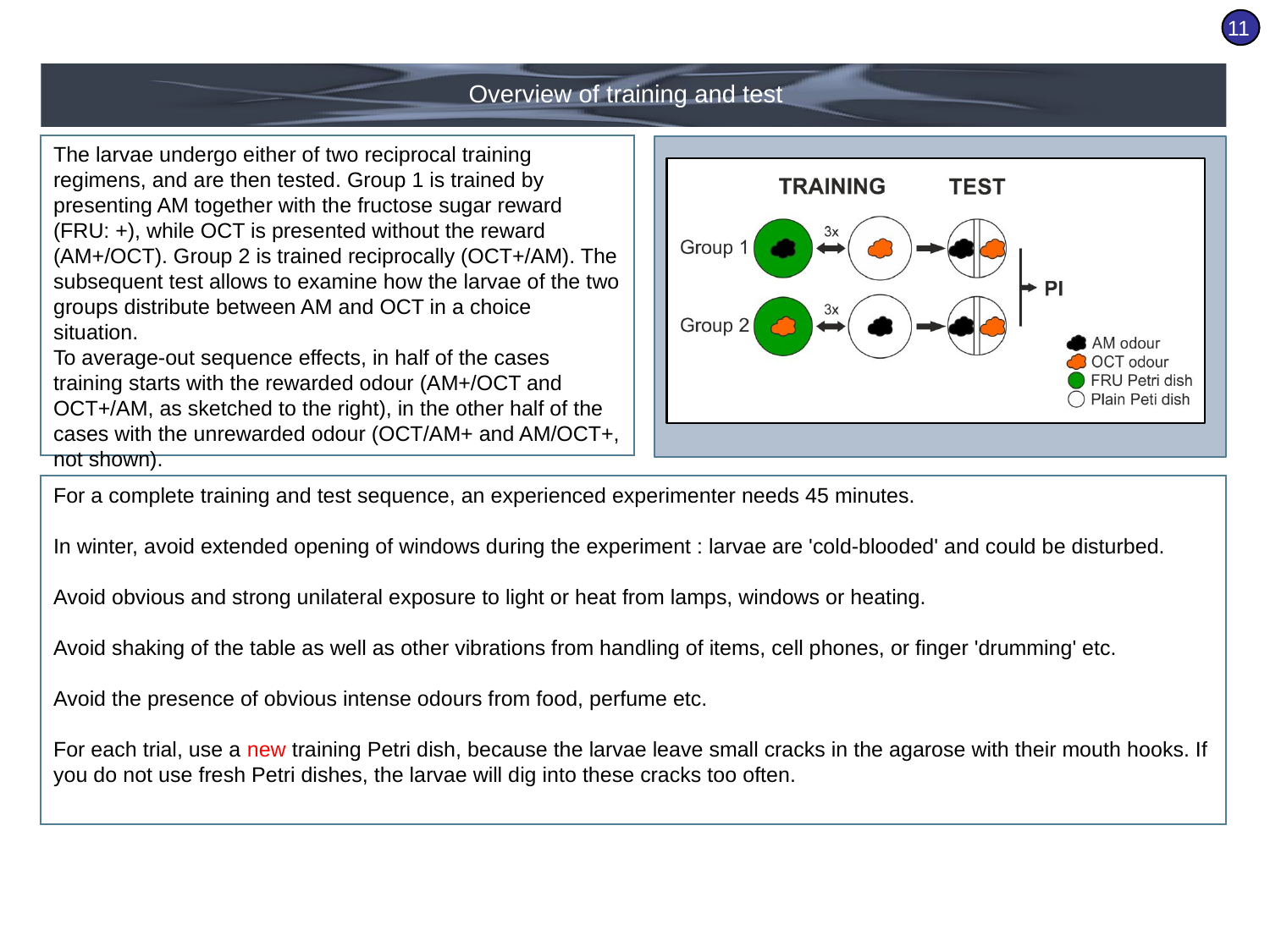

11
Overview of training and test
The larvae undergo either of two reciprocal training regimens, and are then tested. Group 1 is trained by presenting AM together with the fructose sugar reward (FRU: +), while OCT is presented without the reward (AM+/OCT). Group 2 is trained reciprocally (OCT+/AM). The subsequent test allows to examine how the larvae of the two groups distribute between AM and OCT in a choice situation.
To average-out sequence effects, in half of the cases training starts with the rewarded odour (AM+/OCT and OCT+/AM, as sketched to the right), in the other half of the cases with the unrewarded odour (OCT/AM+ and AM/OCT+, not shown).
For a complete training and test sequence, an experienced experimenter needs 45 minutes.
In winter, avoid extended opening of windows during the experiment : larvae are 'cold-blooded' and could be disturbed.
Avoid obvious and strong unilateral exposure to light or heat from lamps, windows or heating.
Avoid shaking of the table as well as other vibrations from handling of items, cell phones, or finger 'drumming' etc.
Avoid the presence of obvious intense odours from food, perfume etc.
For each trial, use a new training Petri dish, because the larvae leave small cracks in the agarose with their mouth hooks. If you do not use fresh Petri dishes, the larvae will dig into these cracks too often.

## Slide 12
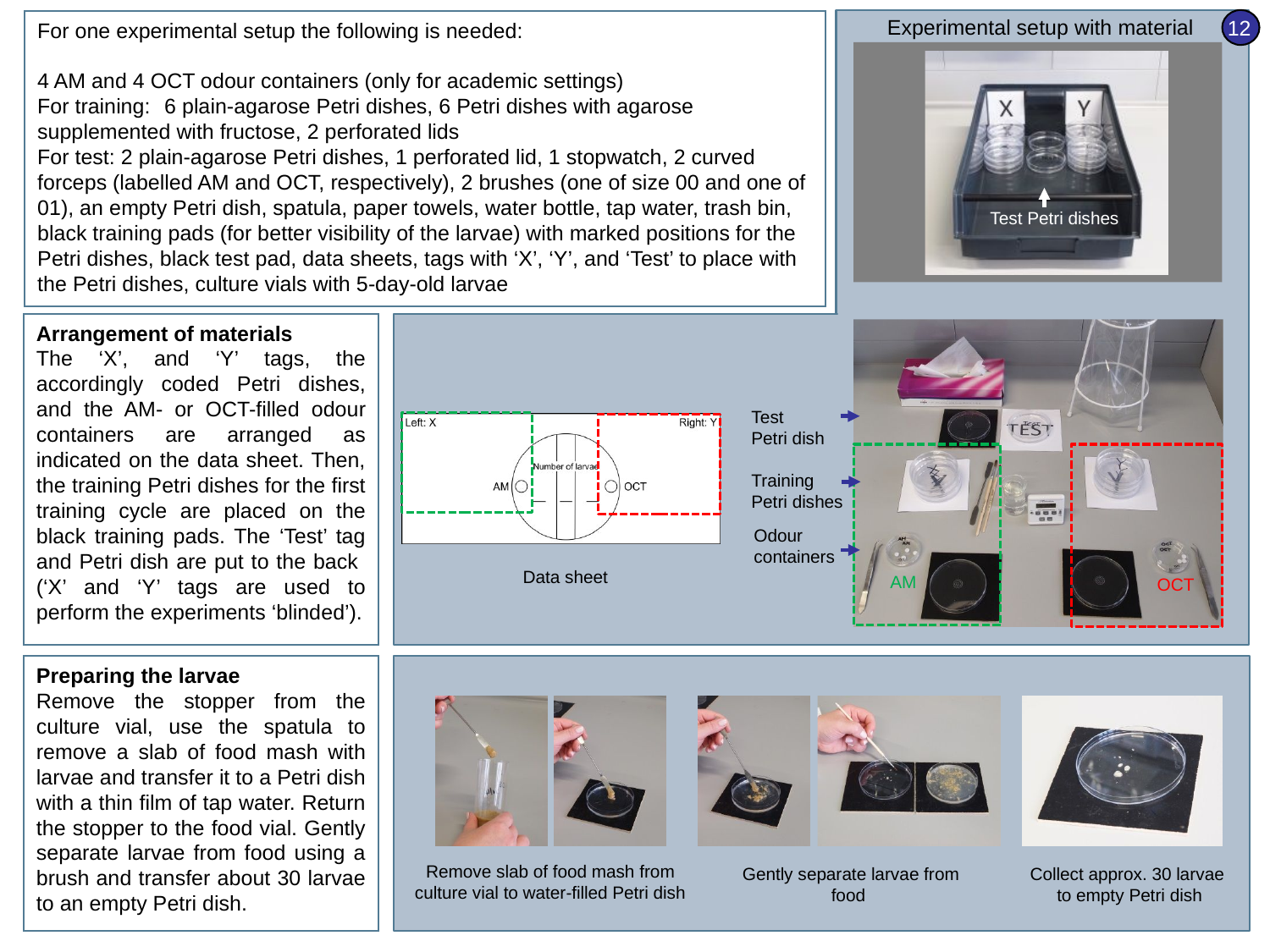

Experimental setup with material
12
For one experimental setup the following is needed:
4 AM and 4 OCT odour containers (only for academic settings)
For training:	6 plain-agarose Petri dishes, 6 Petri dishes with agarose supplemented with fructose, 2 perforated lids
For test: 2 plain-agarose Petri dishes, 1 perforated lid, 1 stopwatch, 2 curved forceps (labelled AM and OCT, respectively), 2 brushes (one of size 00 and one of 01), an empty Petri dish, spatula, paper towels, water bottle, tap water, trash bin, black training pads (for better visibility of the larvae) with marked positions for the Petri dishes, black test pad, data sheets, tags with ‘X’, ‘Y’, and ‘Test’ to place with the Petri dishes, culture vials with 5-day-old larvae
Test Petri dishes
Arrangement of materials
The ‘X’, and ‘Y’ tags, the accordingly coded Petri dishes, and the AM- or OCT-filled odour containers are arranged as indicated on the data sheet. Then, the training Petri dishes for the first training cycle are placed on the black training pads. The ‘Test’ tag and Petri dish are put to the back (‘X’ and ‘Y’ tags are used to perform the experiments ‘blinded’).
Test
Petri dish
OCT
Y
Training
Petri dishes
Odour
containers
Data sheet
AM
OCT
Preparing the larvae
Remove the stopper from the culture vial, use the spatula to remove a slab of food mash with larvae and transfer it to a Petri dish with a thin film of tap water. Return the stopper to the food vial. Gently separate larvae from food using a brush and transfer about 30 larvae to an empty Petri dish.
Remove slab of food mash from culture vial to water-filled Petri dish
Gently separate larvae from food
Collect approx. 30 larvae
 to empty Petri dish

## Slide 13
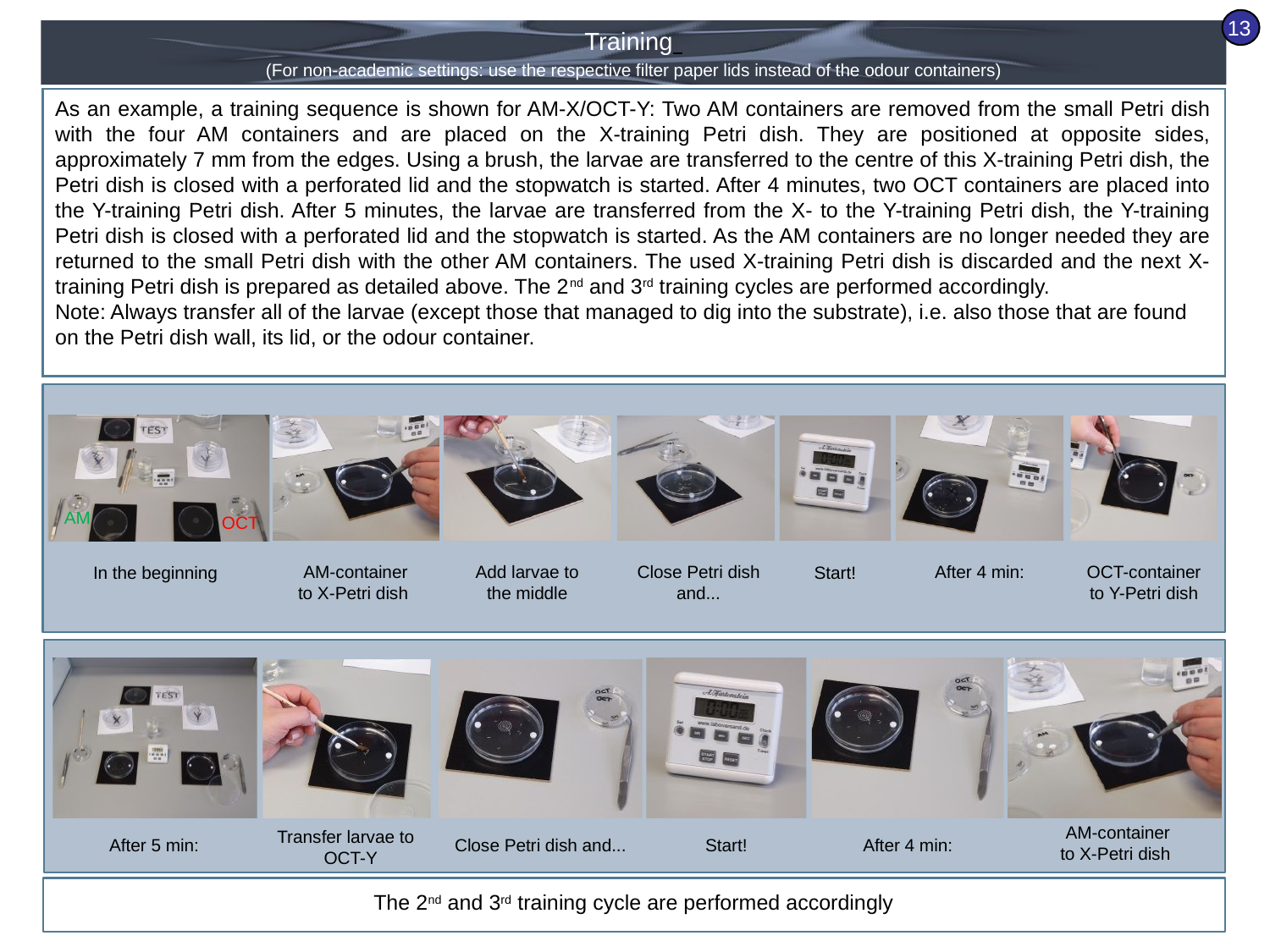

13
# Training
(For non-academic settings: use the respective filter paper lids instead of the odour containers)
As an example, a training sequence is shown for AM-X/OCT-Y: Two AM containers are removed from the small Petri dish with the four AM containers and are placed on the X-training Petri dish. They are positioned at opposite sides, approximately 7 mm from the edges. Using a brush, the larvae are transferred to the centre of this X-training Petri dish, the Petri dish is closed with a perforated lid and the stopwatch is started. After 4 minutes, two OCT containers are placed into the Y-training Petri dish. After 5 minutes, the larvae are transferred from the X- to the Y-training Petri dish, the Y-training Petri dish is closed with a perforated lid and the stopwatch is started. As the AM containers are no longer needed they are returned to the small Petri dish with the other AM containers. The used X-training Petri dish is discarded and the next X-training Petri dish is prepared as detailed above. The 2nd and 3rd training cycles are performed accordingly.
Note: Always transfer all of the larvae (except those that managed to dig into the substrate), i.e. also those that are found on the Petri dish wall, its lid, or the odour container.
AM
OCT
AM-container
to X-Petri dish
After 4 min:
Add larvae to
the middle
Close Petri dish and...
OCT-container
to Y-Petri dish
In the beginning
Start!
AM-container
to X-Petri dish
Transfer larvae to OCT-Y
Close Petri dish and...
After 5 min:
Start!
After 4 min:
The 2nd and 3rd training cycle are performed accordingly

## Slide 14
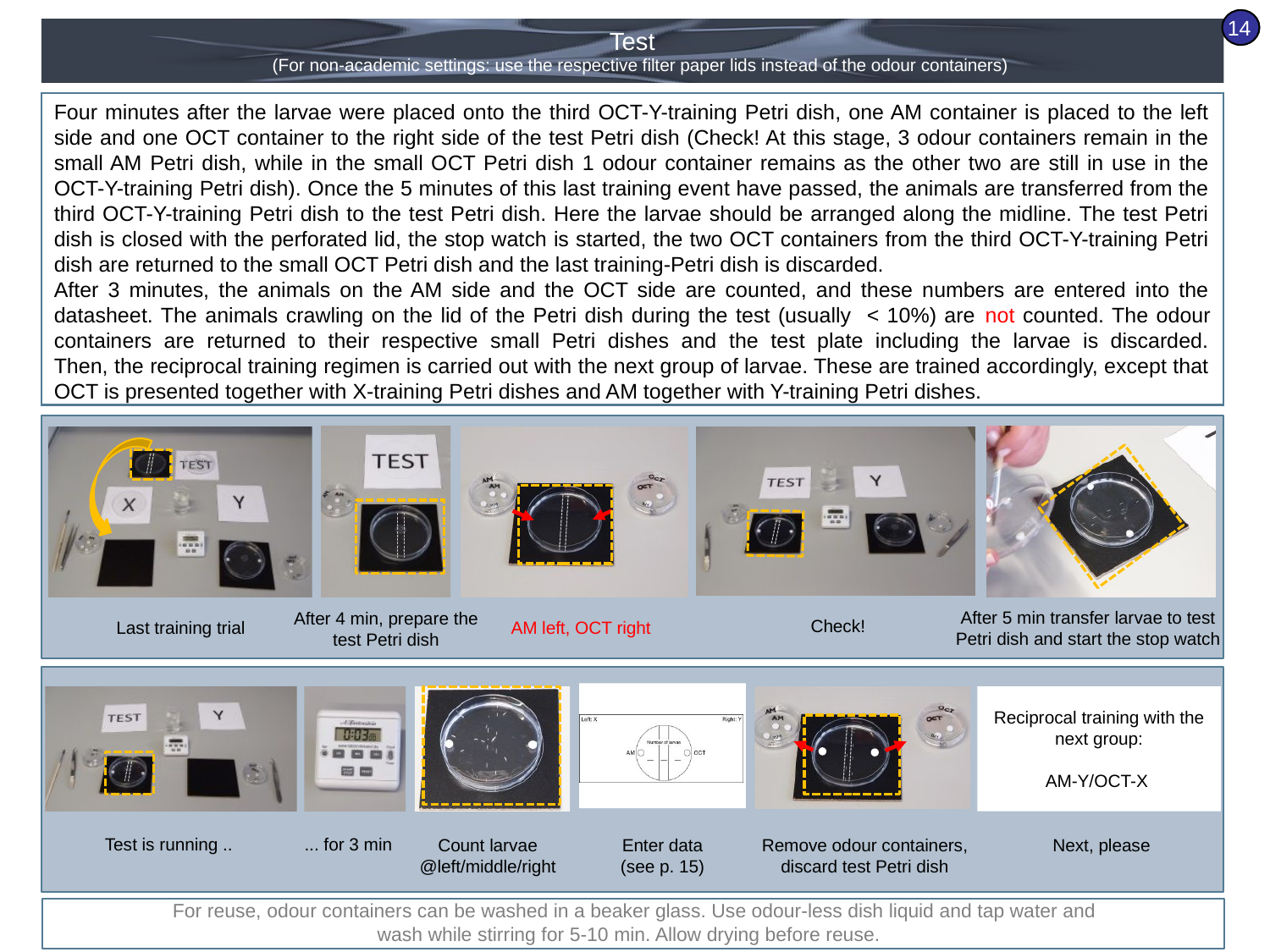

# Test
14
(For non-academic settings: use the respective filter paper lids instead of the odour containers)
Four minutes after the larvae were placed onto the third OCT-Y-training Petri dish, one AM container is placed to the left side and one OCT container to the right side of the test Petri dish (Check! At this stage, 3 odour containers remain in the small AM Petri dish, while in the small OCT Petri dish 1 odour container remains as the other two are still in use in the OCT-Y-training Petri dish). Once the 5 minutes of this last training event have passed, the animals are transferred from the third OCT-Y-training Petri dish to the test Petri dish. Here the larvae should be arranged along the midline. The test Petri dish is closed with the perforated lid, the stop watch is started, the two OCT containers from the third OCT-Y-training Petri dish are returned to the small OCT Petri dish and the last training-Petri dish is discarded.
After 3 minutes, the animals on the AM side and the OCT side are counted, and these numbers are entered into the datasheet. The animals crawling on the lid of the Petri dish during the test (usually < 10%) are not counted. The odour containers are returned to their respective small Petri dishes and the test plate including the larvae is discarded.
Then, the reciprocal training regimen is carried out with the next group of larvae. These are trained accordingly, except that OCT is presented together with X-training Petri dishes and AM together with Y-training Petri dishes.
After 5 min transfer larvae to test
 Petri dish and start the stop watch
After 4 min, prepare the
test Petri dish
Check!
AM left, OCT right
Last training trial
Reciprocal training with the next group:
AM-Y/OCT-X
Test is running ..
... for 3 min
Count larvae
@left/middle/right
Next, please
Enter data
(see p. 15)
Remove odour containers, discard test Petri dish
For reuse, odour containers can be washed in a beaker glass. Use odour-less dish liquid and tap water and
wash while stirring for 5-10 min. Allow drying before reuse.

## Slide 15
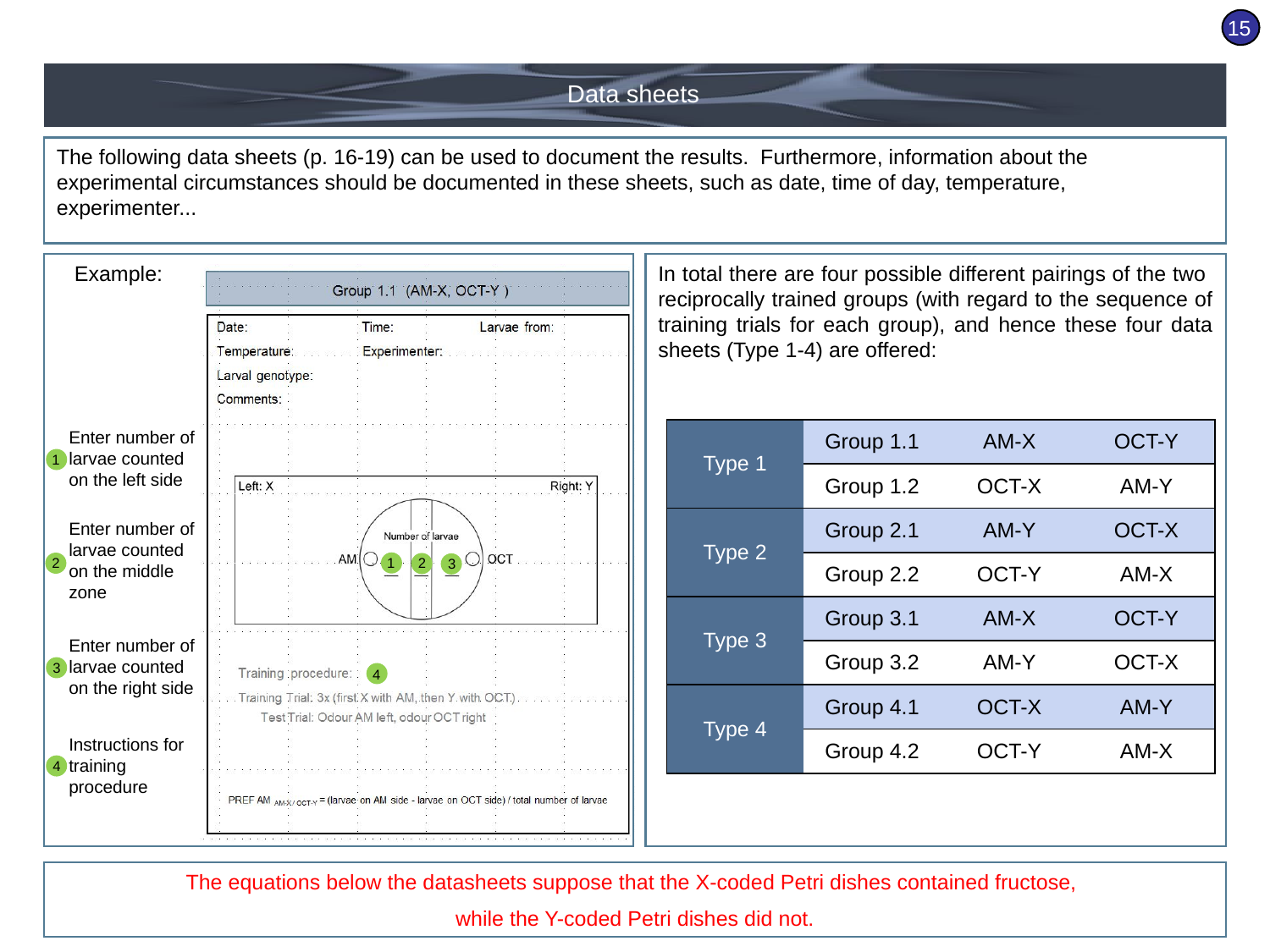

15
Data sheets
The following data sheets (p. 16-19) can be used to document the results. Furthermore, information about the experimental circumstances should be documented in these sheets, such as date, time of day, temperature, experimenter...
 Example:
In total there are four possible different pairings of the two reciprocally trained groups (with regard to the sequence of training trials for each group), and hence these four data sheets (Type 1-4) are offered:
| Type 1 | Group 1.1 | AM-X | OCT-Y |
| --- | --- | --- | --- |
| | Group 1.2 | OCT-X | AM-Y |
| Type 2 | Group 2.1 | AM-Y | OCT-X |
| | Group 2.2 | OCT-Y | AM-X |
| Type 3 | Group 3.1 | AM-X | OCT-Y |
| | Group 3.2 | AM-Y | OCT-X |
| Type 4 | Group 4.1 | OCT-X | AM-Y |
| | Group 4.2 | OCT-Y | AM-X |
Enter number of larvae counted on the left side
1
Enter number of larvae counted on the middle zone
1
2
2
3
Enter number of larvae counted on the right side
3
4
Instructions for training procedure
4
The equations below the datasheets suppose that the X-coded Petri dishes contained fructose,
while the Y-coded Petri dishes did not.

## Slide 16
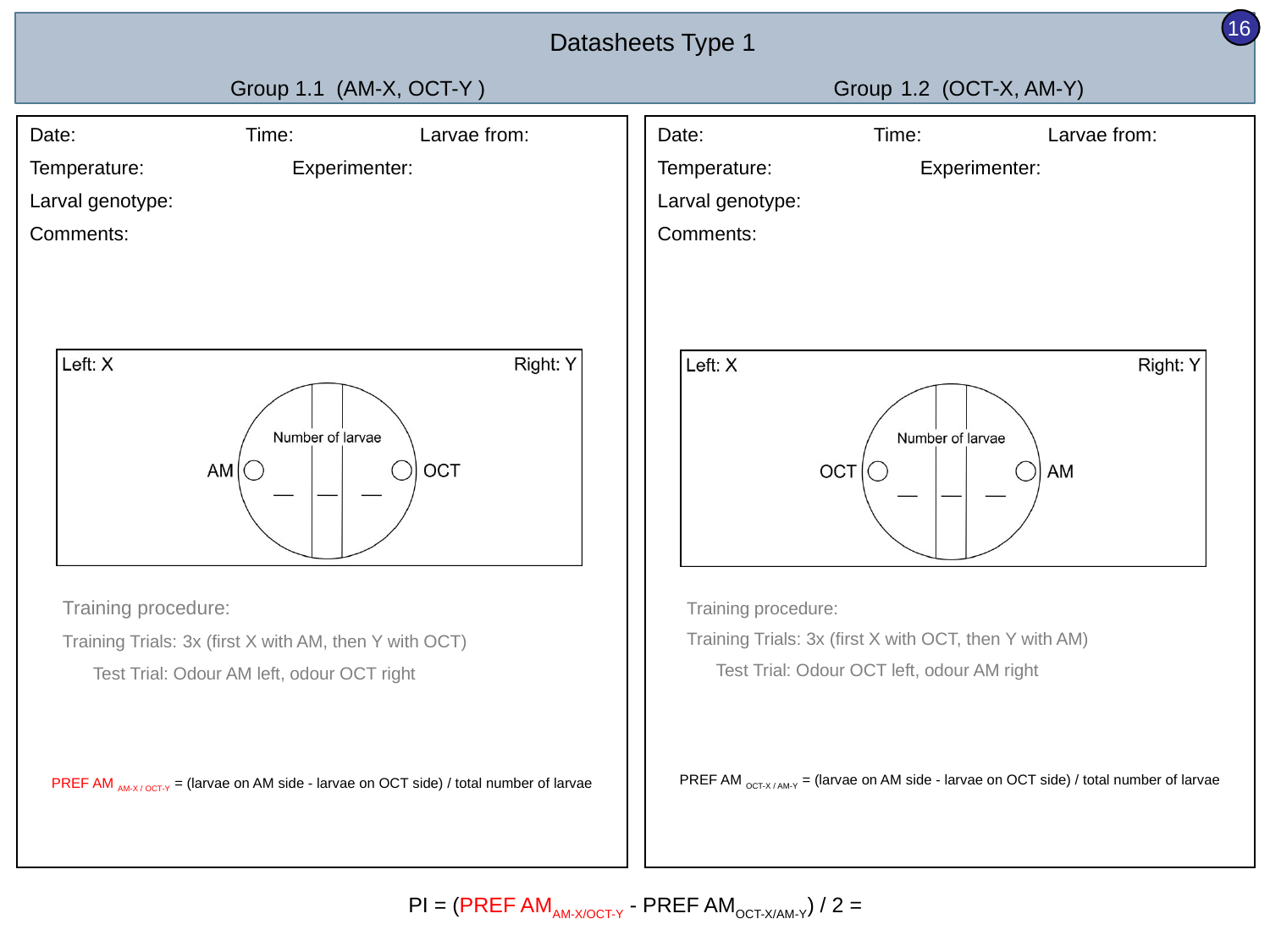

16
# Datasheets Type 1 Group 1.1 (AM-X, OCT-Y ) Group 1.2 (OCT-X, AM-Y)
Date: Time: Larvae from:
Temperature: 	 Experimenter:
Larval genotype:
Comments:
 Training procedure:
 Training Trials: 3x (first X with AM, then Y with OCT)
 Test Trial: Odour AM left, odour OCT right
PREF AM AM-X / OCT-Y = (larvae on AM side - larvae on OCT side) / total number of larvae
Date: Time: Larvae from:
Temperature: 	 Experimenter:
Larval genotype:
Comments:
 Training procedure:
 Training Trials: 3x (first X with OCT, then Y with AM)
 Test Trial: Odour OCT left, odour AM right
PREF AM OCT-X / AM-Y = (larvae on AM side - larvae on OCT side) / total number of larvae
PI = (PREF AMAM-X/OCT-Y - PREF AMOCT-X/AM-Y) / 2 =

## Slide 17
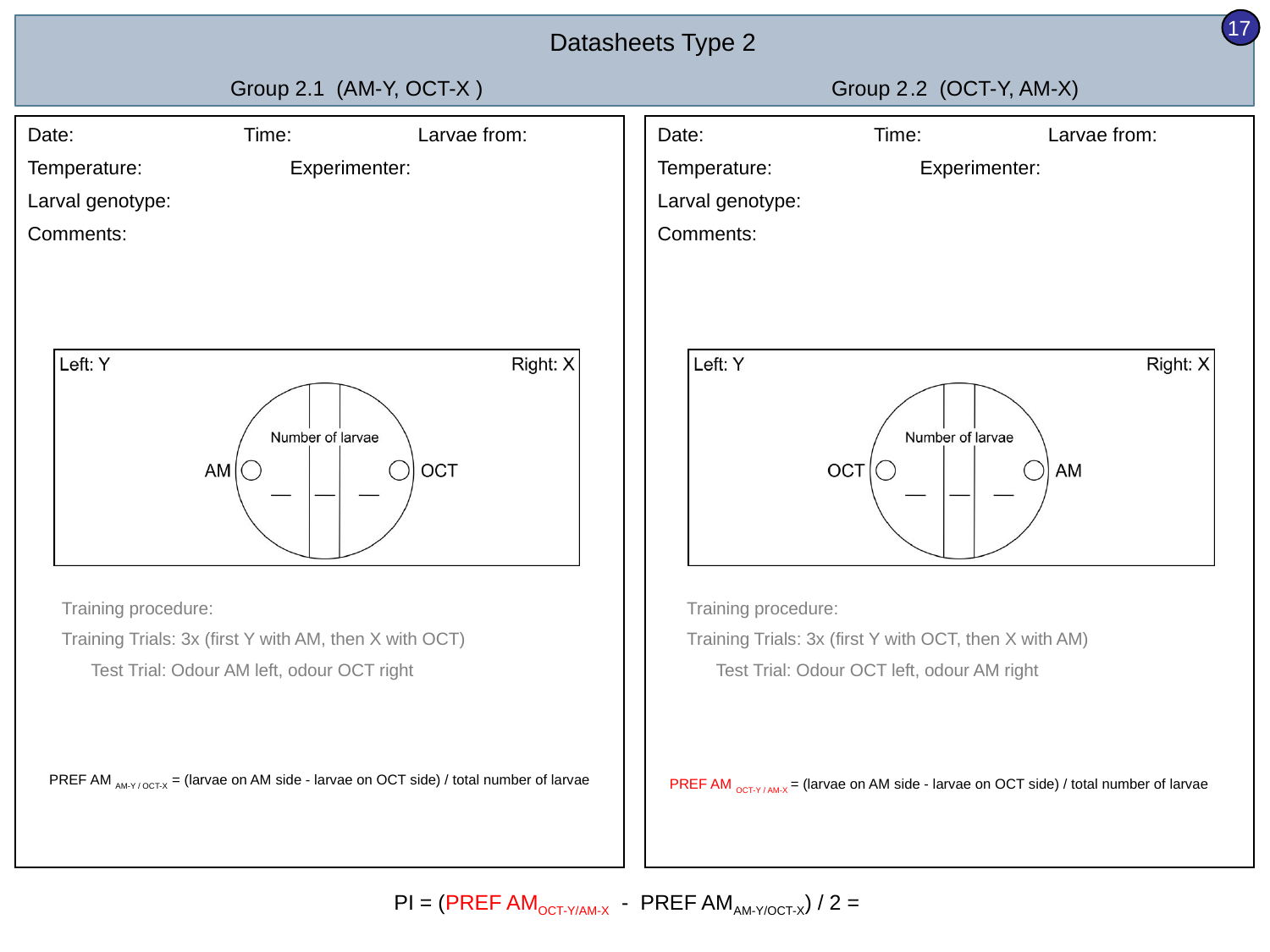

17
				 Datasheets Type 2
 Group 2.1 (AM-Y, OCT-X ) Group 2.2 (OCT-Y, AM-X)
Date: Time: Larvae from:
Temperature: 	 Experimenter:
Larval genotype:
Comments:
 Training procedure:
 Training Trials: 3x (first Y with AM, then X with OCT)
 Test Trial: Odour AM left, odour OCT right
PREF AM AM-Y / OCT-X = (larvae on AM side - larvae on OCT side) / total number of larvae
Date: Time: Larvae from:
Temperature: 	 Experimenter:
Larval genotype:
Comments:
 Training procedure:
 Training Trials: 3x (first Y with OCT, then X with AM)
 Test Trial: Odour OCT left, odour AM right
 PREF AM OCT-Y / AM-X = (larvae on AM side - larvae on OCT side) / total number of larvae
PI = (PREF AMOCT-Y/AM-X - PREF AMAM-Y/OCT-X) / 2 =

## Slide 18
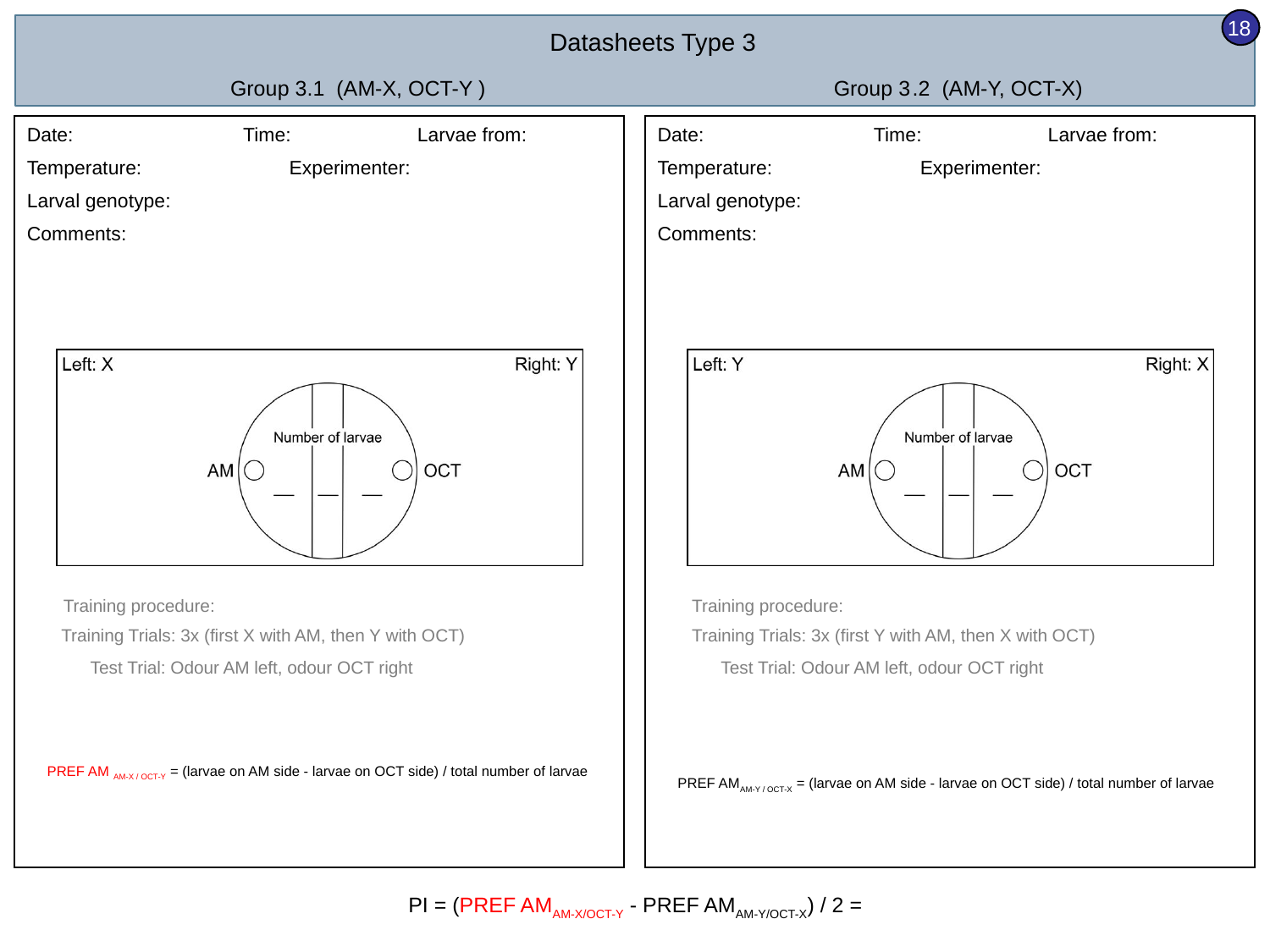

18
				 Datasheets Type 3
 Group 3.1 (AM-X, OCT-Y ) Group 3.2 (AM-Y, OCT-X)
Date: Time: Larvae from:
Temperature: 	 Experimenter:
Larval genotype:
Comments:
 Training procedure:
 Training Trials: 3x (first X with AM, then Y with OCT)
 Test Trial: Odour AM left, odour OCT right
 PREF AM AM-X / OCT-Y = (larvae on AM side - larvae on OCT side) / total number of larvae
Date: Time: Larvae from:
Temperature: 	 Experimenter:
Larval genotype:
Comments:
 Training procedure:
 Training Trials: 3x (first Y with AM, then X with OCT)
 Test Trial: Odour AM left, odour OCT right
 PREF AMAM-Y / OCT-X = (larvae on AM side - larvae on OCT side) / total number of larvae
PI = (PREF AMAM-X/OCT-Y - PREF AMAM-Y/OCT-X) / 2 =

## Slide 19
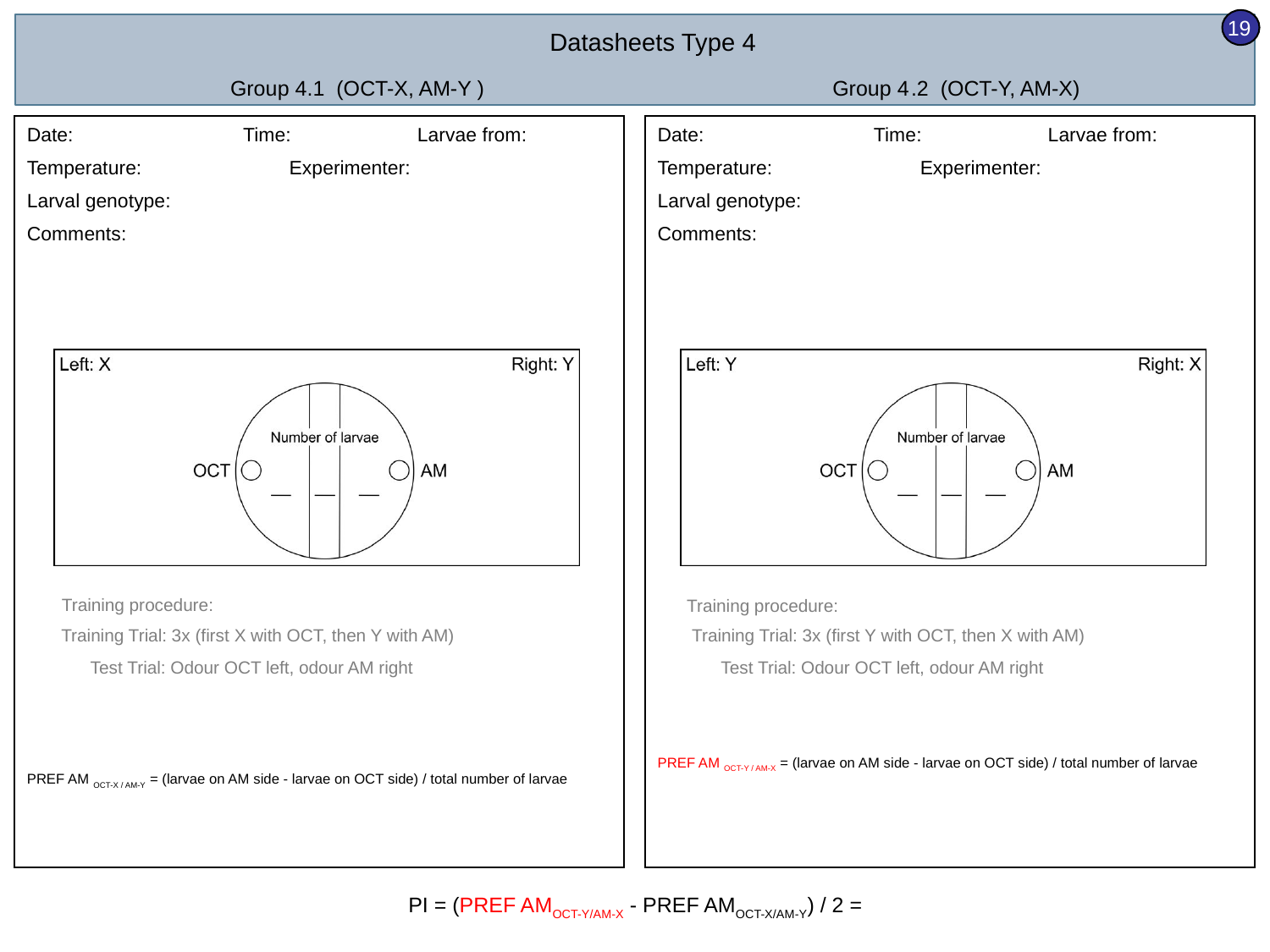

19
				 Datasheets Type 4
 Group 4.1 (OCT-X, AM-Y ) Group 4.2 (OCT-Y, AM-X)
Date: Time: Larvae from:
Temperature: 	 Experimenter:
Larval genotype:
Comments:
 Training procedure:
 Training Trial: 3x (first X with OCT, then Y with AM)
 Test Trial: Odour OCT left, odour AM right
PREF AM OCT-X / AM-Y = (larvae on AM side - larvae on OCT side) / total number of larvae
Date: Time: Larvae from:
Temperature: 	 Experimenter:
Larval genotype:
Comments:
 Training procedure:
 Training Trial: 3x (first Y with OCT, then X with AM)
 Test Trial: Odour OCT left, odour AM right
PREF AM OCT-Y / AM-X = (larvae on AM side - larvae on OCT side) / total number of larvae
PI = (PREF AMOCT-Y/AM-X - PREF AMOCT-X/AM-Y) / 2 =

## Slide 20
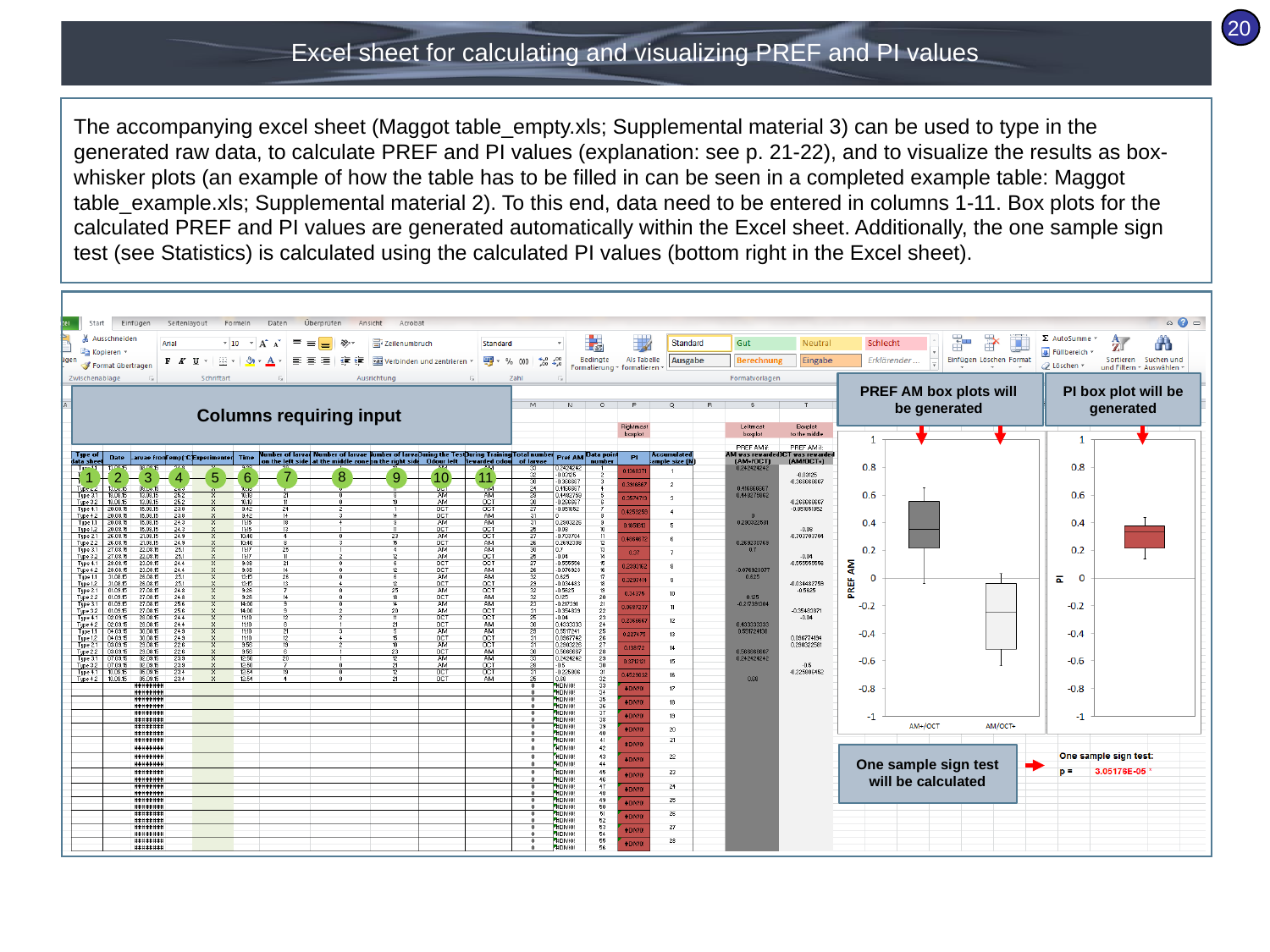

20
Excel sheet for calculating and visualizing PREF and PI values
The accompanying excel sheet (Maggot table_empty.xls; Supplemental material 3) can be used to type in the generated raw data, to calculate PREF and PI values (explanation: see p. 21-22), and to visualize the results as box-whisker plots (an example of how the table has to be filled in can be seen in a completed example table: Maggot table_example.xls; Supplemental material 2). To this end, data need to be entered in columns 1-11. Box plots for the calculated PREF and PI values are generated automatically within the Excel sheet. Additionally, the one sample sign test (see Statistics) is calculated using the calculated PI values (bottom right in the Excel sheet).
PREF AM box plots will be generated
PI box plot will be generated
Columns requiring input
7
8
3
4
2
5
6
9
10
1
11
One sample sign test will be calculated

## Slide 21
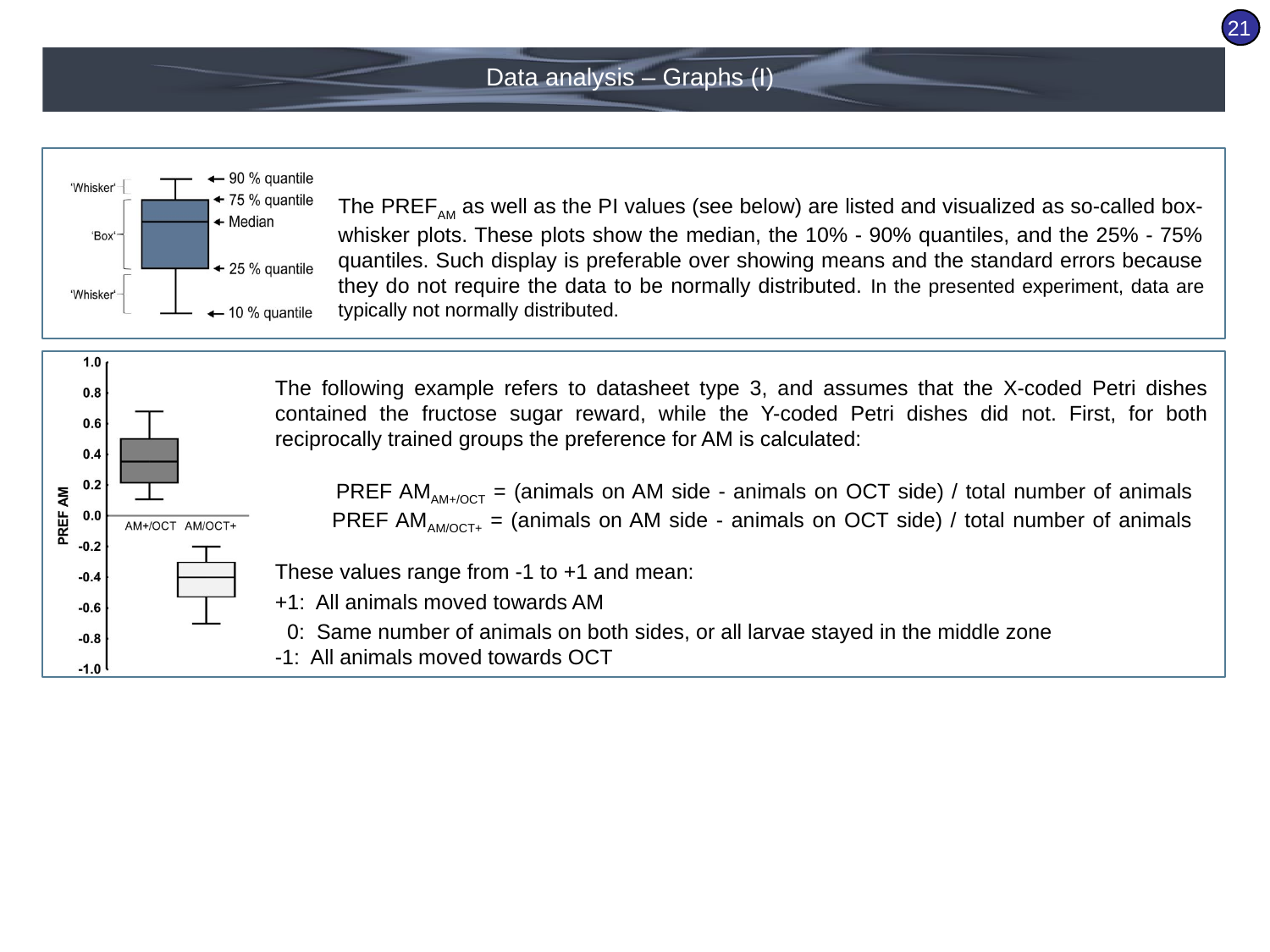

21
Data analysis – Graphs (I)
The PREFAM as well as the PI values (see below) are listed and visualized as so-called box-whisker plots. These plots show the median, the 10% - 90% quantiles, and the 25% - 75% quantiles. Such display is preferable over showing means and the standard errors because they do not require the data to be normally distributed. In the presented experiment, data are typically not normally distributed.
The following example refers to datasheet type 3, and assumes that the X-coded Petri dishes contained the fructose sugar reward, while the Y-coded Petri dishes did not. First, for both reciprocally trained groups the preference for AM is calculated:
 PREF AMAM+/OCT = (animals on AM side - animals on OCT side) / total number of animals
 PREF AMAM/OCT+ = (animals on AM side - animals on OCT side) / total number of animals
These values range from -1 to +1 and mean:
+1: All animals moved towards AM
 0: Same number of animals on both sides, or all larvae stayed in the middle zone
-1: All animals moved towards OCT

## Slide 22
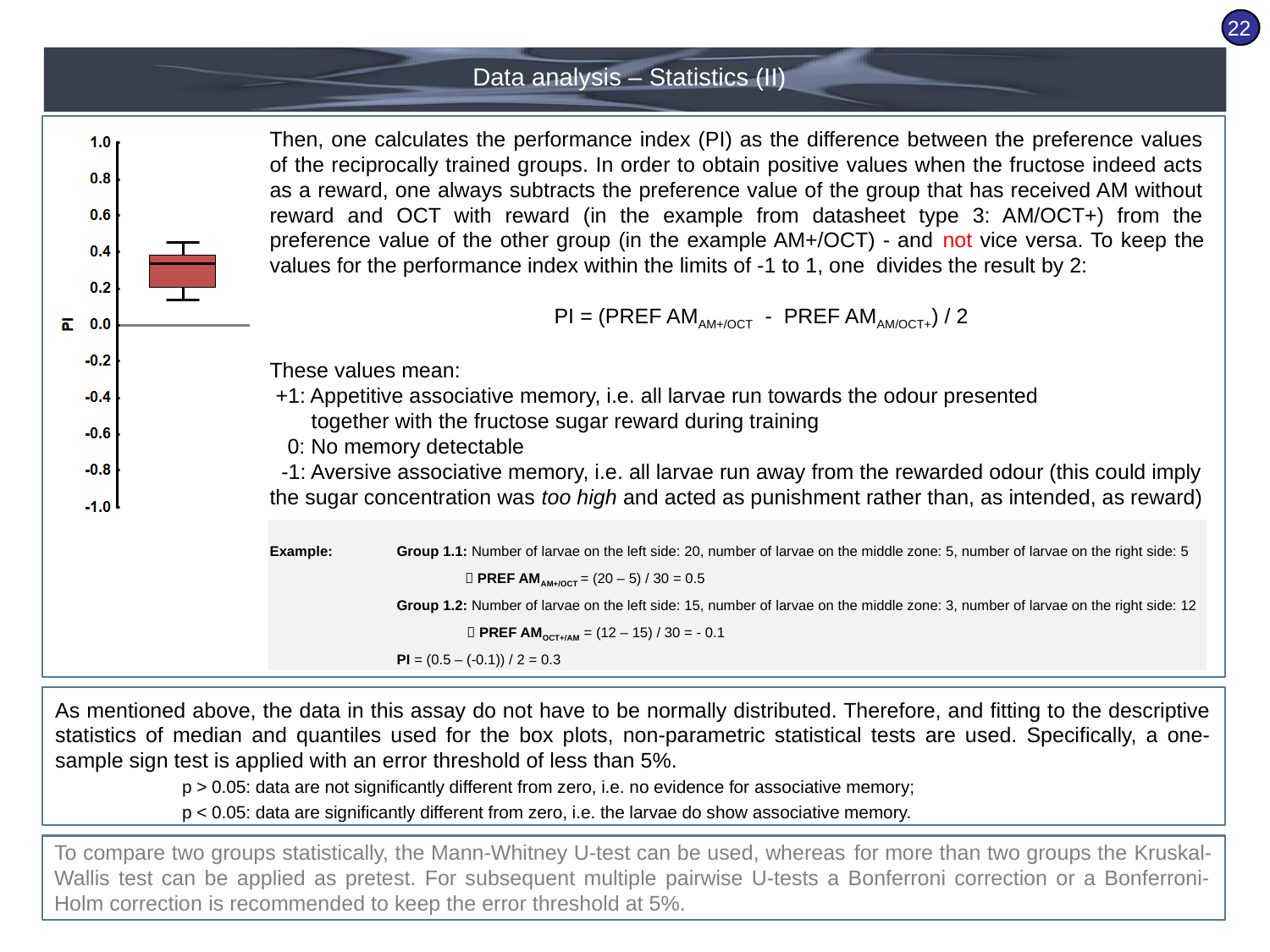

22
Data analysis – Statistics (II)
Then, one calculates the performance index (PI) as the difference between the preference values of the reciprocally trained groups. In order to obtain positive values when the fructose indeed acts as a reward, one always subtracts the preference value of the group that has received AM without reward and OCT with reward (in the example from datasheet type 3: AM/OCT+) from the preference value of the other group (in the example AM+/OCT) - and not vice versa. To keep the values for the performance index within the limits of -1 to 1, one divides the result by 2:
 PI = (PREF AMAM+/OCT - PREF AMAM/OCT+) / 2
These values mean:
 +1: Appetitive associative memory, i.e. all larvae run towards the odour presented
 together with the fructose sugar reward during training
  0: No memory detectable
 -1: Aversive associative memory, i.e. all larvae run away from the rewarded odour (this could imply the sugar concentration was too high and acted as punishment rather than, as intended, as reward)
Example: 	Group 1.1: Number of larvae on the left side: 20, number of larvae on the middle zone: 5, number of larvae on the right side: 5
	  PREF AMAM+/OCT = (20 – 5) / 30 = 0.5
	Group 1.2: Number of larvae on the left side: 15, number of larvae on the middle zone: 3, number of larvae on the right side: 12
  PREF AMOCT+/AM = (12 – 15) / 30 = - 0.1
	PI = (0.5 – (-0.1)) / 2 = 0.3
As mentioned above, the data in this assay do not have to be normally distributed. Therefore, and fitting to the descriptive statistics of median and quantiles used for the box plots, non-parametric statistical tests are used. Specifically, a one-sample sign test is applied with an error threshold of less than 5%.
	p > 0.05: data are not significantly different from zero, i.e. no evidence for associative memory;
	p < 0.05: data are significantly different from zero, i.e. the larvae do show associative memory.
To compare two groups statistically, the Mann-Whitney U-test can be used, whereas for more than two groups the Kruskal-Wallis test can be applied as pretest. For subsequent multiple pairwise U-tests a Bonferroni correction or a Bonferroni-Holm correction is recommended to keep the error threshold at 5%.

## Slide 23
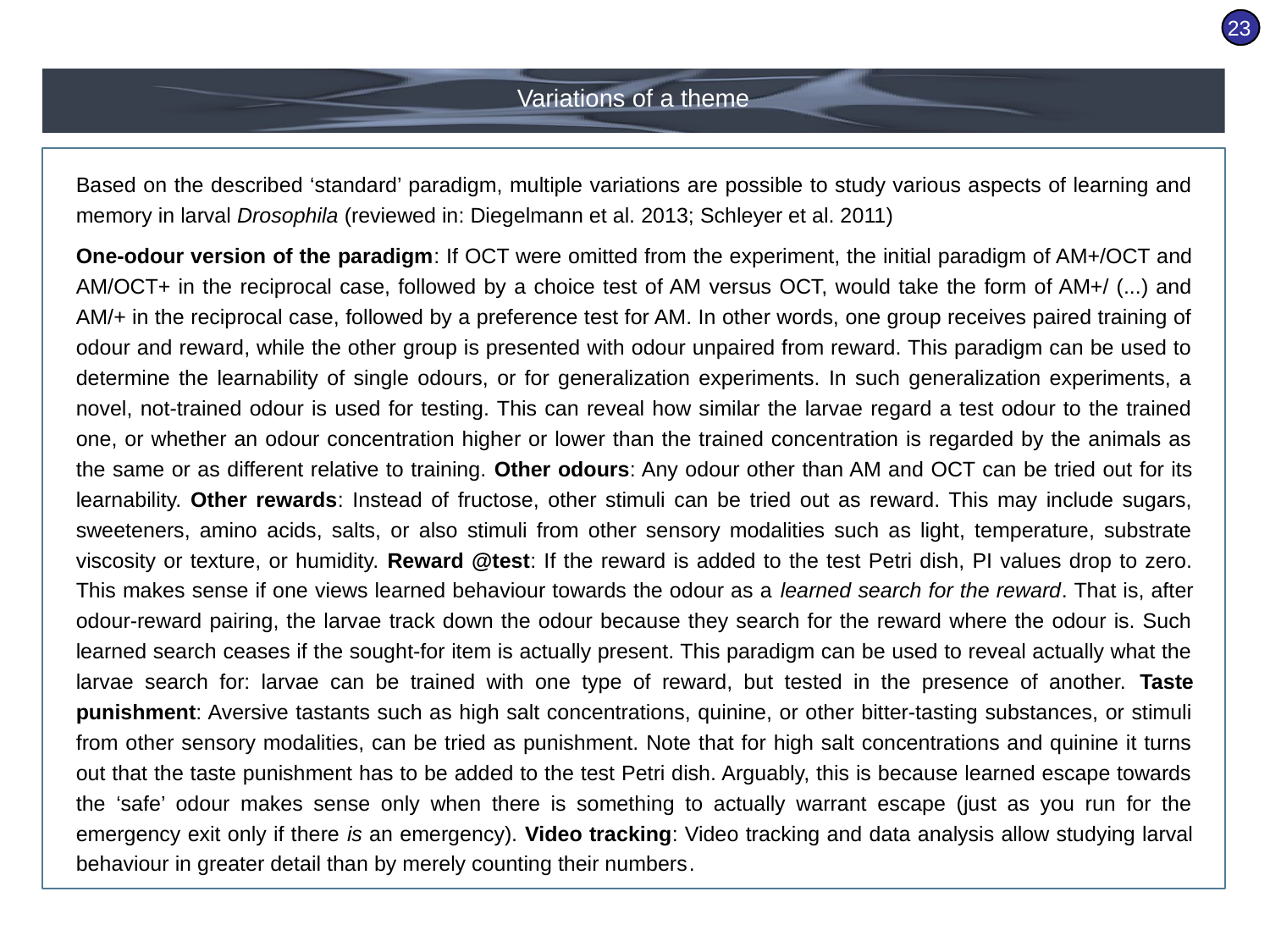

23
Variations of a theme
Based on the described ‘standard’ paradigm, multiple variations are possible to study various aspects of learning and memory in larval Drosophila (reviewed in: Diegelmann et al. 2013; Schleyer et al. 2011)
One-odour version of the paradigm: If OCT were omitted from the experiment, the initial paradigm of AM+/OCT and AM/OCT+ in the reciprocal case, followed by a choice test of AM versus OCT, would take the form of AM+/ (...) and AM/+ in the reciprocal case, followed by a preference test for AM. In other words, one group receives paired training of odour and reward, while the other group is presented with odour unpaired from reward. This paradigm can be used to determine the learnability of single odours, or for generalization experiments. In such generalization experiments, a novel, not-trained odour is used for testing. This can reveal how similar the larvae regard a test odour to the trained one, or whether an odour concentration higher or lower than the trained concentration is regarded by the animals as the same or as different relative to training. Other odours: Any odour other than AM and OCT can be tried out for its learnability. Other rewards: Instead of fructose, other stimuli can be tried out as reward. This may include sugars, sweeteners, amino acids, salts, or also stimuli from other sensory modalities such as light, temperature, substrate viscosity or texture, or humidity. Reward @test: If the reward is added to the test Petri dish, PI values drop to zero. This makes sense if one views learned behaviour towards the odour as a learned search for the reward. That is, after odour-reward pairing, the larvae track down the odour because they search for the reward where the odour is. Such learned search ceases if the sought-for item is actually present. This paradigm can be used to reveal actually what the larvae search for: larvae can be trained with one type of reward, but tested in the presence of another. Taste punishment: Aversive tastants such as high salt concentrations, quinine, or other bitter-tasting substances, or stimuli from other sensory modalities, can be tried as punishment. Note that for high salt concentrations and quinine it turns out that the taste punishment has to be added to the test Petri dish. Arguably, this is because learned escape towards the ‘safe’ odour makes sense only when there is something to actually warrant escape (just as you run for the emergency exit only if there is an emergency). Video tracking: Video tracking and data analysis allow studying larval behaviour in greater detail than by merely counting their numbers.

## Slide 24
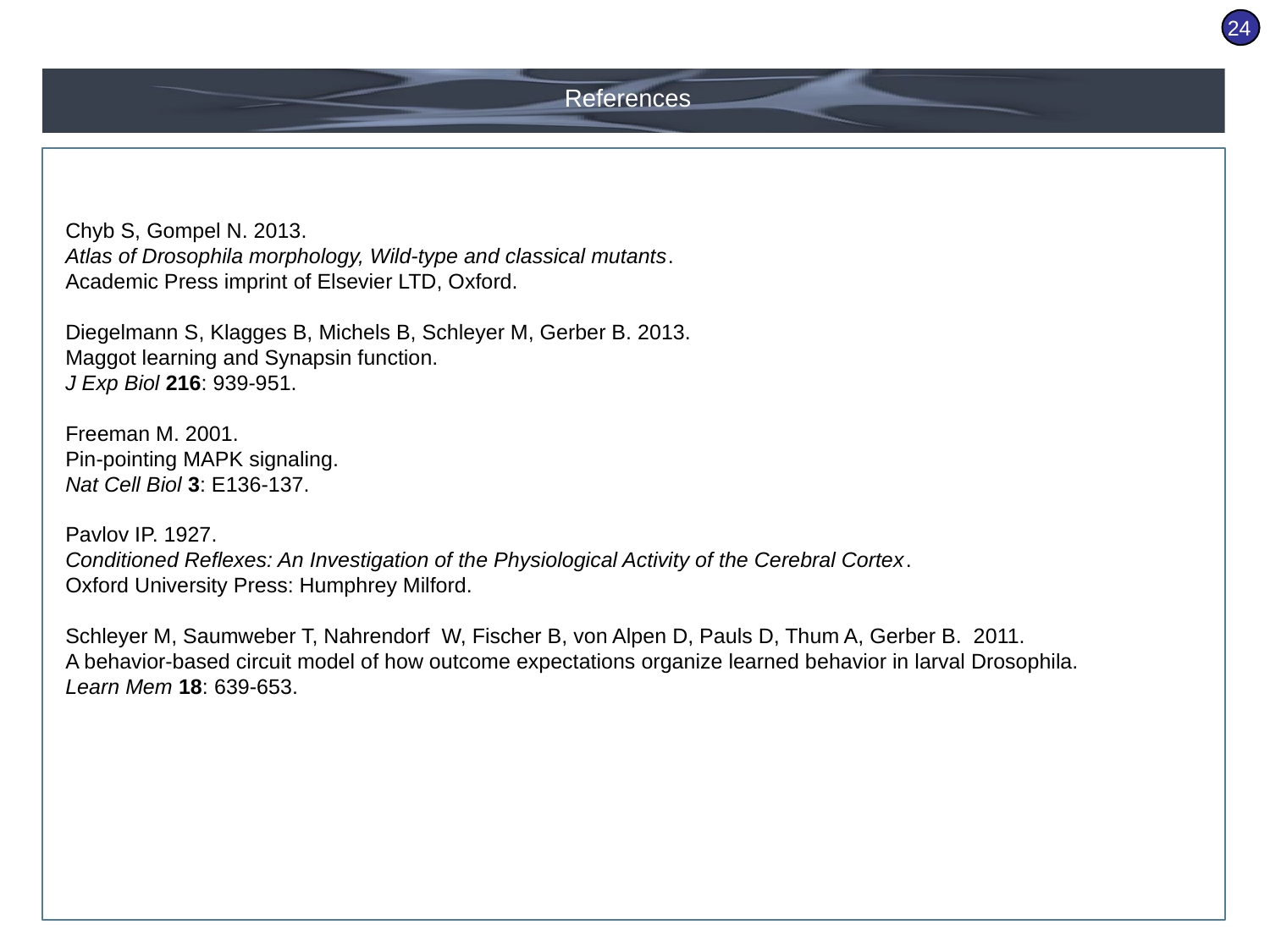

24
References
Chyb S, Gompel N. 2013.
Atlas of Drosophila morphology, Wild-type and classical mutants.
Academic Press imprint of Elsevier LTD, Oxford.
Diegelmann S, Klagges B, Michels B, Schleyer M, Gerber B. 2013.
Maggot learning and Synapsin function.
J Exp Biol 216: 939-951.
Freeman M. 2001.
Pin-pointing MAPK signaling.
Nat Cell Biol 3: E136-137.
Pavlov IP. 1927.
Conditioned Reflexes: An Investigation of the Physiological Activity of the Cerebral Cortex.
Oxford University Press: Humphrey Milford.
Schleyer M, Saumweber T, Nahrendorf W, Fischer B, von Alpen D, Pauls D, Thum A, Gerber B. 2011.
A behavior-based circuit model of how outcome expectations organize learned behavior in larval Drosophila.
Learn Mem 18: 639-653.
