# Supplementary material for: Pavlovian Conditioning of Larval Drosophila: An Illustrated, Multilingual, Hands-On Manual for Odor-Taste Associative Learning in Maggots
Source: Front Behav Neurosci. 2017 Apr 19;11:45. doi: 10.3389/fnbeh.2017.00045 (PMC5395560; doi:10.3389/fnbeh.2017.00045)
Supplement: Supplemental Materials 4–6 — A manual for odor-reward learning in larval Drosophila (Supplemental Material 4), example of a table for data analysis (Supplemental Material 5), and an empty table for entering and analyzing one's own data (Supplemental Material 6), in the German language. Versions of this manual in the English, French, Japanese, Spanish, and Italian languages can be found in Supplemental Materials 1–3, 7–9, 10, 11–13, 14–16, respectively. [file SupplementalMaterial4.pptx]

## Slide 1
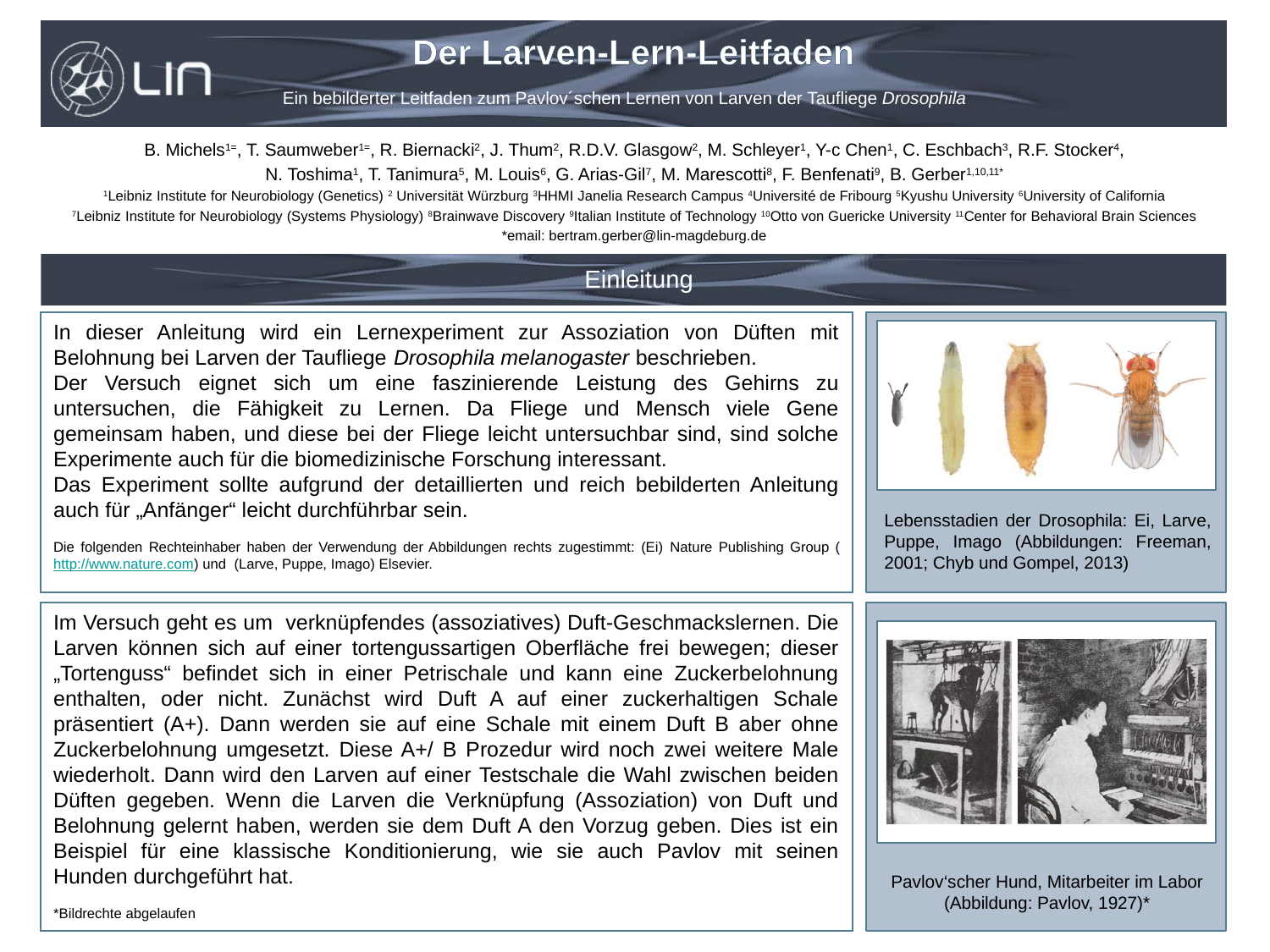

# Ein bebilderter Leitfaden zum Pavlov´schen Lernen von Larven der Taufliege Drosophila
Der Larven-Lern-Leitfaden
B. Michels1=, T. Saumweber1=, R. Biernacki2, J. Thum2, R.D.V. Glasgow2, M. Schleyer1, Y-c Chen1, C. Eschbach3, R.F. Stocker4,
N. Toshima1, T. Tanimura5, M. Louis6, G. Arias-Gil7, M. Marescotti8, F. Benfenati9, B. Gerber1,10,11*
1Leibniz Institute for Neurobiology (Genetics) 2 Universität Würzburg 3HHMI Janelia Research Campus 4Université de Fribourg 5Kyushu University 6University of California
7Leibniz Institute for Neurobiology (Systems Physiology) 8Brainwave Discovery 9Italian Institute of Technology 10Otto von Guericke University 11Center for Behavioral Brain Sciences
*email: bertram.gerber@lin-magdeburg.de
Einleitung
In dieser Anleitung wird ein Lernexperiment zur Assoziation von Düften mit Belohnung bei Larven der Taufliege Drosophila melanogaster beschrieben.
Der Versuch eignet sich um eine faszinierende Leistung des Gehirns zu untersuchen, die Fähigkeit zu Lernen. Da Fliege und Mensch viele Gene gemeinsam haben, und diese bei der Fliege leicht untersuchbar sind, sind solche Experimente auch für die biomedizinische Forschung interessant.
Das Experiment sollte aufgrund der detaillierten und reich bebilderten Anleitung auch für „Anfänger“ leicht durchführbar sein.
Die folgenden Rechteinhaber haben der Verwendung der Abbildungen rechts zugestimmt: (Ei) Nature Publishing Group (http://www.nature.com) und (Larve, Puppe, Imago) Elsevier.
Lebensstadien der Drosophila: Ei, Larve, Puppe, Imago (Abbildungen: Freeman, 2001; Chyb und Gompel, 2013)
Im Versuch geht es um verknüpfendes (assoziatives) Duft-Geschmackslernen. Die Larven können sich auf einer tortengussartigen Oberfläche frei bewegen; dieser „Tortenguss“ befindet sich in einer Petrischale und kann eine Zuckerbelohnung enthalten, oder nicht. Zunächst wird Duft A auf einer zuckerhaltigen Schale präsentiert (A+). Dann werden sie auf eine Schale mit einem Duft B aber ohne Zuckerbelohnung umgesetzt. Diese A+/ B Prozedur wird noch zwei weitere Male wiederholt. Dann wird den Larven auf einer Testschale die Wahl zwischen beiden Düften gegeben. Wenn die Larven die Verknüpfung (Assoziation) von Duft und Belohnung gelernt haben, werden sie dem Duft A den Vorzug geben. Dies ist ein Beispiel für eine klassische Konditionierung, wie sie auch Pavlov mit seinen Hunden durchgeführt hat.
*Bildrechte abgelaufen
Pavlov‘scher Hund, Mitarbeiter im Labor (Abbildung: Pavlov, 1927)*

## Slide 2
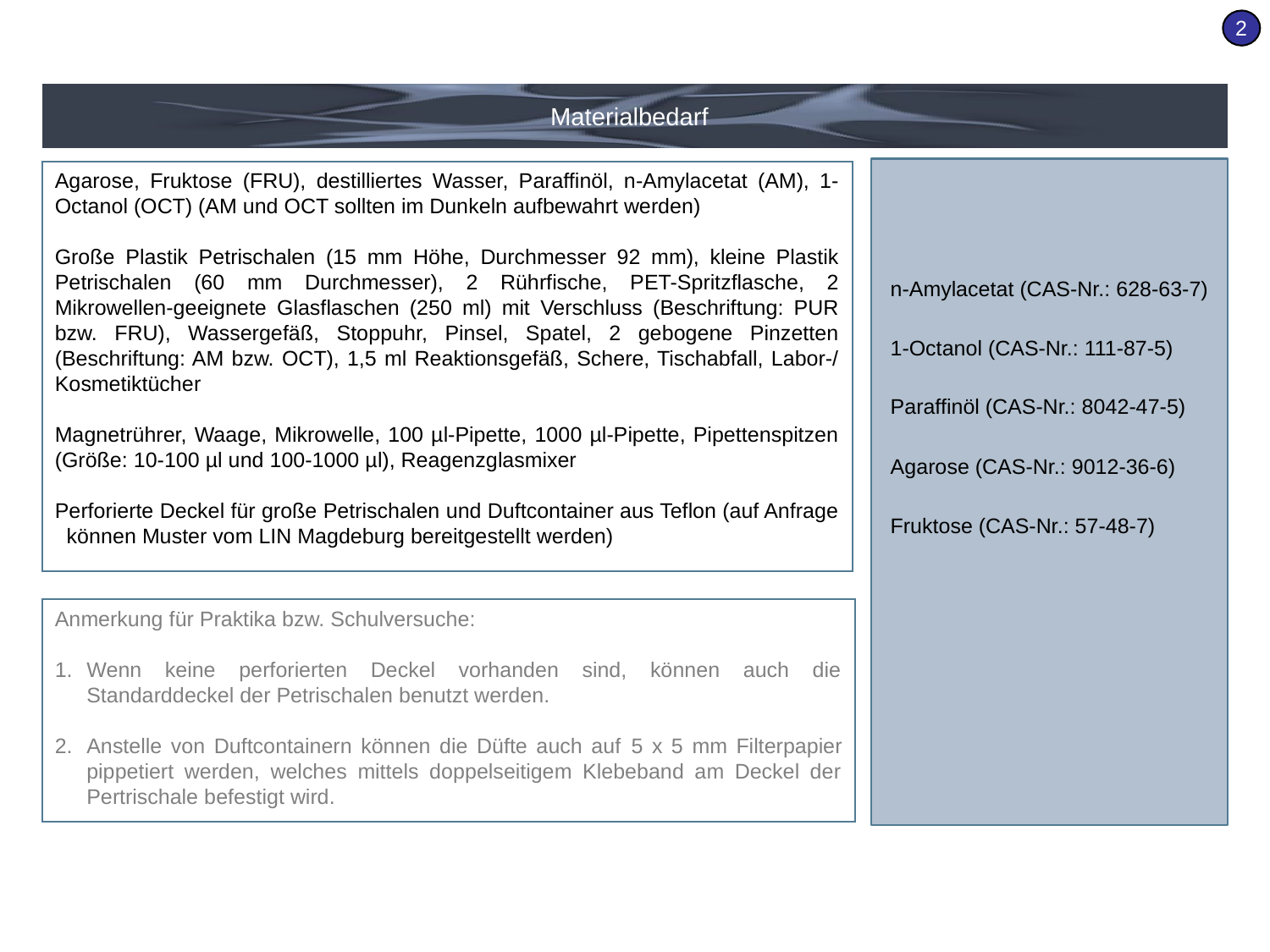

2
Materialbedarf
Agarose, Fruktose (FRU), destilliertes Wasser, Paraffinöl, n-Amylacetat (AM), 1-Octanol (OCT) (AM und OCT sollten im Dunkeln aufbewahrt werden)
Große Plastik Petrischalen (15 mm Höhe, Durchmesser 92 mm), kleine Plastik Petrischalen (60 mm Durchmesser), 2 Rührfische, PET-Spritzflasche, 2 Mikrowellen-geeignete Glasflaschen (250 ml) mit Verschluss (Beschriftung: PUR bzw. FRU), Wassergefäß, Stoppuhr, Pinsel, Spatel, 2 gebogene Pinzetten (Beschriftung: AM bzw. OCT), 1,5 ml Reaktionsgefäß, Schere, Tischabfall, Labor-/ Kosmetiktücher
Magnetrührer, Waage, Mikrowelle, 100 µl-Pipette, 1000 µl-Pipette, Pipettenspitzen (Größe: 10-100 µl und 100-1000 µl), Reagenzglasmixer
Perforierte Deckel für große Petrischalen und Duftcontainer aus Teflon (auf Anfrage können Muster vom LIN Magdeburg bereitgestellt werden)
n-Amylacetat (CAS-Nr.: 628-63-7)
1-Octanol (CAS-Nr.: 111-87-5)
Paraffinöl (CAS-Nr.: 8042-47-5)
Agarose (CAS-Nr.: 9012-36-6)
Fruktose (CAS-Nr.: 57-48-7)
Anmerkung für Praktika bzw. Schulversuche:
Wenn keine perforierten Deckel vorhanden sind, können auch die Standarddeckel der Petrischalen benutzt werden.
Anstelle von Duftcontainern können die Düfte auch auf 5 x 5 mm Filterpapier pippetiert werden, welches mittels doppelseitigem Klebeband am Deckel der Pertrischale befestigt wird.

## Slide 3
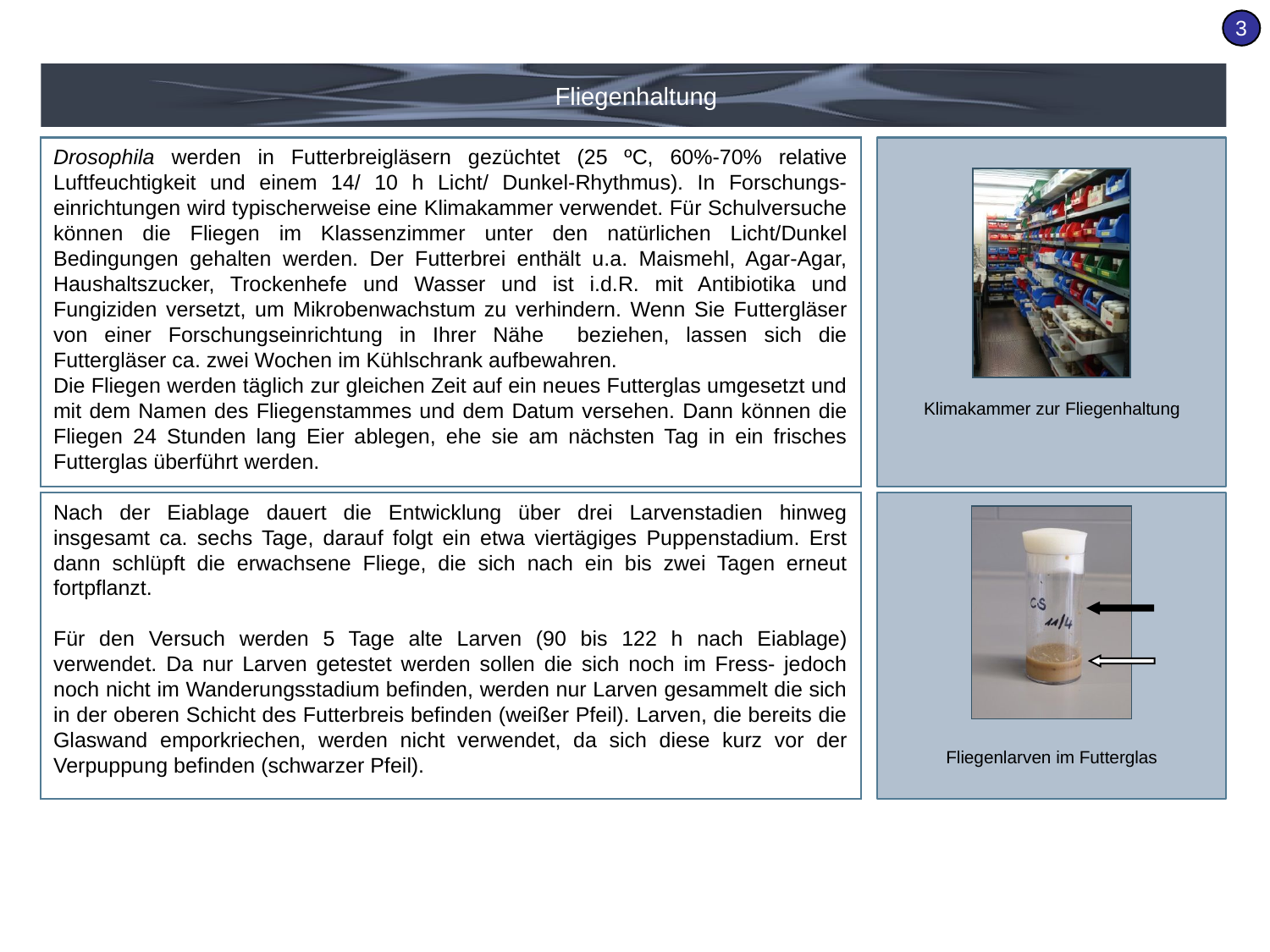

3
# Fliegenhaltung
Drosophila werden in Futterbreigläsern gezüchtet (25 ºC, 60%-70% relative Luftfeuchtigkeit und einem 14/ 10 h Licht/ Dunkel-Rhythmus). In Forschungs-einrichtungen wird typischerweise eine Klimakammer verwendet. Für Schulversuche können die Fliegen im Klassenzimmer unter den natürlichen Licht/Dunkel Bedingungen gehalten werden. Der Futterbrei enthält u.a. Maismehl, Agar-Agar, Haushaltszucker, Trockenhefe und Wasser und ist i.d.R. mit Antibiotika und Fungiziden versetzt, um Mikrobenwachstum zu verhindern. Wenn Sie Futtergläser von einer Forschungseinrichtung in Ihrer Nähe beziehen, lassen sich die Futtergläser ca. zwei Wochen im Kühlschrank aufbewahren.
Die Fliegen werden täglich zur gleichen Zeit auf ein neues Futterglas umgesetzt und mit dem Namen des Fliegenstammes und dem Datum versehen. Dann können die Fliegen 24 Stunden lang Eier ablegen, ehe sie am nächsten Tag in ein frisches Futterglas überführt werden.
Klimakammer zur Fliegenhaltung
Nach der Eiablage dauert die Entwicklung über drei Larvenstadien hinweg insgesamt ca. sechs Tage, darauf folgt ein etwa viertägiges Puppenstadium. Erst dann schlüpft die erwachsene Fliege, die sich nach ein bis zwei Tagen erneut fortpflanzt.
Für den Versuch werden 5 Tage alte Larven (90 bis 122 h nach Eiablage) verwendet. Da nur Larven getestet werden sollen die sich noch im Fress- jedoch noch nicht im Wanderungsstadium befinden, werden nur Larven gesammelt die sich in der oberen Schicht des Futterbreis befinden (weißer Pfeil). Larven, die bereits die Glaswand emporkriechen, werden nicht verwendet, da sich diese kurz vor der Verpuppung befinden (schwarzer Pfeil).
Fliegenlarven im Futterglas

## Slide 4
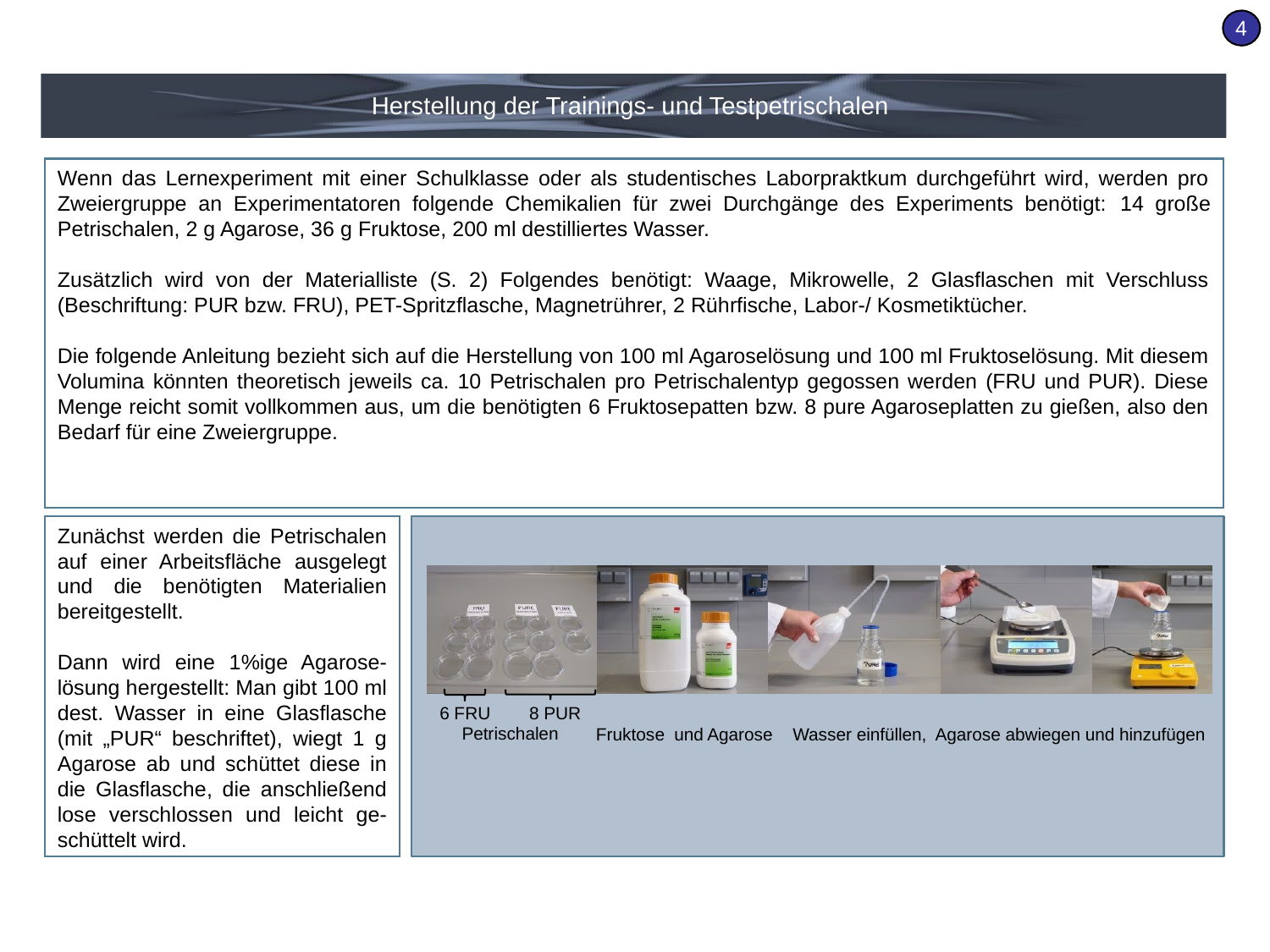

4
# Herstellung der Trainings- und Testpetrischalen
Wenn das Lernexperiment mit einer Schulklasse oder als studentisches Laborpraktkum durchgeführt wird, werden pro Zweiergruppe an Experimentatoren folgende Chemikalien für zwei Durchgänge des Experiments benötigt: 14 große Petrischalen, 2 g Agarose, 36 g Fruktose, 200 ml destilliertes Wasser.
Zusätzlich wird von der Materialliste (S. 2) Folgendes benötigt: Waage, Mikrowelle, 2 Glasflaschen mit Verschluss (Beschriftung: PUR bzw. FRU), PET-Spritzflasche, Magnetrührer, 2 Rührfische, Labor-/ Kosmetiktücher.
Die folgende Anleitung bezieht sich auf die Herstellung von 100 ml Agaroselösung und 100 ml Fruktoselösung. Mit diesem Volumina könnten theoretisch jeweils ca. 10 Petrischalen pro Petrischalentyp gegossen werden (FRU und PUR). Diese Menge reicht somit vollkommen aus, um die benötigten 6 Fruktosepatten bzw. 8 pure Agaroseplatten zu gießen, also den Bedarf für eine Zweiergruppe.
Zunächst werden die Petrischalen auf einer Arbeitsfläche ausgelegt und die benötigten Materialien bereitgestellt.
Dann wird eine 1%ige Agarose-lösung hergestellt: Man gibt 100 ml dest. Wasser in eine Glasflasche (mit „PUR“ beschriftet), wiegt 1 g Agarose ab und schüttet diese in die Glasflasche, die anschließend lose verschlossen und leicht ge-schüttelt wird.
6 FRU 8 PUR
Petrischalen
Fruktose und Agarose
Wasser einfüllen, Agarose abwiegen und hinzufügen

## Slide 5
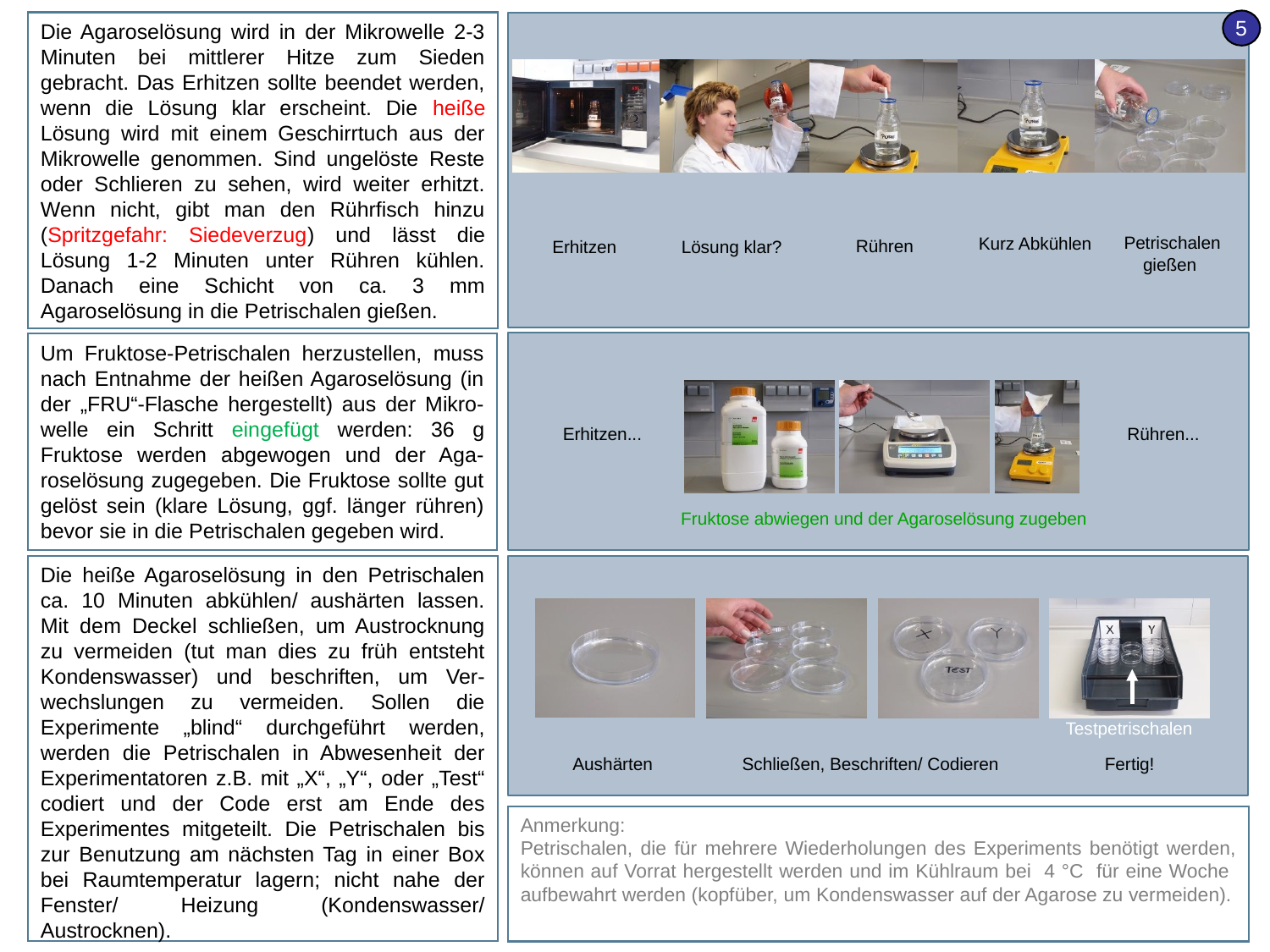

5
Die Agaroselösung wird in der Mikrowelle 2-3 Minuten bei mittlerer Hitze zum Sieden gebracht. Das Erhitzen sollte beendet werden, wenn die Lösung klar erscheint. Die heiße Lösung wird mit einem Geschirrtuch aus der Mikrowelle genommen. Sind ungelöste Reste oder Schlieren zu sehen, wird weiter erhitzt. Wenn nicht, gibt man den Rührfisch hinzu (Spritzgefahr: Siedeverzug) und lässt die Lösung 1-2 Minuten unter Rühren kühlen. Danach eine Schicht von ca. 3 mm Agaroselösung in die Petrischalen gießen.
Petrischalen
gießen
Lösung klar?
Kurz Abkühlen
Rühren
Erhitzen
Um Fruktose-Petrischalen herzustellen, muss nach Entnahme der heißen Agaroselösung (in der „FRU“-Flasche hergestellt) aus der Mikro-welle ein Schritt eingefügt werden: 36 g Fruktose werden abgewogen und der Aga-roselösung zugegeben. Die Fruktose sollte gut gelöst sein (klare Lösung, ggf. länger rühren) bevor sie in die Petrischalen gegeben wird.
Erhitzen...
Rühren...
Fruktose abwiegen und der Agaroselösung zugeben
Die heiße Agaroselösung in den Petrischalen ca. 10 Minuten abkühlen/ aushärten lassen. Mit dem Deckel schließen, um Austrocknung zu vermeiden (tut man dies zu früh entsteht Kondenswasser) und beschriften, um Ver-wechslungen zu vermeiden. Sollen die Experimente „blind“ durchgeführt werden, werden die Petrischalen in Abwesenheit der Experimentatoren z.B. mit „X“, „Y“, oder „Test“ codiert und der Code erst am Ende des Experimentes mitgeteilt. Die Petrischalen bis zur Benutzung am nächsten Tag in einer Box bei Raumtemperatur lagern; nicht nahe der Fenster/ Heizung (Kondenswasser/ Austrocknen).
Testpetrischalen
Aushärten
Schließen, Beschriften/ Codieren
Fertig!
Anmerkung:
Petrischalen, die für mehrere Wiederholungen des Experiments benötigt werden, können auf Vorrat hergestellt werden und im Kühlraum bei 4 °C für eine Woche aufbewahrt werden (kopfüber, um Kondenswasser auf der Agarose zu vermeiden).

## Slide 6
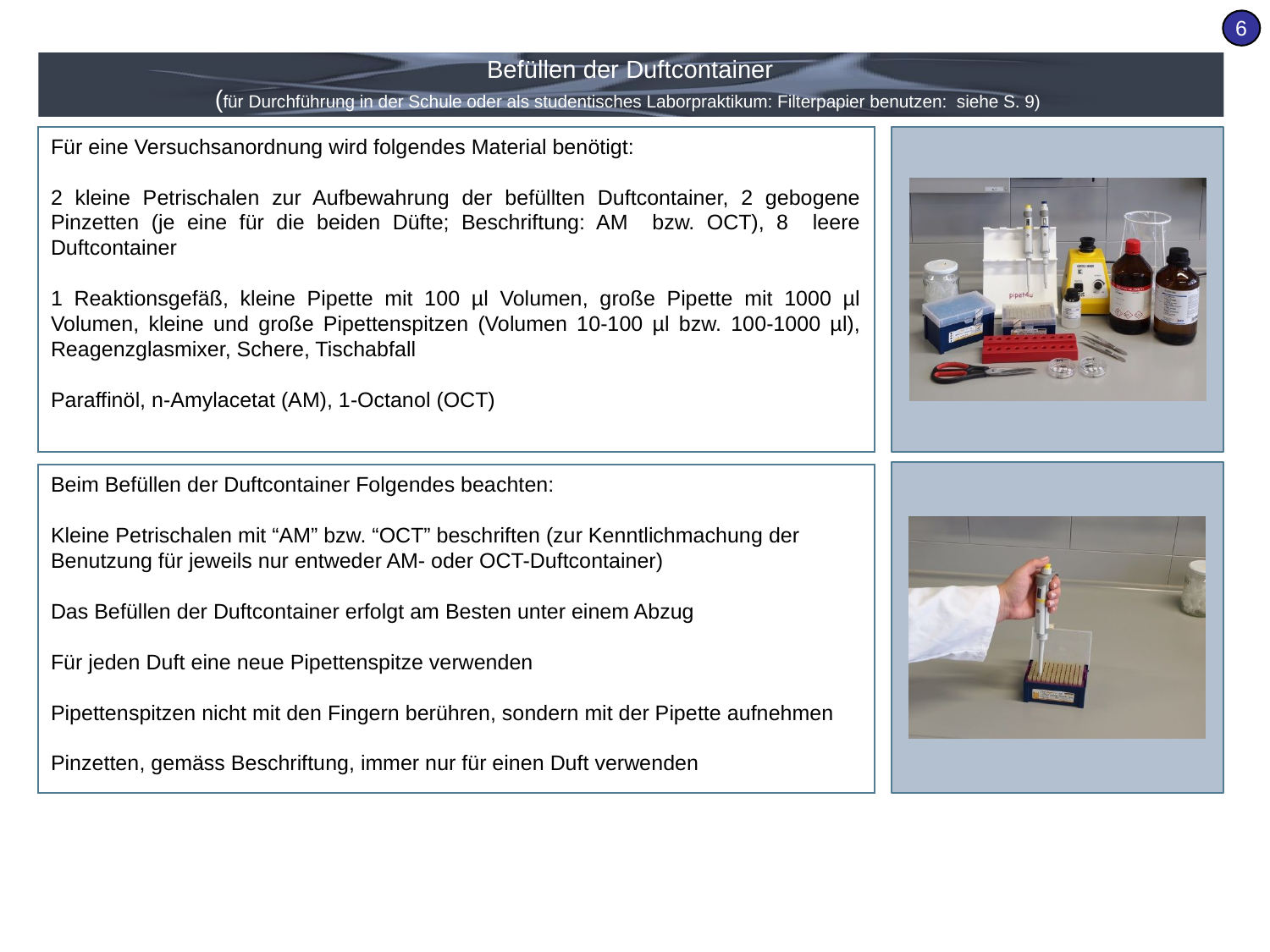

6
# Befüllen der Duftcontainer (für Durchführung in der Schule oder als studentisches Laborpraktikum: Filterpapier benutzen: siehe S. 9)
Für eine Versuchsanordnung wird folgendes Material benötigt:
2 kleine Petrischalen zur Aufbewahrung der befüllten Duftcontainer, 2 gebogene Pinzetten (je eine für die beiden Düfte; Beschriftung: AM bzw. OCT), 8 leere Duftcontainer
1 Reaktionsgefäß, kleine Pipette mit 100 µl Volumen, große Pipette mit 1000 µl Volumen, kleine und große Pipettenspitzen (Volumen 10-100 µl bzw. 100-1000 µl), Reagenzglasmixer, Schere, Tischabfall
Paraffinöl, n-Amylacetat (AM), 1-Octanol (OCT)
Beim Befüllen der Duftcontainer Folgendes beachten:
Kleine Petrischalen mit “AM” bzw. “OCT” beschriften (zur Kenntlichmachung der Benutzung für jeweils nur entweder AM- oder OCT-Duftcontainer)
Das Befüllen der Duftcontainer erfolgt am Besten unter einem Abzug
Für jeden Duft eine neue Pipettenspitze verwenden
Pipettenspitzen nicht mit den Fingern berühren, sondern mit der Pipette aufnehmen
Pinzetten, gemäss Beschriftung, immer nur für einen Duft verwenden

## Slide 7
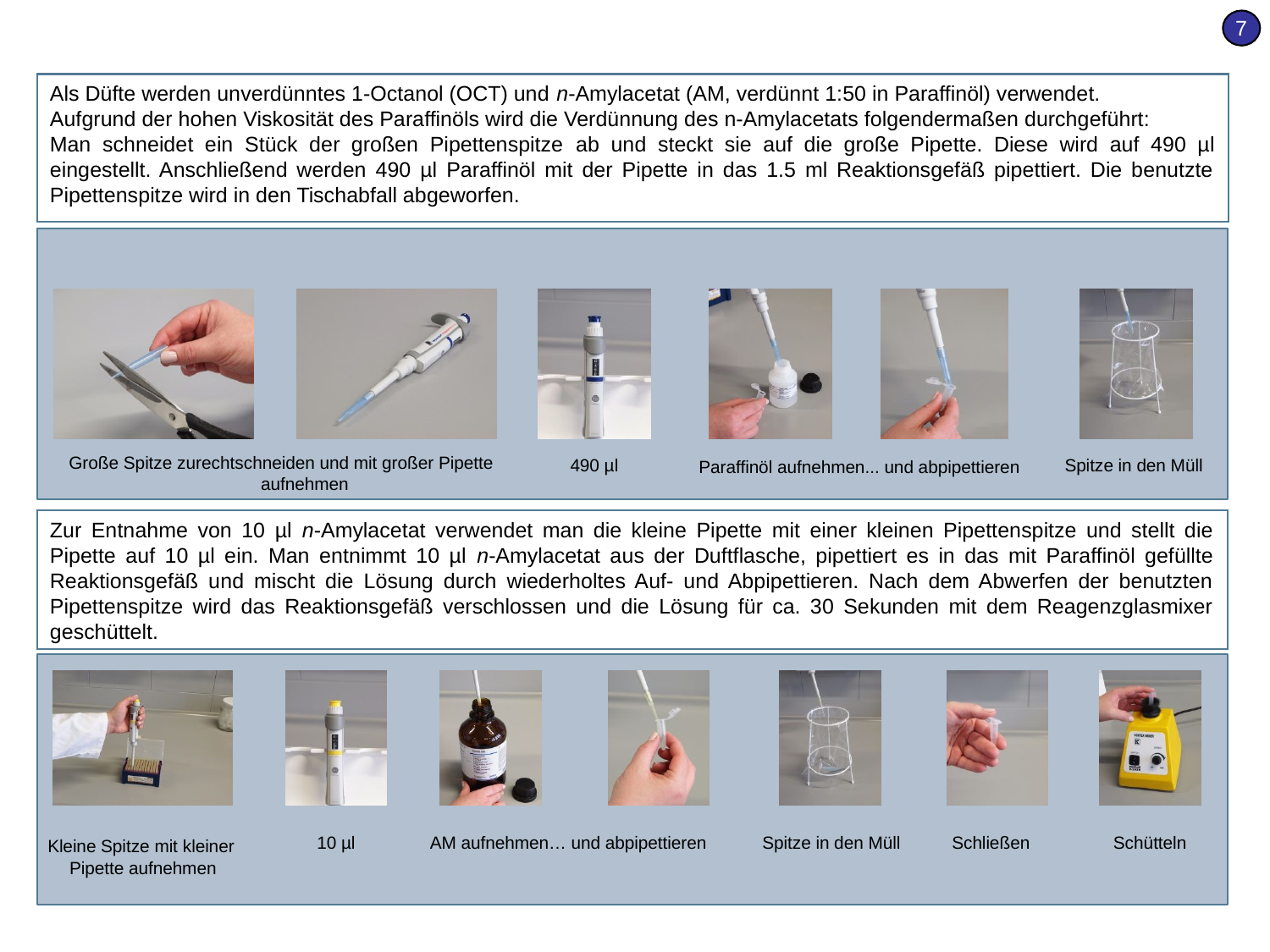

7
Als Düfte werden unverdünntes 1-Octanol (OCT) und n-Amylacetat (AM, verdünnt 1:50 in Paraffinöl) verwendet.
Aufgrund der hohen Viskosität des Paraffinöls wird die Verdünnung des n-Amylacetats folgendermaßen durchgeführt:
Man schneidet ein Stück der großen Pipettenspitze ab und steckt sie auf die große Pipette. Diese wird auf 490 µl eingestellt. Anschließend werden 490 µl Paraffinöl mit der Pipette in das 1.5 ml Reaktionsgefäß pipettiert. Die benutzte Pipettenspitze wird in den Tischabfall abgeworfen.
Große Spitze zurechtschneiden und mit großer Pipette aufnehmen
490 µl
Spitze in den Müll
Paraffinöl aufnehmen... und abpipettieren
Zur Entnahme von 10 µl n-Amylacetat verwendet man die kleine Pipette mit einer kleinen Pipettenspitze und stellt die Pipette auf 10 µl ein. Man entnimmt 10 µl n-Amylacetat aus der Duftflasche, pipettiert es in das mit Paraffinöl gefüllte Reaktionsgefäß und mischt die Lösung durch wiederholtes Auf- und Abpipettieren. Nach dem Abwerfen der benutzten Pipettenspitze wird das Reaktionsgefäß verschlossen und die Lösung für ca. 30 Sekunden mit dem Reagenzglasmixer geschüttelt.
 Kleine Spitze mit kleiner
Pipette aufnehmen
10 µl
AM aufnehmen… und abpipettieren
Spitze in den Müll
Schließen
Schütteln

## Slide 8
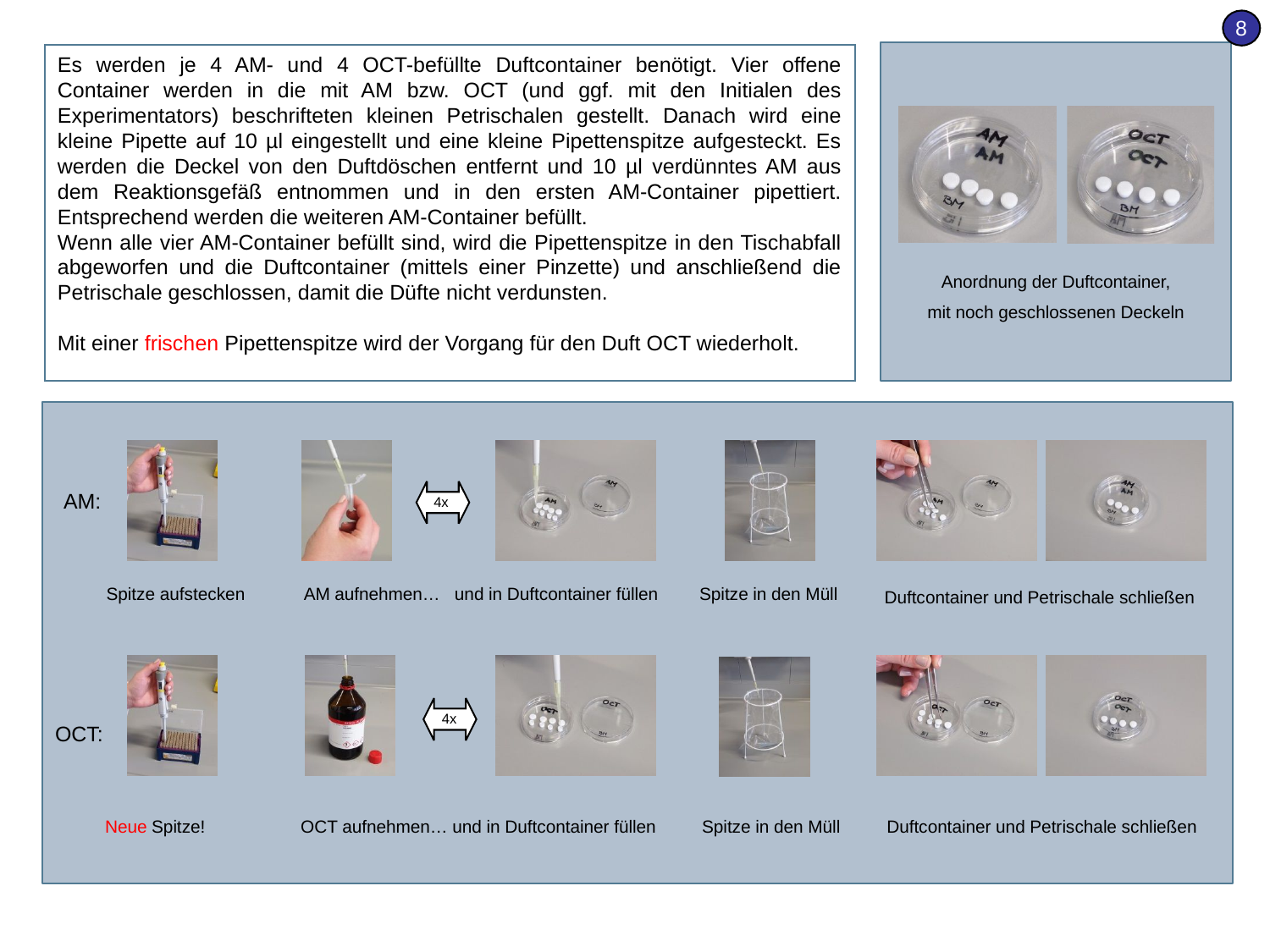

8
Es werden je 4 AM- und 4 OCT-befüllte Duftcontainer benötigt. Vier offene Container werden in die mit AM bzw. OCT (und ggf. mit den Initialen des Experimentators) beschrifteten kleinen Petrischalen gestellt. Danach wird eine kleine Pipette auf 10 µl eingestellt und eine kleine Pipettenspitze aufgesteckt. Es werden die Deckel von den Duftdöschen entfernt und 10 µl verdünntes AM aus dem Reaktionsgefäß entnommen und in den ersten AM-Container pipettiert. Entsprechend werden die weiteren AM-Container befüllt.
Wenn alle vier AM-Container befüllt sind, wird die Pipettenspitze in den Tischabfall abgeworfen und die Duftcontainer (mittels einer Pinzette) und anschließend die Petrischale geschlossen, damit die Düfte nicht verdunsten.
Mit einer frischen Pipettenspitze wird der Vorgang für den Duft OCT wiederholt.
Anordnung der Duftcontainer,
mit noch geschlossenen Deckeln
4x
AM:
Spitze aufstecken
AM aufnehmen… und in Duftcontainer füllen
Spitze in den Müll
Duftcontainer und Petrischale schließen
4x
OCT:
Neue Spitze!
 OCT aufnehmen… und in Duftcontainer füllen
 Spitze in den Müll
 Duftcontainer und Petrischale schließen

## Slide 9
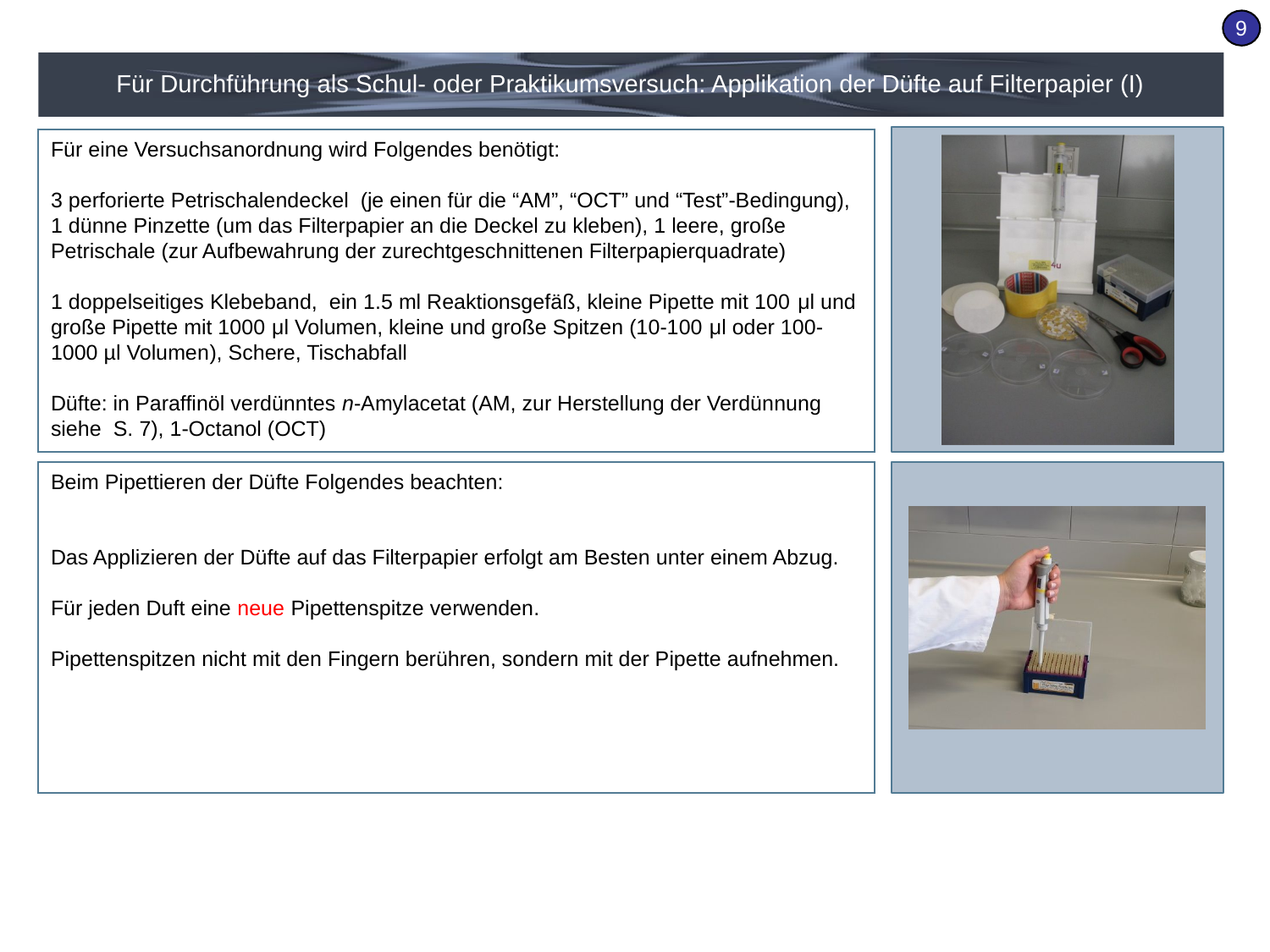

9
# Für Durchführung als Schul- oder Praktikumsversuch: Applikation der Düfte auf Filterpapier (I)
Für eine Versuchsanordnung wird Folgendes benötigt:
3 perforierte Petrischalendeckel (je einen für die “AM”, “OCT” und “Test”-Bedingung), 1 dünne Pinzette (um das Filterpapier an die Deckel zu kleben), 1 leere, große Petrischale (zur Aufbewahrung der zurechtgeschnittenen Filterpapierquadrate)
1 doppelseitiges Klebeband, ein 1.5 ml Reaktionsgefäß, kleine Pipette mit 100 μl und große Pipette mit 1000 μl Volumen, kleine und große Spitzen (10-100 μl oder 100-1000 µl Volumen), Schere, Tischabfall
 
Düfte: in Paraffinöl verdünntes n-Amylacetat (AM, zur Herstellung der Verdünnung siehe S. 7), 1-Octanol (OCT)
Beim Pipettieren der Düfte Folgendes beachten:
Das Applizieren der Düfte auf das Filterpapier erfolgt am Besten unter einem Abzug.
Für jeden Duft eine neue Pipettenspitze verwenden.
Pipettenspitzen nicht mit den Fingern berühren, sondern mit der Pipette aufnehmen.

## Slide 10
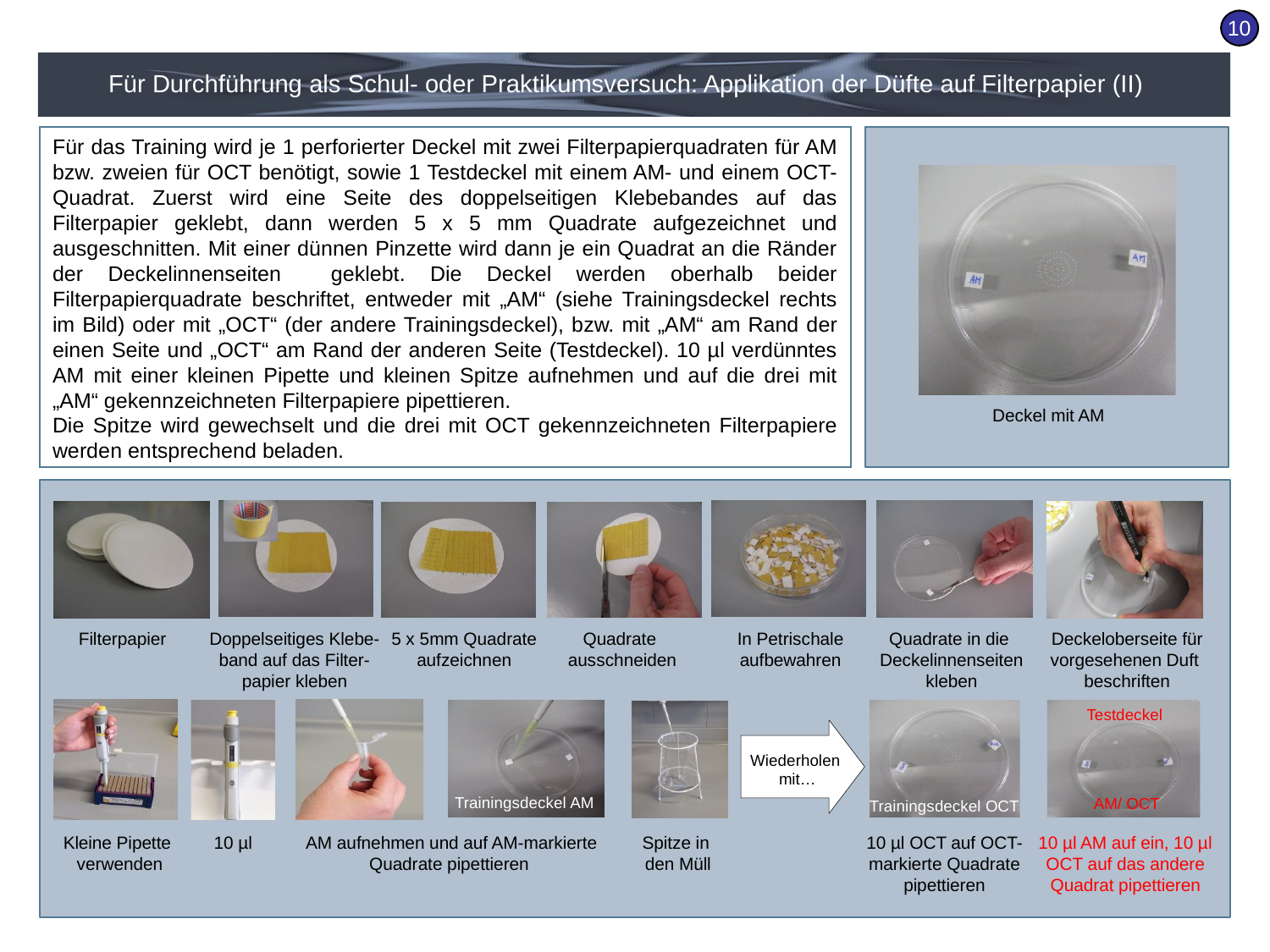

10
# Für Durchführung als Schul- oder Praktikumsversuch: Applikation der Düfte auf Filterpapier (II)
Für das Training wird je 1 perforierter Deckel mit zwei Filterpapierquadraten für AM bzw. zweien für OCT benötigt, sowie 1 Testdeckel mit einem AM- und einem OCT-Quadrat. Zuerst wird eine Seite des doppelseitigen Klebebandes auf das Filterpapier geklebt, dann werden 5 x 5 mm Quadrate aufgezeichnet und ausgeschnitten. Mit einer dünnen Pinzette wird dann je ein Quadrat an die Ränder der Deckelinnenseiten geklebt. Die Deckel werden oberhalb beider Filterpapierquadrate beschriftet, entweder mit „AM“ (siehe Trainingsdeckel rechts im Bild) oder mit „OCT“ (der andere Trainingsdeckel), bzw. mit „AM“ am Rand der einen Seite und „OCT“ am Rand der anderen Seite (Testdeckel). 10 µl verdünntes AM mit einer kleinen Pipette und kleinen Spitze aufnehmen und auf die drei mit „AM“ gekennzeichneten Filterpapiere pipettieren.
Die Spitze wird gewechselt und die drei mit OCT gekennzeichneten Filterpapiere werden entsprechend beladen.
Deckel mit AM
Filterpapier
Doppelseitiges Klebe- band auf das Filter-papier kleben
5 x 5mm Quadrate
aufzeichnen
Quadrate
ausschneiden
In Petrischale
aufbewahren
Quadrate in die
Deckelinnenseiten kleben
Deckeloberseite für vorgesehenen Duft beschriften
Testdeckel
AM/ OCT
Wiederholen
mit…
Trainingsdeckel AM
Trainingsdeckel OCT
Kleine Pipette
verwenden
10 µl
 AM aufnehmen und auf AM-markierte
Quadrate pipettieren
Spitze in
den Müll
10 µl OCT auf OCT-markierte Quadrate pipettieren
10 µl AM auf ein, 10 µl OCT auf das andere Quadrat pipettieren

## Slide 11
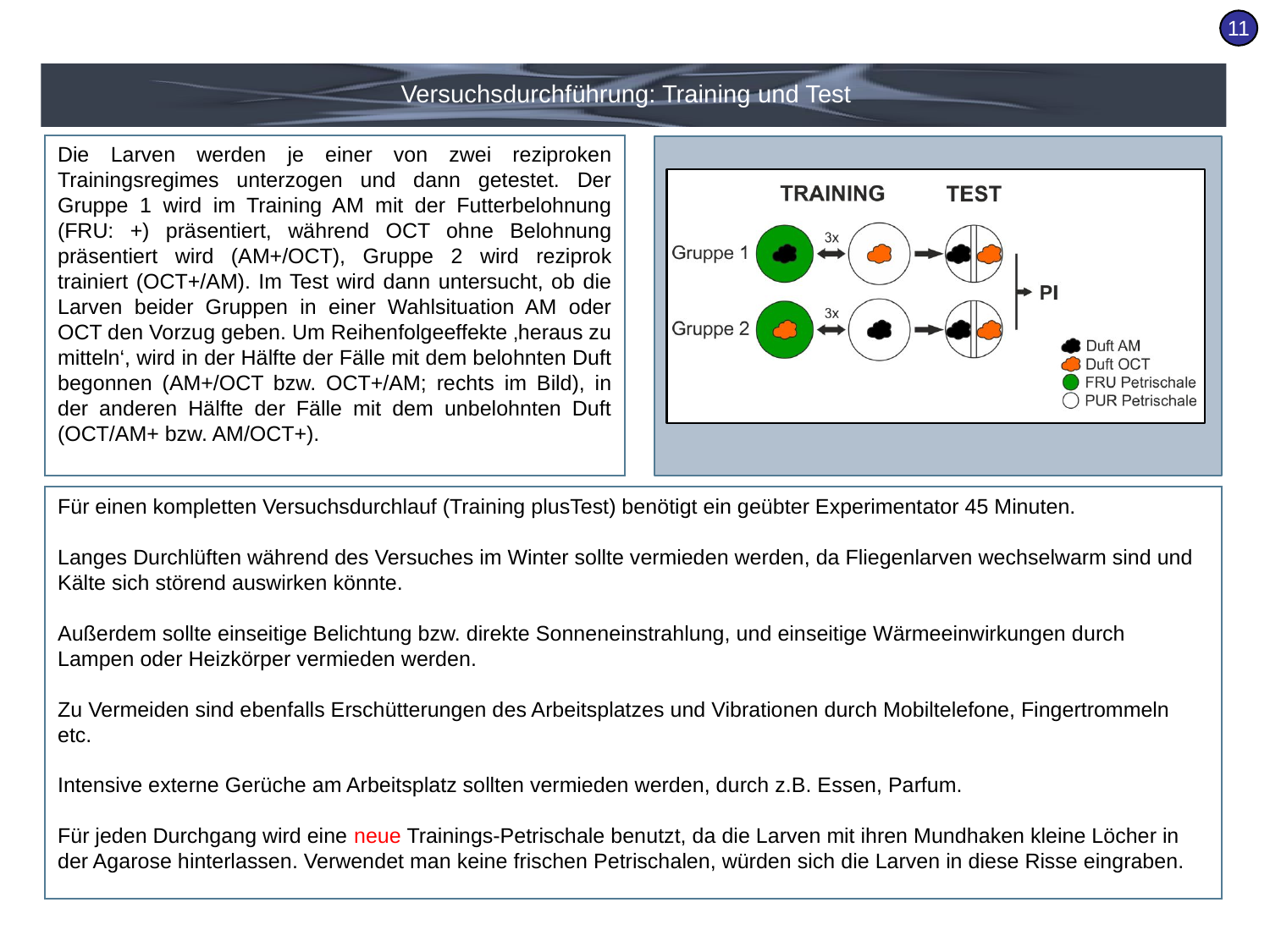

11
Versuchsdurchführung: Training und Test
Die Larven werden je einer von zwei reziproken Trainingsregimes unterzogen und dann getestet. Der Gruppe 1 wird im Training AM mit der Futterbelohnung (FRU: +) präsentiert, während OCT ohne Belohnung präsentiert wird (AM+/OCT), Gruppe 2 wird reziprok trainiert (OCT+/AM). Im Test wird dann untersucht, ob die Larven beider Gruppen in einer Wahlsituation AM oder OCT den Vorzug geben. Um Reihenfolgeeffekte ‚heraus zu mitteln‘, wird in der Hälfte der Fälle mit dem belohnten Duft begonnen (AM+/OCT bzw. OCT+/AM; rechts im Bild), in der anderen Hälfte der Fälle mit dem unbelohnten Duft (OCT/AM+ bzw. AM/OCT+).
Für einen kompletten Versuchsdurchlauf (Training plusTest) benötigt ein geübter Experimentator 45 Minuten.
Langes Durchlüften während des Versuches im Winter sollte vermieden werden, da Fliegenlarven wechselwarm sind und Kälte sich störend auswirken könnte.
Außerdem sollte einseitige Belichtung bzw. direkte Sonneneinstrahlung, und einseitige Wärmeeinwirkungen durch Lampen oder Heizkörper vermieden werden.
Zu Vermeiden sind ebenfalls Erschütterungen des Arbeitsplatzes und Vibrationen durch Mobiltelefone, Fingertrommeln etc.
Intensive externe Gerüche am Arbeitsplatz sollten vermieden werden, durch z.B. Essen, Parfum.
Für jeden Durchgang wird eine neue Trainings-Petrischale benutzt, da die Larven mit ihren Mundhaken kleine Löcher in der Agarose hinterlassen. Verwendet man keine frischen Petrischalen, würden sich die Larven in diese Risse eingraben.

## Slide 12
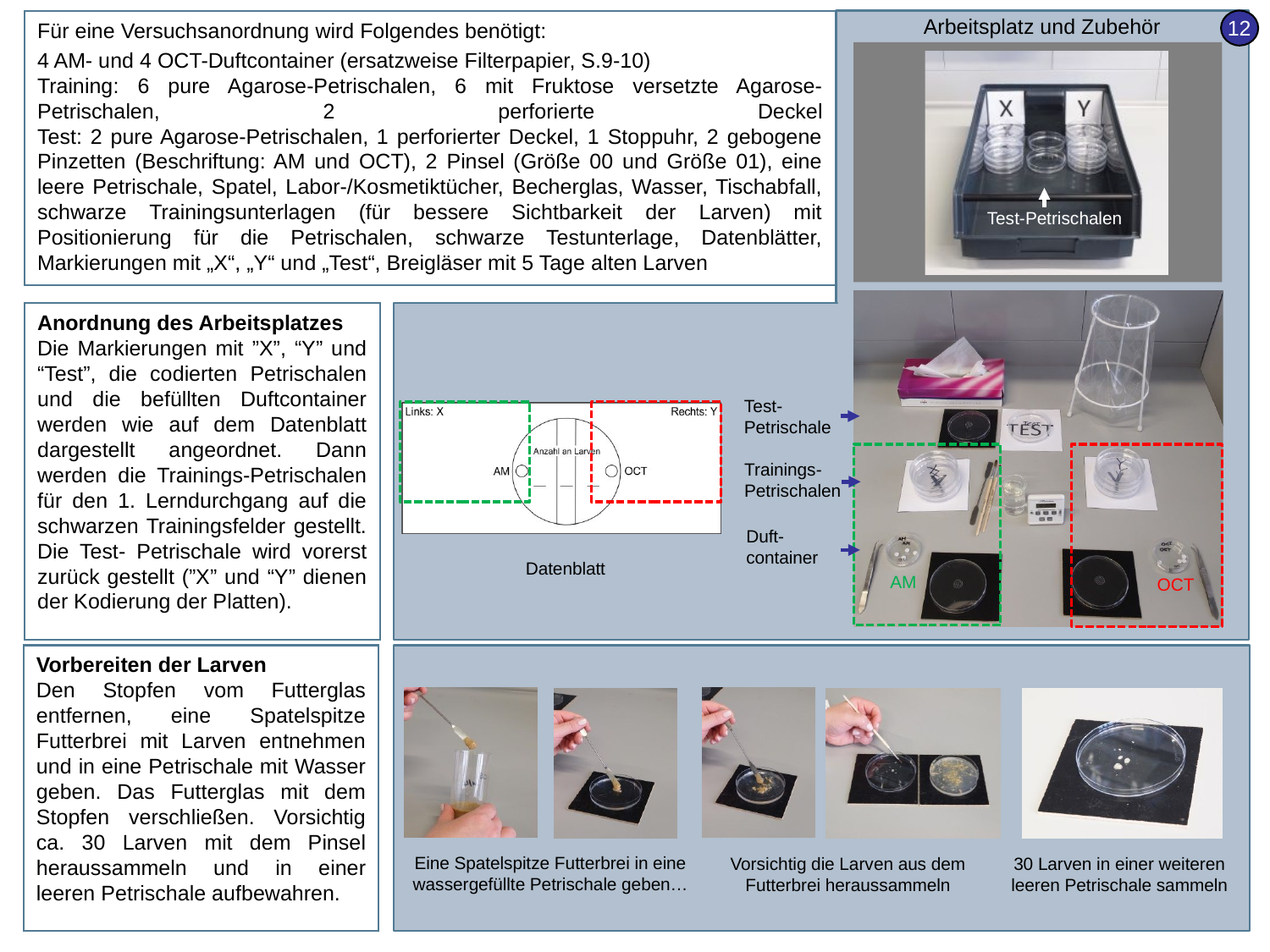

Arbeitsplatz und Zubehör
12
Für eine Versuchsanordnung wird Folgendes benötigt:
4 AM- und 4 OCT-Duftcontainer (ersatzweise Filterpapier, S.9-10)
Training: 6 pure Agarose-Petrischalen, 6 mit Fruktose versetzte Agarose-Petrischalen, 2 perforierte Deckel
Test: 2 pure Agarose-Petrischalen, 1 perforierter Deckel, 1 Stoppuhr, 2 gebogene Pinzetten (Beschriftung: AM und OCT), 2 Pinsel (Größe 00 und Größe 01), eine leere Petrischale, Spatel, Labor-/Kosmetiktücher, Becherglas, Wasser, Tischabfall, schwarze Trainingsunterlagen (für bessere Sichtbarkeit der Larven) mit Positionierung für die Petrischalen, schwarze Testunterlage, Datenblätter, Markierungen mit „X“, „Y“ und „Test“, Breigläser mit 5 Tage alten Larven
Test-Petrischalen
Anordnung des Arbeitsplatzes
Die Markierungen mit ”X”, “Y” und “Test”, die codierten Petrischalen und die befüllten Duftcontainer werden wie auf dem Datenblatt dargestellt angeordnet. Dann werden die Trainings-Petrischalen für den 1. Lerndurchgang auf die schwarzen Trainingsfelder gestellt. Die Test- Petrischale wird vorerst zurück gestellt (”X” und “Y” dienen der Kodierung der Platten).
Test-
Petrischale
Trainings-
Petrischalen
Duft-
container
Datenblatt
AM
OCT
Vorbereiten der Larven
Den Stopfen vom Futterglas entfernen, eine Spatelspitze Futterbrei mit Larven entnehmen und in eine Petrischale mit Wasser geben. Das Futterglas mit dem Stopfen verschließen. Vorsichtig ca. 30 Larven mit dem Pinsel heraussammeln und in einer leeren Petrischale aufbewahren.
Eine Spatelspitze Futterbrei in eine wassergefüllte Petrischale geben…
Vorsichtig die Larven aus dem Futterbrei heraussammeln
30 Larven in einer weiteren
leeren Petrischale sammeln

## Slide 13
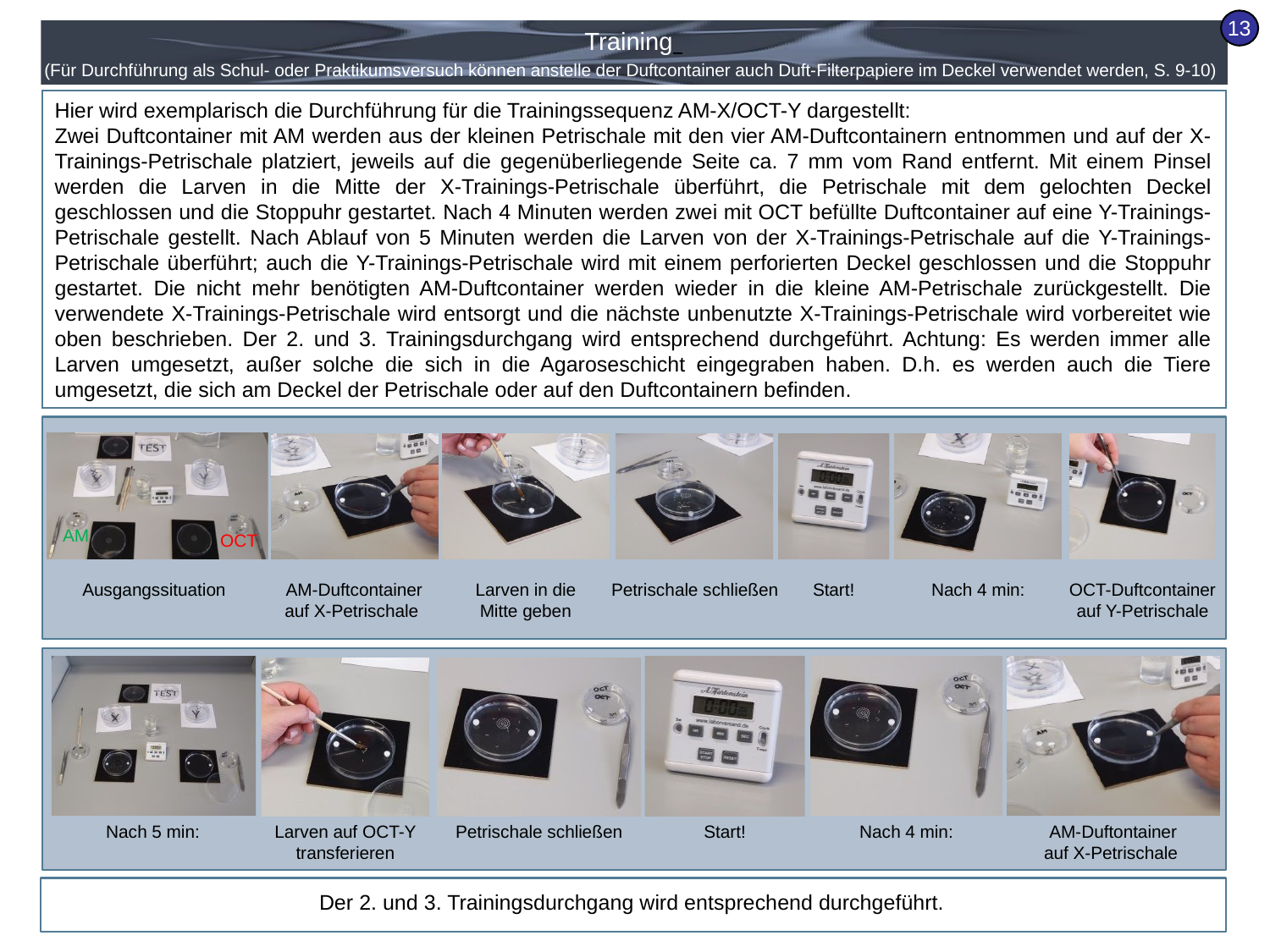

13
# Training
(Für Durchführung als Schul- oder Praktikumsversuch können anstelle der Duftcontainer auch Duft-Filterpapiere im Deckel verwendet werden, S. 9-10)
Hier wird exemplarisch die Durchführung für die Trainingssequenz AM-X/OCT-Y dargestellt:
Zwei Duftcontainer mit AM werden aus der kleinen Petrischale mit den vier AM-Duftcontainern entnommen und auf der X-Trainings-Petrischale platziert, jeweils auf die gegenüberliegende Seite ca. 7 mm vom Rand entfernt. Mit einem Pinsel werden die Larven in die Mitte der X-Trainings-Petrischale überführt, die Petrischale mit dem gelochten Deckel geschlossen und die Stoppuhr gestartet. Nach 4 Minuten werden zwei mit OCT befüllte Duftcontainer auf eine Y-Trainings-Petrischale gestellt. Nach Ablauf von 5 Minuten werden die Larven von der X-Trainings-Petrischale auf die Y-Trainings-Petrischale überführt; auch die Y-Trainings-Petrischale wird mit einem perforierten Deckel geschlossen und die Stoppuhr gestartet. Die nicht mehr benötigten AM-Duftcontainer werden wieder in die kleine AM-Petrischale zurückgestellt. Die verwendete X-Trainings-Petrischale wird entsorgt und die nächste unbenutzte X-Trainings-Petrischale wird vorbereitet wie oben beschrieben. Der 2. und 3. Trainingsdurchgang wird entsprechend durchgeführt. Achtung: Es werden immer alle Larven umgesetzt, außer solche die sich in die Agaroseschicht eingegraben haben. D.h. es werden auch die Tiere umgesetzt, die sich am Deckel der Petrischale oder auf den Duftcontainern befinden.
AM
OCT
Ausgangssituation
AM-Duftcontainer
auf X-Petrischale
Larven in die
Mitte geben
Petrischale schließen
Start!
Nach 4 min:
OCT-Duftcontainer
auf Y-Petrischale
Nach 5 min:
Larven auf OCT-Y transferieren
Petrischale schließen
Start!
Nach 4 min:
AM-Duftontainer
auf X-Petrischale
Der 2. und 3. Trainingsdurchgang wird entsprechend durchgeführt.

## Slide 14
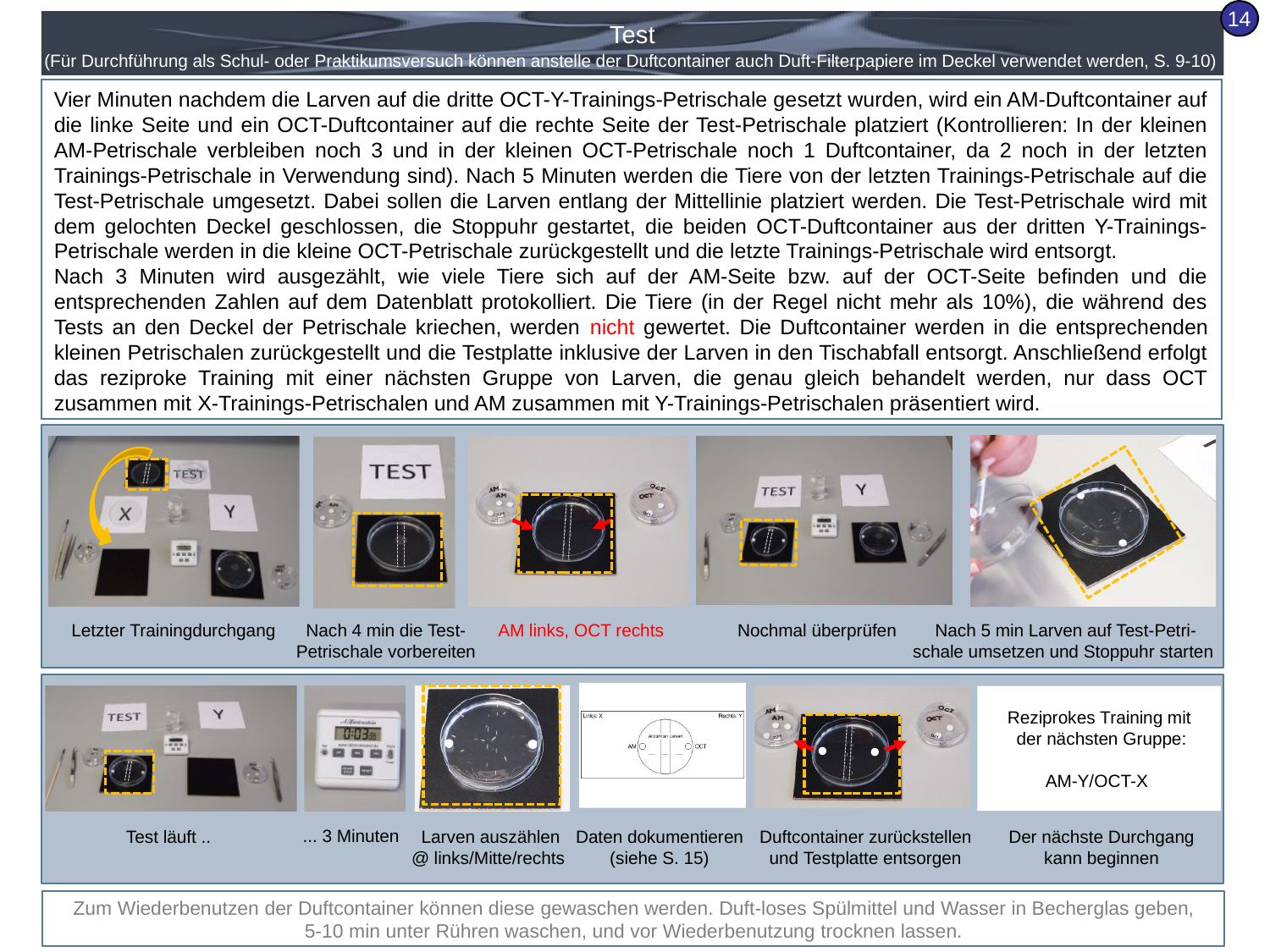

14
# Test
(Für Durchführung als Schul- oder Praktikumsversuch können anstelle der Duftcontainer auch Duft-Filterpapiere im Deckel verwendet werden, S. 9-10)
Vier Minuten nachdem die Larven auf die dritte OCT-Y-Trainings-Petrischale gesetzt wurden, wird ein AM-Duftcontainer auf die linke Seite und ein OCT-Duftcontainer auf die rechte Seite der Test-Petrischale platziert (Kontrollieren: In der kleinen AM-Petrischale verbleiben noch 3 und in der kleinen OCT-Petrischale noch 1 Duftcontainer, da 2 noch in der letzten Trainings-Petrischale in Verwendung sind). Nach 5 Minuten werden die Tiere von der letzten Trainings-Petrischale auf die Test-Petrischale umgesetzt. Dabei sollen die Larven entlang der Mittellinie platziert werden. Die Test-Petrischale wird mit dem gelochten Deckel geschlossen, die Stoppuhr gestartet, die beiden OCT-Duftcontainer aus der dritten Y-Trainings-Petrischale werden in die kleine OCT-Petrischale zurückgestellt und die letzte Trainings-Petrischale wird entsorgt.
Nach 3 Minuten wird ausgezählt, wie viele Tiere sich auf der AM-Seite bzw. auf der OCT-Seite befinden und die entsprechenden Zahlen auf dem Datenblatt protokolliert. Die Tiere (in der Regel nicht mehr als 10%), die während des Tests an den Deckel der Petrischale kriechen, werden nicht gewertet. Die Duftcontainer werden in die entsprechenden kleinen Petrischalen zurückgestellt und die Testplatte inklusive der Larven in den Tischabfall entsorgt. Anschließend erfolgt das reziproke Training mit einer nächsten Gruppe von Larven, die genau gleich behandelt werden, nur dass OCT zusammen mit X-Trainings-Petrischalen und AM zusammen mit Y-Trainings-Petrischalen präsentiert wird.
Letzter Trainingdurchgang
Nach 4 min die Test-
Petrischale vorbereiten
AM links, OCT rechts
Nochmal überprüfen
Nach 5 min Larven auf Test-Petri-schale umsetzen und Stoppuhr starten
Reziprokes Training mit
 der nächsten Gruppe:
AM-Y/OCT-X
... 3 Minuten
Test läuft ..
Larven auszählen
@ links/Mitte/rechts
Daten dokumentieren
(siehe S. 15)
Duftcontainer zurückstellen und Testplatte entsorgen
Der nächste Durchgang kann beginnen
Zum Wiederbenutzen der Duftcontainer können diese gewaschen werden. Duft-loses Spülmittel und Wasser in Becherglas geben,
5-10 min unter Rühren waschen, und vor Wiederbenutzung trocknen lassen.

## Slide 15
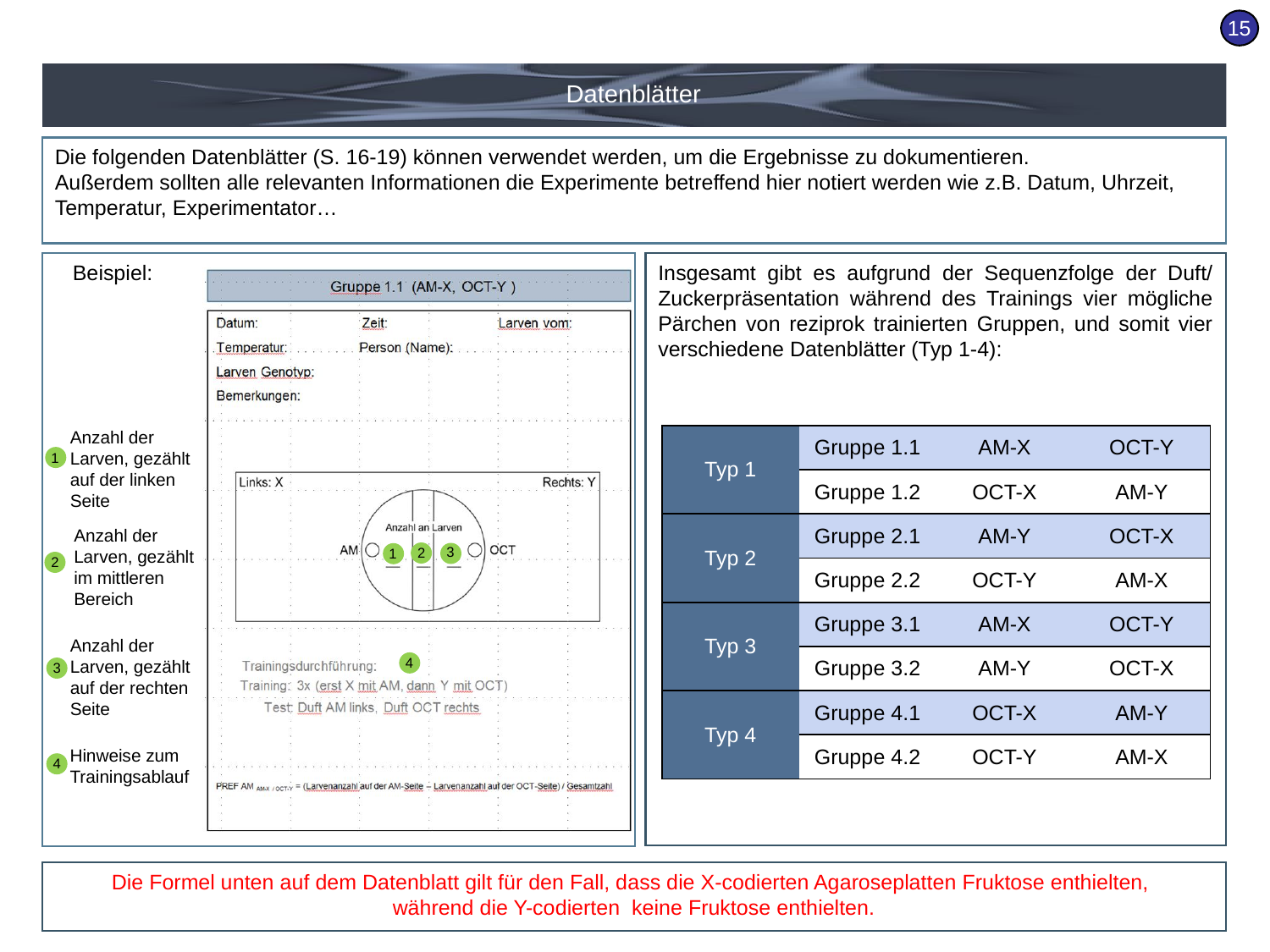

15
Datenblätter
Die folgenden Datenblätter (S. 16-19) können verwendet werden, um die Ergebnisse zu dokumentieren.
Außerdem sollten alle relevanten Informationen die Experimente betreffend hier notiert werden wie z.B. Datum, Uhrzeit, Temperatur, Experimentator…
 Beispiel:
Insgesamt gibt es aufgrund der Sequenzfolge der Duft/ Zuckerpräsentation während des Trainings vier mögliche Pärchen von reziprok trainierten Gruppen, und somit vier verschiedene Datenblätter (Typ 1-4):
Anzahl der Larven, gezählt auf der linken Seite
| Typ 1 | Gruppe 1.1 | AM-X | OCT-Y |
| --- | --- | --- | --- |
| | Gruppe 1.2 | OCT-X | AM-Y |
| Typ 2 | Gruppe 2.1 | AM-Y | OCT-X |
| | Gruppe 2.2 | OCT-Y | AM-X |
| Typ 3 | Gruppe 3.1 | AM-X | OCT-Y |
| | Gruppe 3.2 | AM-Y | OCT-X |
| Typ 4 | Gruppe 4.1 | OCT-X | AM-Y |
| | Gruppe 4.2 | OCT-Y | AM-X |
1
Anzahl der Larven, gezählt im mittleren Bereich
3
2
1
2
Anzahl der Larven, gezählt auf der rechten Seite
4
3
Hinweise zum Trainingsablauf
4
Die Formel unten auf dem Datenblatt gilt für den Fall, dass die X-codierten Agaroseplatten Fruktose enthielten,
während die Y-codierten keine Fruktose enthielten.

## Slide 16
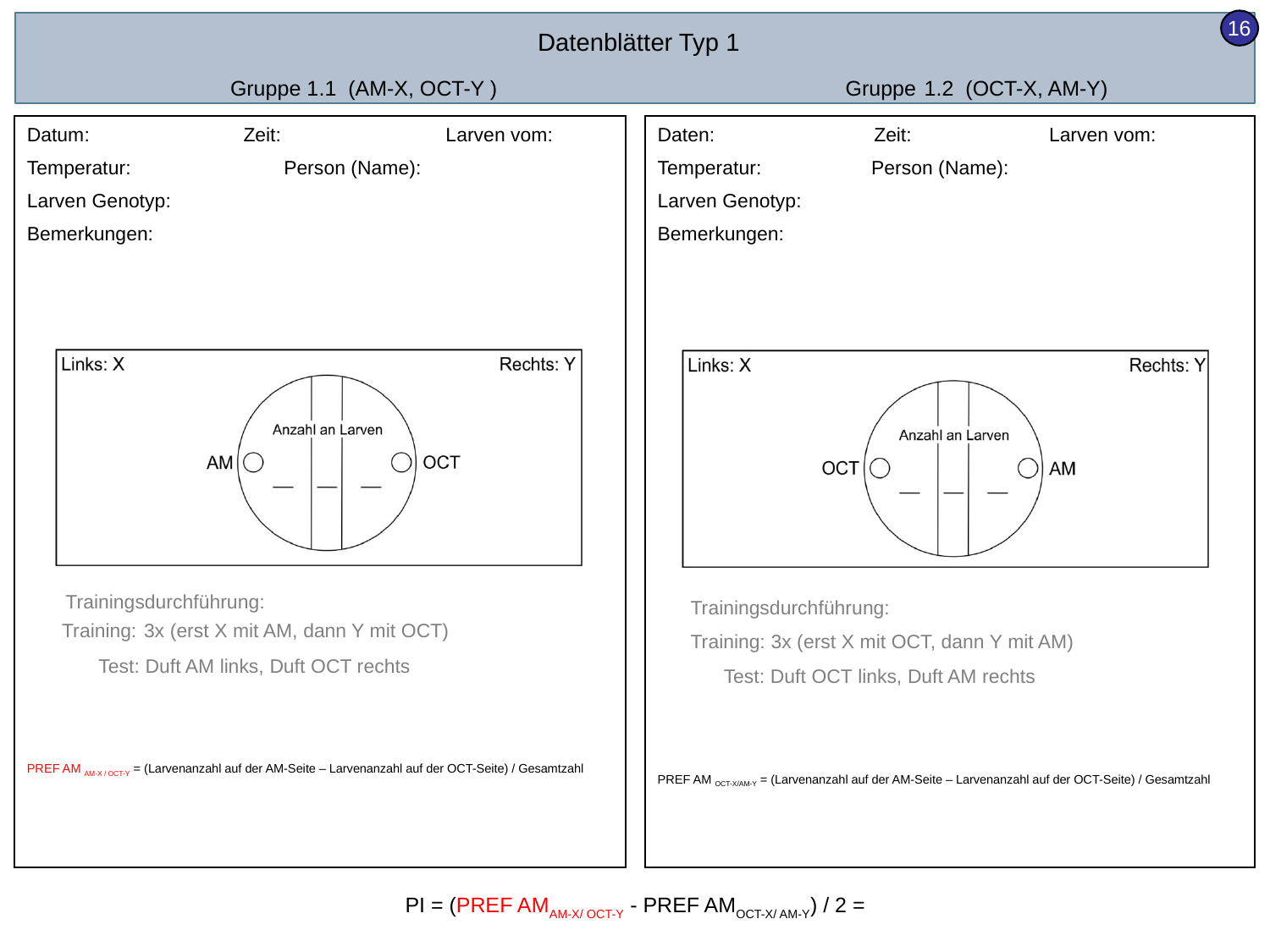

16
# Datenblätter Typ 1 Gruppe 1.1 (AM-X, OCT-Y ) Gruppe 1.2 (OCT-X, AM-Y)
Datum: Zeit: Larven vom:
Temperatur: 	 Person (Name):
Larven Genotyp:
Bemerkungen:
 Trainingsdurchführung:
 Training: 3x (erst X mit AM, dann Y mit OCT)
 Test: Duft AM links, Duft OCT rechts
PREF AM AM-X / OCT-Y = (Larvenanzahl auf der AM-Seite – Larvenanzahl auf der OCT-Seite) / Gesamtzahl
Daten: Zeit: Larven vom:
Temperatur: Person (Name):
Larven Genotyp:
Bemerkungen:
 Trainingsdurchführung:
 Training: 3x (erst X mit OCT, dann Y mit AM)
 Test: Duft OCT links, Duft AM rechts
PREF AM OCT-X/AM-Y = (Larvenanzahl auf der AM-Seite – Larvenanzahl auf der OCT-Seite) / Gesamtzahl
PI = (PREF AMAM-X/ OCT-Y - PREF AMOCT-X/ AM-Y) / 2 =

## Slide 17
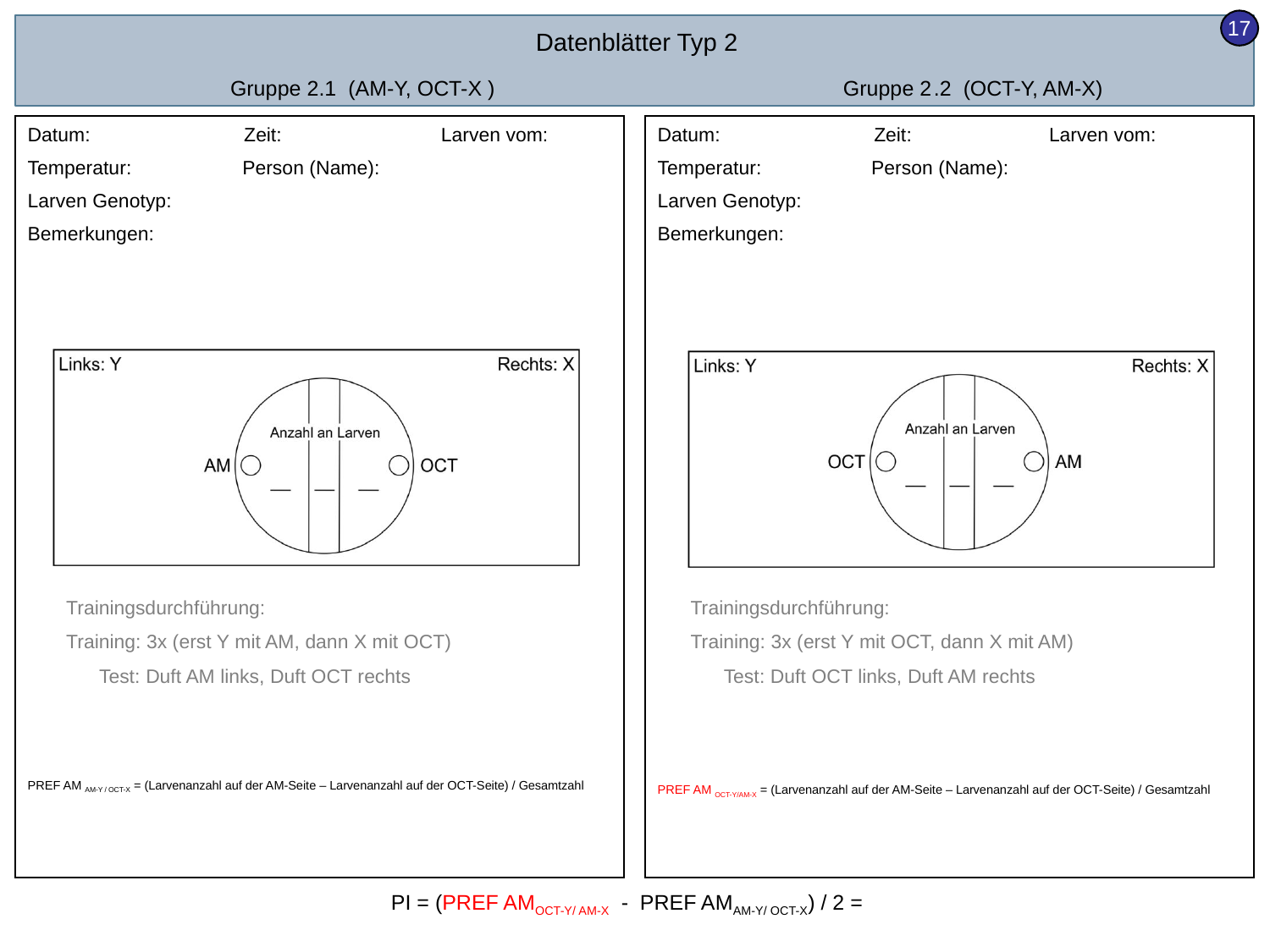

17
				Datenblätter Typ 2
 Gruppe 2.1 (AM-Y, OCT-X ) Gruppe 2.2 (OCT-Y, AM-X)
Datum: Zeit: Larven vom:
Temperatur: 	 Person (Name):
Larven Genotyp:
Bemerkungen:
 Trainingsdurchführung:
 Training: 3x (erst Y mit AM, dann X mit OCT)
 Test: Duft AM links, Duft OCT rechts
PREF AM AM-Y / OCT-X = (Larvenanzahl auf der AM-Seite – Larvenanzahl auf der OCT-Seite) / Gesamtzahl
Datum: Zeit: Larven vom:
Temperatur: Person (Name):
Larven Genotyp:
Bemerkungen:
 Trainingsdurchführung:
 Training: 3x (erst Y mit OCT, dann X mit AM)
 Test: Duft OCT links, Duft AM rechts
PREF AM OCT-Y/AM-X = (Larvenanzahl auf der AM-Seite – Larvenanzahl auf der OCT-Seite) / Gesamtzahl
PI = (PREF AMOCT-Y/ AM-X - PREF AMAM-Y/ OCT-X) / 2 =

## Slide 18
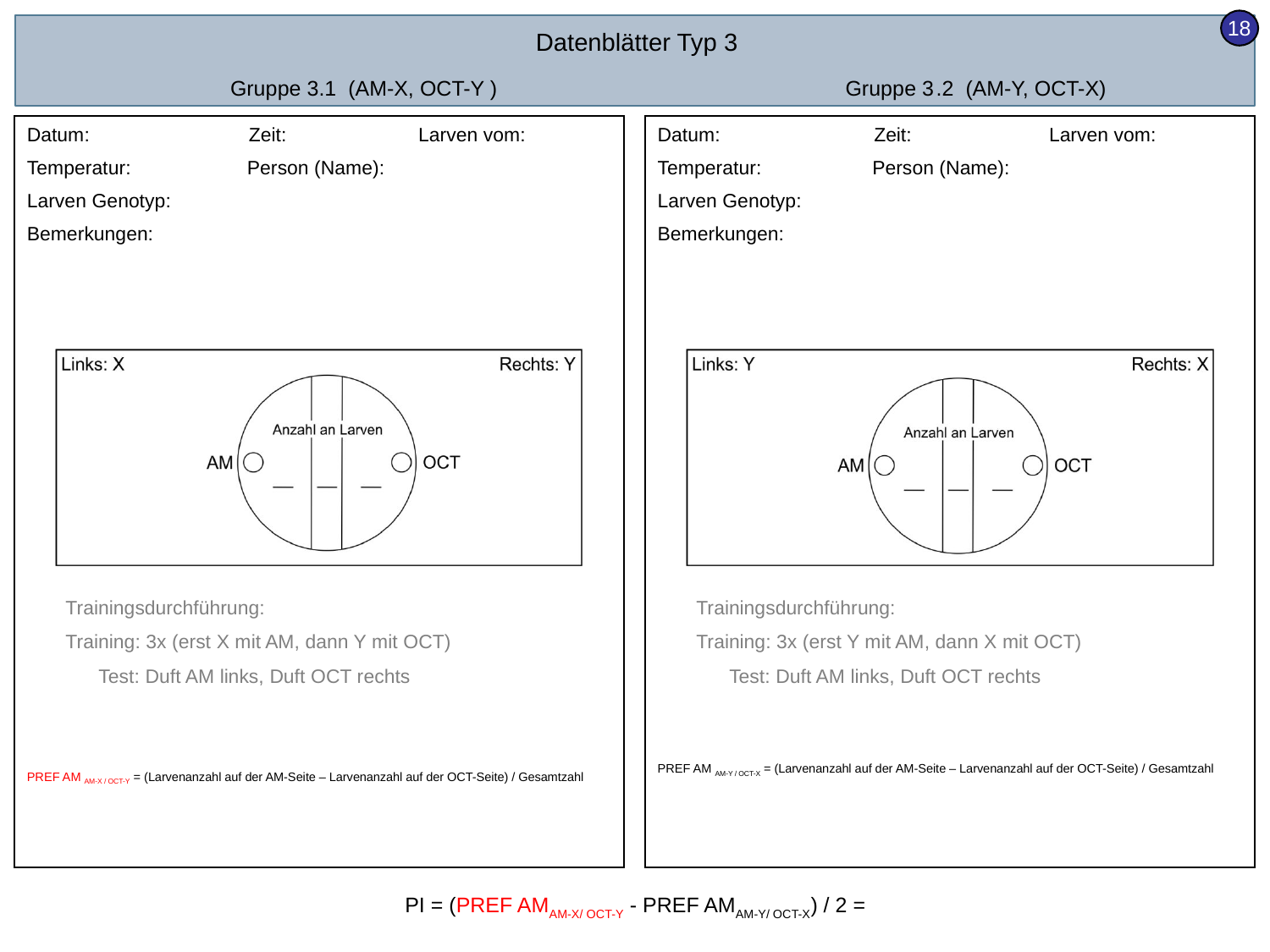

18
				Datenblätter Typ 3
 Gruppe 3.1 (AM-X, OCT-Y ) Gruppe 3.2 (AM-Y, OCT-X)
Datum: Zeit: Larven vom:
Temperatur: 	 Person (Name):
Larven Genotyp:
Bemerkungen:
 Trainingsdurchführung:
 Training: 3x (erst X mit AM, dann Y mit OCT)
 Test: Duft AM links, Duft OCT rechts
PREF AM AM-X / OCT-Y = (Larvenanzahl auf der AM-Seite – Larvenanzahl auf der OCT-Seite) / Gesamtzahl
Datum: Zeit: Larven vom:
Temperatur: 	 Person (Name):
Larven Genotyp:
Bemerkungen:
 Trainingsdurchführung:
 Training: 3x (erst Y mit AM, dann X mit OCT)
 Test: Duft AM links, Duft OCT rechts
PREF AM AM-Y / OCT-X = (Larvenanzahl auf der AM-Seite – Larvenanzahl auf der OCT-Seite) / Gesamtzahl
PI = (PREF AMAM-X/ OCT-Y - PREF AMAM-Y/ OCT-X) / 2 =

## Slide 19
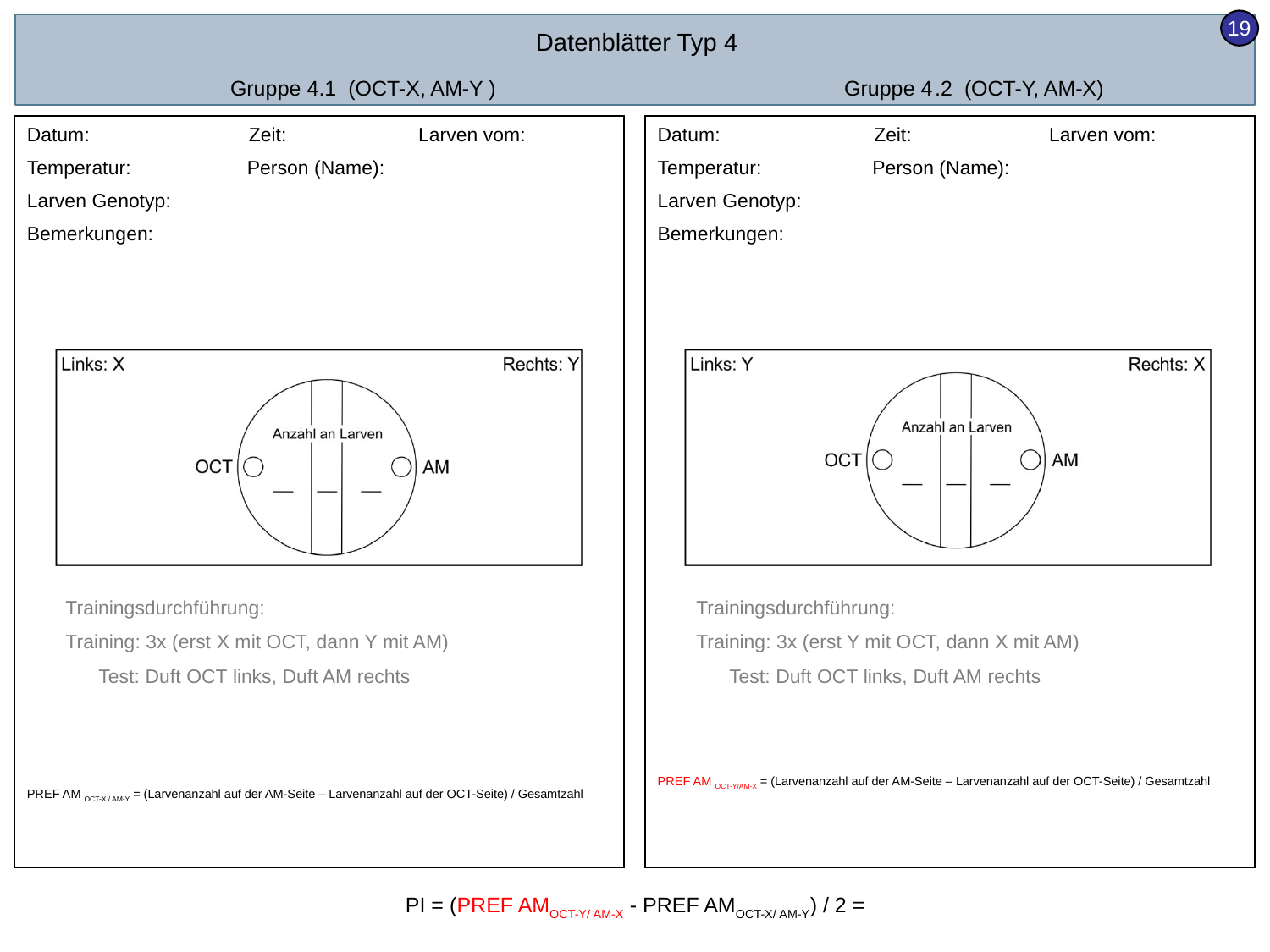

19
				Datenblätter Typ 4
 Gruppe 4.1 (OCT-X, AM-Y ) Gruppe 4.2 (OCT-Y, AM-X)
Datum: Zeit: Larven vom:
Temperatur: 	 Person (Name):
Larven Genotyp:
Bemerkungen:
 Trainingsdurchführung:
 Training: 3x (erst X mit OCT, dann Y mit AM)
 Test: Duft OCT links, Duft AM rechts
PREF AM OCT-X / AM-Y = (Larvenanzahl auf der AM-Seite – Larvenanzahl auf der OCT-Seite) / Gesamtzahl
Datum: Zeit: Larven vom:
Temperatur: 	 Person (Name):
Larven Genotyp:
Bemerkungen:
 Trainingsdurchführung:
 Training: 3x (erst Y mit OCT, dann X mit AM)
 Test: Duft OCT links, Duft AM rechts
PREF AM OCT-Y/AM-X = (Larvenanzahl auf der AM-Seite – Larvenanzahl auf der OCT-Seite) / Gesamtzahl
PI = (PREF AMOCT-Y/ AM-X - PREF AMOCT-X/ AM-Y) / 2 =

## Slide 20
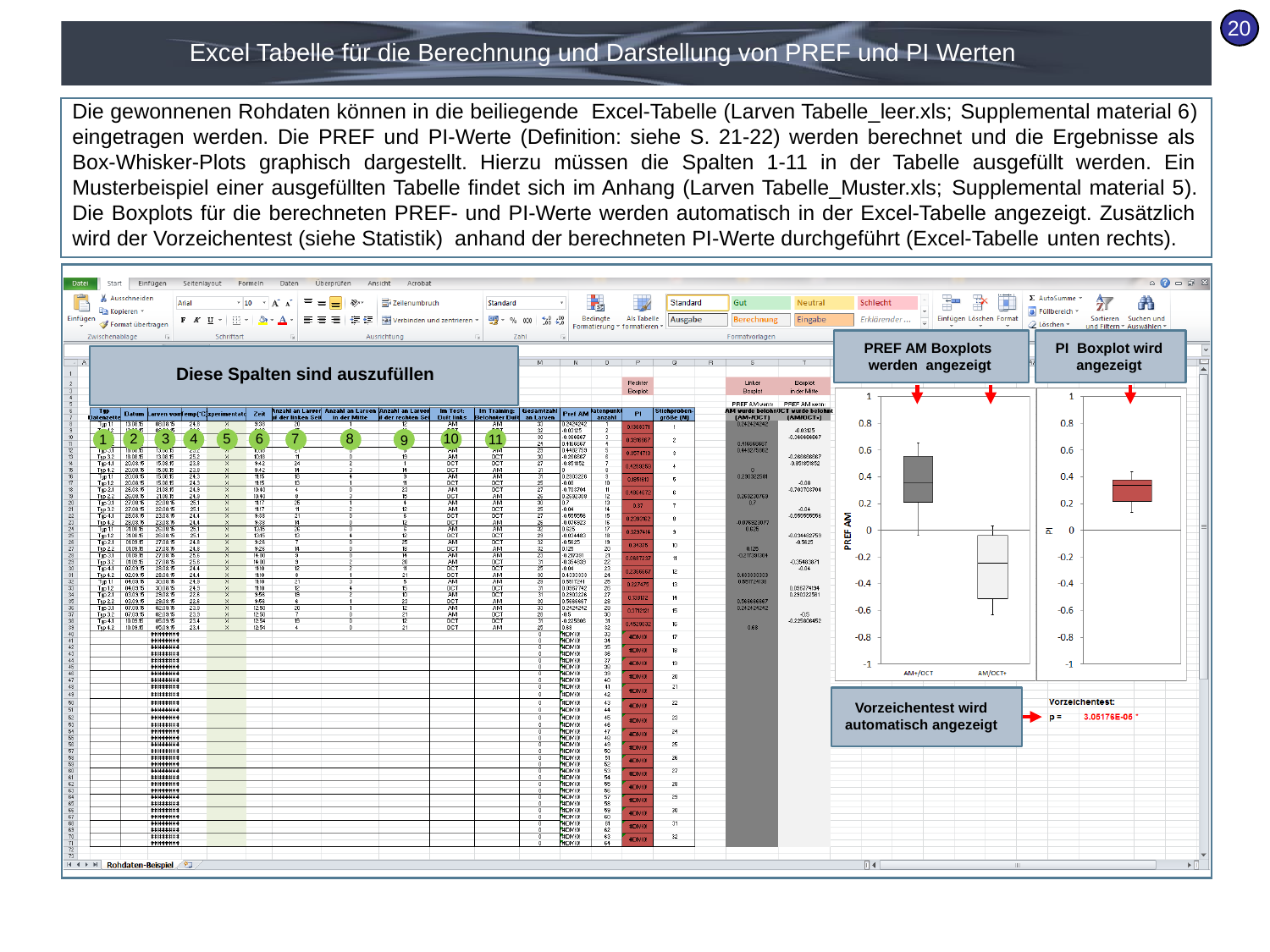

20
Excel Tabelle für die Berechnung und Darstellung von PREF und PI Werten
Die gewonnenen Rohdaten können in die beiliegende Excel-Tabelle (Larven Tabelle_leer.xls; Supplemental material 6) eingetragen werden. Die PREF und PI-Werte (Definition: siehe S. 21-22) werden berechnet und die Ergebnisse als Box-Whisker-Plots graphisch dargestellt. Hierzu müssen die Spalten 1-11 in der Tabelle ausgefüllt werden. Ein Musterbeispiel einer ausgefüllten Tabelle findet sich im Anhang (Larven Tabelle_Muster.xls; Supplemental material 5). Die Boxplots für die berechneten PREF- und PI-Werte werden automatisch in der Excel-Tabelle angezeigt. Zusätzlich wird der Vorzeichentest (siehe Statistik) anhand der berechneten PI-Werte durchgeführt (Excel-Tabelle unten rechts).
PREF AM Boxplots
werden angezeigt
PI Boxplot wird angezeigt
Diese Spalten sind auszufüllen
2
3
4
5
6
7
8
10
1
11
9
Vorzeichentest wird automatisch angezeigt

## Slide 21
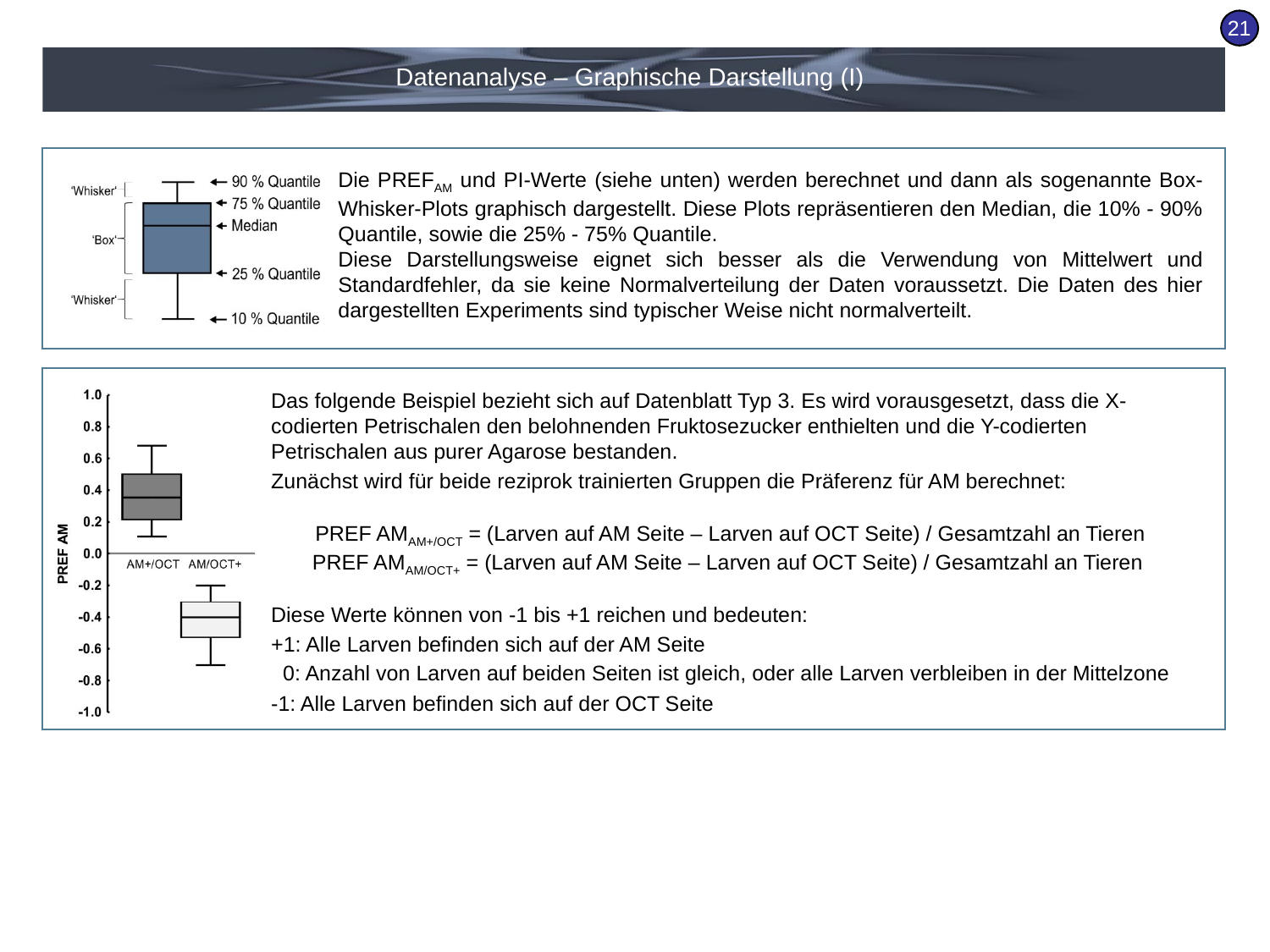

21
Datenanalyse – Graphische Darstellung (I)
Die PREFAM und PI-Werte (siehe unten) werden berechnet und dann als sogenannte Box-Whisker-Plots graphisch dargestellt. Diese Plots repräsentieren den Median, die 10% - 90% Quantile, sowie die 25% - 75% Quantile.
Diese Darstellungsweise eignet sich besser als die Verwendung von Mittelwert und Standardfehler, da sie keine Normalverteilung der Daten voraussetzt. Die Daten des hier dargestellten Experiments sind typischer Weise nicht normalverteilt.
Das folgende Beispiel bezieht sich auf Datenblatt Typ 3. Es wird vorausgesetzt, dass die X-codierten Petrischalen den belohnenden Fruktosezucker enthielten und die Y-codierten Petrischalen aus purer Agarose bestanden.
Zunächst wird für beide reziprok trainierten Gruppen die Präferenz für AM berechnet:
 PREF AMAM+/OCT = (Larven auf AM Seite – Larven auf OCT Seite) / Gesamtzahl an Tieren
 PREF AMAM/OCT+ = (Larven auf AM Seite – Larven auf OCT Seite) / Gesamtzahl an Tieren
Diese Werte können von -1 bis +1 reichen und bedeuten:
+1: Alle Larven befinden sich auf der AM Seite
 0: Anzahl von Larven auf beiden Seiten ist gleich, oder alle Larven verbleiben in der Mittelzone
-1: Alle Larven befinden sich auf der OCT Seite

## Slide 22
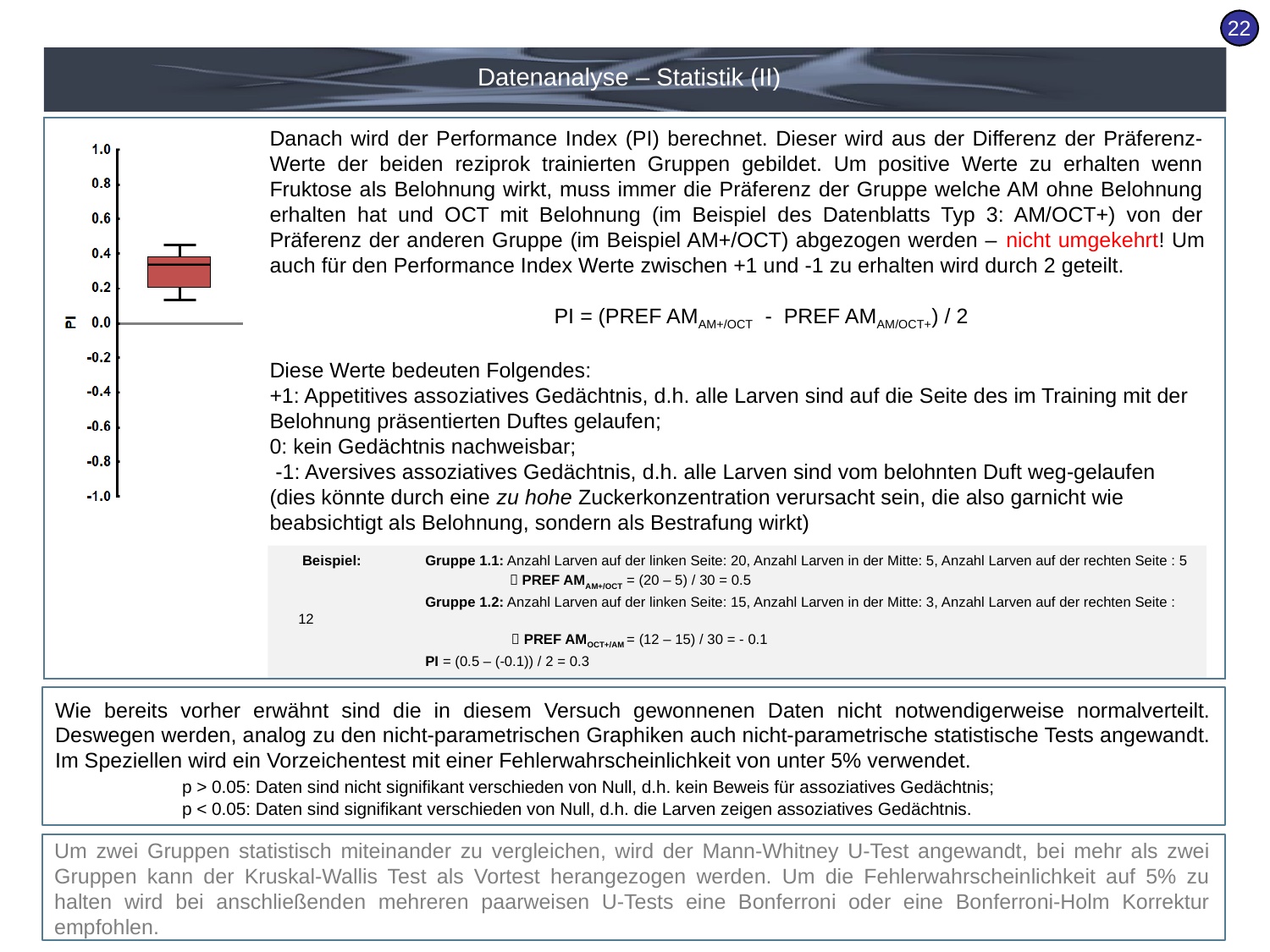

22
Datenanalyse – Statistik (II)
Danach wird der Performance Index (PI) berechnet. Dieser wird aus der Differenz der Präferenz-Werte der beiden reziprok trainierten Gruppen gebildet. Um positive Werte zu erhalten wenn Fruktose als Belohnung wirkt, muss immer die Präferenz der Gruppe welche AM ohne Belohnung erhalten hat und OCT mit Belohnung (im Beispiel des Datenblatts Typ 3: AM/OCT+) von der Präferenz der anderen Gruppe (im Beispiel AM+/OCT) abgezogen werden – nicht umgekehrt! Um auch für den Performance Index Werte zwischen +1 und -1 zu erhalten wird durch 2 geteilt.
 PI = (PREF AMAM+/OCT - PREF AMAM/OCT+) / 2
Diese Werte bedeuten Folgendes:
+1: Appetitives assoziatives Gedächtnis, d.h. alle Larven sind auf die Seite des im Training mit der Belohnung präsentierten Duftes gelaufen;
0: kein Gedächtnis nachweisbar;
 -1: Aversives assoziatives Gedächtnis, d.h. alle Larven sind vom belohnten Duft weg-gelaufen (dies könnte durch eine zu hohe Zuckerkonzentration verursacht sein, die also garnicht wie beabsichtigt als Belohnung, sondern als Bestrafung wirkt)
 Beispiel: 	Gruppe 1.1: Anzahl Larven auf der linken Seite: 20, Anzahl Larven in der Mitte: 5, Anzahl Larven auf der rechten Seite : 5
	  PREF AMAM+/OCT = (20 – 5) / 30 = 0.5
	Gruppe 1.2: Anzahl Larven auf der linken Seite: 15, Anzahl Larven in der Mitte: 3, Anzahl Larven auf der rechten Seite : 12
  PREF AMOCT+/AM = (12 – 15) / 30 = - 0.1
	PI = (0.5 – (-0.1)) / 2 = 0.3
Wie bereits vorher erwähnt sind die in diesem Versuch gewonnenen Daten nicht notwendigerweise normalverteilt. Deswegen werden, analog zu den nicht-parametrischen Graphiken auch nicht-parametrische statistische Tests angewandt. Im Speziellen wird ein Vorzeichentest mit einer Fehlerwahrscheinlichkeit von unter 5% verwendet.
	p > 0.05: Daten sind nicht signifikant verschieden von Null, d.h. kein Beweis für assoziatives Gedächtnis;
	p < 0.05: Daten sind signifikant verschieden von Null, d.h. die Larven zeigen assoziatives Gedächtnis.
Um zwei Gruppen statistisch miteinander zu vergleichen, wird der Mann-Whitney U-Test angewandt, bei mehr als zwei Gruppen kann der Kruskal-Wallis Test als Vortest herangezogen werden. Um die Fehlerwahrscheinlichkeit auf 5% zu halten wird bei anschließenden mehreren paarweisen U-Tests eine Bonferroni oder eine Bonferroni-Holm Korrektur empfohlen.

## Slide 23
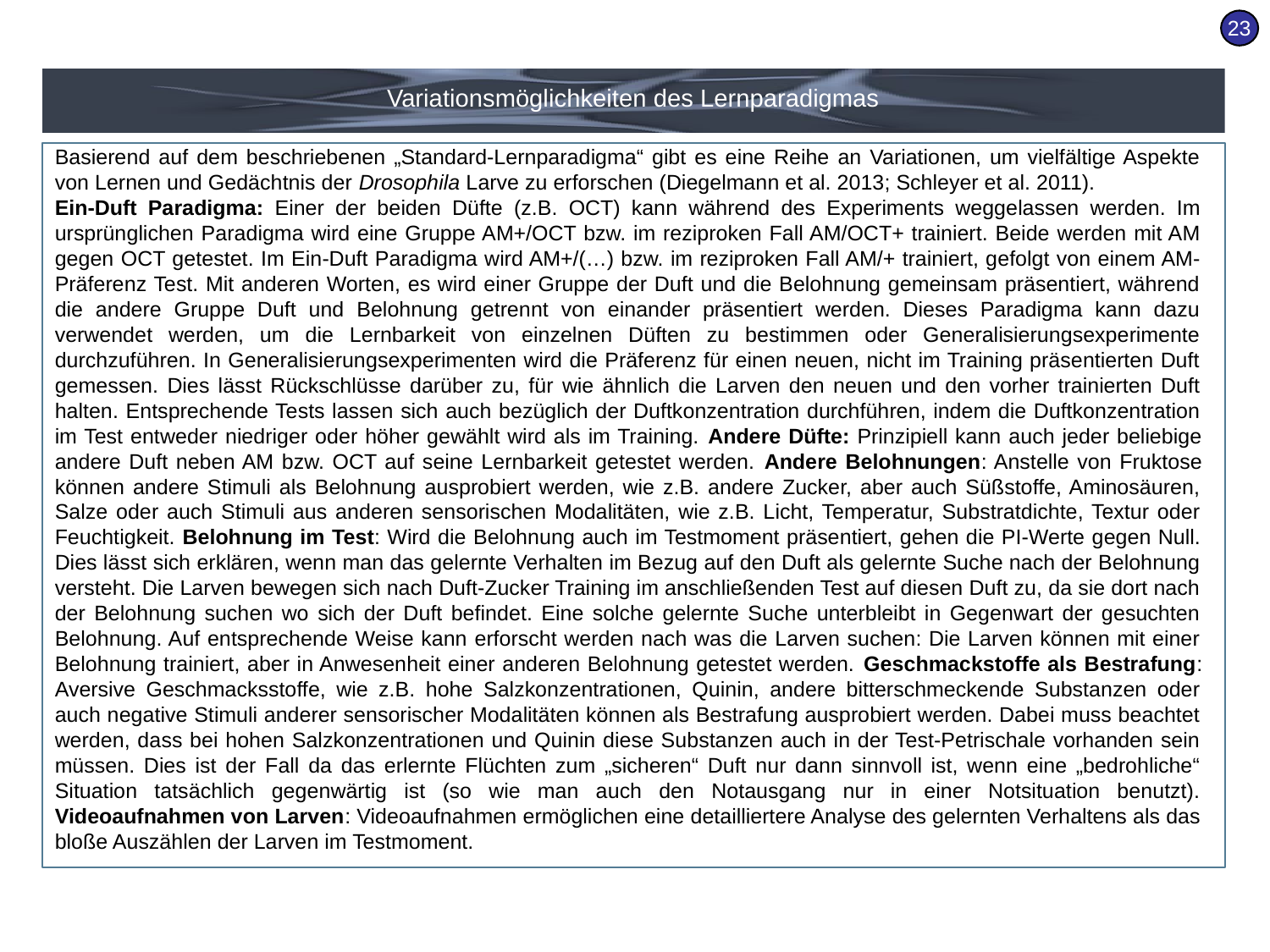

23
Variationsmöglichkeiten des Lernparadigmas
Basierend auf dem beschriebenen „Standard-Lernparadigma“ gibt es eine Reihe an Variationen, um vielfältige Aspekte von Lernen und Gedächtnis der Drosophila Larve zu erforschen (Diegelmann et al. 2013; Schleyer et al. 2011).
Ein-Duft Paradigma: Einer der beiden Düfte (z.B. OCT) kann während des Experiments weggelassen werden. Im ursprünglichen Paradigma wird eine Gruppe AM+/OCT bzw. im reziproken Fall AM/OCT+ trainiert. Beide werden mit AM gegen OCT getestet. Im Ein-Duft Paradigma wird AM+/(…) bzw. im reziproken Fall AM/+ trainiert, gefolgt von einem AM-Präferenz Test. Mit anderen Worten, es wird einer Gruppe der Duft und die Belohnung gemeinsam präsentiert, während die andere Gruppe Duft und Belohnung getrennt von einander präsentiert werden. Dieses Paradigma kann dazu verwendet werden, um die Lernbarkeit von einzelnen Düften zu bestimmen oder Generalisierungsexperimente durchzuführen. In Generalisierungsexperimenten wird die Präferenz für einen neuen, nicht im Training präsentierten Duft gemessen. Dies lässt Rückschlüsse darüber zu, für wie ähnlich die Larven den neuen und den vorher trainierten Duft halten. Entsprechende Tests lassen sich auch bezüglich der Duftkonzentration durchführen, indem die Duftkonzentration im Test entweder niedriger oder höher gewählt wird als im Training. Andere Düfte: Prinzipiell kann auch jeder beliebige andere Duft neben AM bzw. OCT auf seine Lernbarkeit getestet werden. Andere Belohnungen: Anstelle von Fruktose können andere Stimuli als Belohnung ausprobiert werden, wie z.B. andere Zucker, aber auch Süßstoffe, Aminosäuren, Salze oder auch Stimuli aus anderen sensorischen Modalitäten, wie z.B. Licht, Temperatur, Substratdichte, Textur oder Feuchtigkeit. Belohnung im Test: Wird die Belohnung auch im Testmoment präsentiert, gehen die PI-Werte gegen Null. Dies lässt sich erklären, wenn man das gelernte Verhalten im Bezug auf den Duft als gelernte Suche nach der Belohnung versteht. Die Larven bewegen sich nach Duft-Zucker Training im anschließenden Test auf diesen Duft zu, da sie dort nach der Belohnung suchen wo sich der Duft befindet. Eine solche gelernte Suche unterbleibt in Gegenwart der gesuchten Belohnung. Auf entsprechende Weise kann erforscht werden nach was die Larven suchen: Die Larven können mit einer Belohnung trainiert, aber in Anwesenheit einer anderen Belohnung getestet werden. Geschmackstoffe als Bestrafung: Aversive Geschmacksstoffe, wie z.B. hohe Salzkonzentrationen, Quinin, andere bitterschmeckende Substanzen oder auch negative Stimuli anderer sensorischer Modalitäten können als Bestrafung ausprobiert werden. Dabei muss beachtet werden, dass bei hohen Salzkonzentrationen und Quinin diese Substanzen auch in der Test-Petrischale vorhanden sein müssen. Dies ist der Fall da das erlernte Flüchten zum „sicheren“ Duft nur dann sinnvoll ist, wenn eine „bedrohliche“ Situation tatsächlich gegenwärtig ist (so wie man auch den Notausgang nur in einer Notsituation benutzt). Videoaufnahmen von Larven: Videoaufnahmen ermöglichen eine detailliertere Analyse des gelernten Verhaltens als das bloße Auszählen der Larven im Testmoment.

## Slide 24
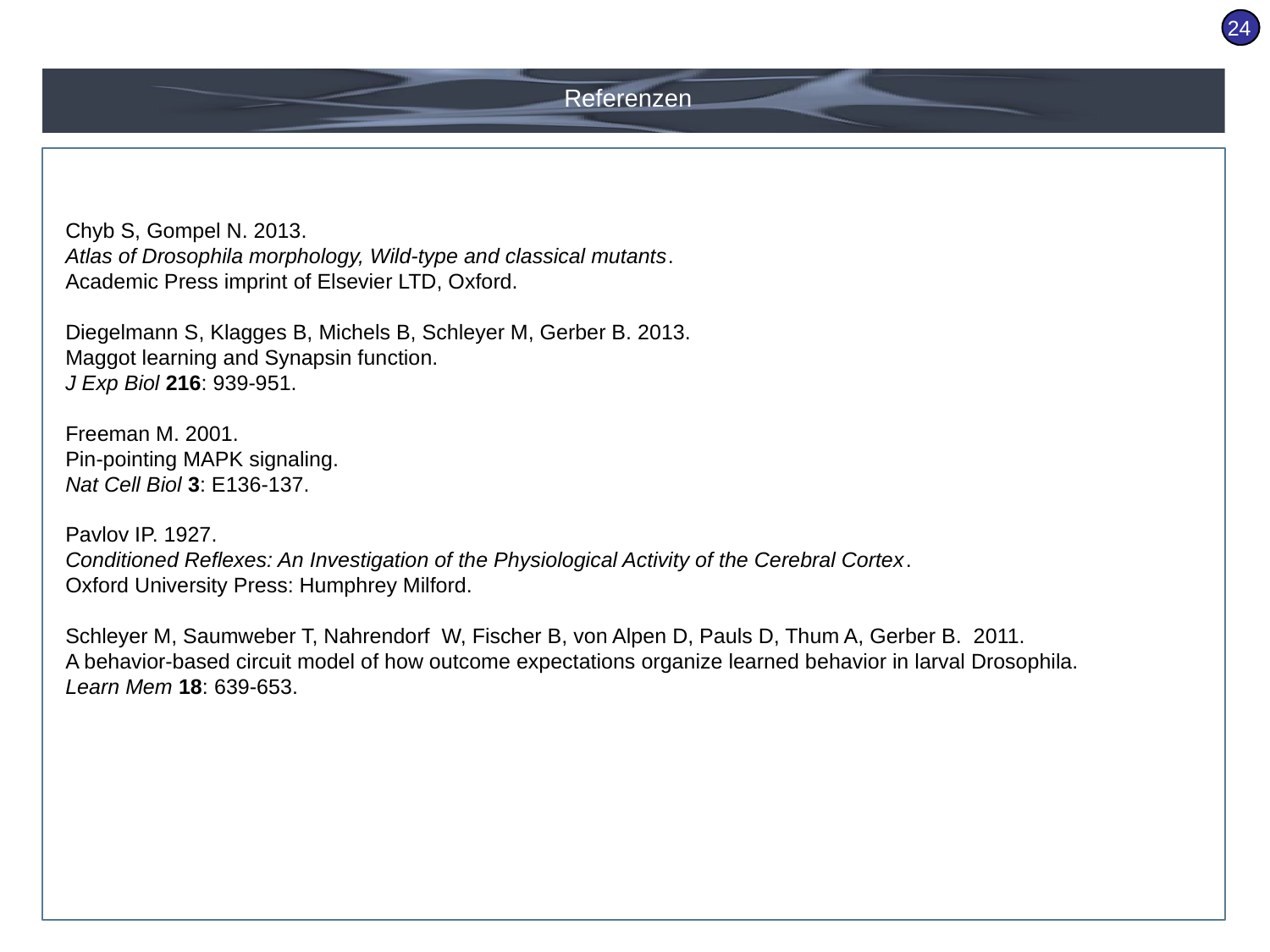

24
Referenzen
Chyb S, Gompel N. 2013.
Atlas of Drosophila morphology, Wild-type and classical mutants.
Academic Press imprint of Elsevier LTD, Oxford.
Diegelmann S, Klagges B, Michels B, Schleyer M, Gerber B. 2013.
Maggot learning and Synapsin function.
J Exp Biol 216: 939-951.
Freeman M. 2001.
Pin-pointing MAPK signaling.
Nat Cell Biol 3: E136-137.
Pavlov IP. 1927.
Conditioned Reflexes: An Investigation of the Physiological Activity of the Cerebral Cortex.
Oxford University Press: Humphrey Milford.
Schleyer M, Saumweber T, Nahrendorf W, Fischer B, von Alpen D, Pauls D, Thum A, Gerber B. 2011.
A behavior-based circuit model of how outcome expectations organize learned behavior in larval Drosophila.
Learn Mem 18: 639-653.
