# Supplementary material for: Pavlovian Conditioning of Larval Drosophila: An Illustrated, Multilingual, Hands-On Manual for Odor-Taste Associative Learning in Maggots
Source: Front Behav Neurosci. 2017 Apr 19;11:45. doi: 10.3389/fnbeh.2017.00045 (PMC5395560; doi:10.3389/fnbeh.2017.00045)
Supplement: Supplemental Materials 7–9 — A manual for odor-reward learning in larval Drosophila (Supplemental Material 7), example of a table for data analysis (Supplemental Material 8), and an empty table for entering and analyzing one's own data (Supplemental Material 9), in the French language. Versions of this manual in the English, German, Japanese, Spanish, and Italian languages can be found in Supplemental Materials 1–3, 4–6, 10, 11–13, 14–16, respectively. [file SupplementalMaterial7.pptx]

## Slide 1
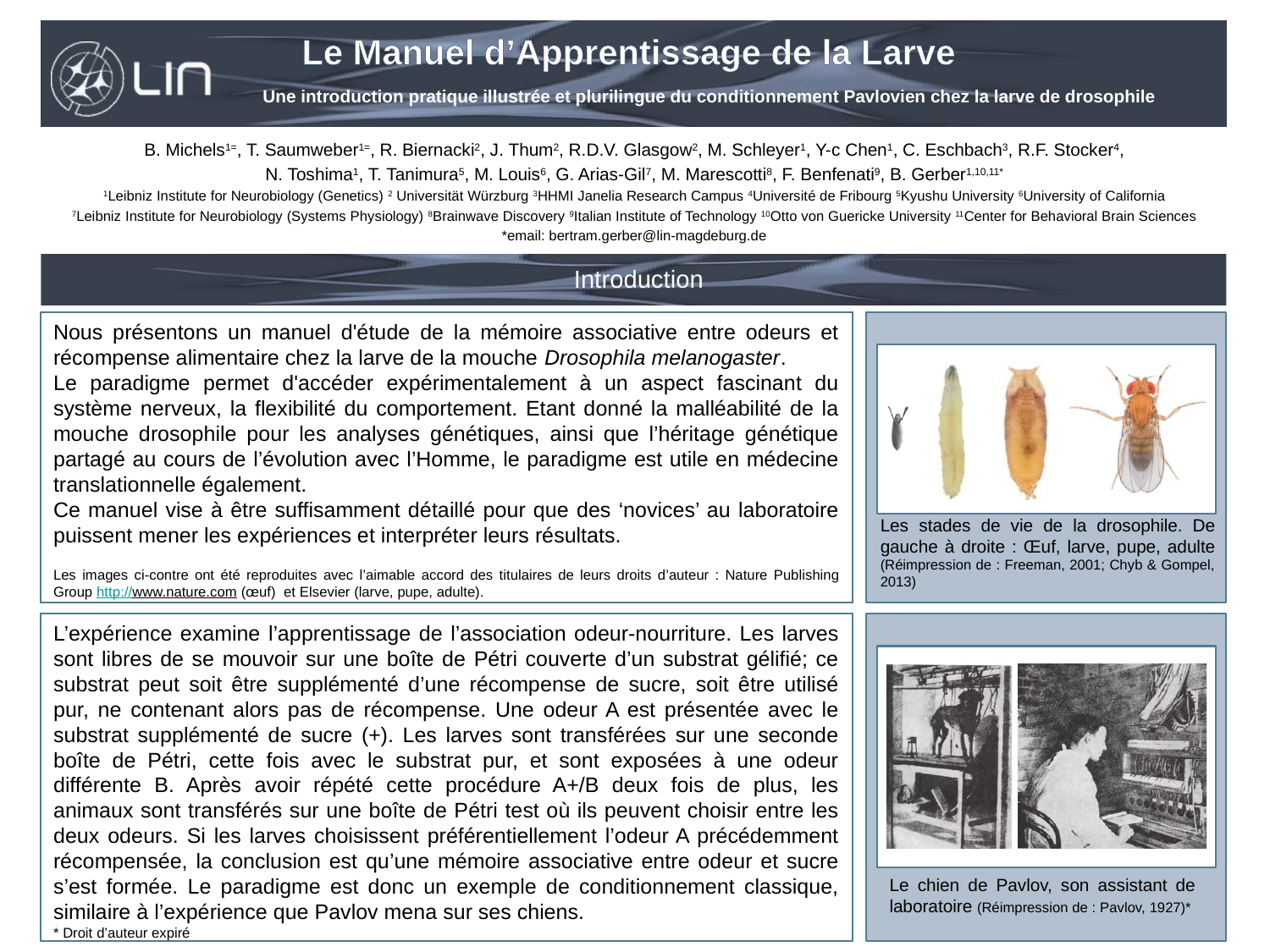

# Une introduction pratique illustrée et plurilingue du conditionnement Pavlovien chez la larve de drosophile
Le Manuel d’Apprentissage de la Larve
B. Michels1=, T. Saumweber1=, R. Biernacki2, J. Thum2, R.D.V. Glasgow2, M. Schleyer1, Y-c Chen1, C. Eschbach3, R.F. Stocker4,
N. Toshima1, T. Tanimura5, M. Louis6, G. Arias-Gil7, M. Marescotti8, F. Benfenati9, B. Gerber1,10,11*
1Leibniz Institute for Neurobiology (Genetics) 2 Universität Würzburg 3HHMI Janelia Research Campus 4Université de Fribourg 5Kyushu University 6University of California
7Leibniz Institute for Neurobiology (Systems Physiology) 8Brainwave Discovery 9Italian Institute of Technology 10Otto von Guericke University 11Center for Behavioral Brain Sciences
*email: bertram.gerber@lin-magdeburg.de
Introduction
Nous présentons un manuel d'étude de la mémoire associative entre odeurs et récompense alimentaire chez la larve de la mouche Drosophila melanogaster.
Le paradigme permet d'accéder expérimentalement à un aspect fascinant du système nerveux, la flexibilité du comportement. Etant donné la malléabilité de la mouche drosophile pour les analyses génétiques, ainsi que l’héritage génétique partagé au cours de l’évolution avec l’Homme, le paradigme est utile en médecine translationnelle également.
Ce manuel vise à être suffisamment détaillé pour que des ‘novices’ au laboratoire puissent mener les expériences et interpréter leurs résultats.
Les images ci-contre ont été reproduites avec l’aimable accord des titulaires de leurs droits d’auteur : Nature Publishing Group http://www.nature.com (œuf) et Elsevier (larve, pupe, adulte).
Les stades de vie de la drosophile. De gauche à droite : Œuf, larve, pupe, adulte (Réimpression de : Freeman, 2001; Chyb & Gompel, 2013)
L’expérience examine l’apprentissage de l’association odeur-nourriture. Les larves sont libres de se mouvoir sur une boîte de Pétri couverte d’un substrat gélifié; ce substrat peut soit être supplémenté d’une récompense de sucre, soit être utilisé pur, ne contenant alors pas de récompense. Une odeur A est présentée avec le substrat supplémenté de sucre (+). Les larves sont transférées sur une seconde boîte de Pétri, cette fois avec le substrat pur, et sont exposées à une odeur différente B. Après avoir répété cette procédure A+/B deux fois de plus, les animaux sont transférés sur une boîte de Pétri test où ils peuvent choisir entre les deux odeurs. Si les larves choisissent préférentiellement l’odeur A précédemment récompensée, la conclusion est qu’une mémoire associative entre odeur et sucre s’est formée. Le paradigme est donc un exemple de conditionnement classique, similaire à l’expérience que Pavlov mena sur ses chiens.
* Droit d’auteur expiré
Le chien de Pavlov, son assistant de laboratoire (Réimpression de : Pavlov, 1927)*

## Slide 2
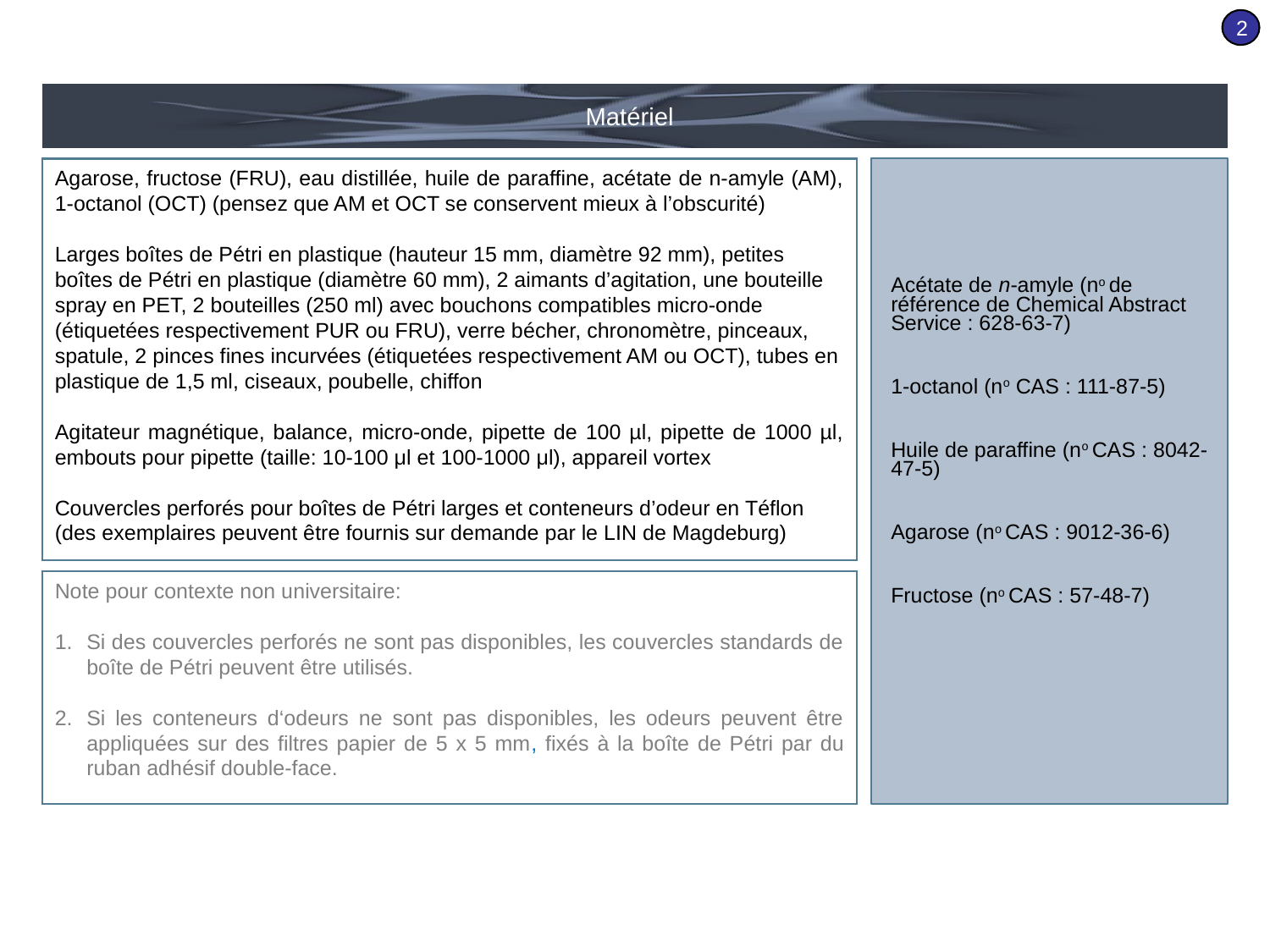

2
Matériel
Agarose, fructose (FRU), eau distillée, huile de paraffine, acétate de n-amyle (AM), 1-octanol (OCT) (pensez que AM et OCT se conservent mieux à l’obscurité)
Larges boîtes de Pétri en plastique (hauteur 15 mm, diamètre 92 mm), petites boîtes de Pétri en plastique (diamètre 60 mm), 2 aimants d’agitation, une bouteille spray en PET, 2 bouteilles (250 ml) avec bouchons compatibles micro-onde (étiquetées respectivement PUR ou FRU), verre bécher, chronomètre, pinceaux, spatule, 2 pinces fines incurvées (étiquetées respectivement AM ou OCT), tubes en plastique de 1,5 ml, ciseaux, poubelle, chiffon
Agitateur magnétique, balance, micro-onde, pipette de 100 µl, pipette de 1000 µl, embouts pour pipette (taille: 10-100 μl et 100-1000 μl), appareil vortex
Couvercles perforés pour boîtes de Pétri larges et conteneurs d’odeur en Téflon
(des exemplaires peuvent être fournis sur demande par le LIN de Magdeburg)
Acétate de n-amyle (no de référence de Chemical Abstract Service : 628-63-7)
1-octanol (no CAS : 111-87-5)
Huile de paraffine (no CAS : 8042-47-5)
Agarose (no CAS : 9012-36-6)
Fructose (no CAS : 57-48-7)
Note pour contexte non universitaire:
Si des couvercles perforés ne sont pas disponibles, les couvercles standards de boîte de Pétri peuvent être utilisés.
Si les conteneurs d‘odeurs ne sont pas disponibles, les odeurs peuvent être appliquées sur des filtres papier de 5 x 5 mm, fixés à la boîte de Pétri par du ruban adhésif double-face.

## Slide 3
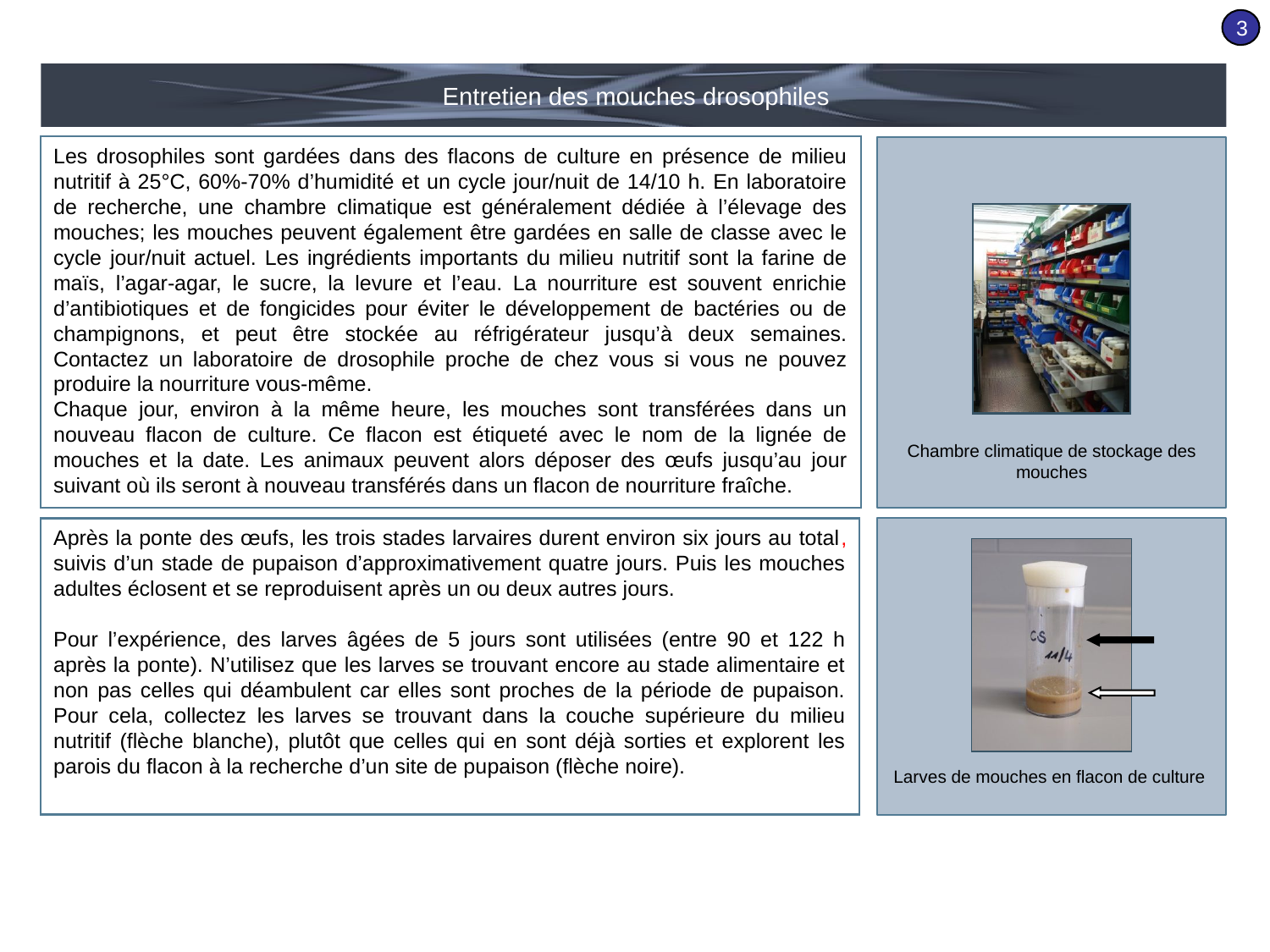

3
# Entretien des mouches drosophiles
Les drosophiles sont gardées dans des flacons de culture en présence de milieu nutritif à 25°C, 60%-70% d’humidité et un cycle jour/nuit de 14/10 h. En laboratoire de recherche, une chambre climatique est généralement dédiée à l’élevage des mouches; les mouches peuvent également être gardées en salle de classe avec le cycle jour/nuit actuel. Les ingrédients importants du milieu nutritif sont la farine de maïs, l’agar-agar, le sucre, la levure et l’eau. La nourriture est souvent enrichie d’antibiotiques et de fongicides pour éviter le développement de bactéries ou de champignons, et peut être stockée au réfrigérateur jusqu’à deux semaines. Contactez un laboratoire de drosophile proche de chez vous si vous ne pouvez produire la nourriture vous-même.
Chaque jour, environ à la même heure, les mouches sont transférées dans un nouveau flacon de culture. Ce flacon est étiqueté avec le nom de la lignée de mouches et la date. Les animaux peuvent alors déposer des œufs jusqu’au jour suivant où ils seront à nouveau transférés dans un flacon de nourriture fraîche.
Chambre climatique de stockage des mouches
Après la ponte des œufs, les trois stades larvaires durent environ six jours au total, suivis d’un stade de pupaison d’approximativement quatre jours. Puis les mouches adultes éclosent et se reproduisent après un ou deux autres jours.
Pour l’expérience, des larves âgées de 5 jours sont utilisées (entre 90 et 122 h après la ponte). N’utilisez que les larves se trouvant encore au stade alimentaire et non pas celles qui déambulent car elles sont proches de la période de pupaison. Pour cela, collectez les larves se trouvant dans la couche supérieure du milieu nutritif (flèche blanche), plutôt que celles qui en sont déjà sorties et explorent les parois du flacon à la recherche d’un site de pupaison (flèche noire).
Larves de mouches en flacon de culture

## Slide 4
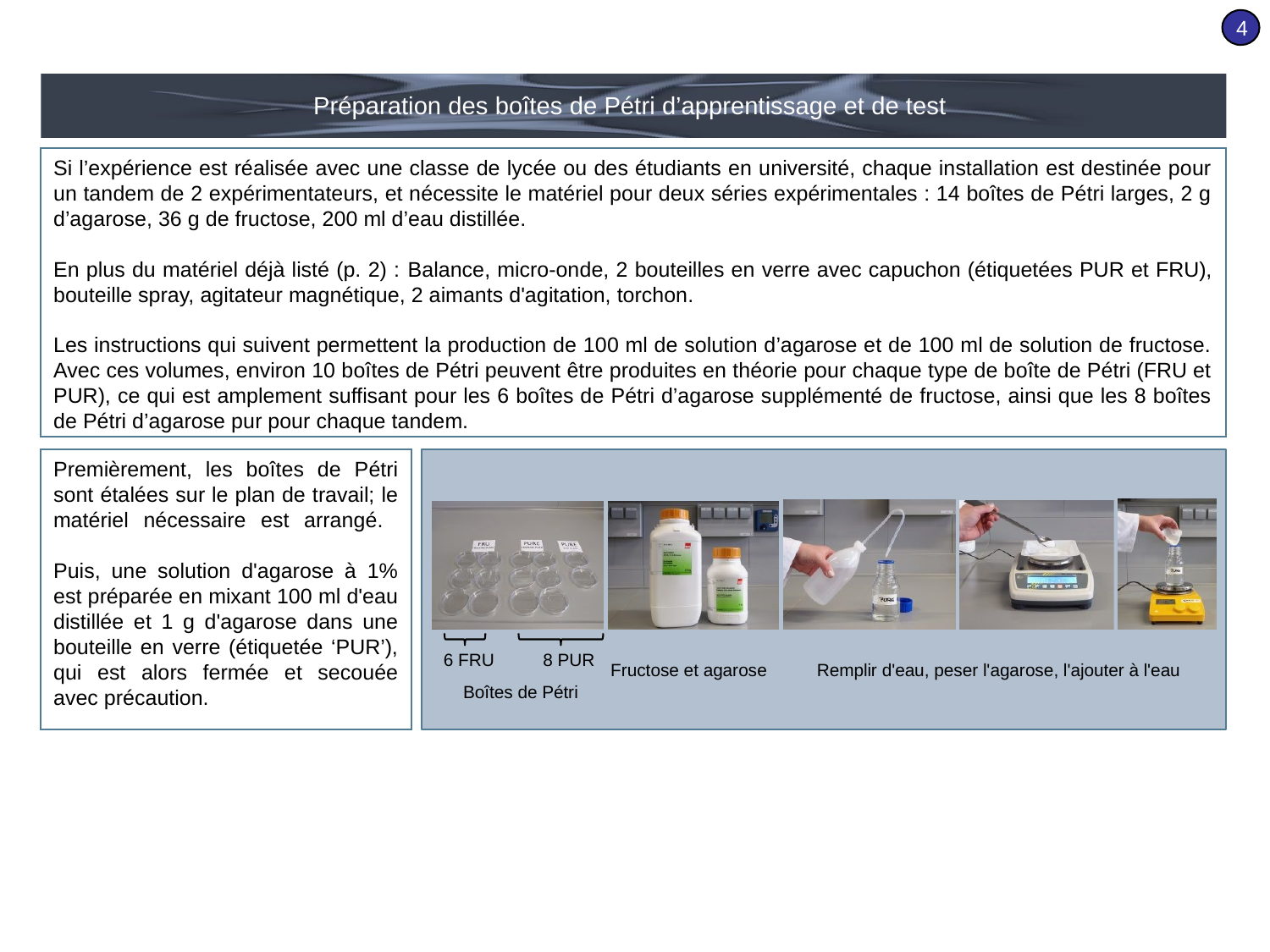

4
# Préparation des boîtes de Pétri d’apprentissage et de test
Si l’expérience est réalisée avec une classe de lycée ou des étudiants en université, chaque installation est destinée pour un tandem de 2 expérimentateurs, et nécessite le matériel pour deux séries expérimentales : 14 boîtes de Pétri larges, 2 g d’agarose, 36 g de fructose, 200 ml d’eau distillée.
En plus du matériel déjà listé (p. 2) : Balance, micro-onde, 2 bouteilles en verre avec capuchon (étiquetées PUR et FRU), bouteille spray, agitateur magnétique, 2 aimants d'agitation, torchon.
Les instructions qui suivent permettent la production de 100 ml de solution d’agarose et de 100 ml de solution de fructose. Avec ces volumes, environ 10 boîtes de Pétri peuvent être produites en théorie pour chaque type de boîte de Pétri (FRU et PUR), ce qui est amplement suffisant pour les 6 boîtes de Pétri d’agarose supplémenté de fructose, ainsi que les 8 boîtes de Pétri d’agarose pur pour chaque tandem.
Premièrement, les boîtes de Pétri sont étalées sur le plan de travail; le matériel nécessaire est arrangé.
Puis, une solution d'agarose à 1% est préparée en mixant 100 ml d'eau distillée et 1 g d'agarose dans une bouteille en verre (étiquetée ‘PUR’), qui est alors fermée et secouée avec précaution.
6 FRU 8 PUR
Fructose et agarose
Remplir d'eau, peser l'agarose, l'ajouter à l'eau
Boîtes de Pétri

## Slide 5
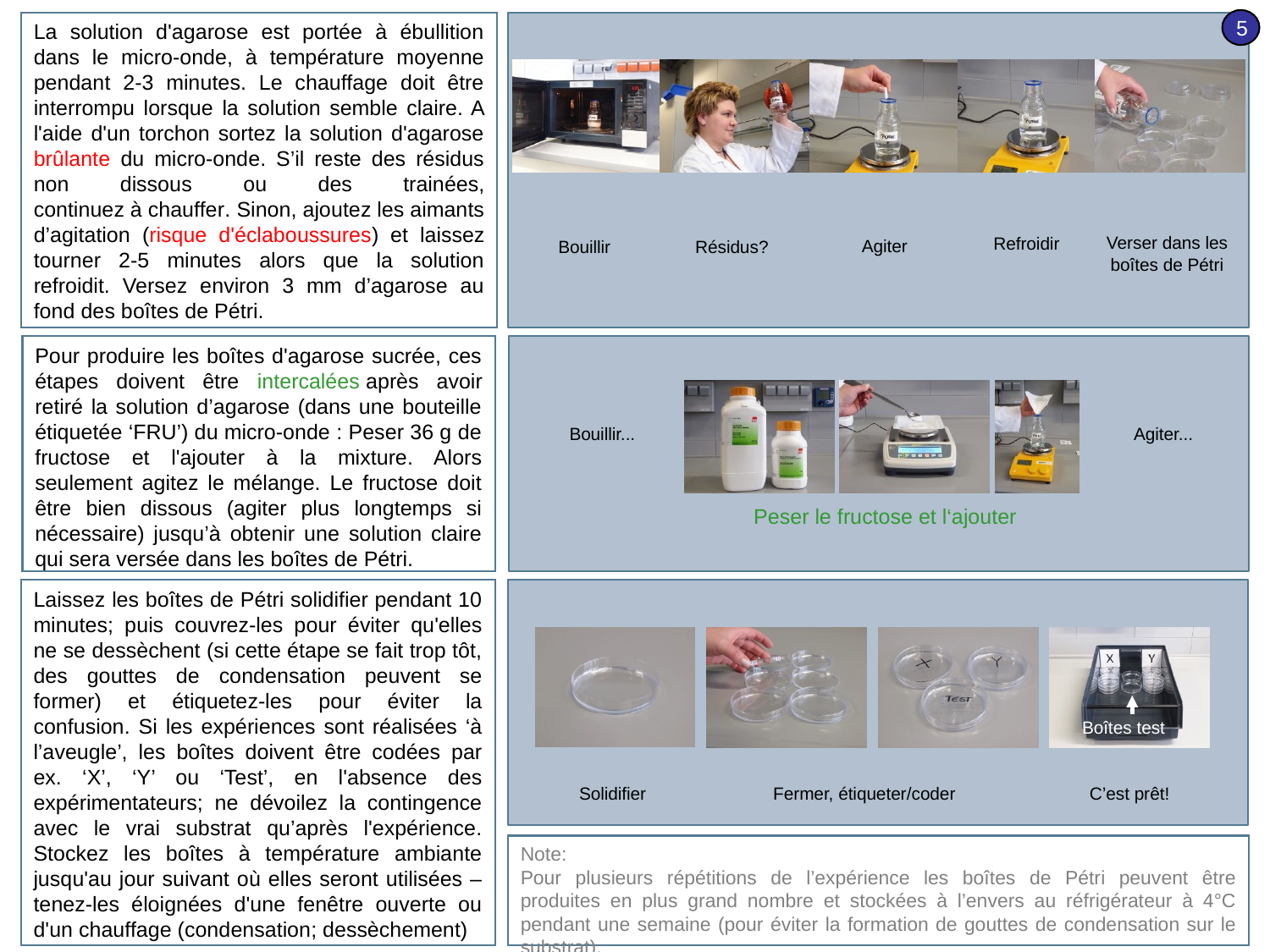

5
La solution d'agarose est portée à ébullition dans le micro-onde, à température moyenne pendant 2-3 minutes. Le chauffage doit être interrompu lorsque la solution semble claire. A l'aide d'un torchon sortez la solution d'agarose brûlante du micro-onde. S’il reste des résidus non dissous ou des trainées, continuez à chauffer. Sinon, ajoutez les aimants d’agitation (risque d'éclaboussures) et laissez tourner 2-5 minutes alors que la solution refroidit. Versez environ 3 mm d’agarose au fond des boîtes de Pétri.
Verser dans les
boîtes de Pétri
Résidus?
Refroidir
Agiter
Bouillir
Pour produire les boîtes d'agarose sucrée, ces étapes doivent être intercalées après avoir retiré la solution d’agarose (dans une bouteille étiquetée ‘FRU’) du micro-onde : Peser 36 g de fructose et l'ajouter à la mixture. Alors seulement agitez le mélange. Le fructose doit être bien dissous (agiter plus longtemps si nécessaire) jusqu’à obtenir une solution claire qui sera versée dans les boîtes de Pétri.
Bouillir...
Agiter...
Peser le fructose et l‘ajouter
Laissez les boîtes de Pétri solidifier pendant 10 minutes; puis couvrez-les pour éviter qu'elles ne se dessèchent (si cette étape se fait trop tôt, des gouttes de condensation peuvent se former) et étiquetez-les pour éviter la confusion. Si les expériences sont réalisées ‘à l’aveugle’, les boîtes doivent être codées par ex. ‘X’, ‘Y’ ou ‘Test’, en l'absence des expérimentateurs; ne dévoilez la contingence avec le vrai substrat qu’après l'expérience. Stockez les boîtes à température ambiante jusqu'au jour suivant où elles seront utilisées – tenez-les éloignées d'une fenêtre ouverte ou d'un chauffage (condensation; dessèchement)
Boîtes test
Solidifier
C’est prêt!
Fermer, étiqueter/coder
Note:
Pour plusieurs répétitions de l’expérience les boîtes de Pétri peuvent être produites en plus grand nombre et stockées à l’envers au réfrigérateur à 4°C pendant une semaine (pour éviter la formation de gouttes de condensation sur le substrat).

## Slide 6
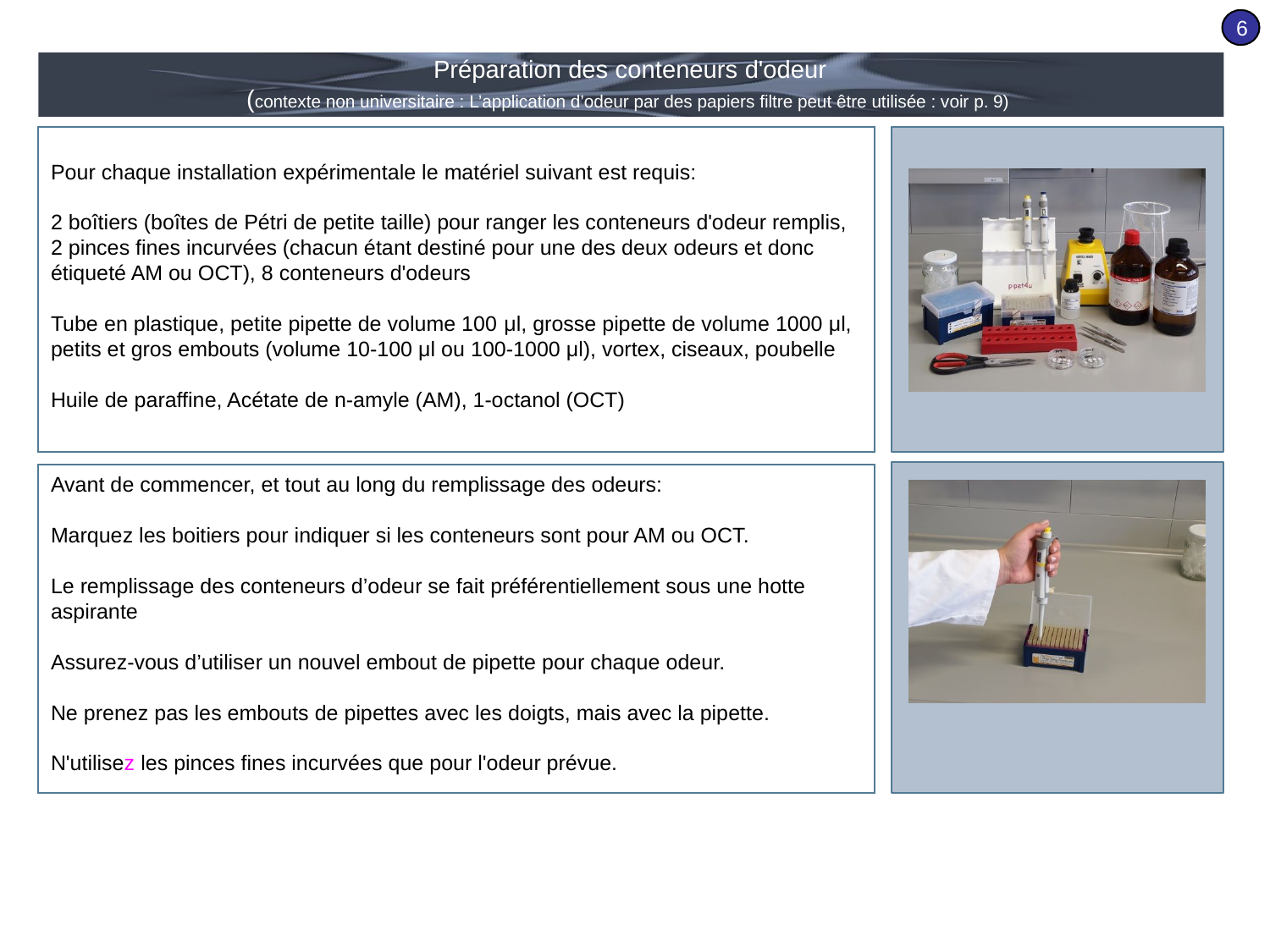

6
# Préparation des conteneurs d'odeur (contexte non universitaire : L’application d’odeur par des papiers filtre peut être utilisée : voir p. 9)
Pour chaque installation expérimentale le matériel suivant est requis:
2 boîtiers (boîtes de Pétri de petite taille) pour ranger les conteneurs d'odeur remplis, 2 pinces fines incurvées (chacun étant destiné pour une des deux odeurs et donc étiqueté AM ou OCT), 8 conteneurs d'odeurs
Tube en plastique, petite pipette de volume 100 μl, grosse pipette de volume 1000 μl, petits et gros embouts (volume 10-100 μl ou 100-1000 μl), vortex, ciseaux, poubelle
 
Huile de paraffine, Acétate de n-amyle (AM), 1-octanol (OCT)
Avant de commencer, et tout au long du remplissage des odeurs:
Marquez les boitiers pour indiquer si les conteneurs sont pour AM ou OCT.
Le remplissage des conteneurs d’odeur se fait préférentiellement sous une hotte aspirante
Assurez-vous d’utiliser un nouvel embout de pipette pour chaque odeur.
Ne prenez pas les embouts de pipettes avec les doigts, mais avec la pipette.
N'utilisez les pinces fines incurvées que pour l'odeur prévue.

## Slide 7
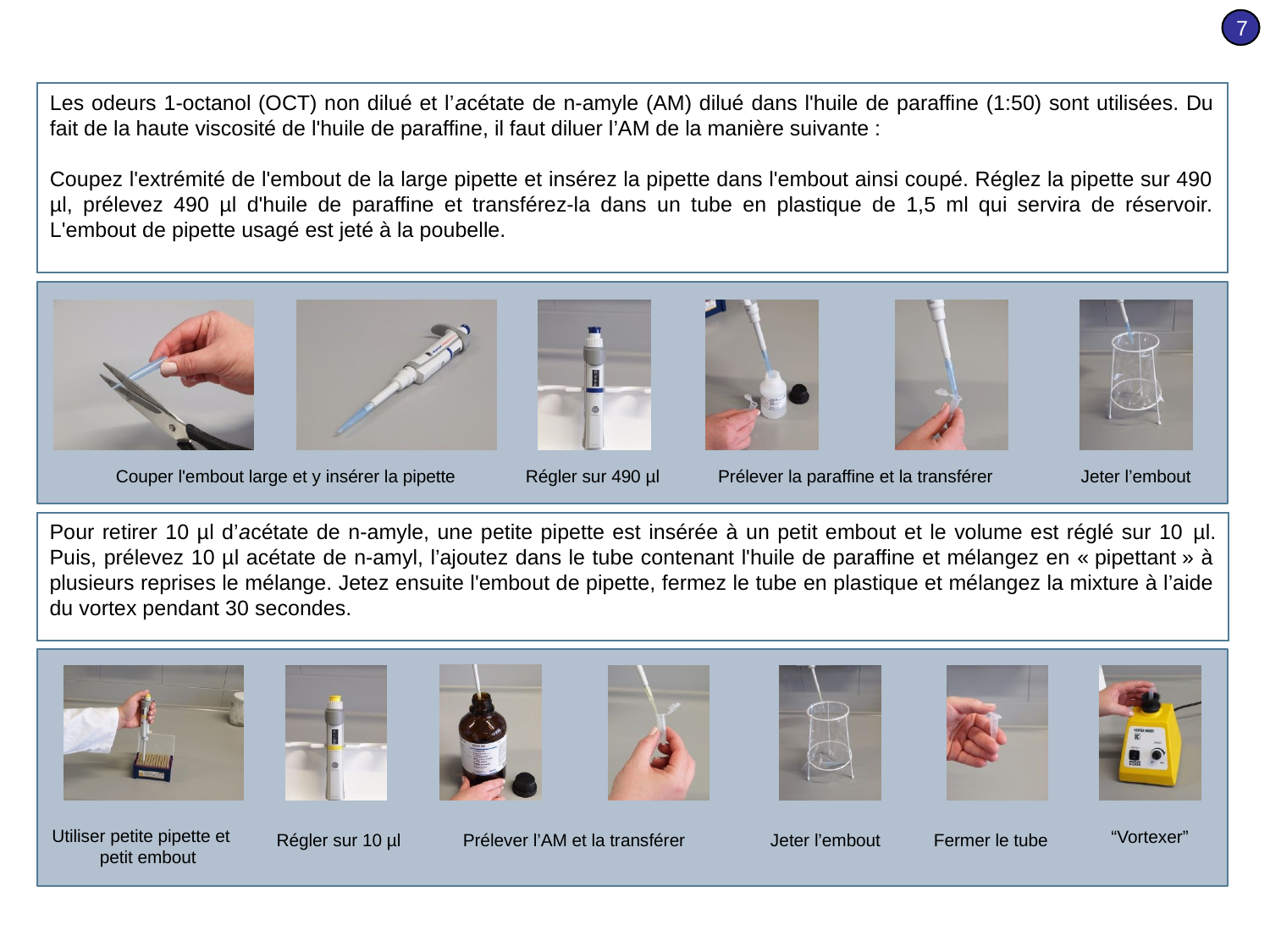

7
Les odeurs 1-octanol (OCT) non dilué et l’acétate de n-amyle (AM) dilué dans l'huile de paraffine (1:50) sont utilisées. Du fait de la haute viscosité de l'huile de paraffine, il faut diluer l’AM de la manière suivante :
Coupez l'extrémité de l'embout de la large pipette et insérez la pipette dans l'embout ainsi coupé. Réglez la pipette sur 490 µl, prélevez 490 µl d'huile de paraffine et transférez-la dans un tube en plastique de 1,5 ml qui servira de réservoir. L'embout de pipette usagé est jeté à la poubelle.
Couper l'embout large et y insérer la pipette
Régler sur 490 µl
Prélever la paraffine et la transférer
Jeter l’embout
Pour retirer 10 µl d’acétate de n-amyle, une petite pipette est insérée à un petit embout et le volume est réglé sur 10 µl. Puis, prélevez 10 µl acétate de n-amyl, l’ajoutez dans le tube contenant l'huile de paraffine et mélangez en « pipettant » à plusieurs reprises le mélange. Jetez ensuite l'embout de pipette, fermez le tube en plastique et mélangez la mixture à l’aide du vortex pendant 30 secondes.
Utiliser petite pipette et petit embout
“Vortexer”
Régler sur 10 µl
Prélever l’AM et la transférer
Jeter l’embout
Fermer le tube

## Slide 8
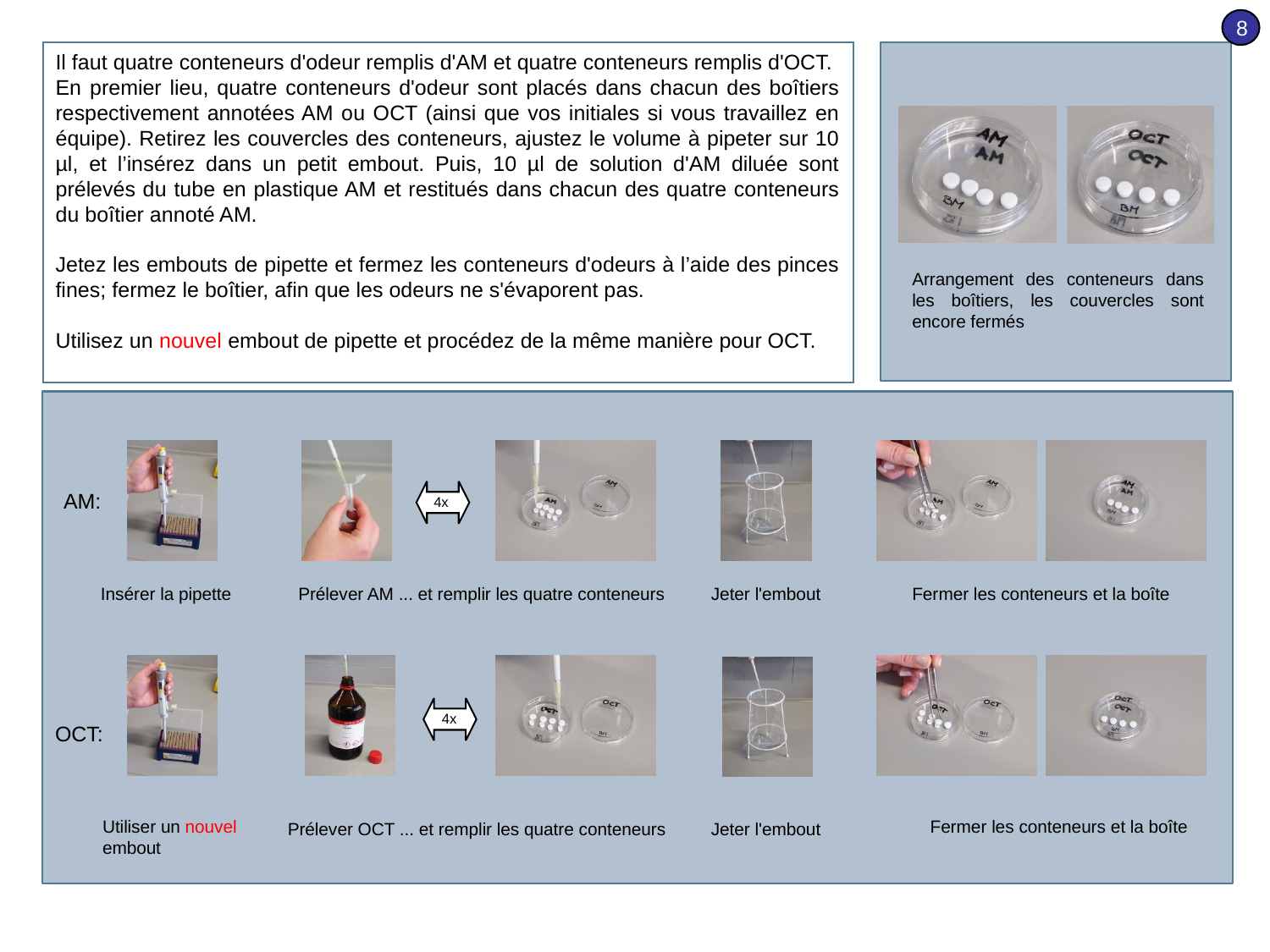

8
Il faut quatre conteneurs d'odeur remplis d'AM et quatre conteneurs remplis d'OCT.
En premier lieu, quatre conteneurs d'odeur sont placés dans chacun des boîtiers respectivement annotées AM ou OCT (ainsi que vos initiales si vous travaillez en équipe). Retirez les couvercles des conteneurs, ajustez le volume à pipeter sur 10 µl, et l’insérez dans un petit embout. Puis, 10 µl de solution d'AM diluée sont prélevés du tube en plastique AM et restitués dans chacun des quatre conteneurs du boîtier annoté AM.
Jetez les embouts de pipette et fermez les conteneurs d'odeurs à l’aide des pinces fines; fermez le boîtier, afin que les odeurs ne s'évaporent pas.
Utilisez un nouvel embout de pipette et procédez de la même manière pour OCT.
Arrangement des conteneurs dans les boîtiers, les couvercles sont encore fermés
4x
AM:
Insérer la pipette
Prélever AM ... et remplir les quatre conteneurs
Jeter l'embout
Fermer les conteneurs et la boîte
4x
OCT:
Utiliser un nouvel embout
Fermer les conteneurs et la boîte
Prélever OCT ... et remplir les quatre conteneurs
Jeter l'embout

## Slide 9
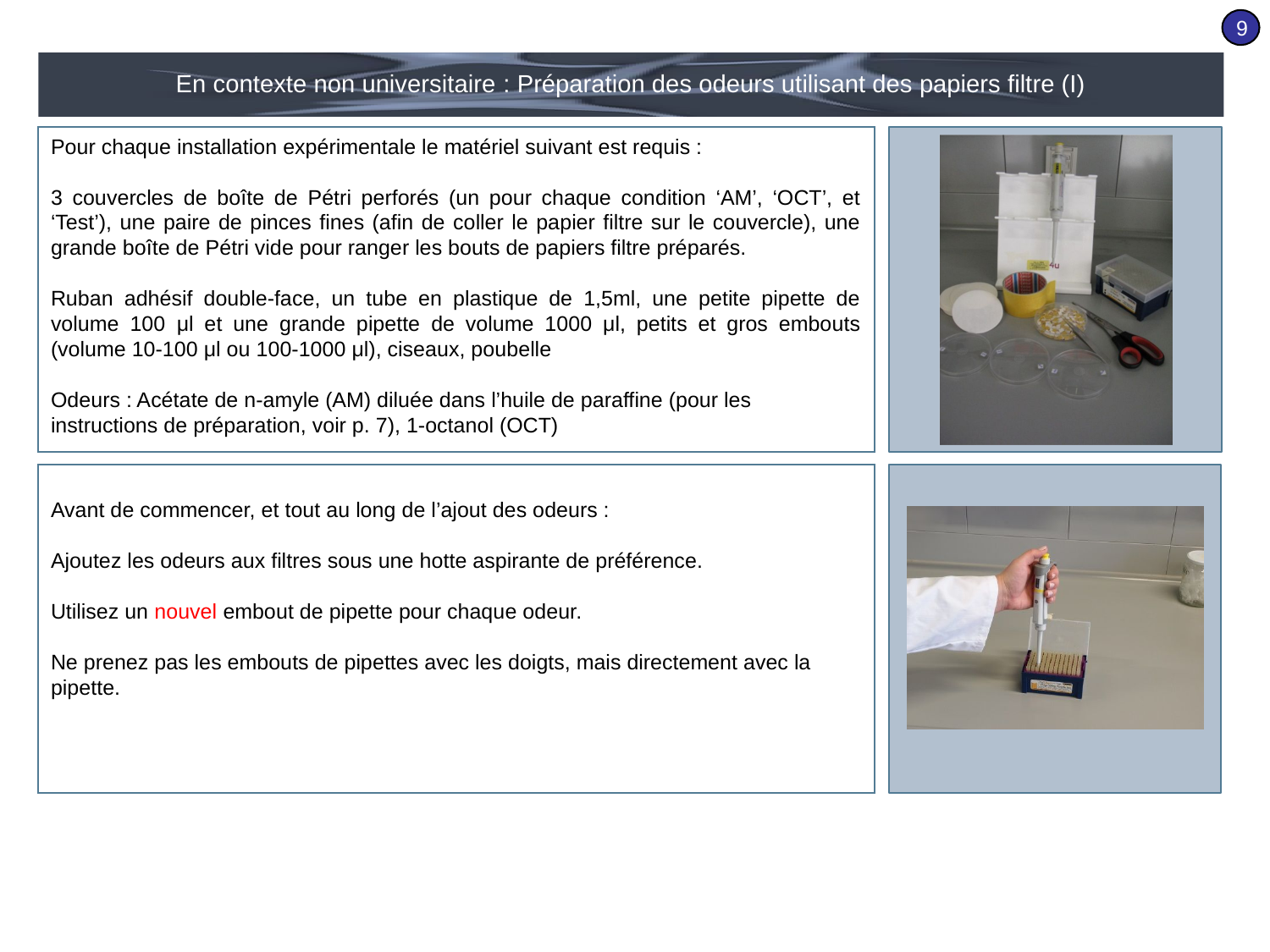

9
# En contexte non universitaire : Préparation des odeurs utilisant des papiers filtre (I)
Pour chaque installation expérimentale le matériel suivant est requis :
3 couvercles de boîte de Pétri perforés (un pour chaque condition ‘AM’, ‘OCT’, et ‘Test’), une paire de pinces fines (afin de coller le papier filtre sur le couvercle), une grande boîte de Pétri vide pour ranger les bouts de papiers filtre préparés.
Ruban adhésif double-face, un tube en plastique de 1,5ml, une petite pipette de volume 100 μl et une grande pipette de volume 1000 μl, petits et gros embouts (volume 10-100 μl ou 100-1000 μl), ciseaux, poubelle
Odeurs : Acétate de n-amyle (AM) diluée dans l’huile de paraffine (pour les instructions de préparation, voir p. 7), 1-octanol (OCT)
Avant de commencer, et tout au long de l’ajout des odeurs :
Ajoutez les odeurs aux filtres sous une hotte aspirante de préférence.
Utilisez un nouvel embout de pipette pour chaque odeur.
Ne prenez pas les embouts de pipettes avec les doigts, mais directement avec la pipette.

## Slide 10
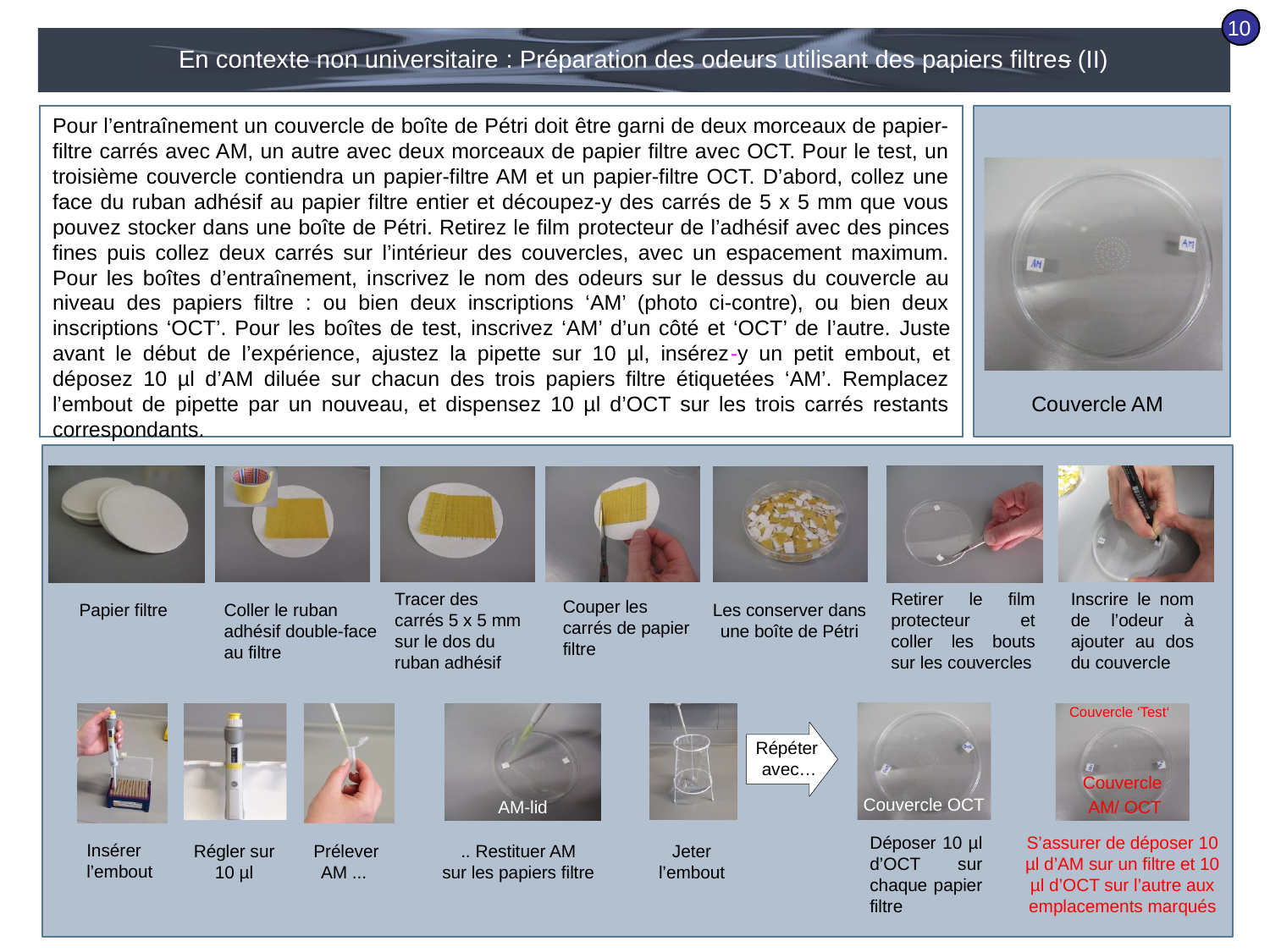

10
# En contexte non universitaire : Préparation des odeurs utilisant des papiers filtres (II)
Pour l’entraînement un couvercle de boîte de Pétri doit être garni de deux morceaux de papier-filtre carrés avec AM, un autre avec deux morceaux de papier filtre avec OCT. Pour le test, un troisième couvercle contiendra un papier-filtre AM et un papier-filtre OCT. D’abord, collez une face du ruban adhésif au papier filtre entier et découpez-y des carrés de 5 x 5 mm que vous pouvez stocker dans une boîte de Pétri. Retirez le film protecteur de l’adhésif avec des pinces fines puis collez deux carrés sur l’intérieur des couvercles, avec un espacement maximum. Pour les boîtes d’entraînement, inscrivez le nom des odeurs sur le dessus du couvercle au niveau des papiers filtre : ou bien deux inscriptions ‘AM’ (photo ci-contre), ou bien deux inscriptions ‘OCT’. Pour les boîtes de test, inscrivez ‘AM’ d’un côté et ‘OCT’ de l’autre. Juste avant le début de l’expérience, ajustez la pipette sur 10 µl, insérez-y un petit embout, et déposez 10 µl d’AM diluée sur chacun des trois papiers filtre étiquetées ‘AM’. Remplacez l’embout de pipette par un nouveau, et dispensez 10 µl d’OCT sur les trois carrés restants correspondants.
Couvercle AM
Tracer des carrés 5 x 5 mm sur le dos du ruban adhésif
Retirer le film protecteur et coller les bouts sur les couvercles
Inscrire le nom de l’odeur à ajouter au dos du couvercle
Couper les carrés de papier filtre
Papier filtre
Coller le ruban adhésif double-face au filtre
Les conserver dans une boîte de Pétri
Couvercle ‘Test‘
Répéter
avec…
Couvercle
AM/ OCT
Couvercle OCT
AM-lid
Déposer 10 µl d’OCT sur chaque papier filtre
S’assurer de déposer 10 µl d’AM sur un filtre et 10 µl d’OCT sur l’autre aux emplacements marqués
Insérer l’embout
Régler sur 10 µl
Prélever AM ...
.. Restituer AM
sur les papiers filtre
Jeter l’embout

## Slide 11
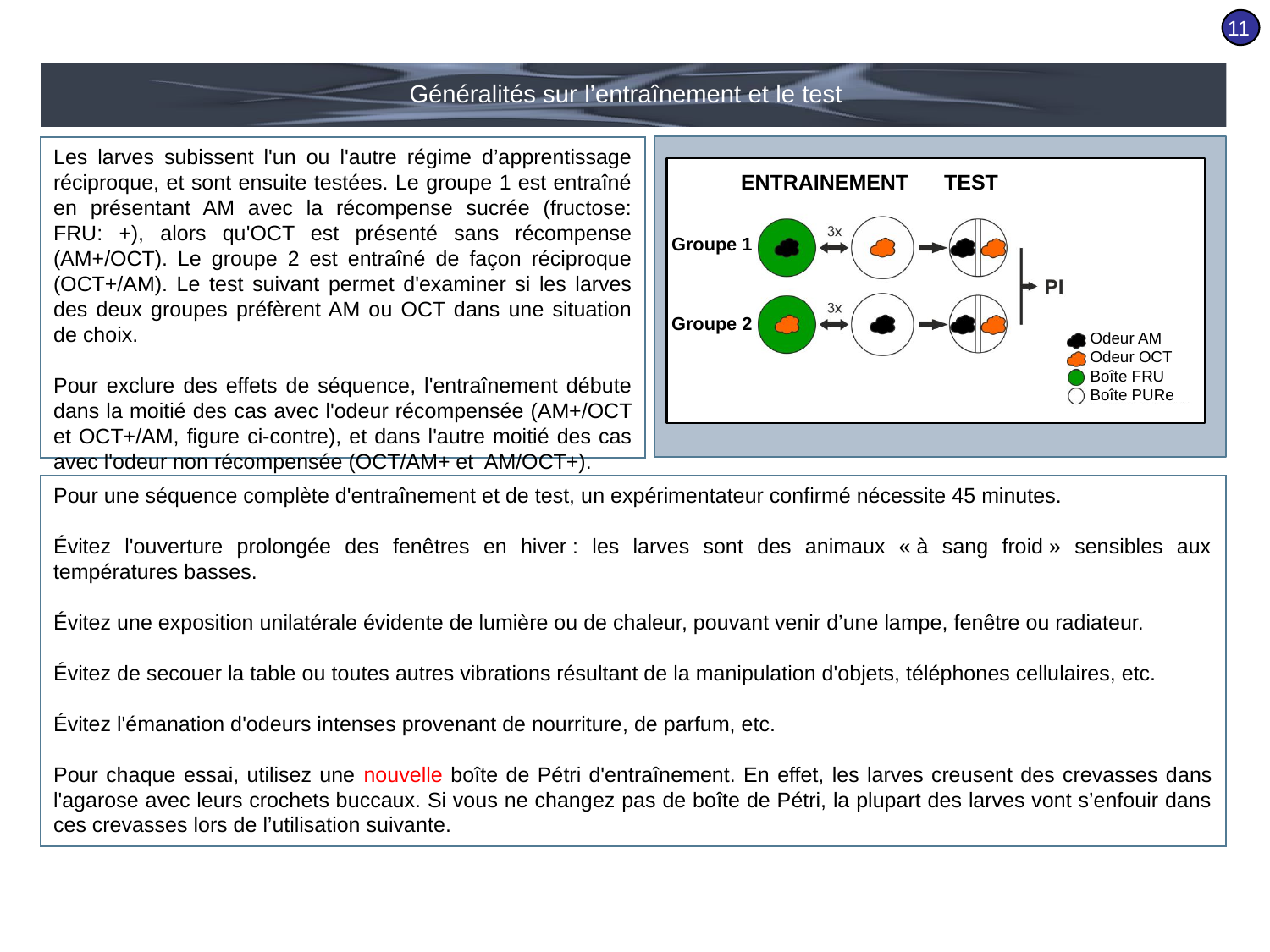

11
Généralités sur l’entraînement et le test
Les larves subissent l'un ou l'autre régime d’apprentissage réciproque, et sont ensuite testées. Le groupe 1 est entraîné en présentant AM avec la récompense sucrée (fructose: FRU: +), alors qu'OCT est présenté sans récompense (AM+/OCT). Le groupe 2 est entraîné de façon réciproque (OCT+/AM). Le test suivant permet d'examiner si les larves des deux groupes préfèrent AM ou OCT dans une situation de choix.
Pour exclure des effets de séquence, l'entraînement débute dans la moitié des cas avec l'odeur récompensée (AM+/OCT et OCT+/AM, figure ci-contre), et dans l'autre moitié des cas avec l'odeur non récompensée (OCT/AM+ et AM/OCT+).
ENTRAINEMENT TEST
Groupe 1
Groupe 2
Odeur AM
Odeur OCT
Boîte FRU
Boîte PURe
Pour une séquence complète d'entraînement et de test, un expérimentateur confirmé nécessite 45 minutes.
Évitez l'ouverture prolongée des fenêtres en hiver : les larves sont des animaux « à sang froid » sensibles aux températures basses.
Évitez une exposition unilatérale évidente de lumière ou de chaleur, pouvant venir d’une lampe, fenêtre ou radiateur.
Évitez de secouer la table ou toutes autres vibrations résultant de la manipulation d'objets, téléphones cellulaires, etc.
Évitez l'émanation d'odeurs intenses provenant de nourriture, de parfum, etc.
Pour chaque essai, utilisez une nouvelle boîte de Pétri d'entraînement. En effet, les larves creusent des crevasses dans l'agarose avec leurs crochets buccaux. Si vous ne changez pas de boîte de Pétri, la plupart des larves vont s’enfouir dans ces crevasses lors de l’utilisation suivante.

## Slide 12
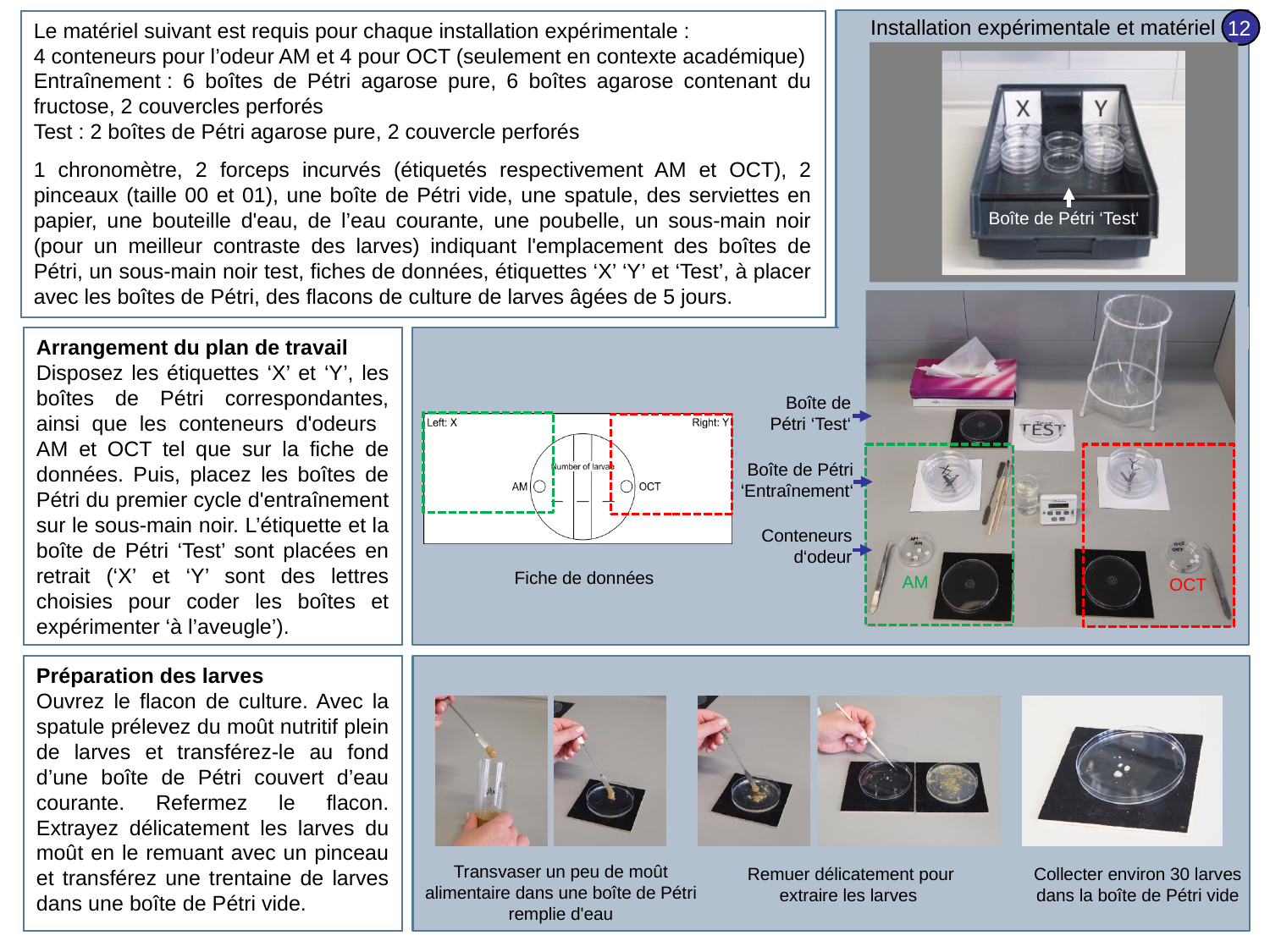

Installation expérimentale et matériel setup with material
12
Le matériel suivant est requis pour chaque installation expérimentale :
4 conteneurs pour l’odeur AM et 4 pour OCT (seulement en contexte académique)
Entraînement : 6 boîtes de Pétri agarose pure, 6 boîtes agarose contenant du fructose, 2 couvercles perforés
Test : 2 boîtes de Pétri agarose pure, 2 couvercle perforés
1 chronomètre, 2 forceps incurvés (étiquetés respectivement AM et OCT), 2 pinceaux (taille 00 et 01), une boîte de Pétri vide, une spatule, des serviettes en papier, une bouteille d'eau, de l’eau courante, une poubelle, un sous-main noir (pour un meilleur contraste des larves) indiquant l'emplacement des boîtes de Pétri, un sous-main noir test, fiches de données, étiquettes ‘X’ ‘Y’ et ‘Test’, à placer avec les boîtes de Pétri, des flacons de culture de larves âgées de 5 jours.
Boîte de Pétri ‘Test‘
Arrangement du plan de travail
Disposez les étiquettes ‘X’ et ‘Y’, les boîtes de Pétri correspondantes, ainsi que les conteneurs d'odeurs AM et OCT tel que sur la fiche de données. Puis, placez les boîtes de Pétri du premier cycle d'entraînement sur le sous-main noir. L’étiquette et la boîte de Pétri ‘Test’ sont placées en retrait (‘X’ et ‘Y’ sont des lettres choisies pour coder les boîtes et expérimenter ‘à l’aveugle’).
Boîte de Pétri ‘Test‘
OCT
Y
Boîte de Pétri ‘Entraînement‘
Conteneurs d‘odeur
Fiche de données
AM
OCT
Préparation des larves
Ouvrez le flacon de culture. Avec la spatule prélevez du moût nutritif plein de larves et transférez-le au fond d’une boîte de Pétri couvert d’eau courante. Refermez le flacon. Extrayez délicatement les larves du moût en le remuant avec un pinceau et transférez une trentaine de larves dans une boîte de Pétri vide.
Transvaser un peu de moût alimentaire dans une boîte de Pétri remplie d'eau
Remuer délicatement pour extraire les larves
Collecter environ 30 larves dans la boîte de Pétri vide

## Slide 13
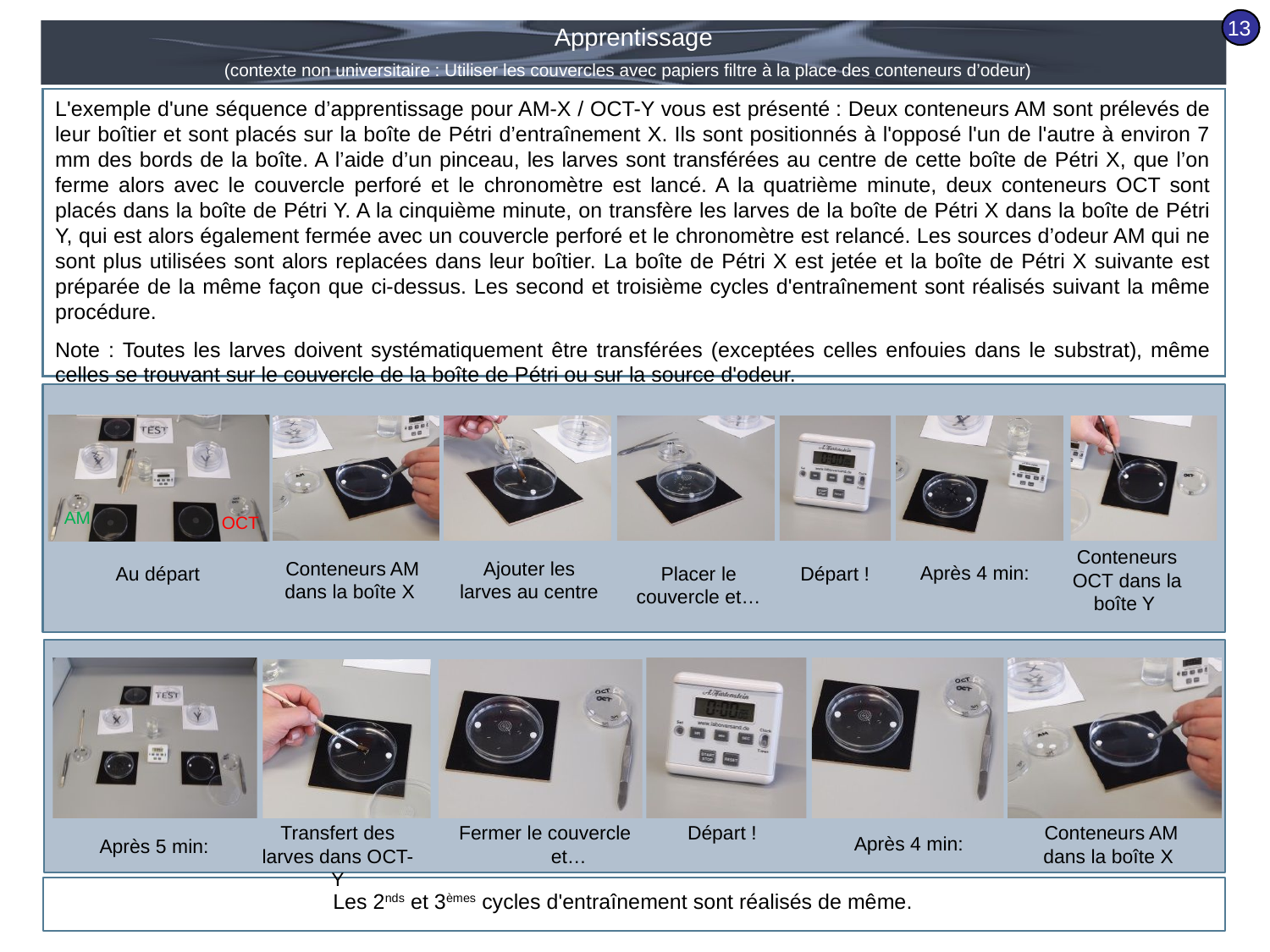

13
# Apprentissage
(contexte non universitaire : Utiliser les couvercles avec papiers filtre à la place des conteneurs d’odeur)
L'exemple d'une séquence d’apprentissage pour AM-X / OCT-Y vous est présenté : Deux conteneurs AM sont prélevés de leur boîtier et sont placés sur la boîte de Pétri d’entraînement X. Ils sont positionnés à l'opposé l'un de l'autre à environ 7 mm des bords de la boîte. A l’aide d’un pinceau, les larves sont transférées au centre de cette boîte de Pétri X, que l’on ferme alors avec le couvercle perforé et le chronomètre est lancé. A la quatrième minute, deux conteneurs OCT sont placés dans la boîte de Pétri Y. A la cinquième minute, on transfère les larves de la boîte de Pétri X dans la boîte de Pétri Y, qui est alors également fermée avec un couvercle perforé et le chronomètre est relancé. Les sources d’odeur AM qui ne sont plus utilisées sont alors replacées dans leur boîtier. La boîte de Pétri X est jetée et la boîte de Pétri X suivante est préparée de la même façon que ci-dessus. Les second et troisième cycles d'entraînement sont réalisés suivant la même procédure.
Note : Toutes les larves doivent systématiquement être transférées (exceptées celles enfouies dans le substrat), même celles se trouvant sur le couvercle de la boîte de Pétri ou sur la source d'odeur.
AM
OCT
Conteneurs OCT dans la boîte Y
Conteneurs AM dans la boîte X
Ajouter les larves au centre
Après 4 min:
Placer le couvercle et…
Au départ
Départ !
Transfert des larves dans OCT-Y
Fermer le couvercle et…
Départ !
Conteneurs AM dans la boîte X
Après 4 min:
Après 5 min:
Les 2nds et 3èmes cycles d'entraînement sont réalisés de même.

## Slide 14
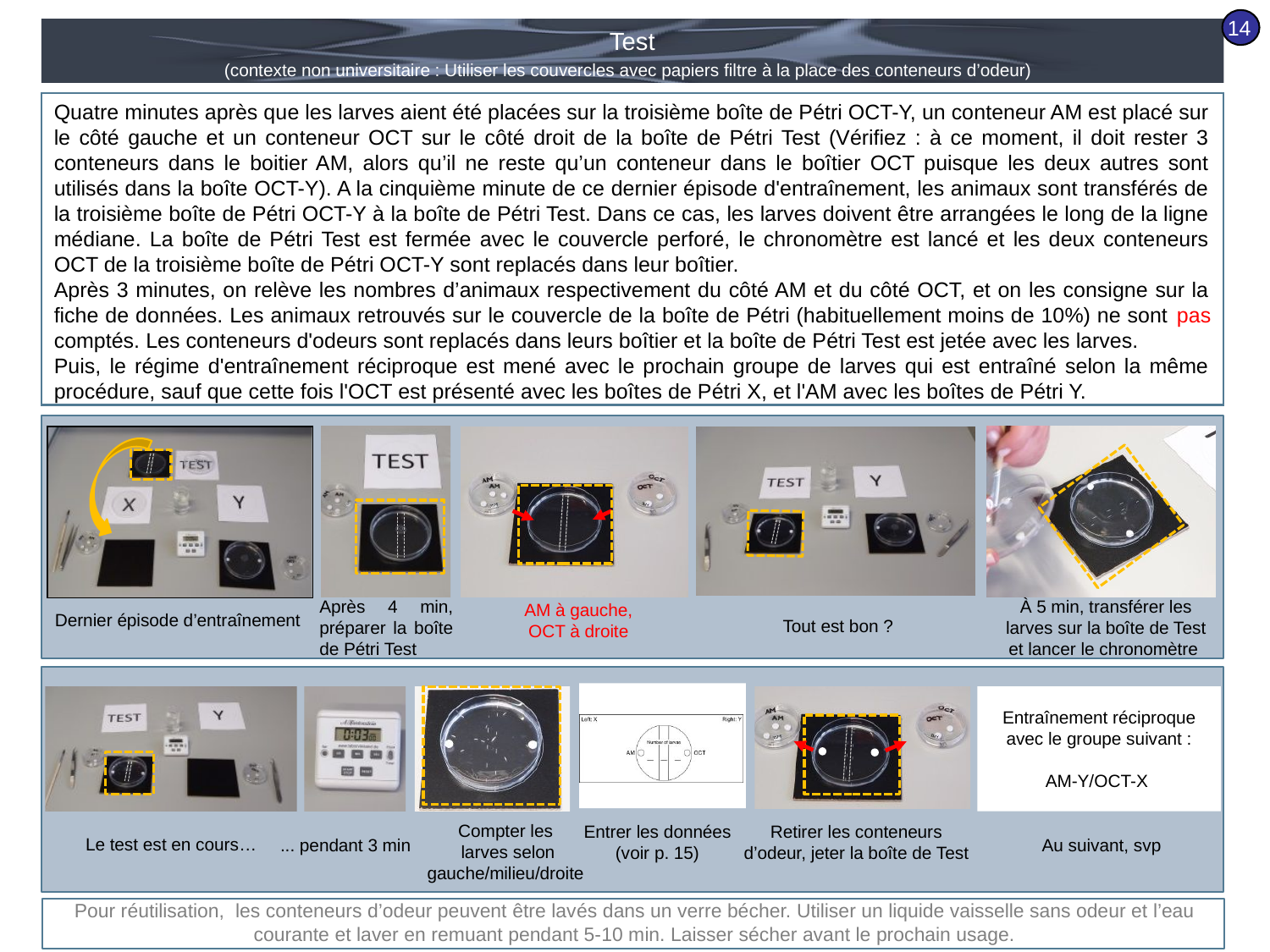

# Test
14
(contexte non universitaire : Utiliser les couvercles avec papiers filtre à la place des conteneurs d’odeur)
Quatre minutes après que les larves aient été placées sur la troisième boîte de Pétri OCT-Y, un conteneur AM est placé sur le côté gauche et un conteneur OCT sur le côté droit de la boîte de Pétri Test (Vérifiez : à ce moment, il doit rester 3 conteneurs dans le boitier AM, alors qu’il ne reste qu’un conteneur dans le boîtier OCT puisque les deux autres sont utilisés dans la boîte OCT-Y). A la cinquième minute de ce dernier épisode d'entraînement, les animaux sont transférés de la troisième boîte de Pétri OCT-Y à la boîte de Pétri Test. Dans ce cas, les larves doivent être arrangées le long de la ligne médiane. La boîte de Pétri Test est fermée avec le couvercle perforé, le chronomètre est lancé et les deux conteneurs OCT de la troisième boîte de Pétri OCT-Y sont replacés dans leur boîtier.
Après 3 minutes, on relève les nombres d’animaux respectivement du côté AM et du côté OCT, et on les consigne sur la fiche de données. Les animaux retrouvés sur le couvercle de la boîte de Pétri (habituellement moins de 10%) ne sont pas comptés. Les conteneurs d'odeurs sont replacés dans leurs boîtier et la boîte de Pétri Test est jetée avec les larves.
Puis, le régime d'entraînement réciproque est mené avec le prochain groupe de larves qui est entraîné selon la même procédure, sauf que cette fois l'OCT est présenté avec les boîtes de Pétri X, et l'AM avec les boîtes de Pétri Y.
À 5 min, transférer les larves sur la boîte de Test et lancer le chronomètre
Après 4 min, préparer la boîte de Pétri Test
AM à gauche, OCT à droite
Dernier épisode d’entraînement
Tout est bon ?
Entraînement réciproque avec le groupe suivant :
AM-Y/OCT-X
Compter les
larves selon
gauche/milieu/droite
Entrer les données
(voir p. 15)
Retirer les conteneurs d’odeur, jeter la boîte de Test
Le test est en cours…
Au suivant, svp
... pendant 3 min
Pour réutilisation, les conteneurs d’odeur peuvent être lavés dans un verre bécher. Utiliser un liquide vaisselle sans odeur et l’eau courante et laver en remuant pendant 5-10 min. Laisser sécher avant le prochain usage.

## Slide 15
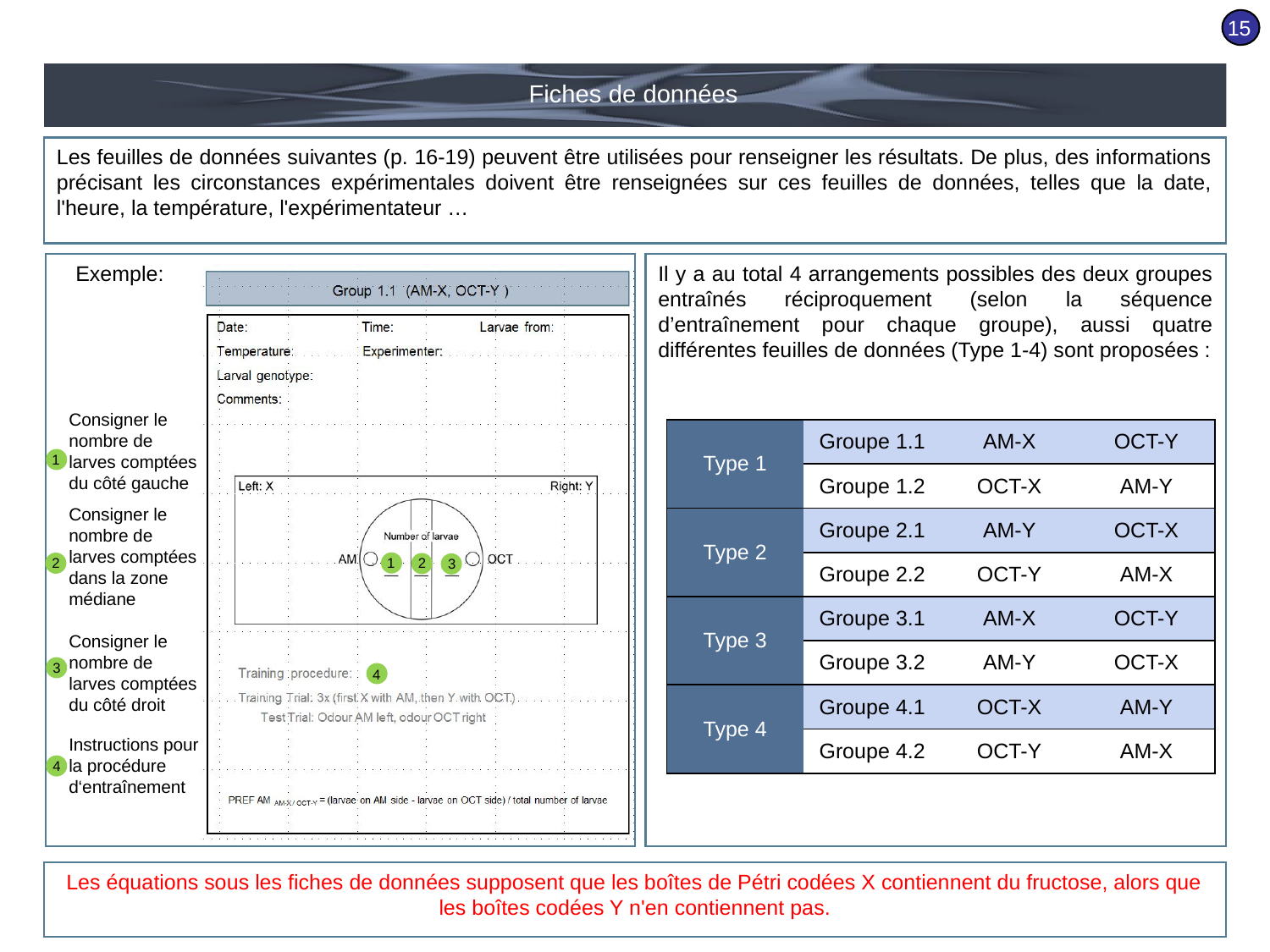

15
Fiches de données
Les feuilles de données suivantes (p. 16-19) peuvent être utilisées pour renseigner les résultats. De plus, des informations précisant les circonstances expérimentales doivent être renseignées sur ces feuilles de données, telles que la date, l'heure, la température, l'expérimentateur …
 Exemple:
Il y a au total 4 arrangements possibles des deux groupes entraînés réciproquement (selon la séquence d’entraînement pour chaque groupe), aussi quatre différentes feuilles de données (Type 1-4) sont proposées :
Consigner le nombre de larves comptées du côté gauche
| Type 1 | Groupe 1.1 | AM-X | OCT-Y |
| --- | --- | --- | --- |
| | Groupe 1.2 | OCT-X | AM-Y |
| Type 2 | Groupe 2.1 | AM-Y | OCT-X |
| | Groupe 2.2 | OCT-Y | AM-X |
| Type 3 | Groupe 3.1 | AM-X | OCT-Y |
| | Groupe 3.2 | AM-Y | OCT-X |
| Type 4 | Groupe 4.1 | OCT-X | AM-Y |
| | Groupe 4.2 | OCT-Y | AM-X |
1
Consigner le nombre de larves comptées dans la zone médiane
1
2
2
3
Consigner le nombre de larves comptées du côté droit
3
4
Instructions pour la procédure d‘entraînement
4
Les équations sous les fiches de données supposent que les boîtes de Pétri codées X contiennent du fructose, alors que les boîtes codées Y n'en contiennent pas.

## Slide 16
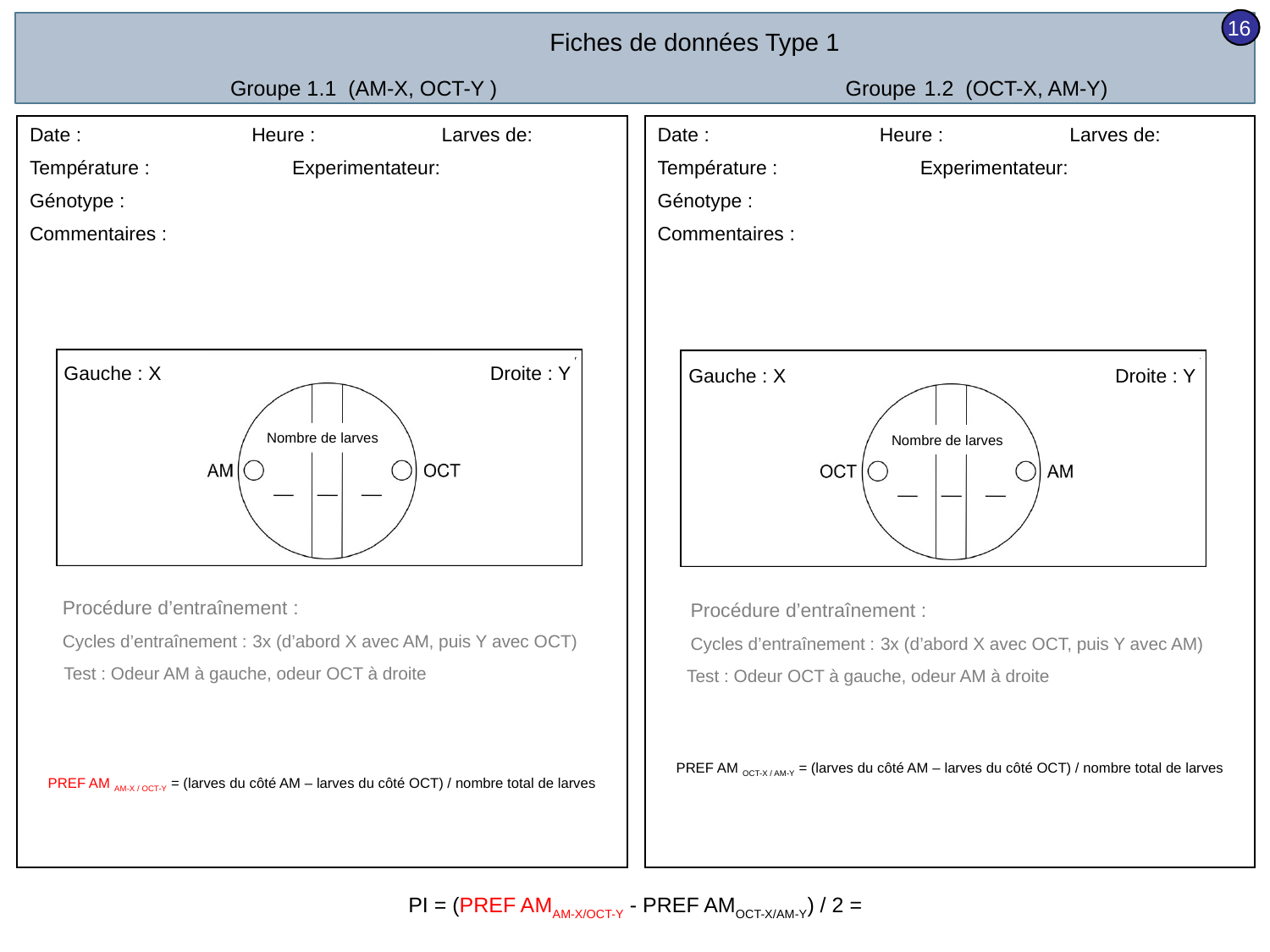

16
# Fiches de données Type 1 Groupe 1.1 (AM-X, OCT-Y ) Groupe 1.2 (OCT-X, AM-Y)
Date : Heure : Larves de:
Température : 	 Experimentateur:
Génotype :
Commentaires :
 Procédure d’entraînement :
 Cycles d’entraînement : 3x (d’abord X avec AM, puis Y avec OCT)
 Test : Odeur AM à gauche, odeur OCT à droite
PREF AM AM-X / OCT-Y = (larves du côté AM – larves du côté OCT) / nombre total de larves
Date : Heure : Larves de:
Température : 	 Experimentateur:
Génotype :
Commentaires :
 Procédure d’entraînement :
 Cycles d’entraînement : 3x (d’abord X avec OCT, puis Y avec AM)
 Test : Odeur OCT à gauche, odeur AM à droite
PREF AM OCT-X / AM-Y = (larves du côté AM – larves du côté OCT) / nombre total de larves
Gauche : X
Droite : Y
Gauche : X
Droite : Y
Nombre de larves
Nombre de larves
PI = (PREF AMAM-X/OCT-Y - PREF AMOCT-X/AM-Y) / 2 =

## Slide 17
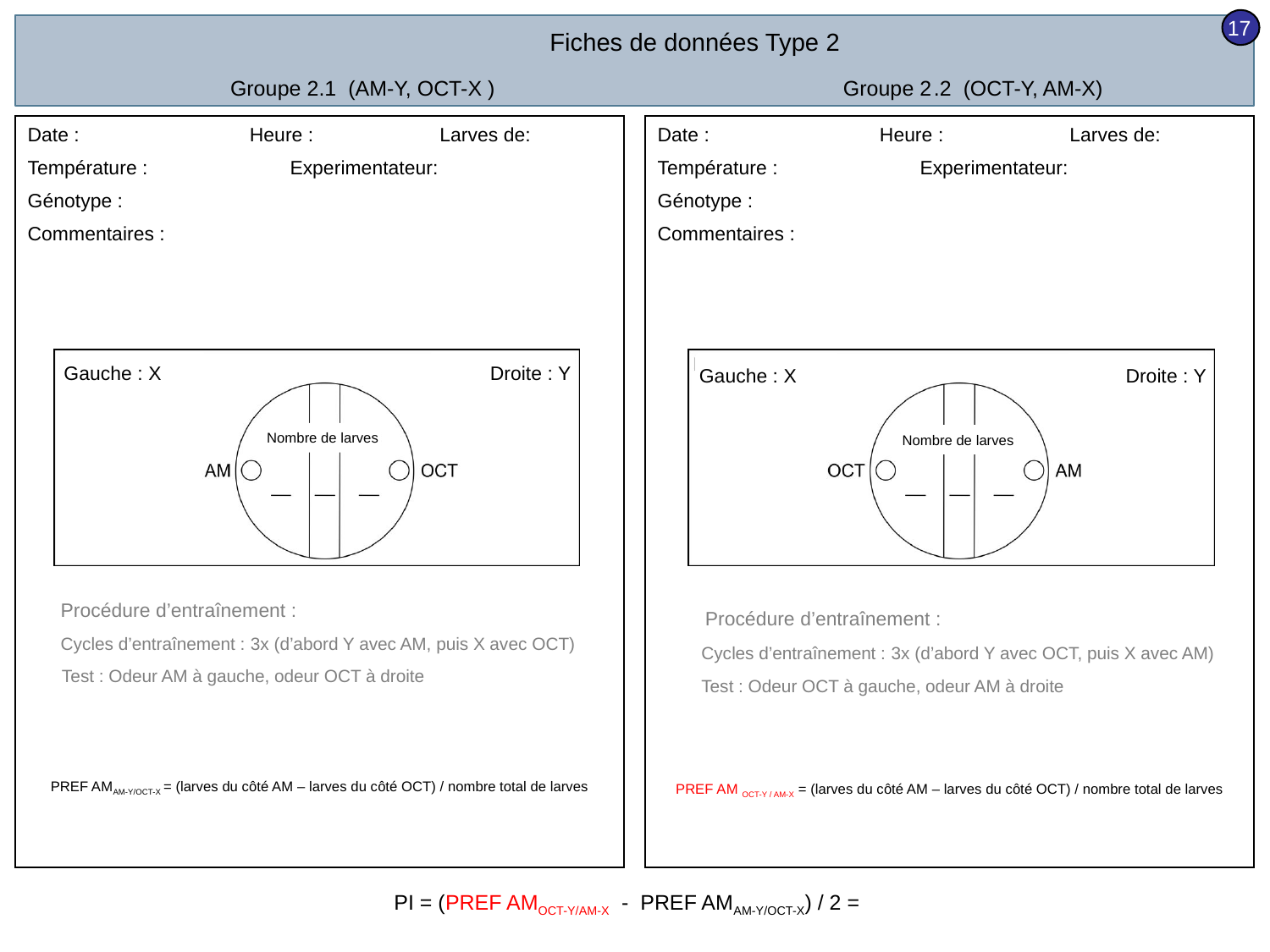

17
				 Fiches de données Type 2
 Groupe 2.1 (AM-Y, OCT-X ) Groupe 2.2 (OCT-Y, AM-X)
Date : Heure : Larves de:
Température : 	 Experimentateur:
Génotype :
Commentaires :
 Procédure d’entraînement :
 Cycles d’entraînement : 3x (d’abord Y avec AM, puis X avec OCT)
 Test : Odeur AM à gauche, odeur OCT à droite
PREF AMAM-Y/OCT-X = (larves du côté AM – larves du côté OCT) / nombre total de larves
Date : Heure : Larves de:
Température : 	 Experimentateur:
Génotype :
Commentaires :
 	Procédure d’entraînement :
 Cycles d’entraînement : 3x (d’abord Y avec OCT, puis X avec AM)
 Test : Odeur OCT à gauche, odeur AM à droite
PREF AM OCT-Y / AM-X = (larves du côté AM – larves du côté OCT) / nombre total de larves
Gauche : X
Droite : Y
Gauche : X
Droite : Y
Nombre de larves
Nombre de larves
PI = (PREF AMOCT-Y/AM-X - PREF AMAM-Y/OCT-X) / 2 =

## Slide 18
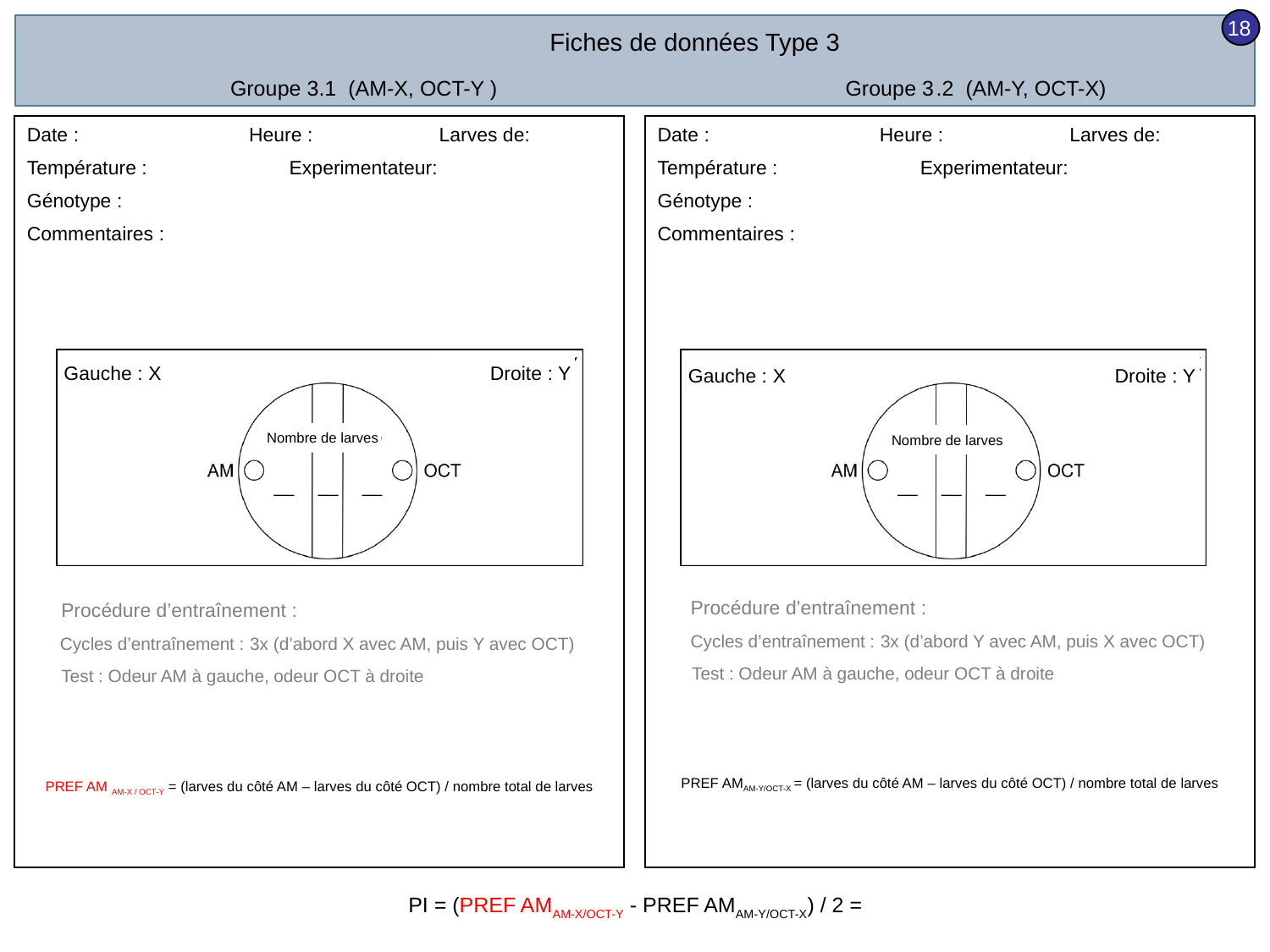

18
				 Fiches de données Type 3
 Groupe 3.1 (AM-X, OCT-Y ) Groupe 3.2 (AM-Y, OCT-X)
Date : Heure : Larves de:
Température : 	 Experimentateur:
Génotype :
Commentaires :
 Procédure d’entraînement :
 Cycles d’entraînement : 3x (d’abord X avec AM, puis Y avec OCT)
 Test : Odeur AM à gauche, odeur OCT à droite
PREF AM AM-X / OCT-Y = (larves du côté AM – larves du côté OCT) / nombre total de larves
Date : Heure : Larves de:
Température : 	 Experimentateur:
Génotype :
Commentaires :
 Procédure d’entraînement :
 Cycles d’entraînement : 3x (d’abord Y avec AM, puis X avec OCT)
 Test : Odeur AM à gauche, odeur OCT à droite
PREF AMAM-Y/OCT-X = (larves du côté AM – larves du côté OCT) / nombre total de larves
Gauche : X
Droite : Y
Gauche : X
Droite : Y
Nombre de larves
Nombre de larves
PI = (PREF AMAM-X/OCT-Y - PREF AMAM-Y/OCT-X) / 2 =

## Slide 19
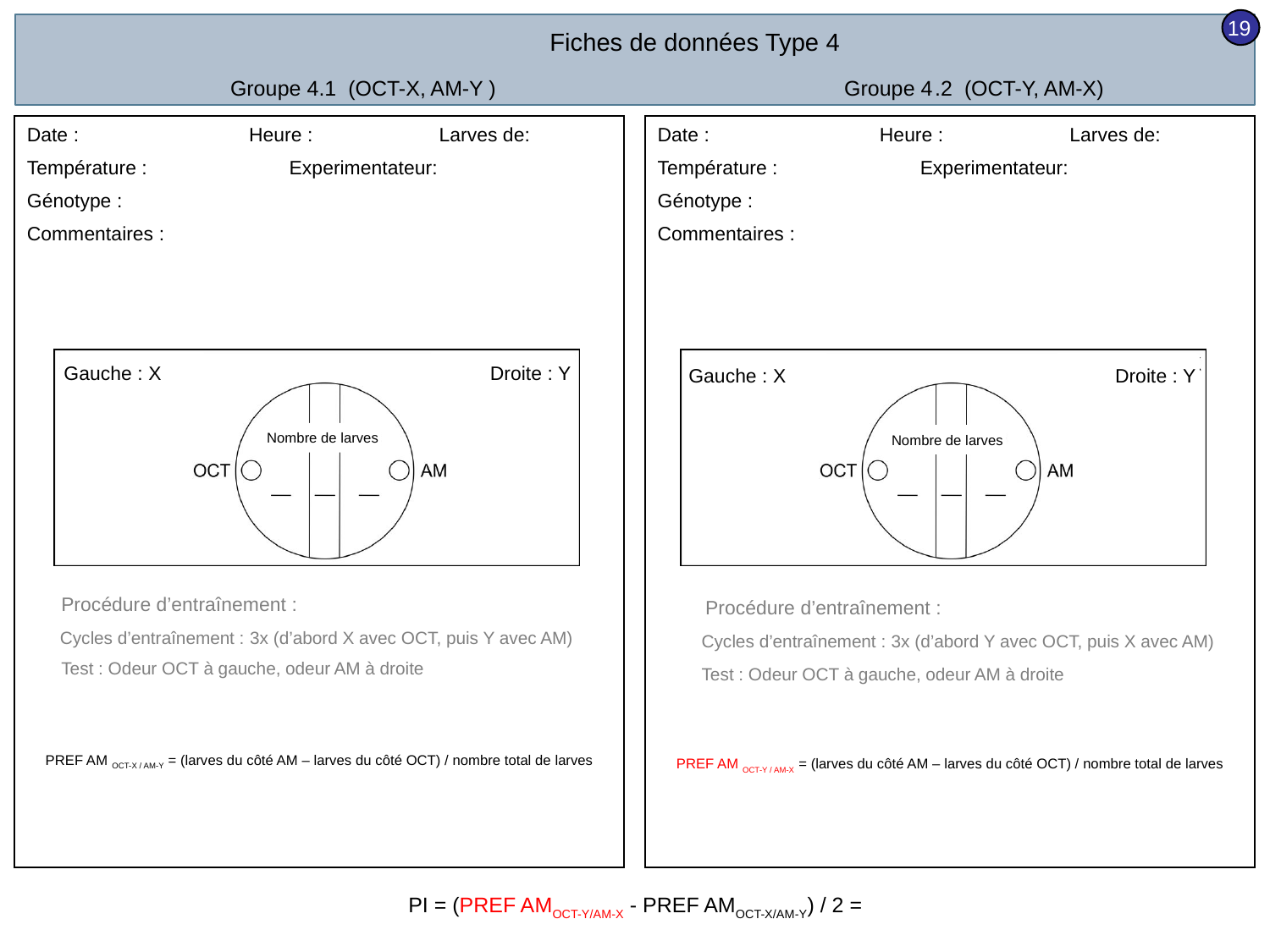

19
				 Fiches de données Type 4
 Groupe 4.1 (OCT-X, AM-Y ) Groupe 4.2 (OCT-Y, AM-X)
Date : Heure : Larves de:
Température : 	 Experimentateur:
Génotype :
Commentaires :
 Procédure d’entraînement :
 Cycles d’entraînement : 3x (d’abord X avec OCT, puis Y avec AM)
 Test : Odeur OCT à gauche, odeur AM à droite
PREF AM OCT-X / AM-Y = (larves du côté AM – larves du côté OCT) / nombre total de larves
Date : Heure : Larves de:
Température : 	 Experimentateur:
Génotype :
Commentaires :
 	Procédure d’entraînement :
 Cycles d’entraînement : 3x (d’abord Y avec OCT, puis X avec AM)
 Test : Odeur OCT à gauche, odeur AM à droite
PREF AM OCT-Y / AM-X = (larves du côté AM – larves du côté OCT) / nombre total de larves
Gauche : X
Droite : Y
Gauche : X
Droite : Y
Nombre de larves
Nombre de larves
PI = (PREF AMOCT-Y/AM-X - PREF AMOCT-X/AM-Y) / 2 =

## Slide 20
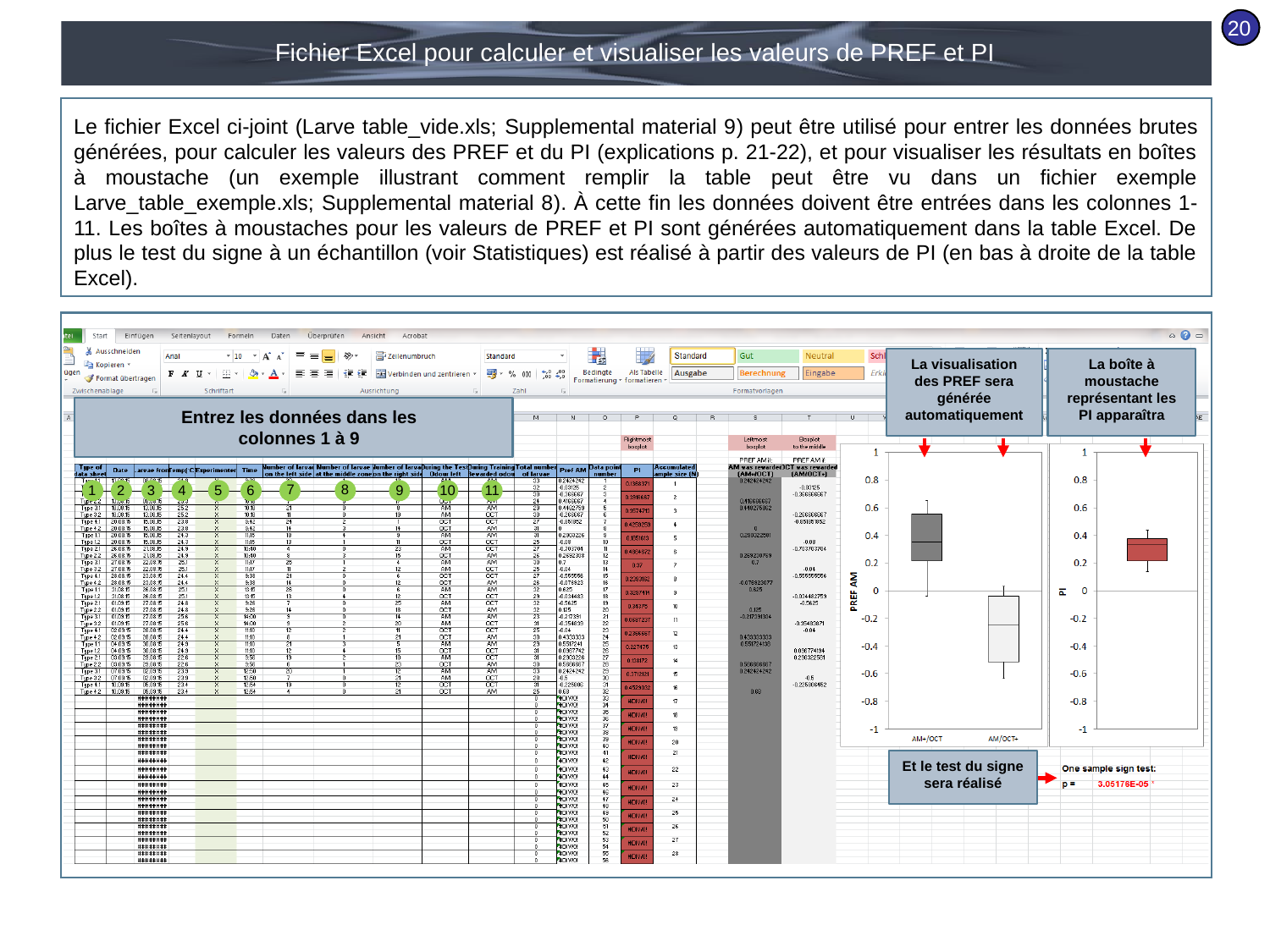

20
Fichier Excel pour calculer et visualiser les valeurs de PREF et PI
Le fichier Excel ci-joint (Larve table_vide.xls; Supplemental material 9) peut être utilisé pour entrer les données brutes générées, pour calculer les valeurs des PREF et du PI (explications p. 21-22), et pour visualiser les résultats en boîtes à moustache (un exemple illustrant comment remplir la table peut être vu dans un fichier exemple Larve_table_exemple.xls; Supplemental material 8). À cette fin les données doivent être entrées dans les colonnes 1-11. Les boîtes à moustaches pour les valeurs de PREF et PI sont générées automatiquement dans la table Excel. De plus le test du signe à un échantillon (voir Statistiques) est réalisé à partir des valeurs de PI (en bas à droite de la table Excel).
La boîte à moustache représentant les PI apparaîtra
La visualisation des PREF sera générée automatiquement
Entrez les données dans les colonnes 1 à 9
7
8
3
4
2
5
6
9
1
10
11
Et le test du signe sera réalisé

## Slide 21
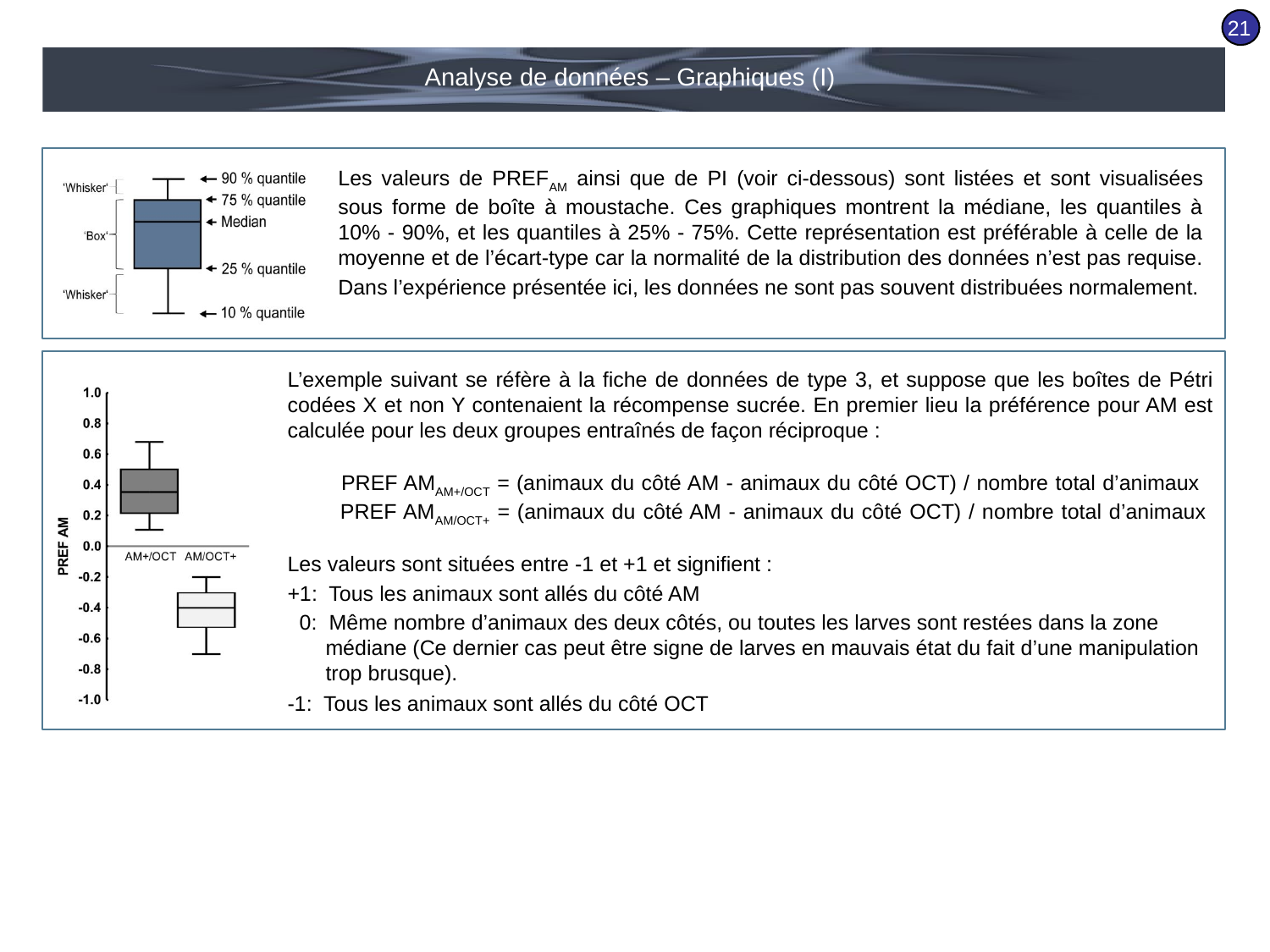

21
Analyse de données – Graphiques (I)
Les valeurs de PREFAM ainsi que de PI (voir ci-dessous) sont listées et sont visualisées sous forme de boîte à moustache. Ces graphiques montrent la médiane, les quantiles à 10% - 90%, et les quantiles à 25% - 75%. Cette représentation est préférable à celle de la moyenne et de l’écart-type car la normalité de la distribution des données n’est pas requise.
Dans l’expérience présentée ici, les données ne sont pas souvent distribuées normalement.
L’exemple suivant se réfère à la fiche de données de type 3, et suppose que les boîtes de Pétri codées X et non Y contenaient la récompense sucrée. En premier lieu la préférence pour AM est calculée pour les deux groupes entraînés de façon réciproque :
 PREF AMAM+/OCT = (animaux du côté AM - animaux du côté OCT) / nombre total d’animaux
 PREF AMAM/OCT+ = (animaux du côté AM - animaux du côté OCT) / nombre total d’animaux
Les valeurs sont situées entre -1 et +1 et signifient :
+1: Tous les animaux sont allés du côté AM
 0: Même nombre d’animaux des deux côtés, ou toutes les larves sont restées dans la zone médiane (Ce dernier cas peut être signe de larves en mauvais état du fait d’une manipulation trop brusque).
-1: Tous les animaux sont allés du côté OCT

## Slide 22
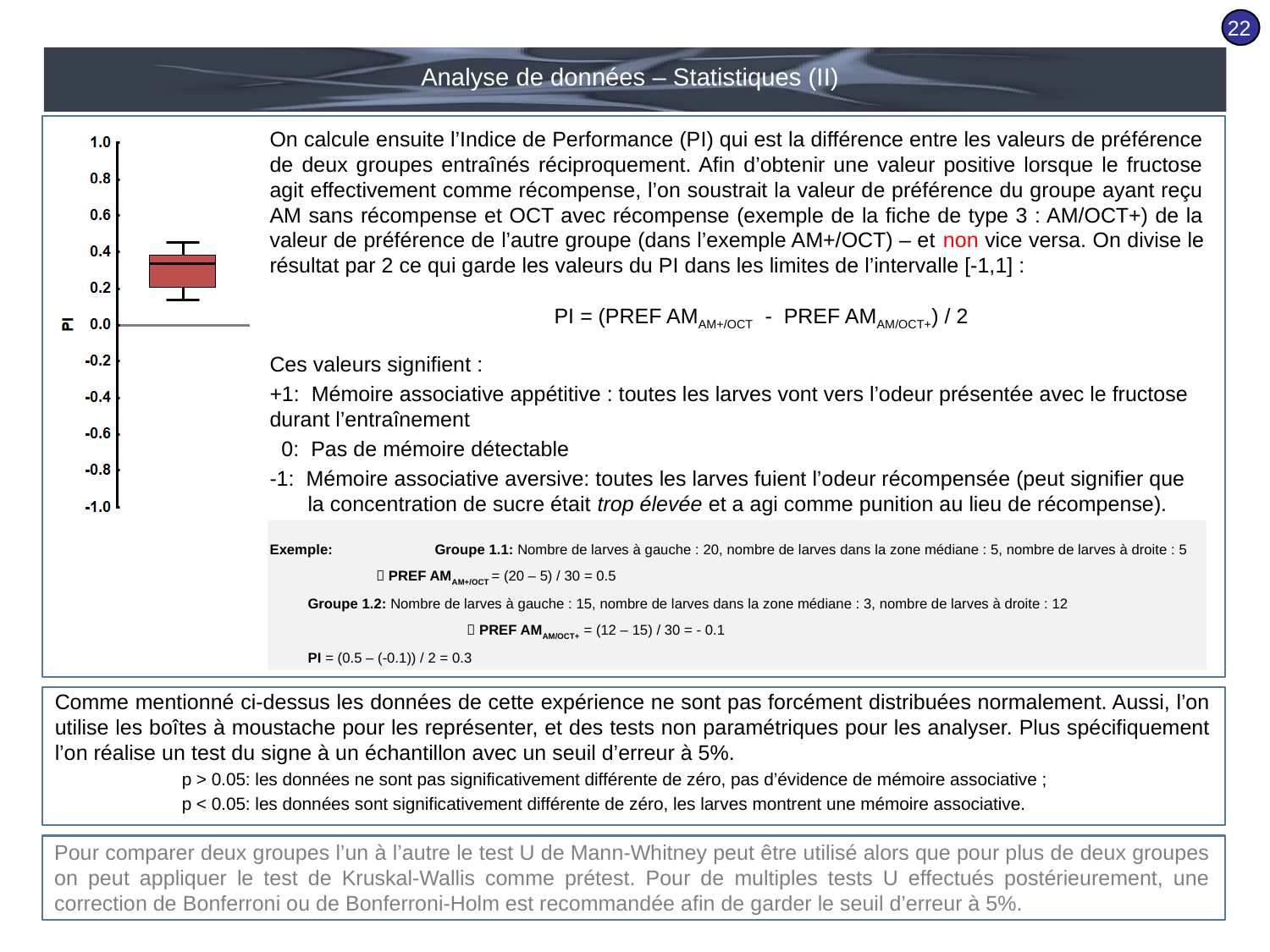

22
Analyse de données – Statistiques (II)
On calcule ensuite l’Indice de Performance (PI) qui est la différence entre les valeurs de préférence de deux groupes entraînés réciproquement. Afin d’obtenir une valeur positive lorsque le fructose agit effectivement comme récompense, l’on soustrait la valeur de préférence du groupe ayant reçu AM sans récompense et OCT avec récompense (exemple de la fiche de type 3 : AM/OCT+) de la valeur de préférence de l’autre groupe (dans l’exemple AM+/OCT) – et non vice versa. On divise le résultat par 2 ce qui garde les valeurs du PI dans les limites de l’intervalle [-1,1] :
 PI = (PREF AMAM+/OCT - PREF AMAM/OCT+) / 2
Ces valeurs signifient :
+1: Mémoire associative appétitive : toutes les larves vont vers l’odeur présentée avec le fructose durant l’entraînement
 0: Pas de mémoire détectable
-1: Mémoire associative aversive: toutes les larves fuient l’odeur récompensée (peut signifier que la concentration de sucre était trop élevée et a agi comme punition au lieu de récompense).
Exemple: 	Groupe 1.1: Nombre de larves à gauche : 20, nombre de larves dans la zone médiane : 5, nombre de larves à droite : 5
	  PREF AMAM+/OCT = (20 – 5) / 30 = 0.5
	Groupe 1.2: Nombre de larves à gauche : 15, nombre de larves dans la zone médiane : 3, nombre de larves à droite : 12
  PREF AMAM/OCT+ = (12 – 15) / 30 = - 0.1
	PI = (0.5 – (-0.1)) / 2 = 0.3
Comme mentionné ci-dessus les données de cette expérience ne sont pas forcément distribuées normalement. Aussi, l’on utilise les boîtes à moustache pour les représenter, et des tests non paramétriques pour les analyser. Plus spécifiquement l’on réalise un test du signe à un échantillon avec un seuil d’erreur à 5%.
	p > 0.05: les données ne sont pas significativement différente de zéro, pas d’évidence de mémoire associative ;
	p < 0.05: les données sont significativement différente de zéro, les larves montrent une mémoire associative.
Pour comparer deux groupes l’un à l’autre le test U de Mann-Whitney peut être utilisé alors que pour plus de deux groupes on peut appliquer le test de Kruskal-Wallis comme prétest. Pour de multiples tests U effectués postérieurement, une correction de Bonferroni ou de Bonferroni-Holm est recommandée afin de garder le seuil d’erreur à 5%.

## Slide 23
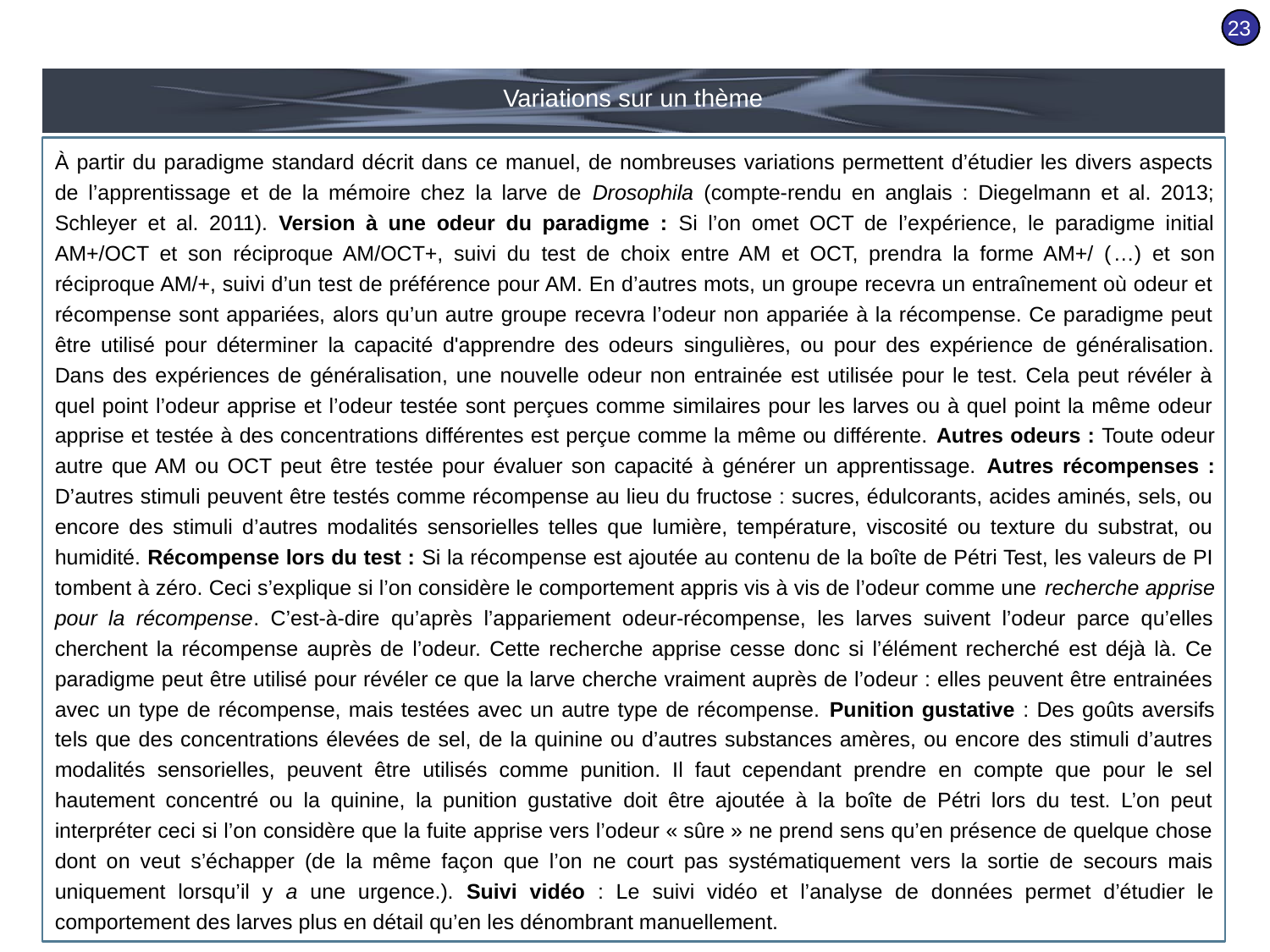

23
Variations sur un thème
À partir du paradigme standard décrit dans ce manuel, de nombreuses variations permettent d’étudier les divers aspects de l’apprentissage et de la mémoire chez la larve de Drosophila (compte-rendu en anglais : Diegelmann et al. 2013; Schleyer et al. 2011). Version à une odeur du paradigme : Si l’on omet OCT de l’expérience, le paradigme initial AM+/OCT et son réciproque AM/OCT+, suivi du test de choix entre AM et OCT, prendra la forme AM+/ (…) et son réciproque AM/+, suivi d’un test de préférence pour AM. En d’autres mots, un groupe recevra un entraînement où odeur et récompense sont appariées, alors qu’un autre groupe recevra l’odeur non appariée à la récompense. Ce paradigme peut être utilisé pour déterminer la capacité d'apprendre des odeurs singulières, ou pour des expérience de généralisation. Dans des expériences de généralisation, une nouvelle odeur non entrainée est utilisée pour le test. Cela peut révéler à quel point l’odeur apprise et l’odeur testée sont perçues comme similaires pour les larves ou à quel point la même odeur apprise et testée à des concentrations différentes est perçue comme la même ou différente. Autres odeurs : Toute odeur autre que AM ou OCT peut être testée pour évaluer son capacité à générer un apprentissage. Autres récompenses : D’autres stimuli peuvent être testés comme récompense au lieu du fructose : sucres, édulcorants, acides aminés, sels, ou encore des stimuli d’autres modalités sensorielles telles que lumière, température, viscosité ou texture du substrat, ou humidité. Récompense lors du test : Si la récompense est ajoutée au contenu de la boîte de Pétri Test, les valeurs de PI tombent à zéro. Ceci s’explique si l’on considère le comportement appris vis à vis de l’odeur comme une recherche apprise pour la récompense. C’est-à-dire qu’après l’appariement odeur-récompense, les larves suivent l’odeur parce qu’elles cherchent la récompense auprès de l’odeur. Cette recherche apprise cesse donc si l’élément recherché est déjà là. Ce paradigme peut être utilisé pour révéler ce que la larve cherche vraiment auprès de l’odeur : elles peuvent être entrainées avec un type de récompense, mais testées avec un autre type de récompense. Punition gustative : Des goûts aversifs tels que des concentrations élevées de sel, de la quinine ou d’autres substances amères, ou encore des stimuli d’autres modalités sensorielles, peuvent être utilisés comme punition. Il faut cependant prendre en compte que pour le sel hautement concentré ou la quinine, la punition gustative doit être ajoutée à la boîte de Pétri lors du test. L’on peut interpréter ceci si l’on considère que la fuite apprise vers l’odeur « sûre » ne prend sens qu’en présence de quelque chose dont on veut s’échapper (de la même façon que l’on ne court pas systématiquement vers la sortie de secours mais uniquement lorsqu’il y a une urgence.). Suivi vidéo : Le suivi vidéo et l’analyse de données permet d’étudier le comportement des larves plus en détail qu’en les dénombrant manuellement.

## Slide 24
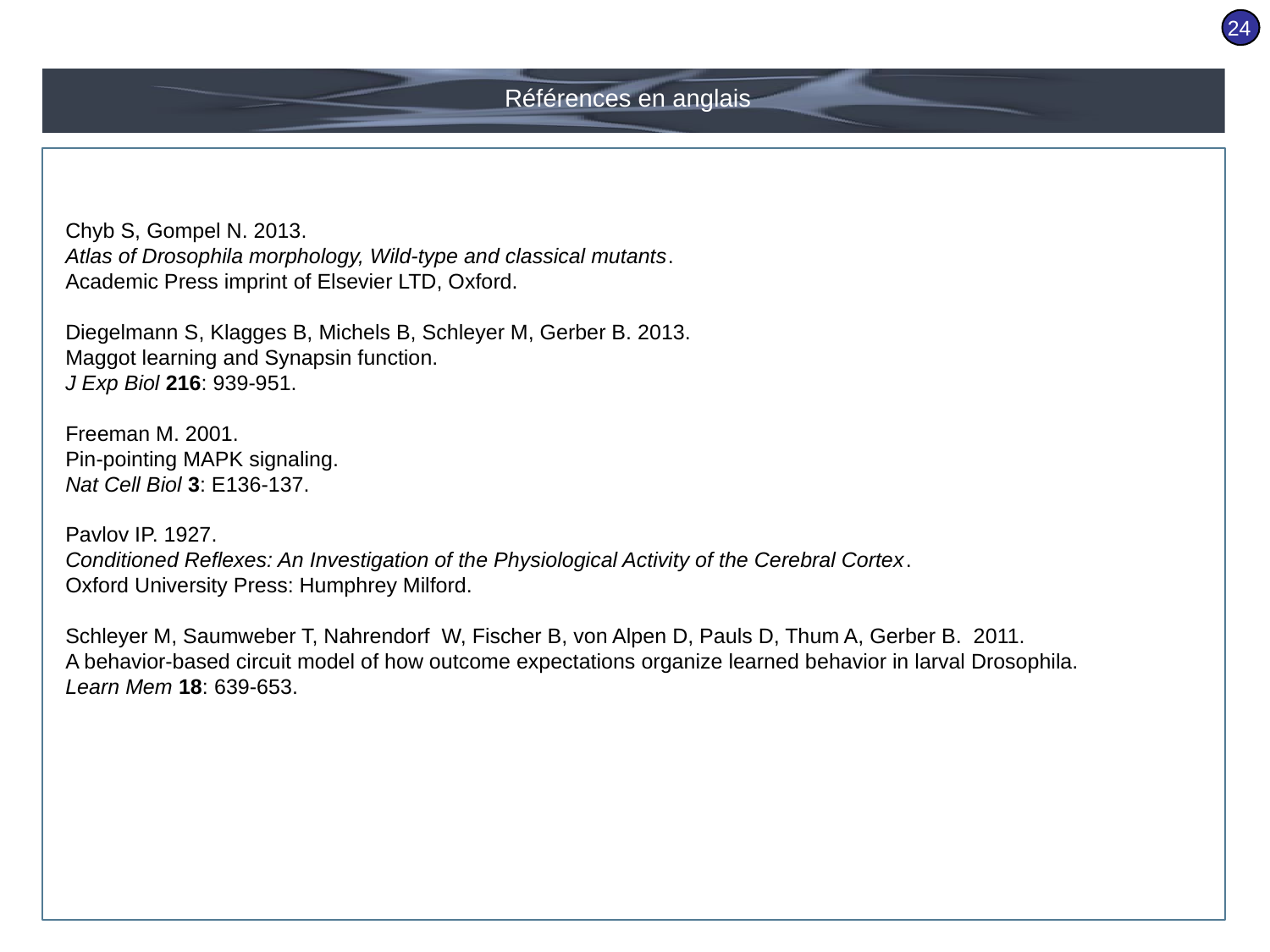

24
Références en anglais
Chyb S, Gompel N. 2013.
Atlas of Drosophila morphology, Wild-type and classical mutants.
Academic Press imprint of Elsevier LTD, Oxford.
Diegelmann S, Klagges B, Michels B, Schleyer M, Gerber B. 2013.
Maggot learning and Synapsin function.
J Exp Biol 216: 939-951.
Freeman M. 2001.
Pin-pointing MAPK signaling.
Nat Cell Biol 3: E136-137.
Pavlov IP. 1927.
Conditioned Reflexes: An Investigation of the Physiological Activity of the Cerebral Cortex.
Oxford University Press: Humphrey Milford.
Schleyer M, Saumweber T, Nahrendorf W, Fischer B, von Alpen D, Pauls D, Thum A, Gerber B. 2011.
A behavior-based circuit model of how outcome expectations organize learned behavior in larval Drosophila.
Learn Mem 18: 639-653.
