# Supplementary material for: Pavlovian Conditioning of Larval Drosophila: An Illustrated, Multilingual, Hands-On Manual for Odor-Taste Associative Learning in Maggots
Source: Front Behav Neurosci. 2017 Apr 19;11:45. doi: 10.3389/fnbeh.2017.00045 (PMC5395560; doi:10.3389/fnbeh.2017.00045)
Supplement: Supplemental Material 10 — A manual for odor-reward learning in larval Drosophila in the Japanese language. Versions of this manual in the English, German, French, Spanish, and Italian languages can be found in Supplemental Materials 1–3, 4–6, 7–9, 11–13, 14–16, respectively. For a table for data analysis and an empty table for entering and analyzing one's own data, please use the document in the English language (Supplemental Materials 2, 3, respectively). [file SupplementalMaterial10.pptx]

## Slide 1
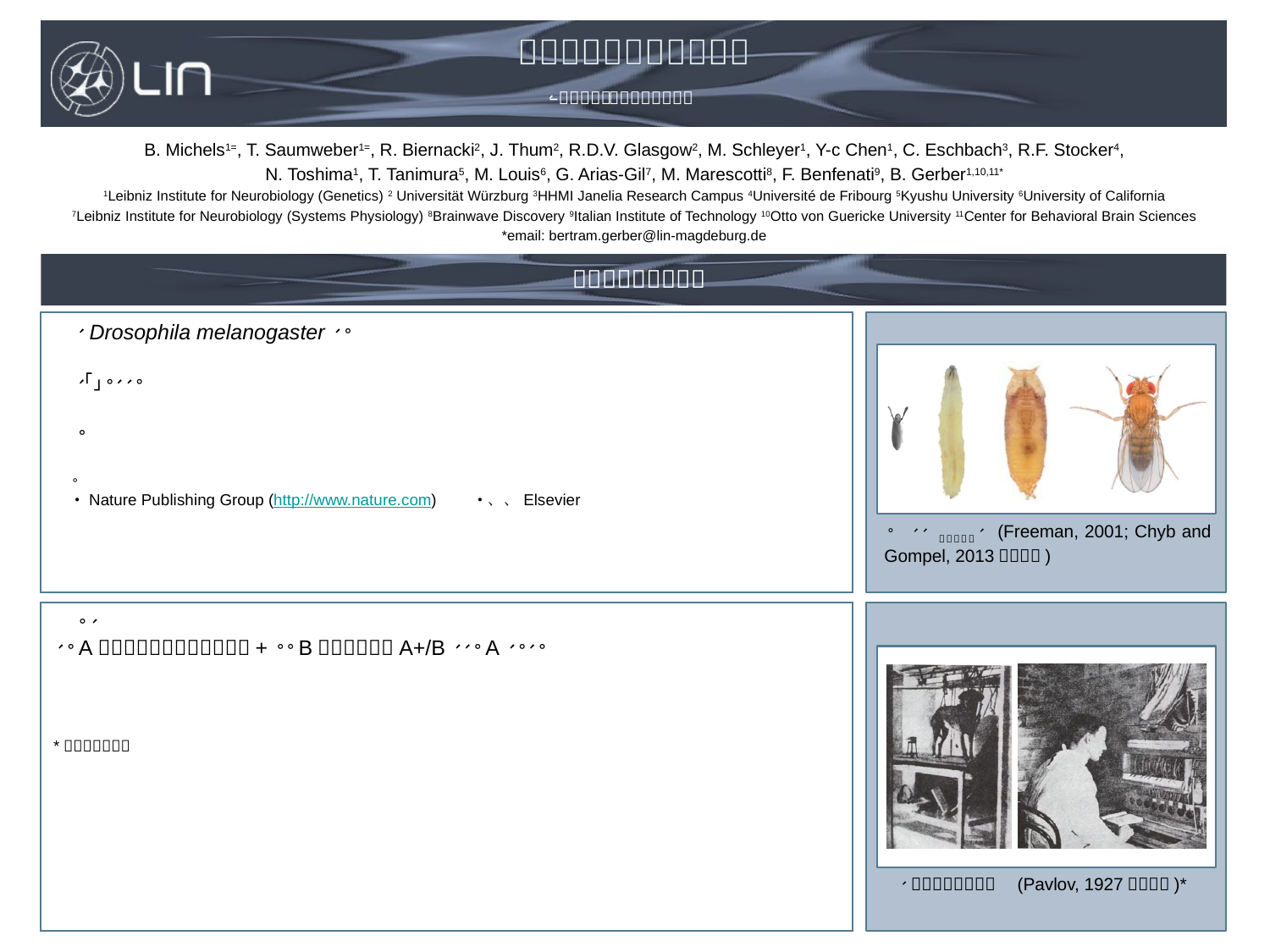

# ショウジョウバエ幼虫を用いたパブロフ式条件付け実験 –図説付き、実践的多言語解説
幼虫学習実験マニュアル
B. Michels1=, T. Saumweber1=, R. Biernacki2, J. Thum2, R.D.V. Glasgow2, M. Schleyer1, Y-c Chen1, C. Eschbach3, R.F. Stocker4,
N. Toshima1, T. Tanimura5, M. Louis6, G. Arias-Gil7, M. Marescotti8, F. Benfenati9, B. Gerber1,10,11*
1Leibniz Institute for Neurobiology (Genetics) 2 Universität Würzburg 3HHMI Janelia Research Campus 4Université de Fribourg 5Kyushu University 6University of California
7Leibniz Institute for Neurobiology (Systems Physiology) 8Brainwave Discovery 9Italian Institute of Technology 10Otto von Guericke University 11Center for Behavioral Brain Sciences
*email: bertram.gerber@lin-magdeburg.de
イントロダクション
　この実験マニュアルは、キイロショウジョウバエ（Drosophila melanogaster）の幼虫を用いて、匂いと味の報酬の連合学習を研究するためのものである。
　この実験手法を用いて、「行動の柔軟性（可塑性）」という興味深い神経機構を理解することができる。ショウジョウバエは多様な遺伝学的な解析が可能で、ヒトとの遺伝的な共通点も多く存在するため、生体臨床医学分野への応用も期待できる。
　実験からデータ解析までを初心者が行えるように懇切丁寧なマニュアルを作成した。
　右上の画像の使用は以下の著作権保持者による許諾済みである。
　・卵：Nature Publishing Group (http://www.nature.com)　　・幼虫、蛹、成虫：Elsevier
ショウジョウバエの生活史。左から：卵、幼虫、蛹（さなぎ）、成虫 (Freeman, 2001; Chyb and Gompel, 2013より転載)
　実験では匂いと糖の連合記憶について調べる。寒天の培地を底に入れたシャーレ内で幼虫は自由に動き回るが、このとき培地は報酬として糖を加えたものか、もしくは糖を含まない無味のものを用いる。匂いAを糖培地と共に提示する（+）。幼虫を次のシャーレに移す。このシャーレでは無味の培地と共に匂いBを提示する。A+/Bの手順をさらに２回繰り返した後、幼虫をテスト用シャーレに移し、２種類の匂いを選ばせる。もし幼虫が報酬と共に提示された匂いAを好んで選択すれば、匂いと糖の連合記憶が形成されたと結論づけられる。つまりこのパラダイムは、パブロフが犬を用いて行った実験と同様な古典的な条件付け実験である。
*著作権期限切れ
パブロフの犬、 実験アシスタント　(Pavlov, 1927より転載)*

## Slide 2
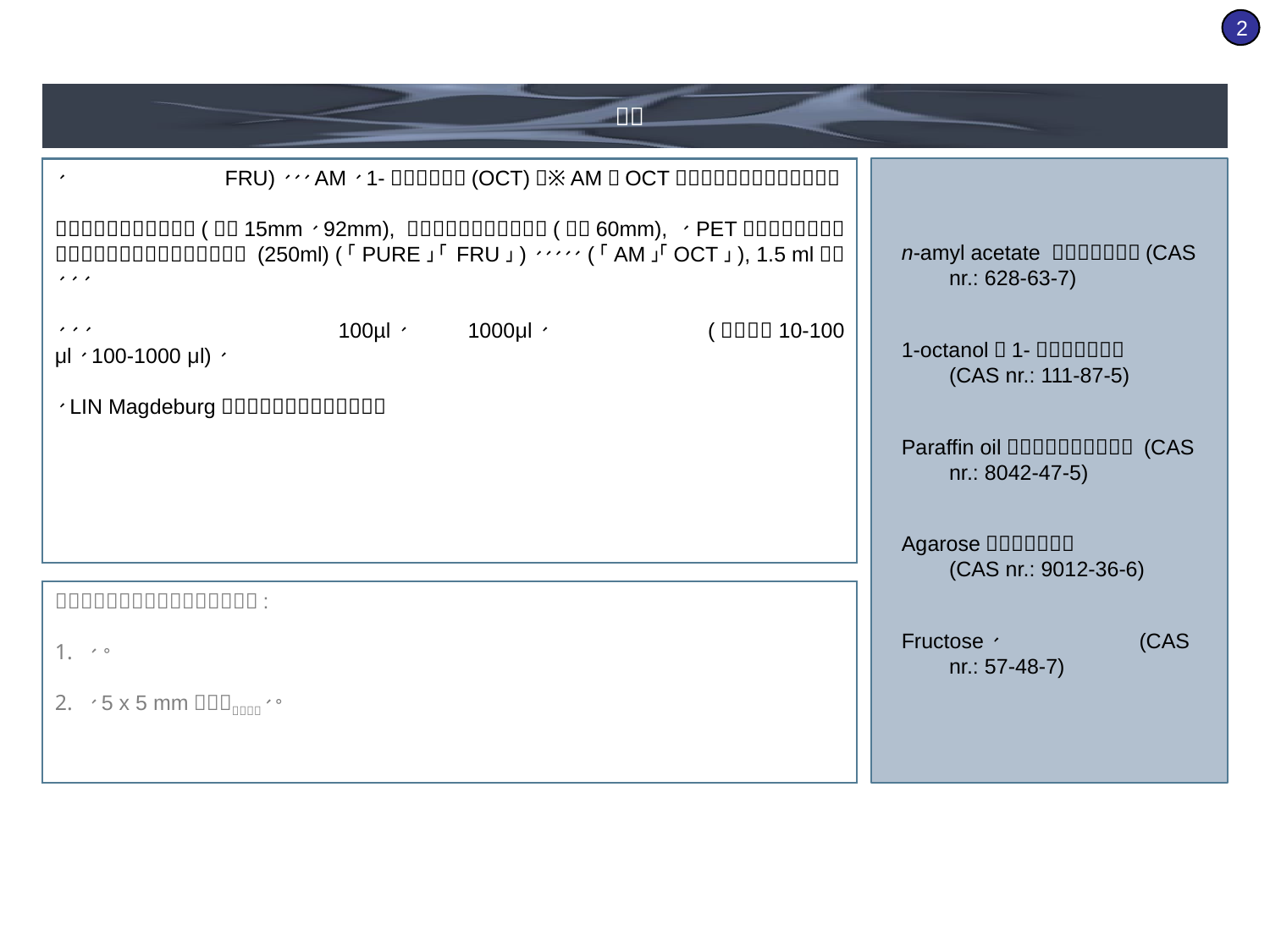

2
材料
アガロース、フルクトース（FRU)、蒸留水、パラフィンオイル、酢酸アミル（AM）、1-オクタノール(OCT)（※AMとOCTは暗所に保管した方がよい）
プラスチックシャーレ大(高さ15mm、直径92mm), プラスチックシャーレ小(直径60mm), 撹拌子（スターラーバー）２個、PETスプレーボトル、電子レンジ可の蓋付きボトル２本 (250ml) (それぞれに「PURE」「FRU」とラベル)、ビーカー、ストップウォッチ、細筆、スパチュラ、湾曲ピンセット２本(それぞれに「AM」「OCT」とラベル), 1.5 mlプラスチックチューブ、ハサミ、ゴミ袋、布巾
マグネティックスターラー、秤、電子レンジ、100µlピペット、1000μlピペット、ピペットチップ (サイズ：10-100 μl、100-1000 μl)、ボルテックスミキサー
シャーレ大の蓋（穴あき）、テフロン製の匂い容器（LIN Magdeburgにサンプルのリクエスト可）
n-amyl acetate （酢酸アミル）(CAS nr.: 628-63-7)
1-octanol（1-オクタノール）　　　 (CAS nr.: 111-87-5)
Paraffin oil（パラフィンオイル） (CAS nr.: 8042-47-5)
Agarose（アガロース）　　　　　(CAS nr.: 9012-36-6)
Fructose（フルクトース、果糖） (CAS nr.: 57-48-7)
学術研究外の用途で実験を行う場合:
穴あきの蓋が入手できない場合は、通常のシャーレの蓋を用いてもよい。
匂い容器が入手できない場合は、匂い物質を5 x 5 mmの濾紙（ろし）に染み込ませ、両面テープでシャーレの蓋に固定する手法でもよい。

## Slide 3
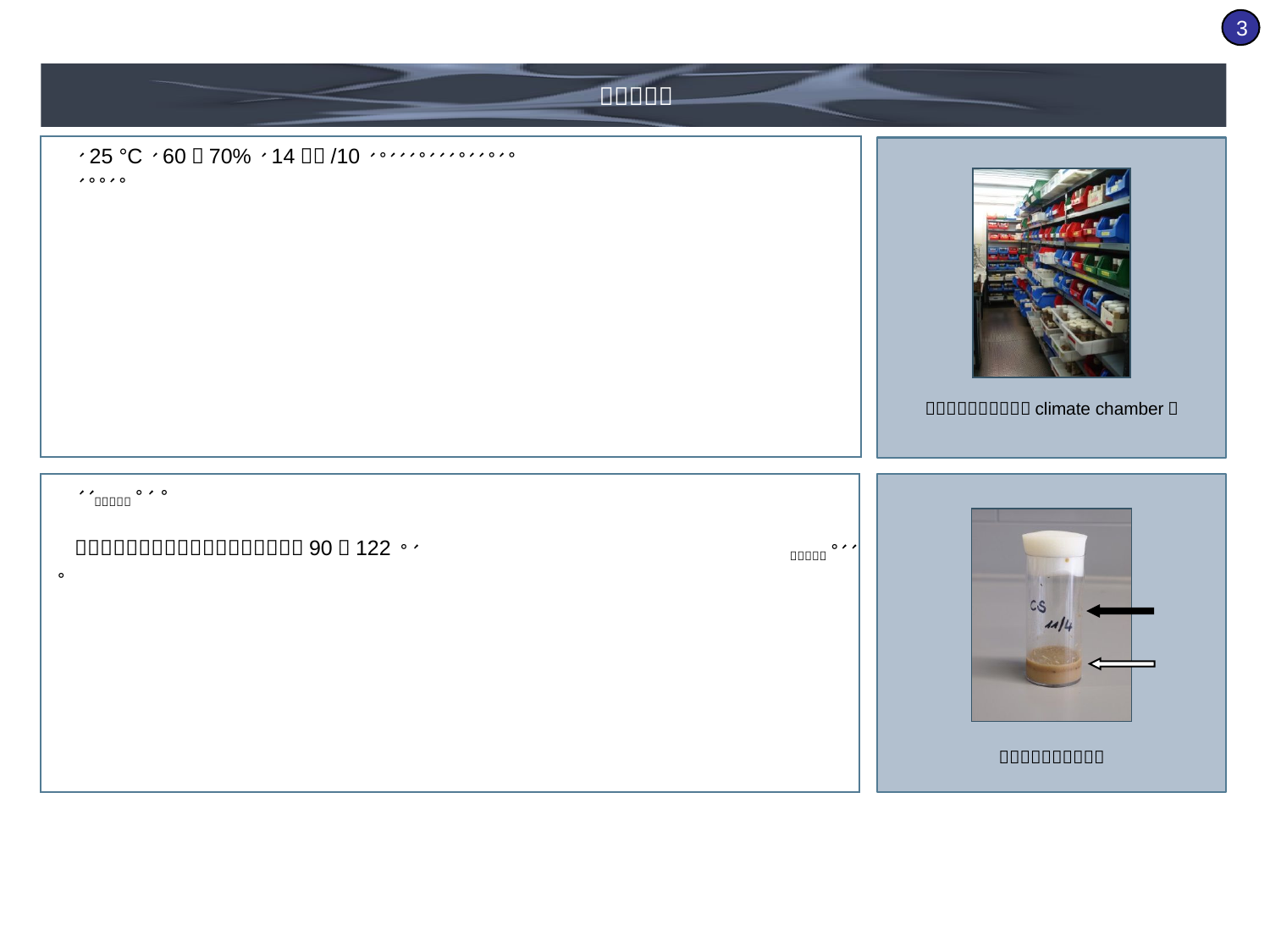

3
# ハエの飼育
　ショウジョウバエは、室温25 °C、湿度60〜70%、14時間/10時間の明暗サイクルの条件下で、培地の入ったバイアルで育てる。学術研究においては、環境が管理された飼育室でハエの飼育を行うが、インキュベータがない場合は、自然光の入る通常の部屋を用いてもよい。培地の材料はコーンミールと寒天粉末、糖、イースト、水である。菌やバクテリアが増殖するのを防ぐため、通常培地には抗生剤や抗菌剤を加え、冷蔵庫に保管して２週間以内に使用する。自分で培地を作ることが難しい場合には、ショウジョウバエ研究を行っている近くの研究施設に相談してみるとよい。
　毎日、同じ時間帯にハエを新しい培地バイアルに移す。バイアルにはハエの系統名と日付を記す。丸１日卵を産ませ、また新たなバイアルにハエを移す。
ハエ飼育用の環境室（climate chamber）
　産卵の後、幼虫期（１齢〜３齢）が約６日間続き、次に約４日間蛹（さなぎ）の時期がある。蛹の殻を破って成虫が羽化し、１〜２日後には交尾を行う。
　実験には５日齢の幼虫を用いる（産卵後90〜122時間）。成長期の幼虫のみを使い、徘徊期にあるものは避ける（蛹化（ようか）直前の幼虫は使わないこと）。そのためには、壁面を動き回って蛹になる場所を探している幼虫（黒矢印）ではなく、培地の上層（白矢印）から幼虫を集めるとよい。
培地バイアル内の幼虫

## Slide 4
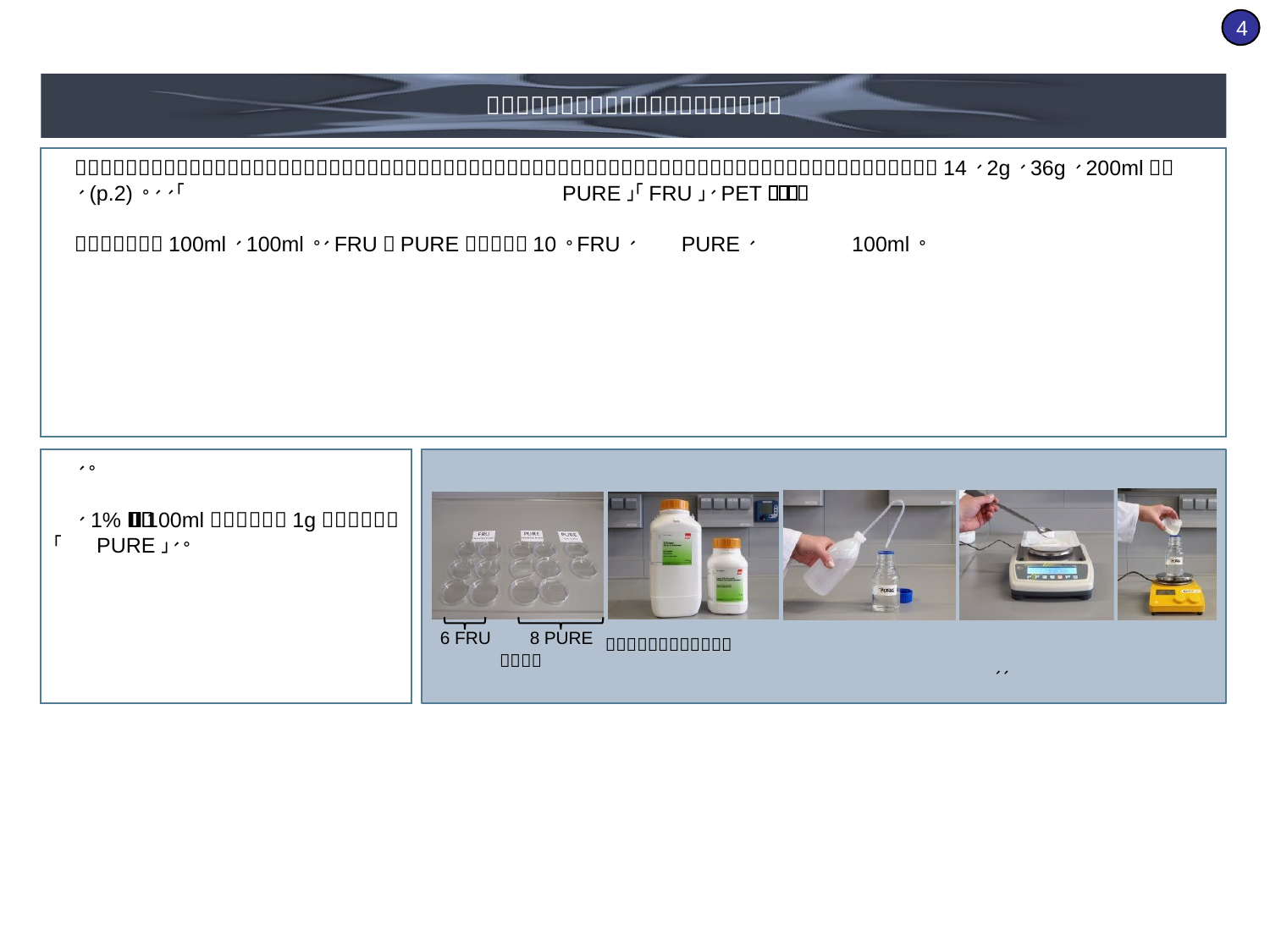

4
# トレーニング及びテスト用のシャーレの準備
　高等学校や大学の授業で実験を行う場合、学生２人１組で準備を行うものとして説明する。そのため、材料は実験２回相当分が必要である（シャーレ大14枚、アガロース2g、フルクトース36g、蒸留水200ml）。
　さらに、材料リスト(p.2)より次のものが必要である。：秤、電子レンジ、蓋付きガラス瓶２本（「PURE」「FRU」とラベルする）、PETスプレーボトル、マグネティックスターラー、撹拌子（スターラーバー）２個、布巾
　アガロース溶液100ml、フルクトース溶液100mlを調製するための手順を次に示す。その分量から、それぞれのシャーレ（FRUとPURE）がおよそ10枚ずつ作成できる。１組の実験に必要なシャーレはFRUが６枚、PUREが８枚なので、各100mlで十分である。
　シャーレを平らな場所に並べ、必要なものを揃える。
　次に、1%アガロース溶液を調製する。蒸留水100mlとアガロース1gをガラスボトル（「PURE」とラベル）に入れ、蓋をして静かに振る。
6 FRU 8 PURE
フルクトースとアガロース
シャーレ
ボトルに水を入れ、アガロースを計り、アガロースを加える

## Slide 5
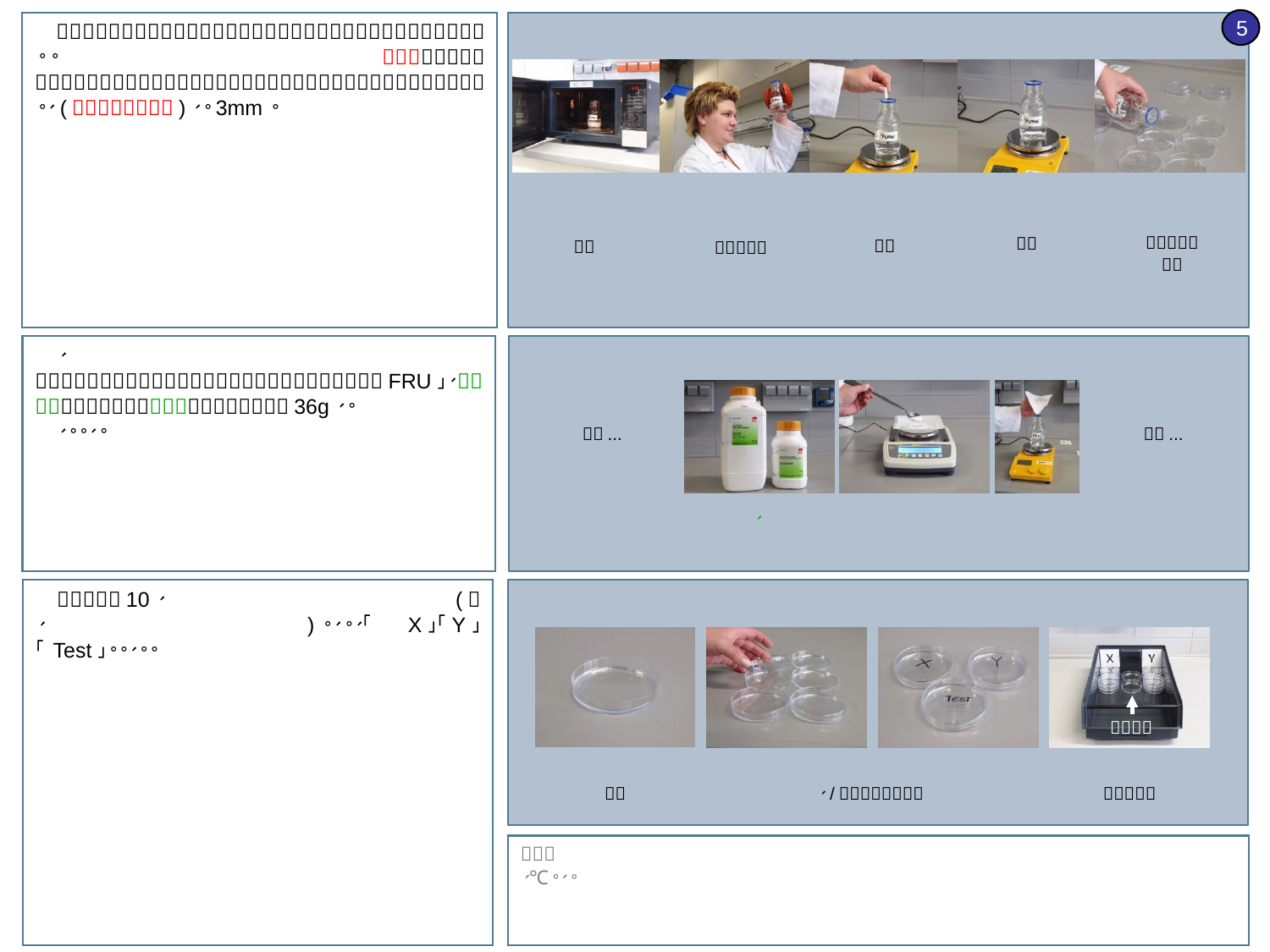

5
　アガロース溶液を中くらいの強度で２〜３分電子レンジにかけ、沸騰させる。溶液が透明になってきたら加熱を止める。布巾を使って熱したアガロース溶液のボトルを電子レンジから取り出す。アガロースはすぐには溶けない。溶け残りがある場合はさらに加熱する。完全に溶けたのを確認したら、攪拌子を入れ(液の跳びはね注意)、静かに撹拌しながら１〜２分冷却する。アガロース溶液をシャーレに3mm程度の層になるようにして注ぐ。
シャーレに
注ぐ
冷却
撹拌
沸騰
溶解を確認
　フルクトース入りのシャーレを作製する時は、上記手順の加熱したアガロース溶液を電子レンジから取り出した後に（ボトルには「FRU」とラベル）、以下の手順をでフルクトースを加える。：フルクトース36gを計り、アガロース溶液に加える。
　その後、溶液を撹拌する。フルクトースは溶解しやすい。溶液が透明になったのを確認したら、上記手順と同様にシャーレに注ぐ。
沸騰...
撹拌...
フルクトースを秤量した後、アガロース溶液に加える
　シャーレは10分ほど冷却させた後、乾燥を防ぐために蓋を閉じる(蓋をするのが早すぎると、内部に結露ができる)。シャーレを取り違えることがないよう、ラベルする。実験をブラインド条件で行う場合には、実験者の見ていない所で「X」「Y」「Test」のようにラベルする。実際の培地条件は実験後に開示する。シャーレは箱にいれ、翌日使うまで室温で保管する。窓際や暑くなる場所には置かない（結露や乾燥を防ぐため）。
テスト用
固化
蓋を閉じ、ラベル/コードを書き込む
準備完了！
メモ：
実験を複数回繰り返す場合には、シャーレをまとめて作製して冷蔵庫（４℃）で１週間まで保管できる。このとき、シャーレは蓋を下側にして重ねる（結露が培地表面に落ちるのを防ぐため）。

## Slide 6
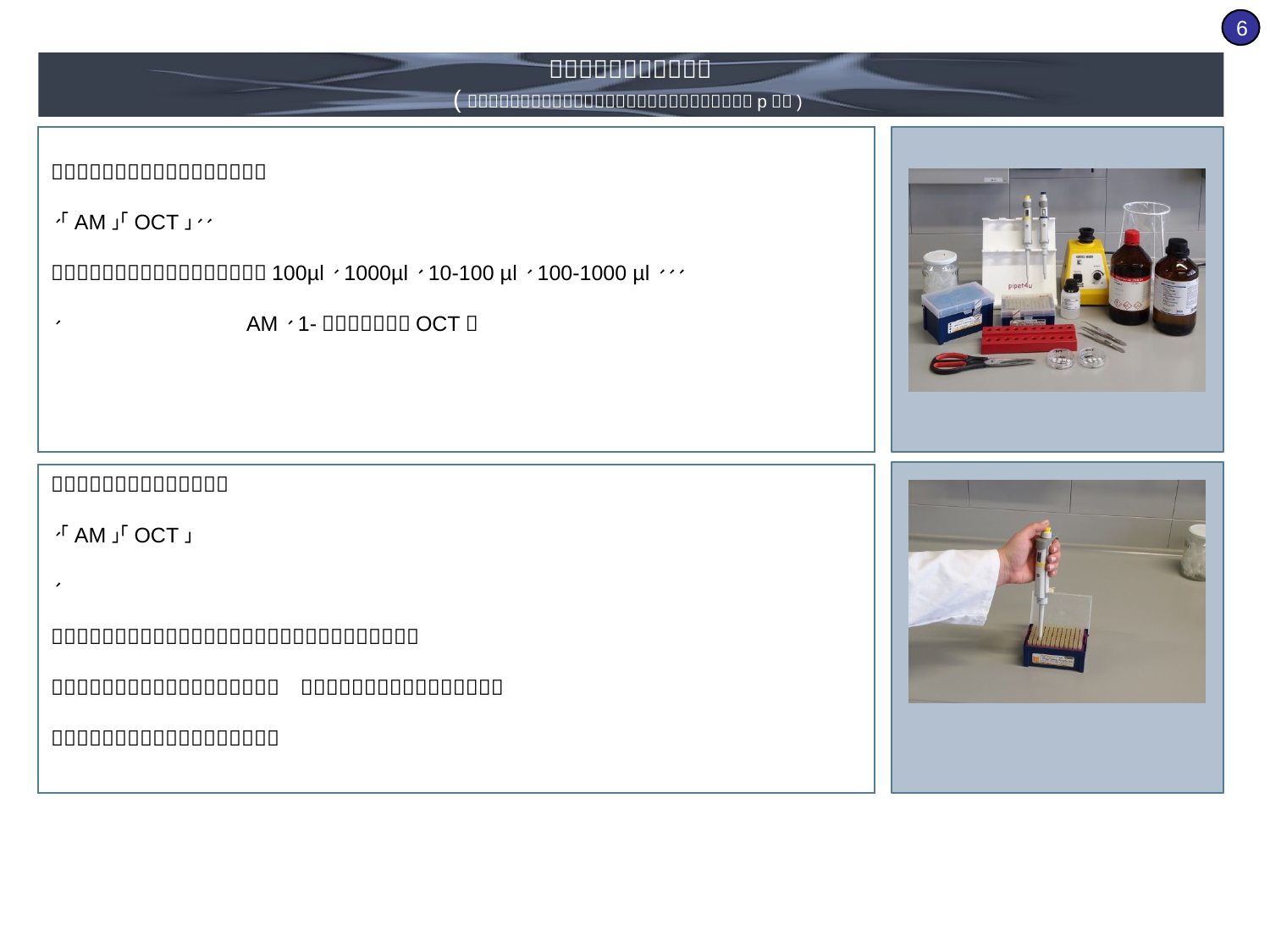

6
# 匂い物質の容器への充填 (学術研究でない場合：匂いの提示法として濾紙の使用も可：p９へ)
実験のセットアップ毎に必要な材料：
匂い容器保管用のシャーレ小２枚、湾曲ピンセット２本（「AM」「OCT」とラベルし、それぞれの匂い専用とする）、匂い物質の充填容器８個
プラスチックチューブ、ピペット小（100µl）、ピペット大（1000µl）、ピペットチップ小と大（10-100 µl、100-1000 µl）、ボルテックスミキサー、ハサミ、ゴミ袋
 
パラフィンオイル、酢酸アミル（AM）、1-オクタノール（OCT）
匂い物質の充填を始める前に：
シャーレ小２枚の蓋に、それぞれ「AM」もしくは「OCT」と書き込む
匂い物質の充填作業は、できればドラフト内で行うのがよい
ピペットで異なる匂い物質を吸う前には新たなチップに交換する
ピペットチップに手で触れてはいけない　常にピペットのエジェクターを使う
ピンセットは匂い物質ごとに使い分ける

## Slide 7
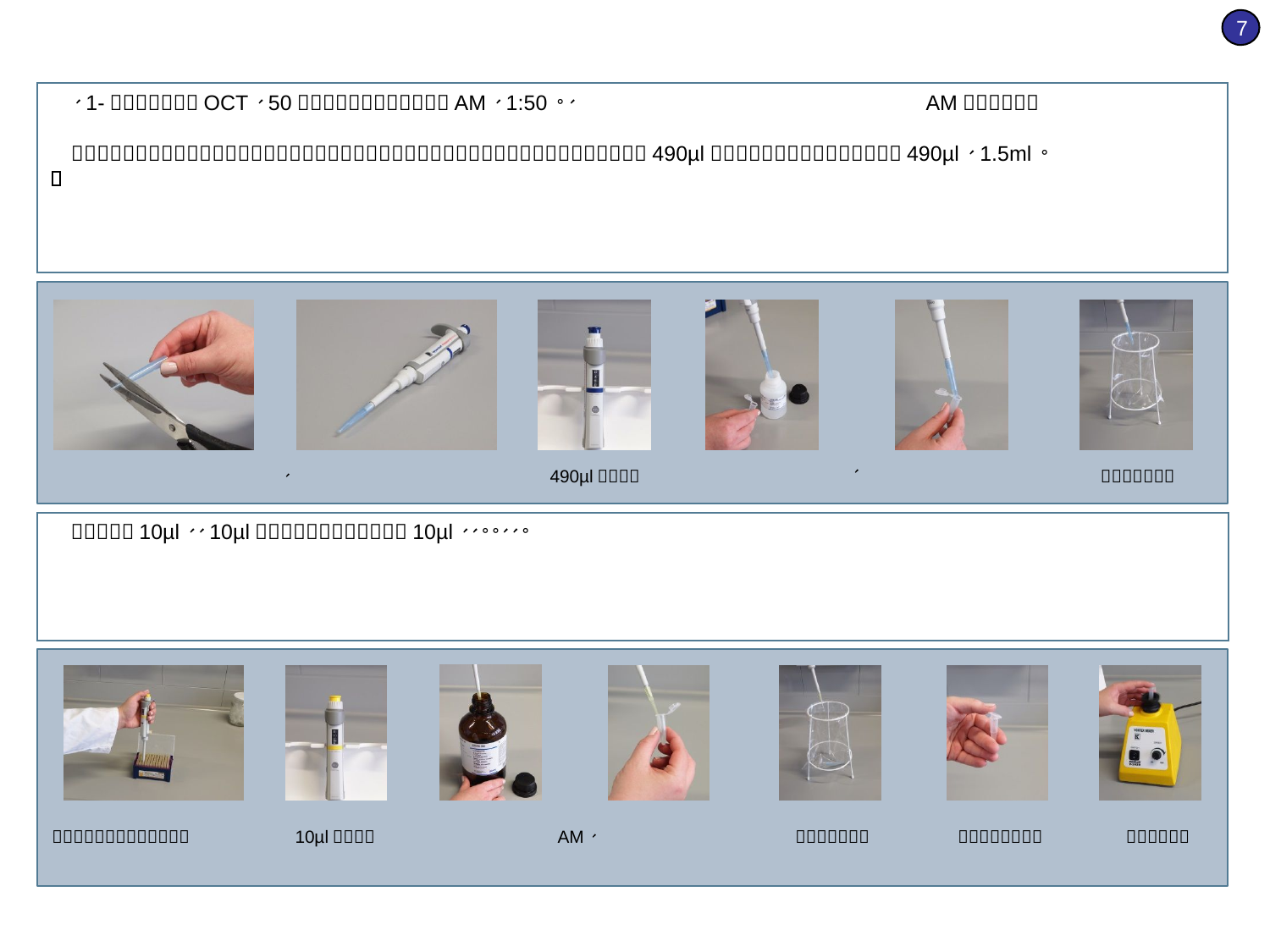

7
　匂い物質として、希釈なしの1-オクタノール（OCT）と、パラフィンオイルで50倍に希釈した酢酸アミル（AM、1:50）を用いる。パラフィンオイルは粘性が高いため、以下の手順でAMを希釈する：
　ピペットチップ大の先端をハサミで切り落としたものを、ピペットに装着。ピペットのボリュームを490µlにセットする。パラフィンオイル490µlをゆっくり吸い上げ、1.5mlチューブに入れる。使用済みのチップはゴミ袋に捨てる。
パラフィンを吸い上げ、チューブに入れる
チップ大の先端を切り落とし、ピペットに装着
490µlにセット
チップを捨てる
　酢酸アミル10µlを添加するために、まずピペット小にチップを装着し、10µlにセットする。酢酸アミル10µlを吸い上げ、先にパラフィンオイルを入れたチューブに添加し、ピペッティング（吸い上げと押し出し）を繰り返し混ぜ合わせる。チップは廃棄する。チューブの蓋を閉じ、ボルテックスミキサーに約３０秒かけ、よく混ぜ合わせる。
ピペット小とチップ小を使用
10µlにセット
AMを吸い上げ、チューブに入れる
チップを捨てる
チューブを閉じる
ボルテックス

## Slide 8
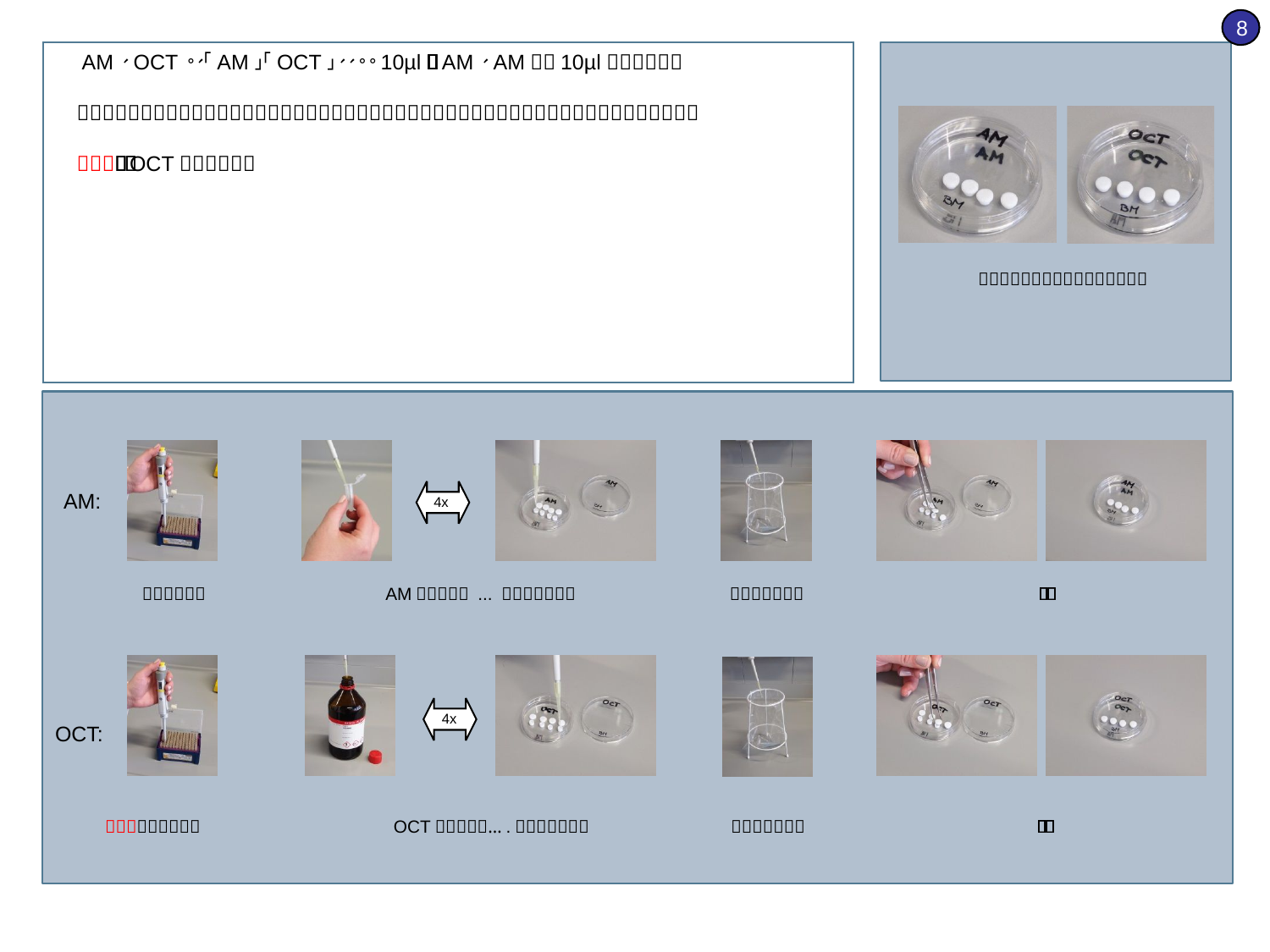

8
　AMを入れた匂い容器４個、OCTを入れた匂い容器４個が必要である。まず、２枚のシャーレ小に「AM」「OCT」とラベルし（実験を複数人のチームで行う場合には、実験者の名前もラベル）、それぞれに匂い容器を４個ずつ入れる。匂い容器の蓋をとる。ピペット小を10µlにセットしてチップ小を取り付ける。AM用の匂い容器４個それぞれに、希釈したAM溶液10µlを充填する。
　ピペットチップを捨て、ピンセットを使って匂い容器の蓋を閉じる。蒸発を防ぐため、シャーレの蓋をする。
　新たなチップに交換し、同様にOCTを充填する。
シャーレ小に匂い容器を入れた状態
4x
AM:
チップを装着
AMを吸い上げ ... 容器４個に添加
チップを捨てる
匂い容器を閉じ、シャーレを閉じる
4x
OCT:
新しいチップを使う
OCTを吸い上げ….容器４個に添加
チップを捨てる
匂い容器を閉じ、シャーレを閉じる

## Slide 9
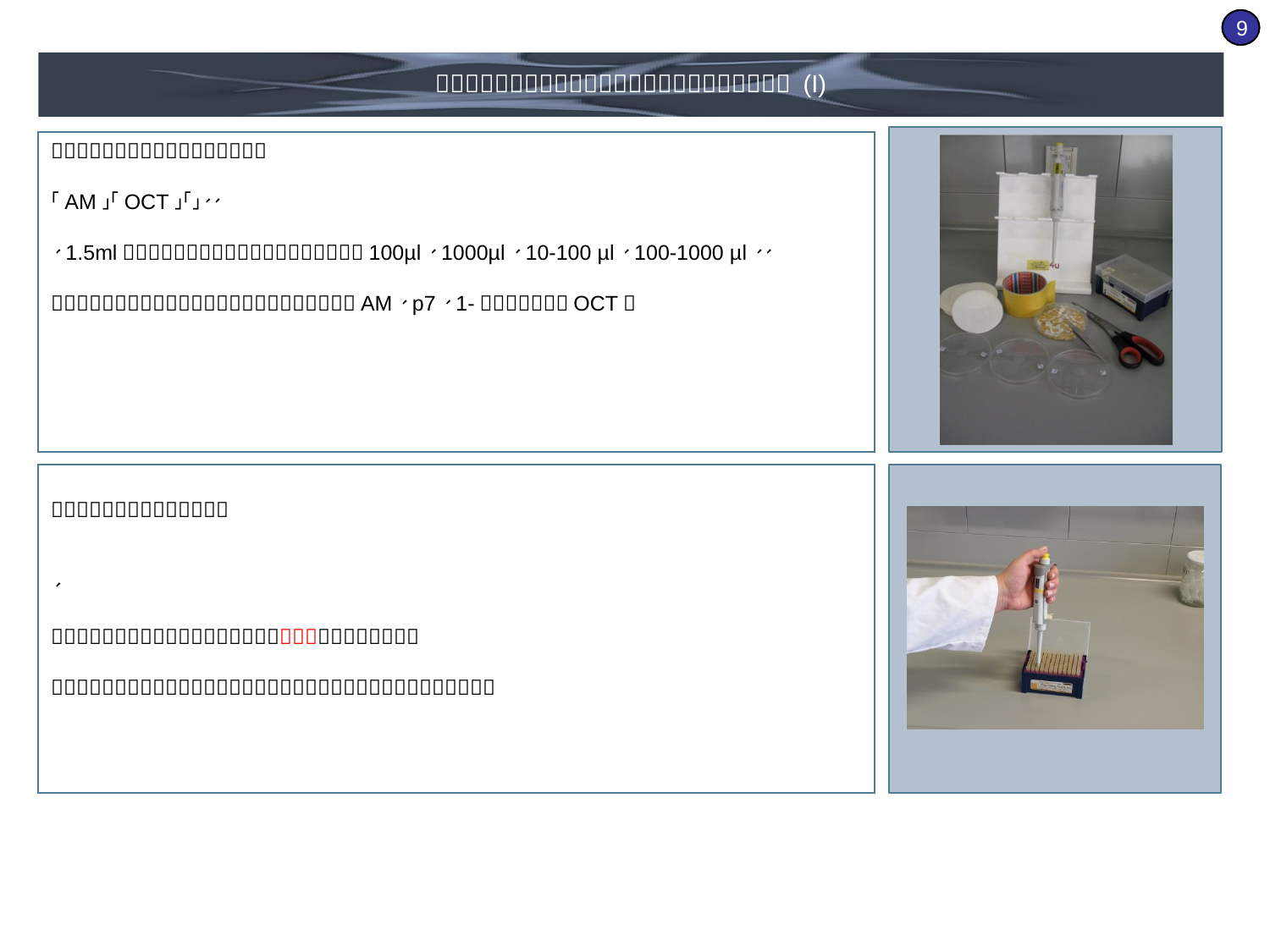

9
# 学術研究でない場合：濾紙を使った匂い物質の提示法 (I)
実験のセットアップ毎に必要な材料：
穴あきのシャーレ蓋３枚（それぞれ「AM」「OCT」「テスト」用）、細いピンセット１つ（濾紙をシャーレ蓋に貼付けるため）、シャーレ大１枚（切った濾紙の保管用）
両面テープ、1.5mlプラスチックチューブ１個、ピペット小（100µl）、ピペット大（1000µl）、ピペットチップ小と大（10-100 µl、100-1000 µl）、ハサミ、ゴミ袋
 
匂い物質：パラフィンオイルで希釈した酢酸アミル（AM、調製法はp7参照）、1-オクタノール（OCT）
匂い物質の充填を始める前に：
匂い物質の濾紙への添加作業は、できればドラフト内で行うのがよい
ピペットで異なる匂い物質を吸う前には新たなチップに交換する
ピペットチップに手で触れてはいけない。常にピペットのエジェクターを使う

## Slide 10
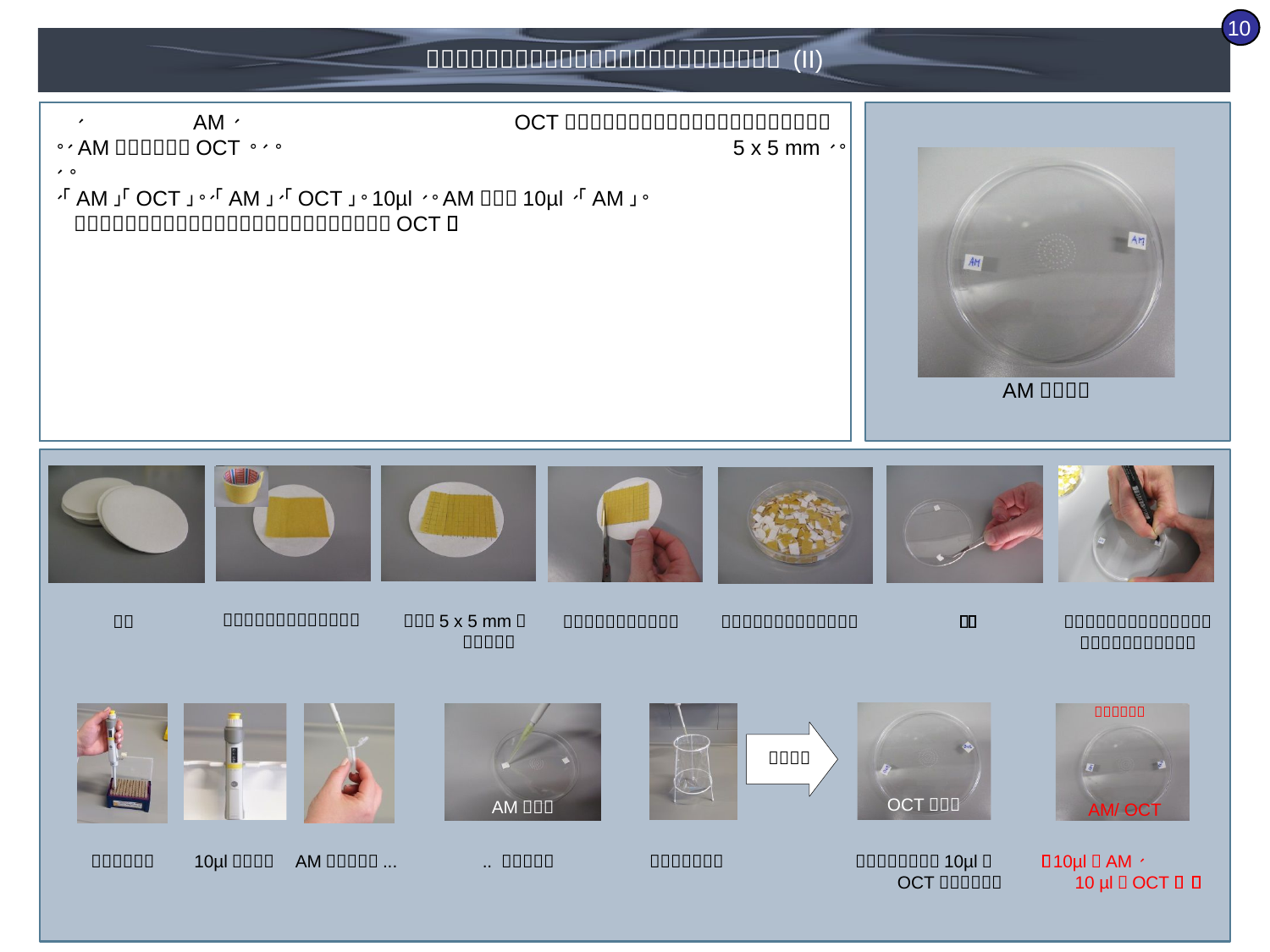

10
# 学術研究でない場合：濾紙を使った匂い物質の提示法 (II)
　トレーニングには、AM用としてシャーレの蓋１枚に正方形の濾紙２枚、OCT用として同様にシャーレの蓋１枚に正方形濾紙２枚が必要である。テストには、シャーレの蓋１枚に対してAMの濾紙１枚とOCTの濾紙１枚が必要である。まず、両面テープを濾紙に貼付ける。ペンで両面テープに5 x 5 mm角の正方形になるよう線を引き、線に合わせて切り取る。細いピンセットを使って両面テープの保護シートをはぎ取り、シャーレの蓋内側の両端に１枚ずつ貼付ける。トレーニング用のシャーレには、濾紙を貼付けた場所の蓋の外側に「AM」（右写真参考）もしくは「OCT」とラベルする。テスト用のシャーレには、片方の濾紙には「AM」、もう片側には「OCT」とラベルする。ピペット小を10µlにセットし、チップ小を装着する。希釈したAM溶液を10µlずつ、「AM」とラベルした３カ所の濾紙に添加する。
　ピペットチップを捨て、新たなチップを装着し、同様にOCTを３カ所の濾紙に添加する。
AM添加の蓋
濾紙に両面テープを貼付ける
濾紙に5 x 5 mm角の線を引く
濾紙
濾紙を線に合わせて切る
切った濾紙はシャーレに保管
テープをはがし、シャーレの蓋内側に貼付ける
シャーレの外側に添加する匂い物質の名前をラベルする
テスト用の蓋
繰り返す
OCT用の蓋
AM用の蓋
AM/ OCT
チップを装着
10µlにセット
AMを吸い上げ...
.. 濾紙に添加
チップを捨てる
同様にピペットで10µlのOCTを濾紙に添加
テスト用には、10µlのAMを片方の濾紙に、10 µlのOCTをもう一方の濾紙に添加すること

## Slide 11
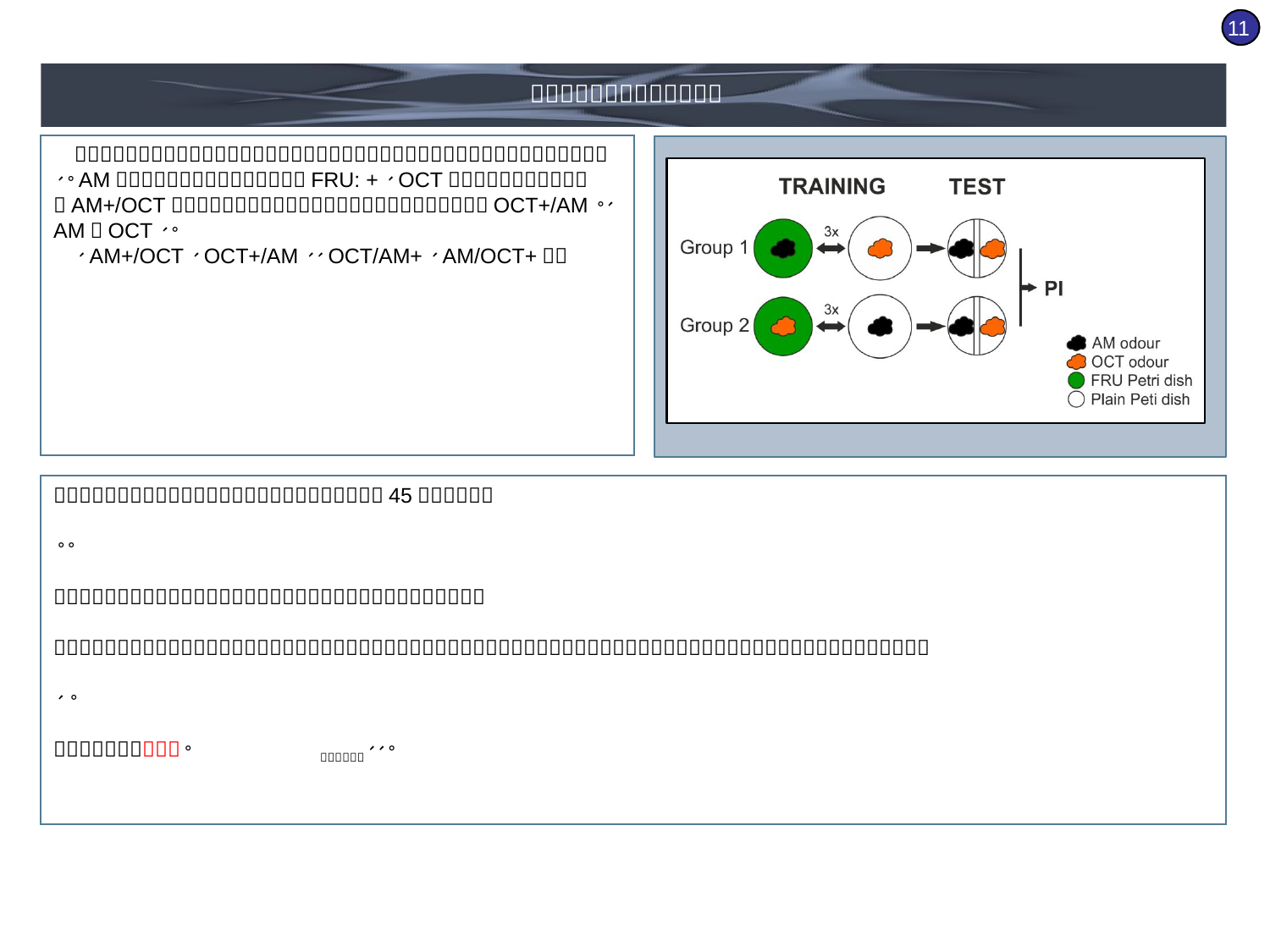

11
トレーニングとテストの概要
　実験は対応する２グループで１セットである。対応グループのうち片方のトレーニング手順を行った後、テストを行う。グループ１のトレーニングではAMと共にフルクトース報酬を提示（FRU: +）し、OCTは報酬無しで提示される（AM+/OCT）。グループ２は匂いと報酬の組み合わせを逆にする（OCT+/AM）。その後のテストでは、幼虫にAMとOCTの匂いを選択させ、対応する２グループの幼虫がどのようにテストシャーレ上に分布するかを見る。
　順序効果を相殺するため、実験のうち半分は報酬を先に提示し（AM+/OCT、OCT+/AM、右図参照）、残りの半分は報酬を後に提示する（OCT/AM+、AM/OCT+）。
トレーニングからテストまでを終えるのに慣れた実験者で45分を要する。
冬に実験を行う場合には室温に注意する。幼虫は変温動物であり寒さは実験に影響する。
ランプや窓、暖房などからの強い光や熱が片側から当たらないようにする。
実験中はテーブルを揺らさない。実験台上での道具の取り扱い、携帯電話のバイブレーションなどには注意し、また手や足でテーブルに振動を加えない。
実験室では、食べ物や香水などの強い匂いがないようにする。
シャーレは毎回新しいものを使う。幼虫が口鉤（こうこう）でアガロース表面に傷をつけるため、繰り返し使用すると、幼虫がその傷からアガロース内部に潜り込むことがある。

## Slide 12
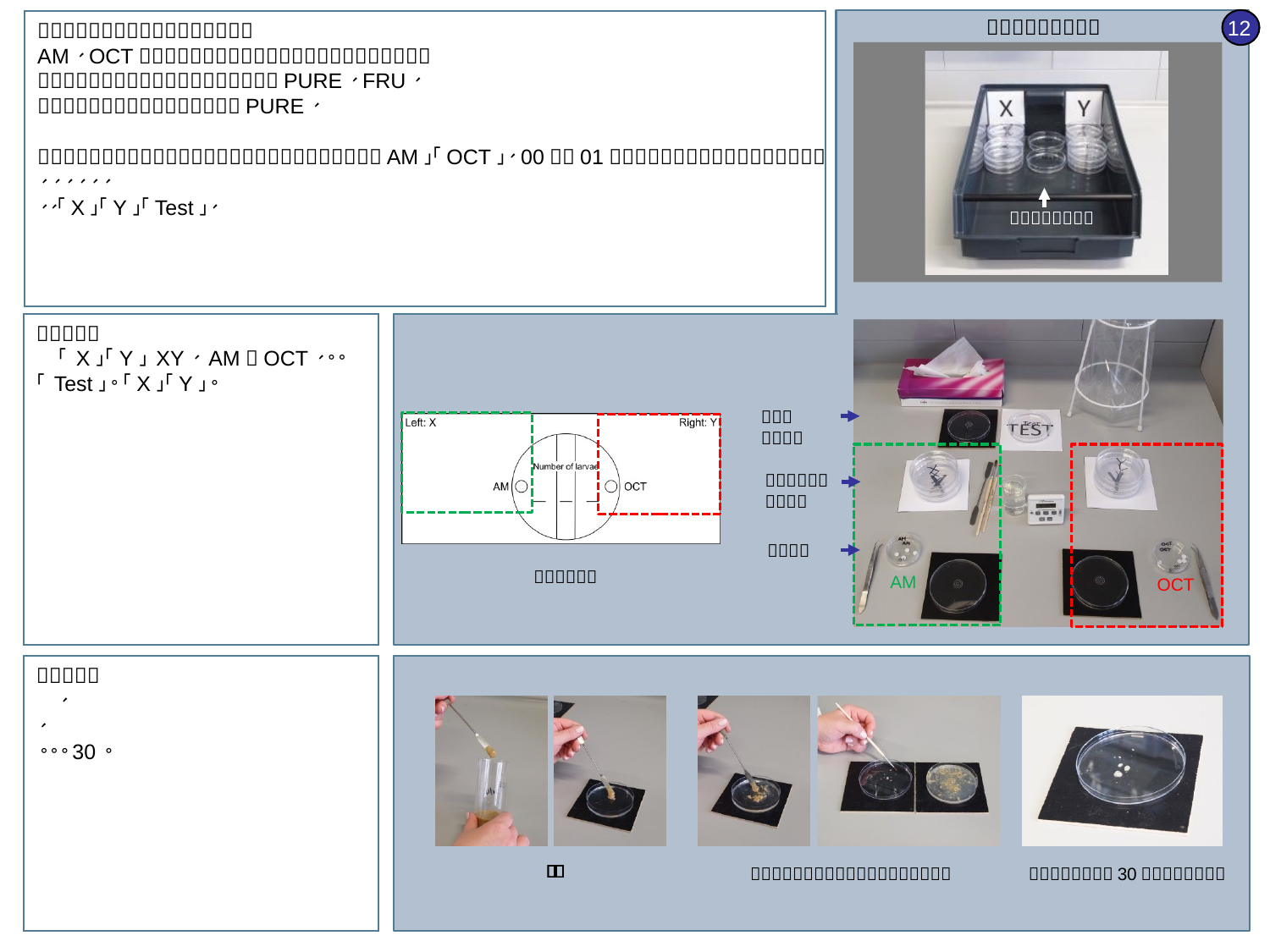

実験のセットアップ
12
実験のセットアップ毎に必要な材料：
AM匂い容器４個、OCT匂い容器４個（学術研究用のセットアップの場合）
トレーニング：アガロースシャーレ６枚（PURE）、フルクトースシャーレ６枚（FRU）、穴あきのシャーレ蓋２枚
テスト：アガロースシャーレ２枚（PURE）、穴あきのシャーレ蓋１枚
ストップウォッチ１個、湾曲ピンセット２本（それぞれに「AM」「OCT」とラベル）、筆２本（サイズ00号と01号を１本ずつ）、空のシャーレ１枚、スパチュラ、ティッシュ、ビーカー、水道水、ゴミ袋、トレーニング用の黒い板２枚（黒い方が幼虫が見やすいため）、テスト用の黒い板にシャーレの位置をトレースしたもの１枚、データシート、シャーレを置く位置を示すための紙（「X」「Y」「Test」と書かれたもの）、５日齢の幼虫の入った培養バイアル
テスト用シャーレ
道具の配置
　「X」「Y」と書かれた紙とXYそれぞれのシャーレ、AMとOCTを添加した匂い容器を、データシートに記された通りの配置に並べる。１巡目のトレーニング用シャーレをトレーニング用の黒い板上に置く。「Test」と書かれた紙とテスト用シャーレは奥に置いておく。 （「X」「Y」はブラインド条件で実験を行うためのコードである。）
テスト
シャーレ
OCT
Y
トレーニング
シャーレ
匂い容器
データシート
AM
OCT
幼虫の準備
　飼育バイアルの蓋を外し、スパチュラを使って幼虫をエサと共にひとすくい取り出し、水を張ったシャーレに移す。培養バイアルの蓋を閉じる。筆を使って静かに幼虫をエサから取り出す。約30匹の幼虫を空のシャーレに集める。
培養バイアルからエサをひとすくい取り出し、水を入れたシャーレに移す
エサと幼虫を静かに混ぜて幼虫を取り出す
空のシャーレに約30匹の幼虫を集める

## Slide 13
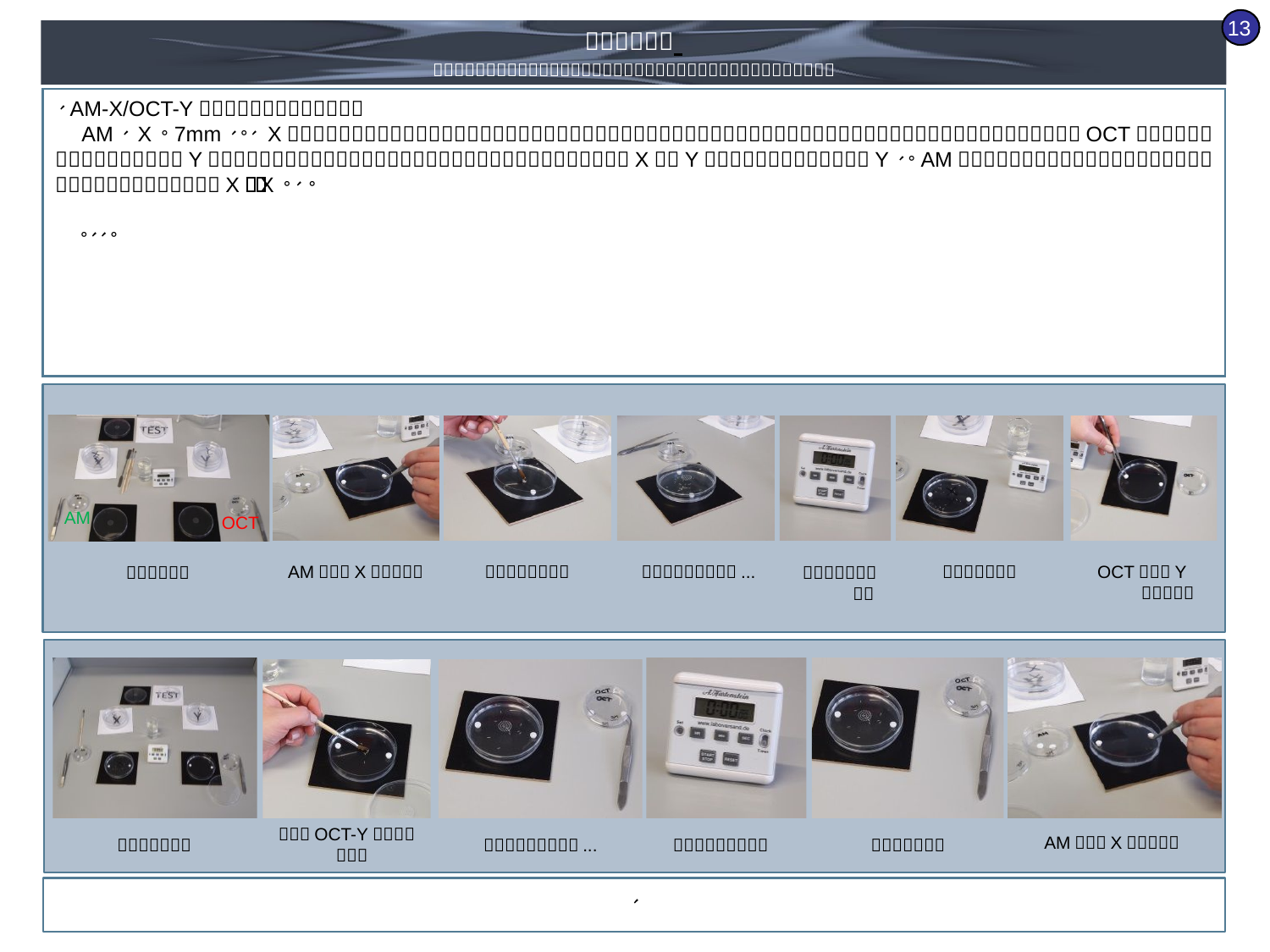

13
# トレーニング
（学術研究でない場合：匂い容器の代わりに濾紙を貼付けたシャーレの蓋を用いる）
一例として、AM-X/OCT-Yのトレーニング手順を示す：
　AM容器２個をシャーレ小から取り出し、トレーニング用シャーレX内に置く。シャーレの端から7mmほど離して、対角線上になるように配置する。筆を使って、幼虫をトレーニング用シャーレXの中央に移す。穴あきのシャーレ蓋で蓋をし、ストップウォッチのカウントを開始する。ストップウォッチが４分を示したタイミングで、OCT容器２個をトレーニング用シャーレY内にセットする。ストップウォッチが５分を示したタイミングで、幼虫をXからYのシャーレに移す。シャーレYに穴あきの蓋をし、ストップウォッチをリセットして再開する。AM容器はシャーレ小の中に戻しておき、使用済みのトレーニング用シャーレXは廃棄する。２巡目のトレーニング用シャーレXを１巡目と同様に配置する。２巡目、３巡目のトレーニングを同様の手順で行う。
　注意：常に全部の幼虫を移す（培地内に潜り込んだものを除いて）。シャーレの蓋、壁、匂い容器上にいる幼虫もすべて集める。
AM
OCT
AM容器をXシャーレへ
タイマー４分：
中央に幼虫を置く
シャーレの蓋を閉じ...
OCT容器をYシャーレへ
開始時の状態
タイマースタート！
幼虫をOCT-Yシャーレに移動
AM容器をXシャーレへ
シャーレの蓋を閉じ...
タイマー５分：
タイマースタート！
タイマー４分：
２巡目、３巡目のトレーニングも同様の手順で行う

## Slide 14
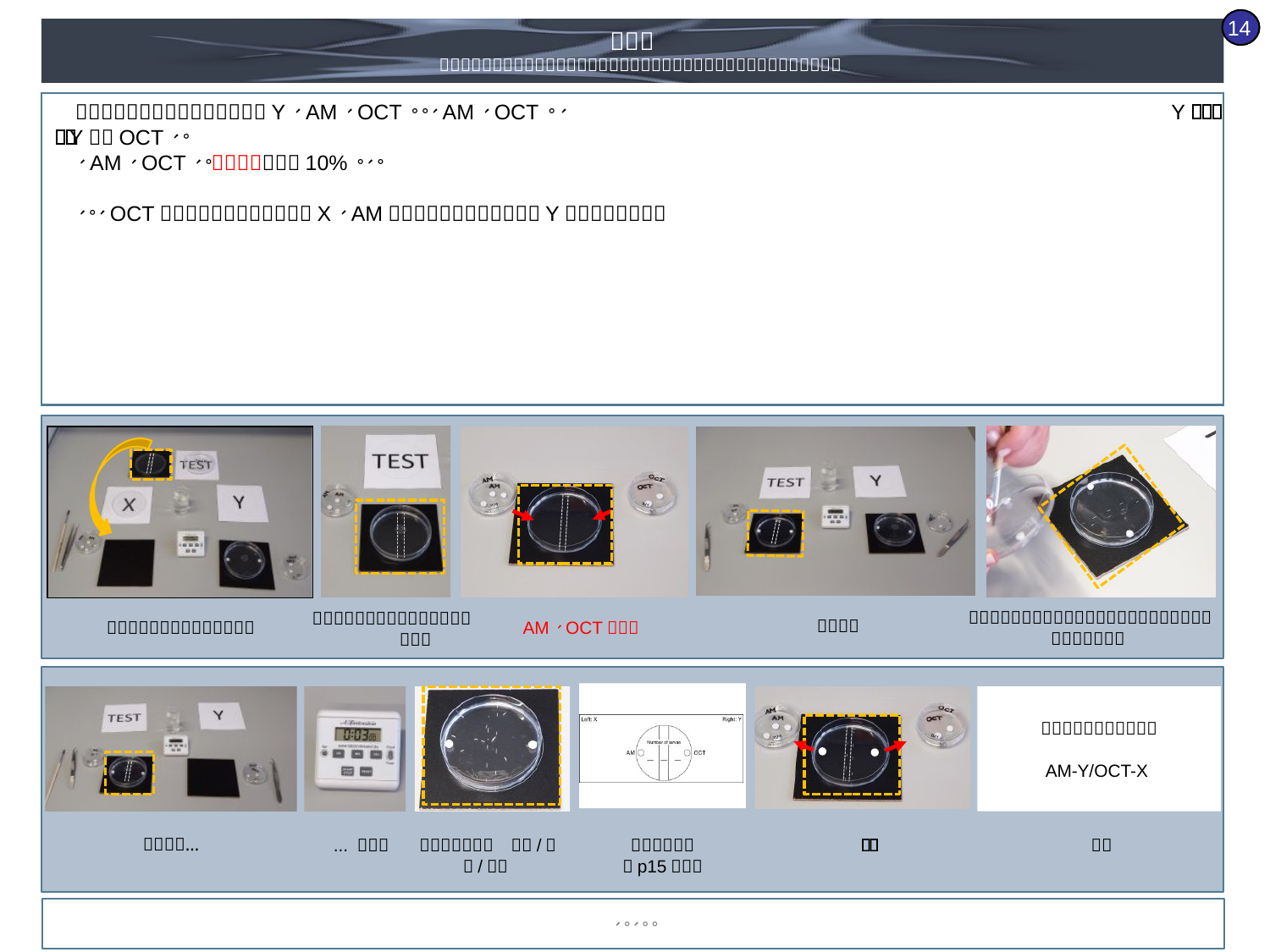

# テスト
14
（学術研究でない場合：匂い容器の代わりに濾紙を貼付けたシャーレの蓋を用いる）
　３巡目のトレーニング用シャーレYに幼虫を入れてから４分経過した時に、テスト用シャーレの左端にAM容器を、右端にOCT容器を置く。（匂い容器の取り扱いは混乱しやすいので要注意。この時、小シャーレ内にAM容器は３個、OCT容器は１個残っているはずである。）最後のトレーニングが５分経過したら、幼虫をトレーニング用シャーレYからテスト用シャーレに移す。幼虫は中央ゾーン内に置く。穴あきのシャーレ蓋でテスト用シャーレに蓋をし、ストップウォッチをリセットして再開する。３巡目のトレーニング用シャーレY上のOCT容器はシャーレ小に戻し、使用済みのシャーレは廃棄する。
　テスト開始後３分経過したら、AM側、OCT側の幼虫の数をカウントし、データシートに書き取る。シャーレの蓋に登っている幼虫は数えない（通常10%以下である）。匂い容器はそれぞれシャーレ小に戻し、使用済みのシャーレと幼虫は廃棄する。
　テストを終えたら、対応グループの実験を新たに集めた幼虫で始める。実験手順は同様であるが、今度はOCTをトレーニング用シャーレXと共に、AMをトレーニング用シャーレYと共に提示する。
タイマー５分：幼虫をテスト用シャーレに移し、タイマーを再始動
タイマー４分：テスト用シャーレを準備
再確認！
AMを左、OCTを右に
最後のトレーニング（３巡目）
対応グループの実験へ：
AM-Y/OCT-X
テスト中…
幼虫をカウント　（左/中央/右）
次へ
... ３分後
データを記入
（p15参照）
匂い容器を取り出し、テスト用シャーレを廃棄
使い終わった匂い容器は、ビーカーで洗う。ビーカーに無香料の食器用洗剤と水道水を入れ、容器を５〜１０分撹拌する。再使用するまで乾かす。

## Slide 15
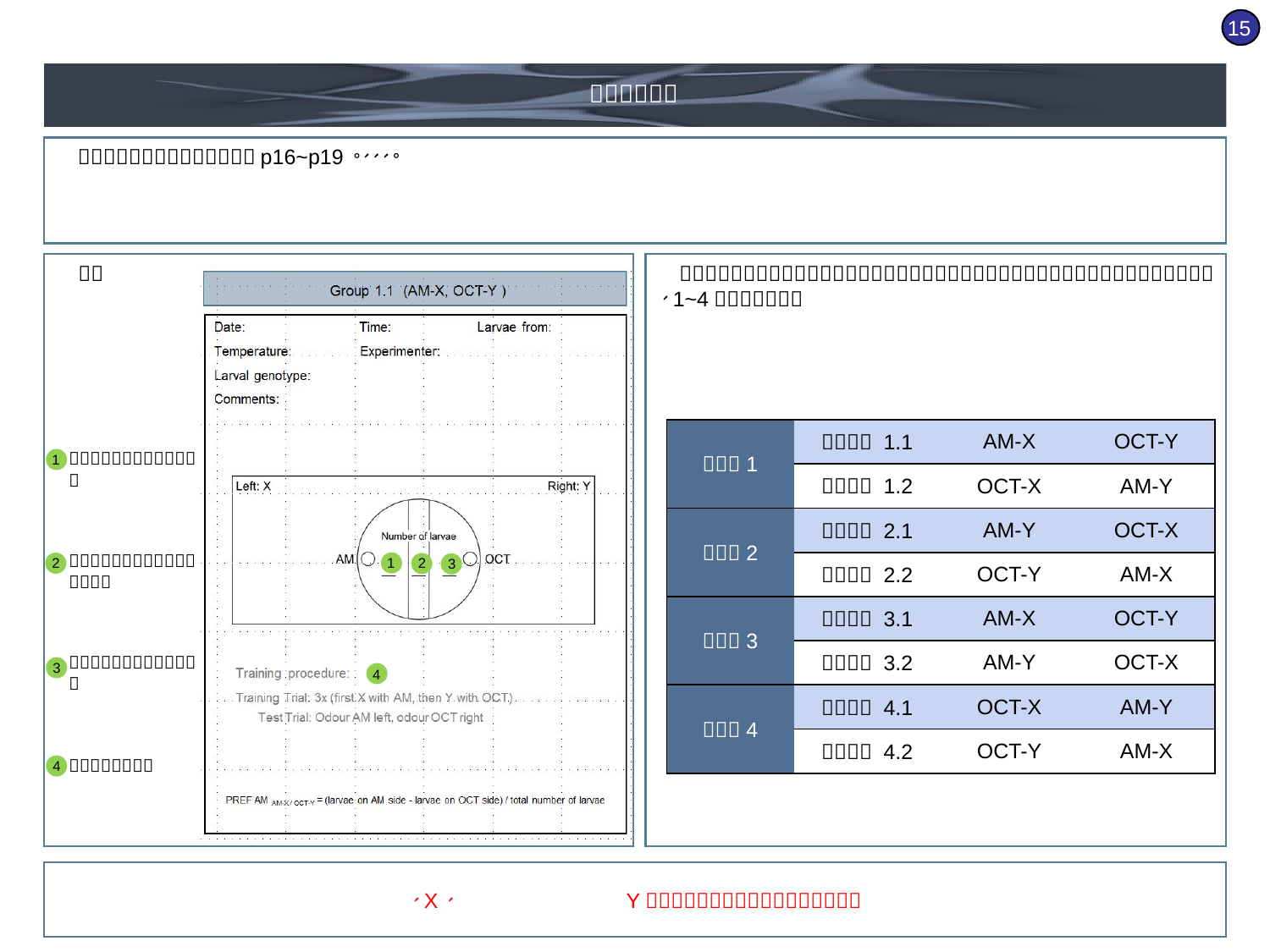

15
データシート
　次ページからのデータシート（p16~p19）は結果の記録用である。日付、時間、気温、実験者などの情報もデータシートに記録する。
 例：
　対応グループのペアは、トレーニング順序を入れ替えたものも含めて合計４種類である。そこで、これら４種類のデータシート（タイプ1~4）を次に示す。
| タイプ1 | グループ 1.1 | AM-X | OCT-Y |
| --- | --- | --- | --- |
| | グループ 1.2 | OCT-X | AM-Y |
| タイプ2 | グループ 2.1 | AM-Y | OCT-X |
| | グループ 2.2 | OCT-Y | AM-X |
| タイプ3 | グループ 3.1 | AM-X | OCT-Y |
| | グループ 3.2 | AM-Y | OCT-X |
| タイプ4 | グループ 4.1 | OCT-X | AM-Y |
| | グループ 4.2 | OCT-Y | AM-X |
左側の幼虫の数を数えて記入
1
中央ゾーンの幼虫の数を数えて記入
1
2
2
3
右側の幼虫の数を数えて記入
3
4
トレーニング手順
4
各データシート下部の式は、シャーレXをフルクトース入り、シャーレYをフルクトース無としたものである。

## Slide 16
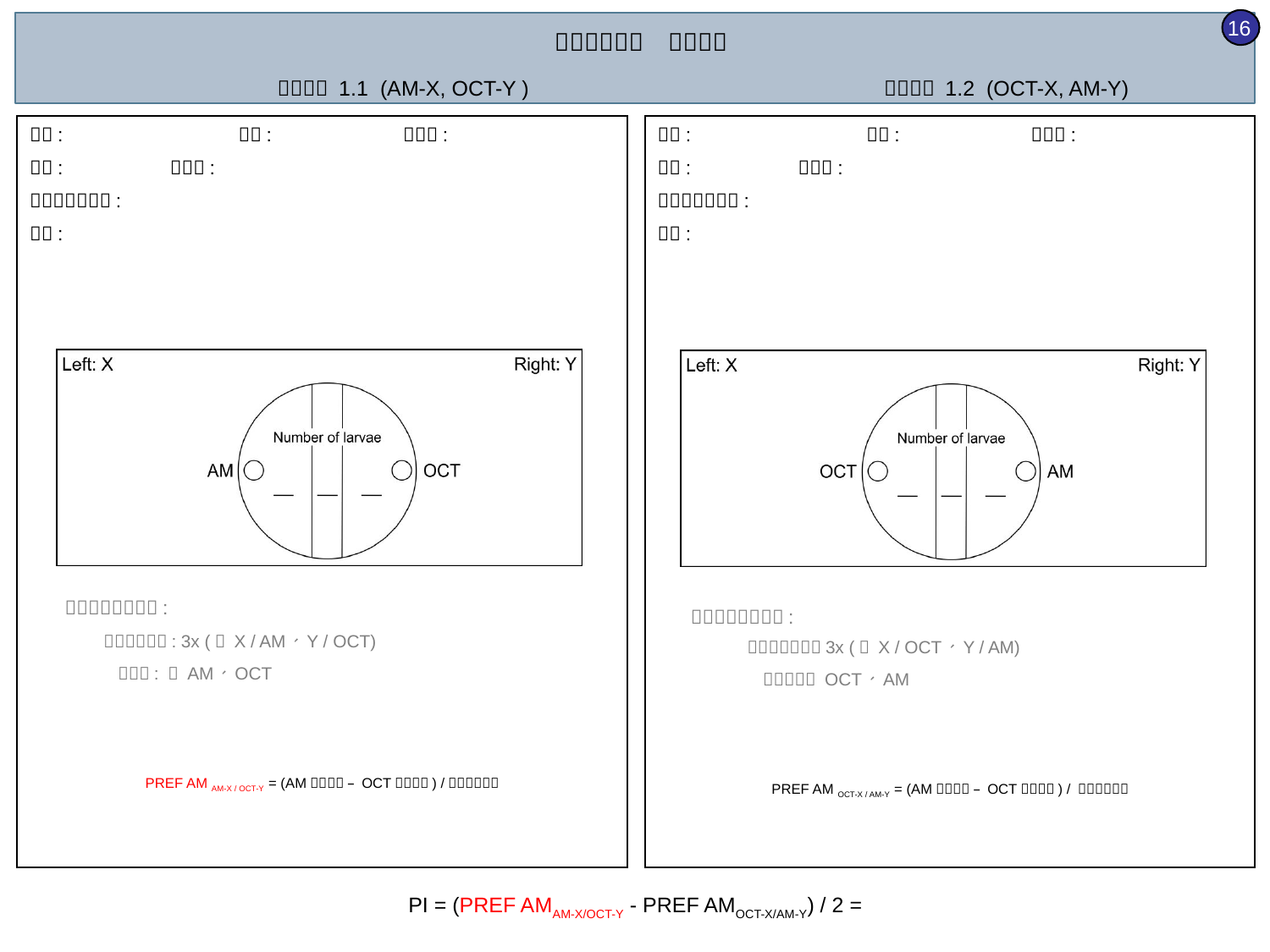

16
# データシート　タイプ１ 　　　　　　　　　　　 　　　　 　　　 グループ 1.1 (AM-X, OCT-Y ) グループ 1.2 (OCT-X, AM-Y)
日付: 時間: 産卵日:
気温: 	 実験者:
幼虫の遺伝子型:
備考:
 トレーニング手順:
 　　トレーニング: 3x (先 X / AM、後 Y / OCT)
 　　　　テスト: 左 AM、右 OCT
PREF AM AM-X / OCT-Y = (AM側の幼虫 – OCT側の幼虫) /幼虫の合計数
日付: 時間: 産卵日:
気温: 	 実験者:
幼虫の遺伝子型:
備考:
 トレーニング手順:
 　 　　トレーニング：3x (先 X / OCT、後 Y / AM)
　　　　　　テスト：左 OCT、右 AM
PREF AM OCT-X / AM-Y = (AM側の幼虫 – OCT側の幼虫) / 幼虫の合計数
PI = (PREF AMAM-X/OCT-Y - PREF AMOCT-X/AM-Y) / 2 =

## Slide 17
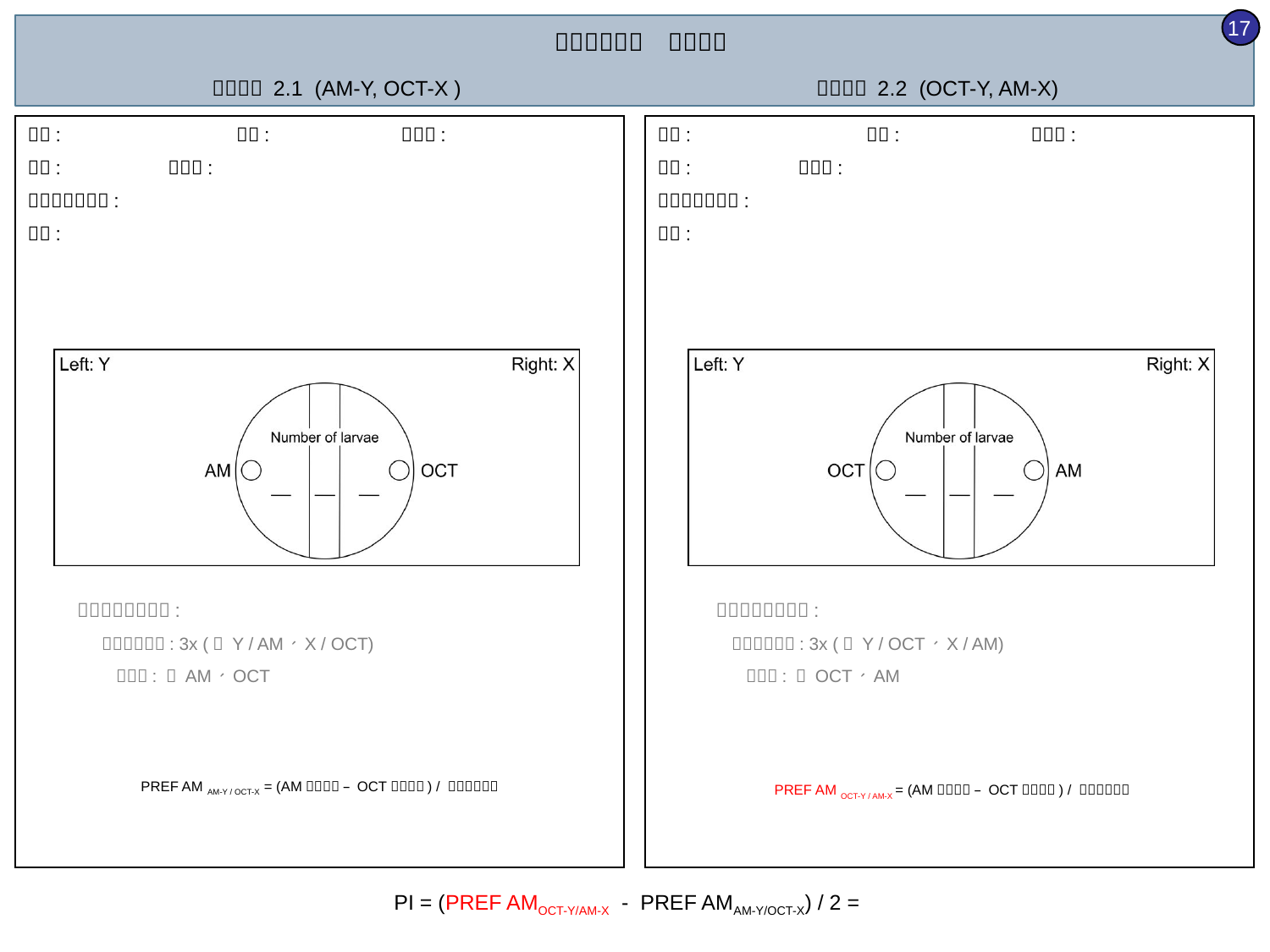

17
				 データシート　タイプ２
 グループ 2.1 (AM-Y, OCT-X ) グループ 2.2 (OCT-Y, AM-X)
日付: 時間: 産卵日:
気温: 	 実験者:
幼虫の遺伝子型:
備考:
 　トレーニング手順:
 　　トレーニング: 3x (先 Y / AM、後 X / OCT)
 　　　　テスト: 左 AM、右 OCT
PREF AM AM-Y / OCT-X = (AM側の幼虫 – OCT側の幼虫) / 幼虫の合計数
日付: 時間: 産卵日:
気温: 	 実験者:
幼虫の遺伝子型:
備考:
　　　トレーニング手順:
 　　トレーニング: 3x (先 Y / OCT、後 X / AM)
 　　　　テスト: 左 OCT、右 AM
 　　　　　　　 PREF AM OCT-Y / AM-X = (AM側の幼虫 – OCT側の幼虫) / 幼虫の合計数
PI = (PREF AMOCT-Y/AM-X - PREF AMAM-Y/OCT-X) / 2 =

## Slide 18
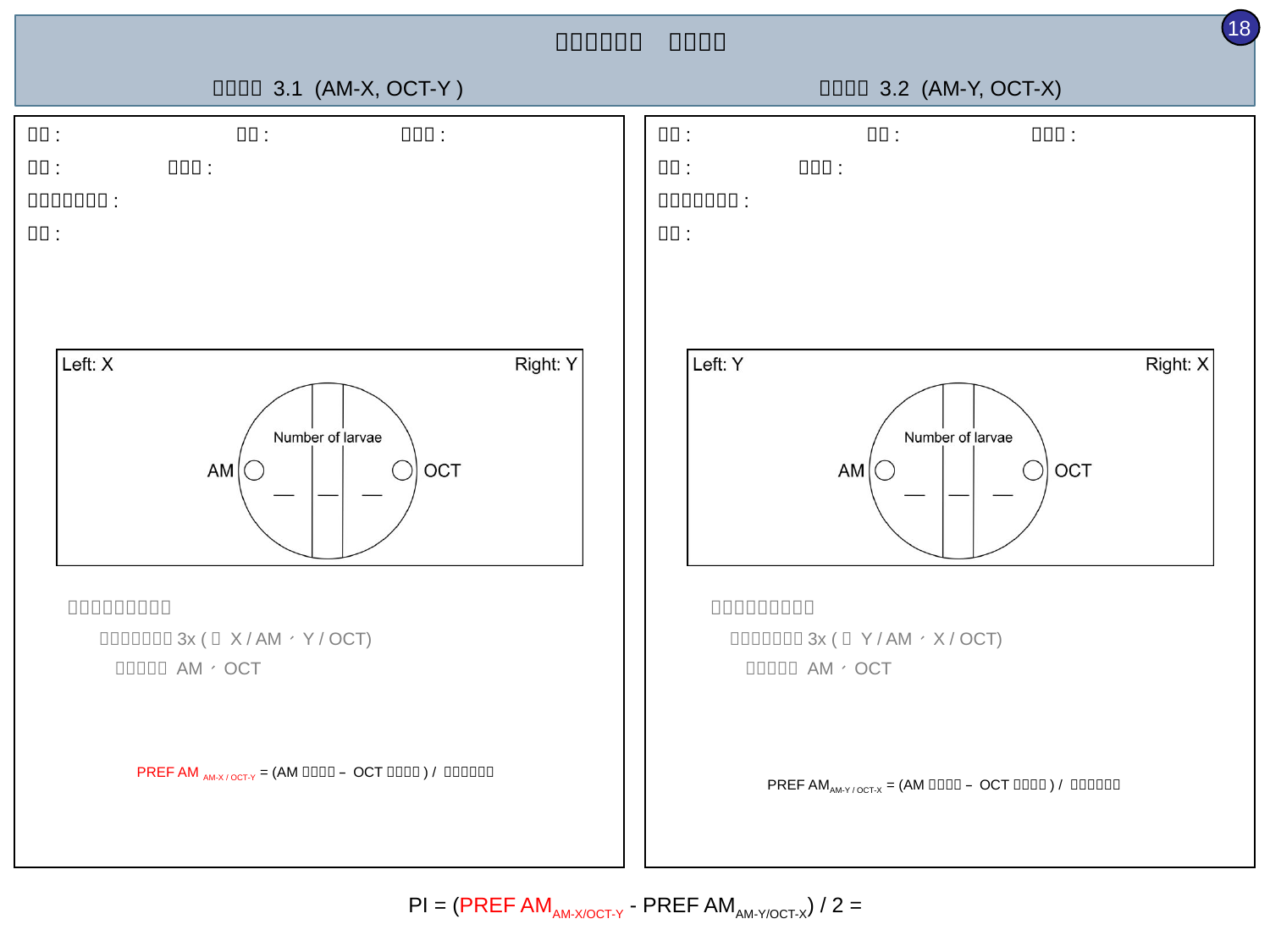

18
				 データシート　タイプ３
 グループ 3.1 (AM-X, OCT-Y ) グループ 3.2 (AM-Y, OCT-X)
日付: 時間: 産卵日:
気温: 	 実験者:
幼虫の遺伝子型:
備考:
 トレーニング手順：
 　　トレーニング：3x (先 X / AM、後 Y / OCT)
　　　　　テスト：左 AM、右 OCT
 　　　　　　 PREF AM AM-X / OCT-Y = (AM側の幼虫 – OCT側の幼虫) / 幼虫の合計数
日付: 時間: 産卵日:
気温: 	 実験者:
幼虫の遺伝子型:
備考:
　　　トレーニング手順：
 　　トレーニング：3x (先 Y / AM、後 X / OCT)
　　　　　テスト：左 AM、右 OCT
 　　　　　　PREF AMAM-Y / OCT-X = (AM側の幼虫 – OCT側の幼虫) / 幼虫の合計数
PI = (PREF AMAM-X/OCT-Y - PREF AMAM-Y/OCT-X) / 2 =

## Slide 19
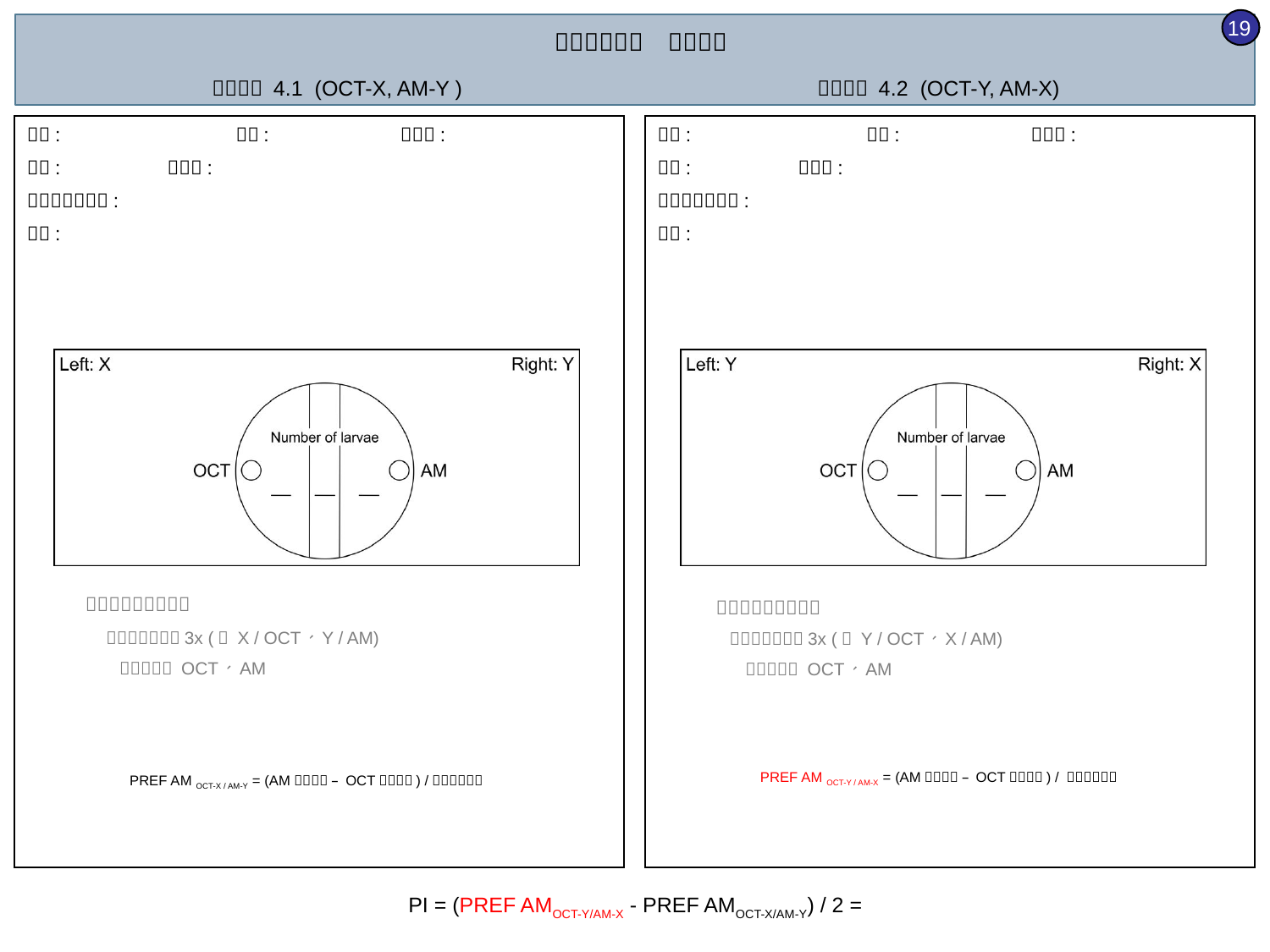

19
				 データシート　タイプ４
 グループ 4.1 (OCT-X, AM-Y ) グループ 4.2 (OCT-Y, AM-X)
日付: 時間: 産卵日:
気温: 	 実験者:
幼虫の遺伝子型:
備考:
　　　トレーニング手順：
 　　トレーニング：3x (先 X / OCT、後 Y / AM)
　　　　 　テスト：左 OCT、右 AM
　　　　　　　PREF AM OCT-X / AM-Y = (AM側の幼虫 – OCT側の幼虫) /幼虫の合計数
日付: 時間: 産卵日:
気温: 	 実験者:
幼虫の遺伝子型:
備考:
　　　トレーニング手順：
 　　トレーニング：3x (先 Y / OCT、後 X / AM)
　　　　　テスト：左 OCT、右 AM
　　　　　　　PREF AM OCT-Y / AM-X = (AM側の幼虫 – OCT側の幼虫) / 幼虫の合計数
PI = (PREF AMOCT-Y/AM-X - PREF AMOCT-X/AM-Y) / 2 =

## Slide 20
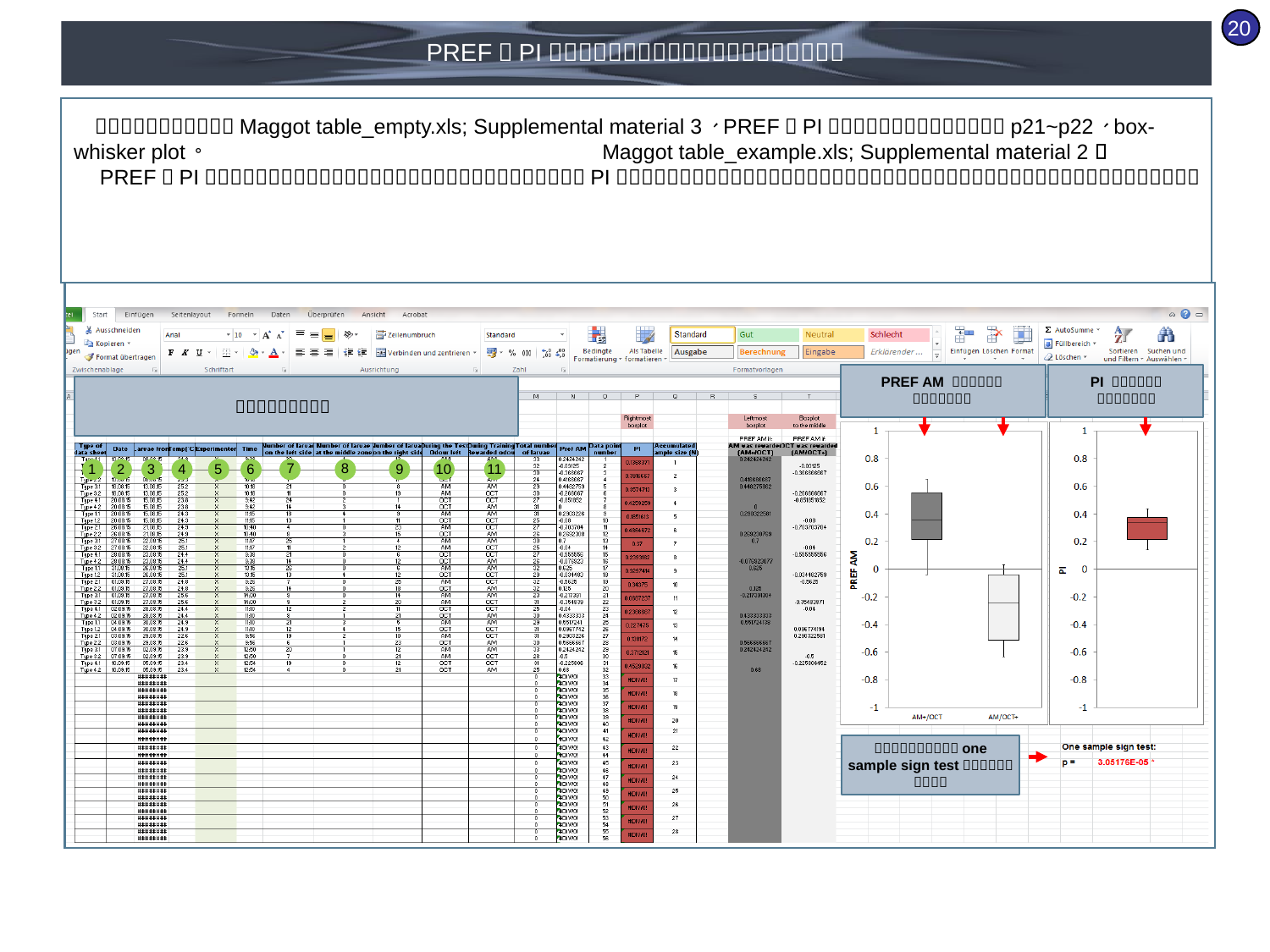

20
PREFとPI値の計算とグラフ化のためのエクセルシート
　付属のエクセルシート（Maggot table_empty.xls; Supplemental material 3）に生データを入力すると、PREFとPI値が自動的に計算され（詳細はp21~p22を参照）、結果が箱ひげ図（ボックスプロット；box-whisker plot）として可視化される。（表の入力例は付属のエクセルファイル参照：Maggot table_example.xls; Supplemental material 2）カラム１〜１１にデータを入力する。
　PREFとPI値の箱ひげ図が自動的にエクセルシート内に作成される。加えて、PI値より１サンプル符号検定（統計処理のページ参照）の結果が計算される（エクセルシートの右下）。
PREF AM の箱ひげ図が
ここに描かれる
PI の箱ひげ図が
ここに描かれる
入力が必要なカラム
7
8
3
4
10
11
2
5
6
9
1
１サンプル符号検定（one sample sign test）の結果が計算される

## Slide 21
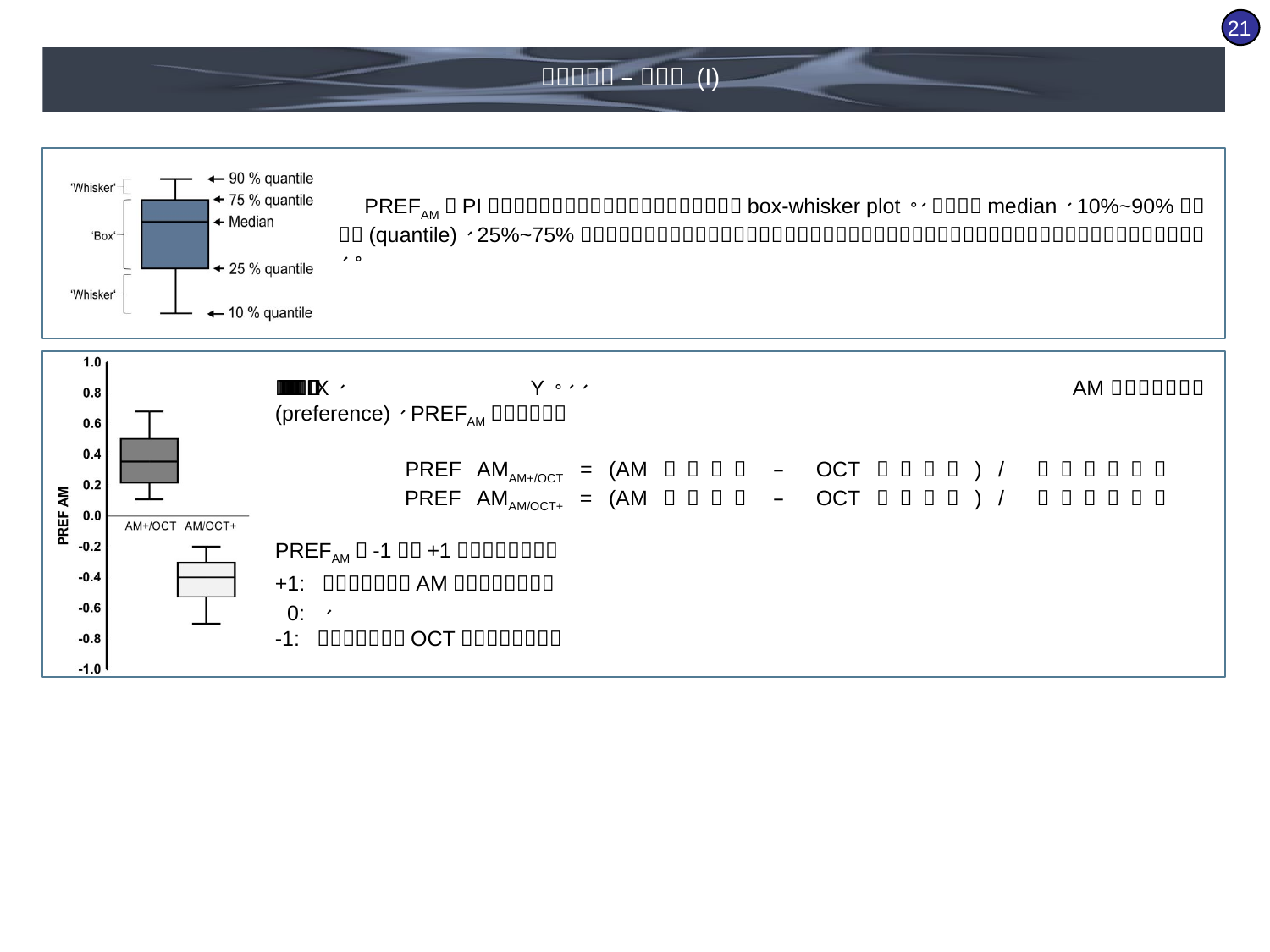

21
データ解析 – グラフ (I)
　PREFAMとPI値（詳細は後に記述）を算出し、箱ひげ図（box-whisker plot）として表示する。このグラフでは、 中央値（median）、10%~90%の分位点(quantile)、25%~75%の分位点が示される。このグラフの方が、平均値と標準誤差を示すグラフよりも適している。このグラフではデータが正規分布している必要がなく、またこの幼虫の実験データは基本的に正規分布を示さないためである。
次に示す例はタイプ３のデータシートに基づく。シャーレXをフルクトース添加、シャーレYをフルクトース無しのものとする。まず、２つの対応グループの結果から、それぞれAMに対する嗜好度(preference)、PREFAMを算出する：
 PREF AMAM+/OCT = (AM側の幼虫 – OCT側の幼虫) / 幼虫の合計数
 PREF AMAM/OCT+ = (AM側の幼虫 – OCT側の幼虫) / 幼虫の合計数
PREFAMは-1から+1までの値をとる：
+1: すべての幼虫がAMに誘引された場合
 0: 左右に同数の幼虫が別れた場合、もしくはすべての幼虫が中央ゾーンに留まった場合
-1: すべての幼虫がOCTに誘引された場合

## Slide 22
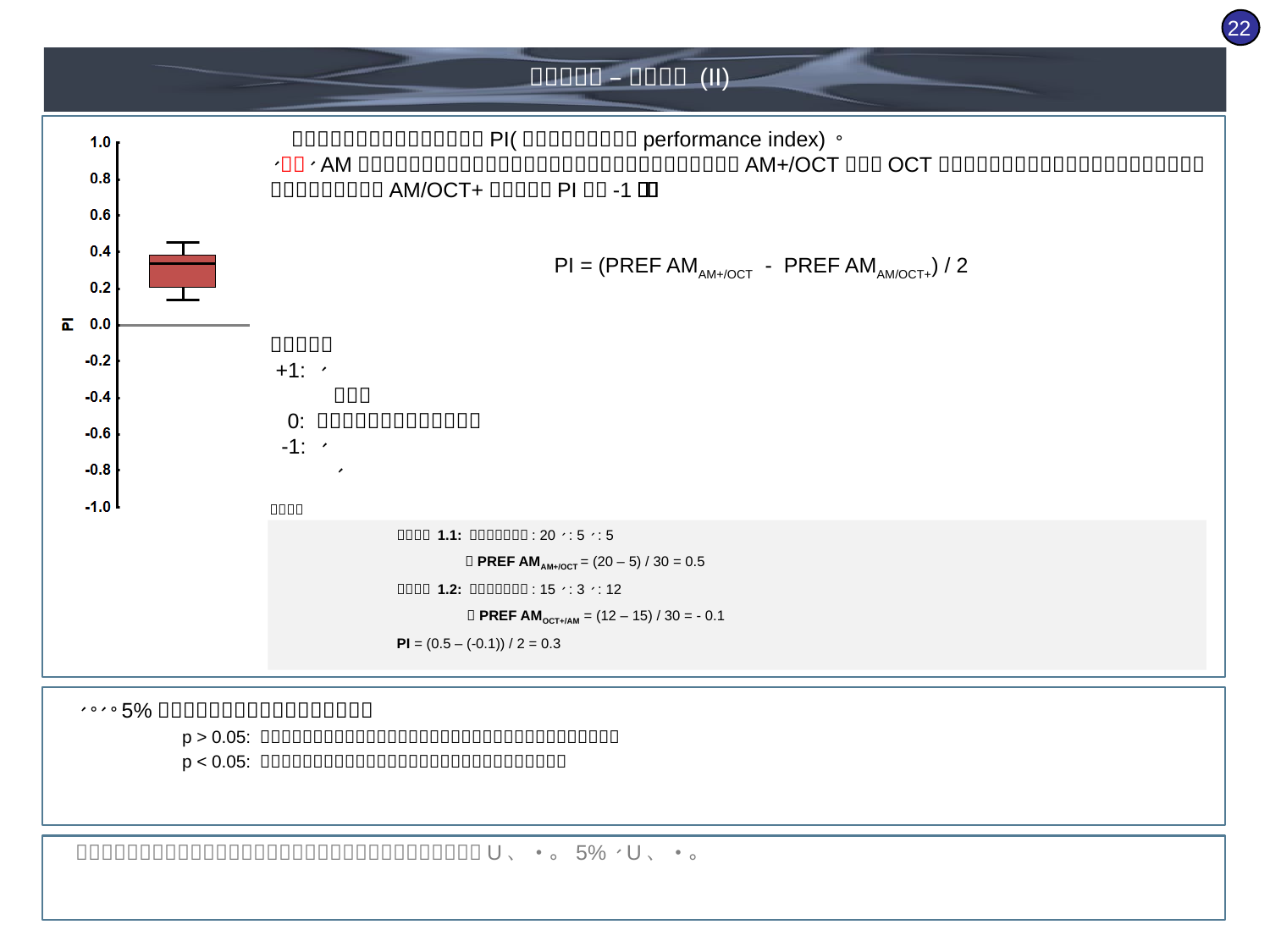

22
データ解析 – 統計処理 (II)
　対応グループ間の嗜好度の差からPI(パフォーマンス値；performance index)を算出する。フルクトースが報酬として働くことがわかっているので、正の値を得るために常に、AMを報酬と共に提示したグループの嗜好度（タイプ３を例とした場合AM+/OCT）からOCTを報酬と共に提示したグループの嗜好度（タイプ３を例とした場合AM/OCT+）を引く。PI値が-1から１までの値をとるよう、計算結果を２で割る：
 PI = (PREF AMAM+/OCT - PREF AMAM/OCT+) / 2
値の意味：
 +1: 連合記憶が形成され、すべての幼虫がトレーニングでフルクトースと共に提示された匂いに誘引
　　　された
  0: 連合記憶は形成されなかった
 -1: 嫌悪連合記憶が形成され、すべての幼虫が報酬と結びつけられた匂いを忌避した（糖濃度が高すぎ
　　　て、報酬ではなく罰として働いた可能性が考えられる）
計算例：
	グループ 1.1: 左側の幼虫の数: 20、中央ゾーンの幼虫の数: 5、右側の幼虫の数: 5
	  PREF AMAM+/OCT = (20 – 5) / 30 = 0.5
	グループ 1.2: 左側の幼虫の数: 15、中央ゾーンの幼虫の数: 3、右側の幼虫の数: 12
  PREF AMOCT+/AM = (12 – 15) / 30 = - 0.1
	PI = (0.5 – (-0.1)) / 2 = 0.3
　先に述べた通り、このデータは正規分布である必要はない。箱ひげ図で用いた中央値と分位値の統計学的観点から見ても、ノンパラメトリックな統計手法を用いるべきである。誤差閾を5%として１サンプル符号検定を用いる。
	p > 0.05: データの値は０と有意差が無い、つまり連合記憶が形成されたとは言えない
	p < 0.05: データの値は有意に０と異なる、つまり幼虫は連合記憶を示した
　２つのグループを統計学的に比較するためには、マン・ホイットニーのU検定を用いるが、３つ以上のグループを比較する場合には事前にクラスカル・ウォリス検定が必要である。誤差閾を5%に保つために、U検定の後にはボンフェローニの調整、もしくはボンフェローニ・ホルムの調整をかけることが望ましい。

## Slide 23
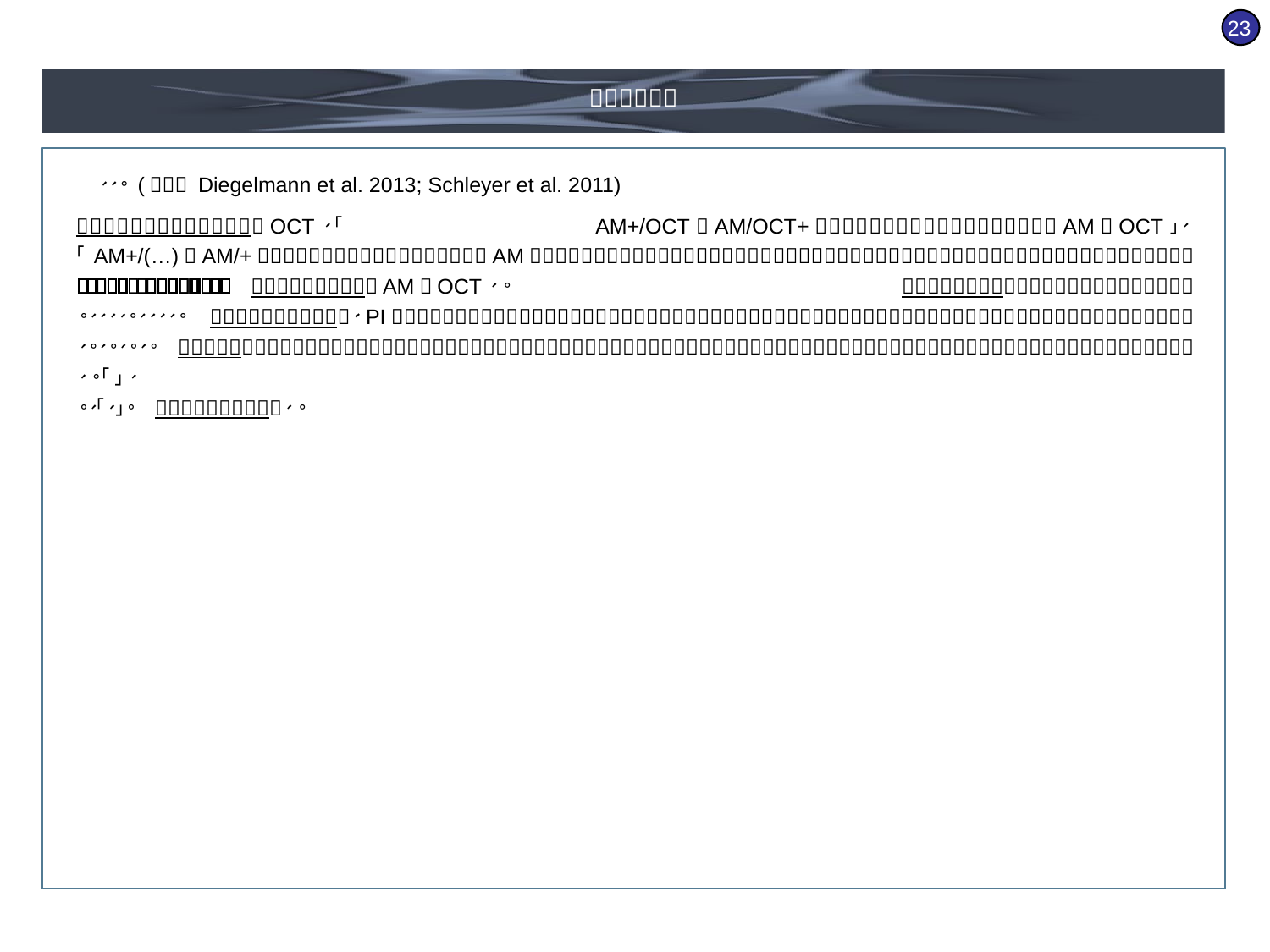

23
テーマの応用
　ここに述べた標準的な実験手法をもとにして、ショウジョウバエ幼虫の記憶学習の多様な側面を研究するために、様々な応用的な実験が可能である。 (参照： Diegelmann et al. 2013; Schleyer et al. 2011)
匂い１種を用いたパラダイム：OCTを実験に使わない場合、元のパラダイムの「AM+/OCTとAM/OCT+という対応グループでのトレーニングとAM対OCTの選択テスト」は、「AM+/(…)とAM/+という対応グループでのトレーニングとAMの嗜好性テスト」に変わる。言い換えると、一方のグループの幼虫は「匂いと報酬の対応」を学習するが、他方のグループの幼虫は「匂いと報酬の非対応」を学習することになる。このパラダイムによって、単一の匂いを学習できるか調べることができ、また「一般化」の実験にも用いられる。一般化の実験には、トレーニングとテストで異なる匂いを用いる。それによって、幼虫にとってトレーニング時の匂いとテスト時の匂いがどれほど似通っているかを知ることができる。もしくは、テスト時にトレーニング時よりも高濃度か低濃度の同じ匂いを用いた場合、幼虫がそれらを同じものとして受け取るかどうかを調べることも可能である。　匂いの種類を変える：AMとOCT以外の様々な匂いについて、学習可能であるか調べることができる。　別の報酬を用いる：フルクトースの代わりに別の報酬を試してもよい。例えば、別の糖、人工甘味料、アミノ酸、塩など。さらには、光、温度、培地の粘性や質感、湿度など味覚以外の刺激を用いることも可能である。　報酬をテスト中に提示：報酬をテスト用シャーレに加えると、PI値は０に下がる。これは、匂いに対する学習行動を、報酬の探索行動であると捉えるとわかりやすい。つまり、幼虫は一旦匂いと報酬の結びつきを学習すると、その匂いの元に報酬を求めて寄って行くのである。このように学習された探索行動は、求める物質の存在下ではもはや必要ない。このパラダイムを用いれば、幼虫が実際に何を探しているのかを知ることができる。ある報酬でトレーニングした後、別の物質をテスト用シャーレに入れて実験してみるとよい。　味覚罰学習：高濃度の塩、キニーネや他の苦味物質などの忌避性の味物質や、または味覚以外の刺激を罰として試してみるのもよい。ただし、高濃度の塩やキニーネをトレーニングに用いた場合、テスト用のシャーレにも同様の忌避物質を添加する必要がある。学習された「安全な」匂いへの逃避行動は、実際に逃げるべき状況にある時にしか引き出されないためであると考えられる。（私たちに置き換えて考えても、「非常事態が起きない限り、誰も非常口に向かって走らない」のと同じである。）　ビデオトラッキング：ビデオ撮影により幼虫の動きを追跡（トラッキング）し解析すれば、ただ数をカウントするだけの実験よりも詳細に幼虫の行動を研究することができる。

## Slide 24
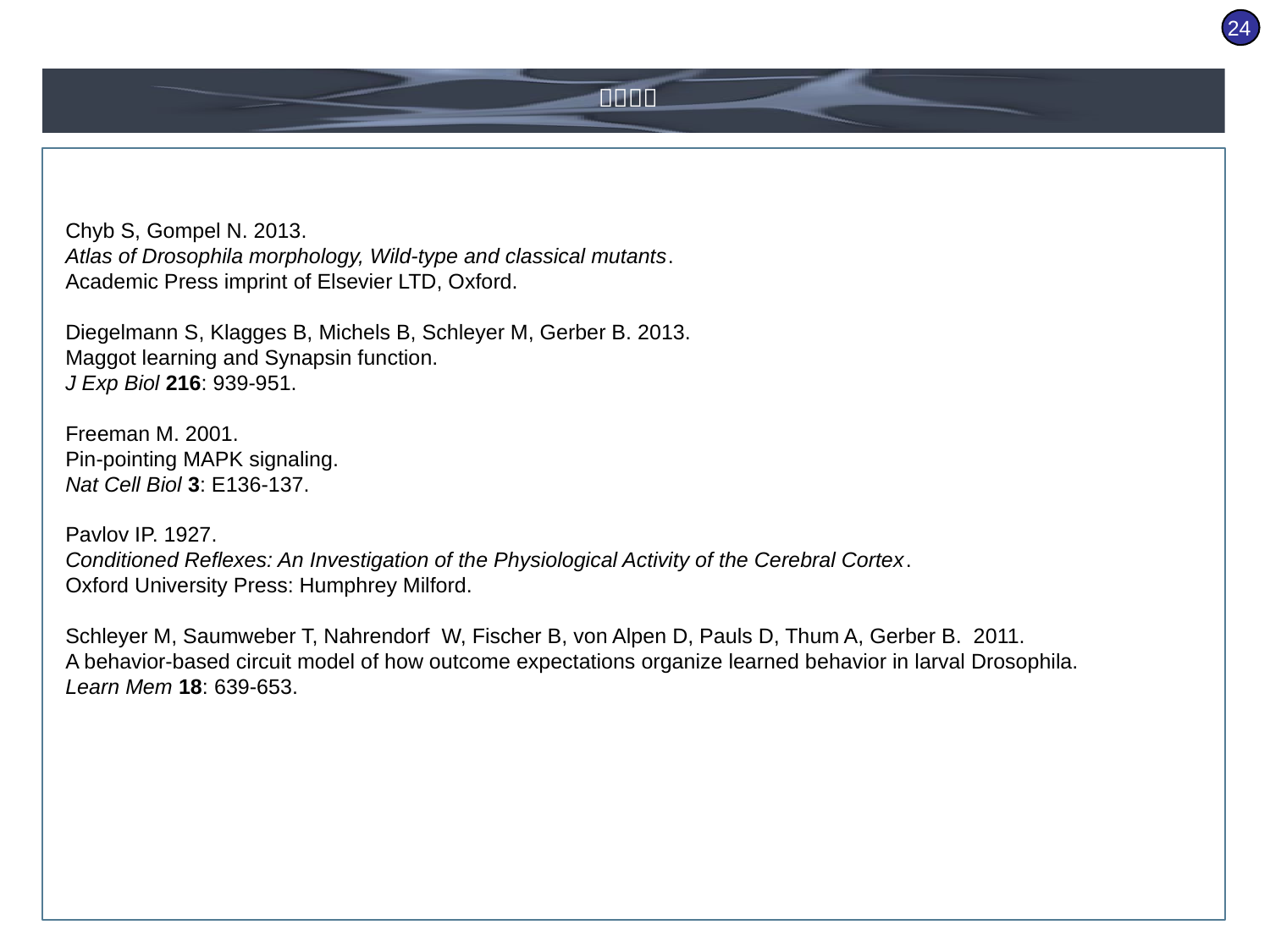

24
参考論文
Chyb S, Gompel N. 2013.
Atlas of Drosophila morphology, Wild-type and classical mutants.
Academic Press imprint of Elsevier LTD, Oxford.
Diegelmann S, Klagges B, Michels B, Schleyer M, Gerber B. 2013.
Maggot learning and Synapsin function.
J Exp Biol 216: 939-951.
Freeman M. 2001.
Pin-pointing MAPK signaling.
Nat Cell Biol 3: E136-137.
Pavlov IP. 1927.
Conditioned Reflexes: An Investigation of the Physiological Activity of the Cerebral Cortex.
Oxford University Press: Humphrey Milford.
Schleyer M, Saumweber T, Nahrendorf W, Fischer B, von Alpen D, Pauls D, Thum A, Gerber B. 2011.
A behavior-based circuit model of how outcome expectations organize learned behavior in larval Drosophila.
Learn Mem 18: 639-653.
