# Supplementary material for: Pavlovian Conditioning of Larval Drosophila: An Illustrated, Multilingual, Hands-On Manual for Odor-Taste Associative Learning in Maggots
Source: Front Behav Neurosci. 2017 Apr 19;11:45. doi: 10.3389/fnbeh.2017.00045 (PMC5395560; doi:10.3389/fnbeh.2017.00045)
Supplement: Supplemental Materials 11–13 — A manual for odor-reward learning in larval Drosophila (Supplemental Material 11), example of a table for data analysis (Supplemental Material 12), and an empty table for entering and analyzing one's own data (Supplemental Material 13), in the Spanish language. Versions of this manual in the English, German, French, Japanese, and Italian languages can be found in Supplemental Materials 1–3, 4–6, 7–9, 10, 14–16, respectively. [file SupplementalMaterial11.pptx]

## Slide 1
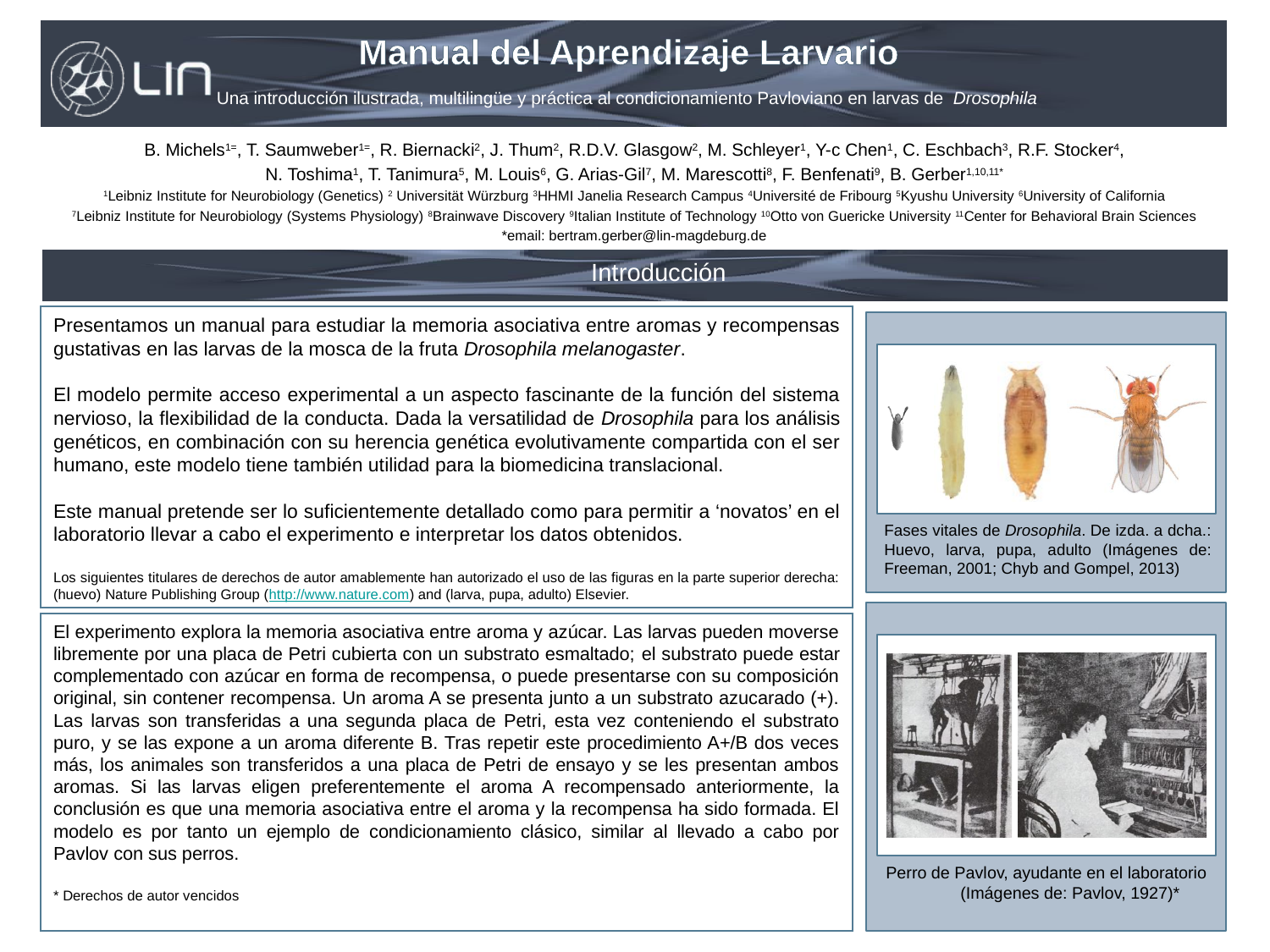

# Una introducción ilustrada, multilingüe y práctica al condicionamiento Pavloviano en larvas de Drosophila
Manual del Aprendizaje Larvario
B. Michels1=, T. Saumweber1=, R. Biernacki2, J. Thum2, R.D.V. Glasgow2, M. Schleyer1, Y-c Chen1, C. Eschbach3, R.F. Stocker4,
N. Toshima1, T. Tanimura5, M. Louis6, G. Arias-Gil7, M. Marescotti8, F. Benfenati9, B. Gerber1,10,11*
1Leibniz Institute for Neurobiology (Genetics) 2 Universität Würzburg 3HHMI Janelia Research Campus 4Université de Fribourg 5Kyushu University 6University of California
7Leibniz Institute for Neurobiology (Systems Physiology) 8Brainwave Discovery 9Italian Institute of Technology 10Otto von Guericke University 11Center for Behavioral Brain Sciences
*email: bertram.gerber@lin-magdeburg.de
Introducción
Presentamos un manual para estudiar la memoria asociativa entre aromas y recompensas gustativas en las larvas de la mosca de la fruta Drosophila melanogaster.
El modelo permite acceso experimental a un aspecto fascinante de la función del sistema nervioso, la flexibilidad de la conducta. Dada la versatilidad de Drosophila para los análisis genéticos, en combinación con su herencia genética evolutivamente compartida con el ser humano, este modelo tiene también utilidad para la biomedicina translacional.
Este manual pretende ser lo suficientemente detallado como para permitir a ‘novatos’ en el laboratorio llevar a cabo el experimento e interpretar los datos obtenidos.
Los siguientes titulares de derechos de autor amablemente han autorizado el uso de las figuras en la parte superior derecha: (huevo) Nature Publishing Group (http://www.nature.com) and (larva, pupa, adulto) Elsevier.
Fases vitales de Drosophila. De izda. a dcha.: Huevo, larva, pupa, adulto (Imágenes de: Freeman, 2001; Chyb and Gompel, 2013)
El experimento explora la memoria asociativa entre aroma y azúcar. Las larvas pueden moverse libremente por una placa de Petri cubierta con un substrato esmaltado; el substrato puede estar complementado con azúcar en forma de recompensa, o puede presentarse con su composición original, sin contener recompensa. Un aroma A se presenta junto a un substrato azucarado (+). Las larvas son transferidas a una segunda placa de Petri, esta vez conteniendo el substrato puro, y se las expone a un aroma diferente B. Tras repetir este procedimiento A+/B dos veces más, los animales son transferidos a una placa de Petri de ensayo y se les presentan ambos aromas. Si las larvas eligen preferentemente el aroma A recompensado anteriormente, la conclusión es que una memoria asociativa entre el aroma y la recompensa ha sido formada. El modelo es por tanto un ejemplo de condicionamiento clásico, similar al llevado a cabo por Pavlov con sus perros.
* Derechos de autor vencidos
Perro de Pavlov, ayudante en el laboratorio (Imágenes de: Pavlov, 1927)*

## Slide 2
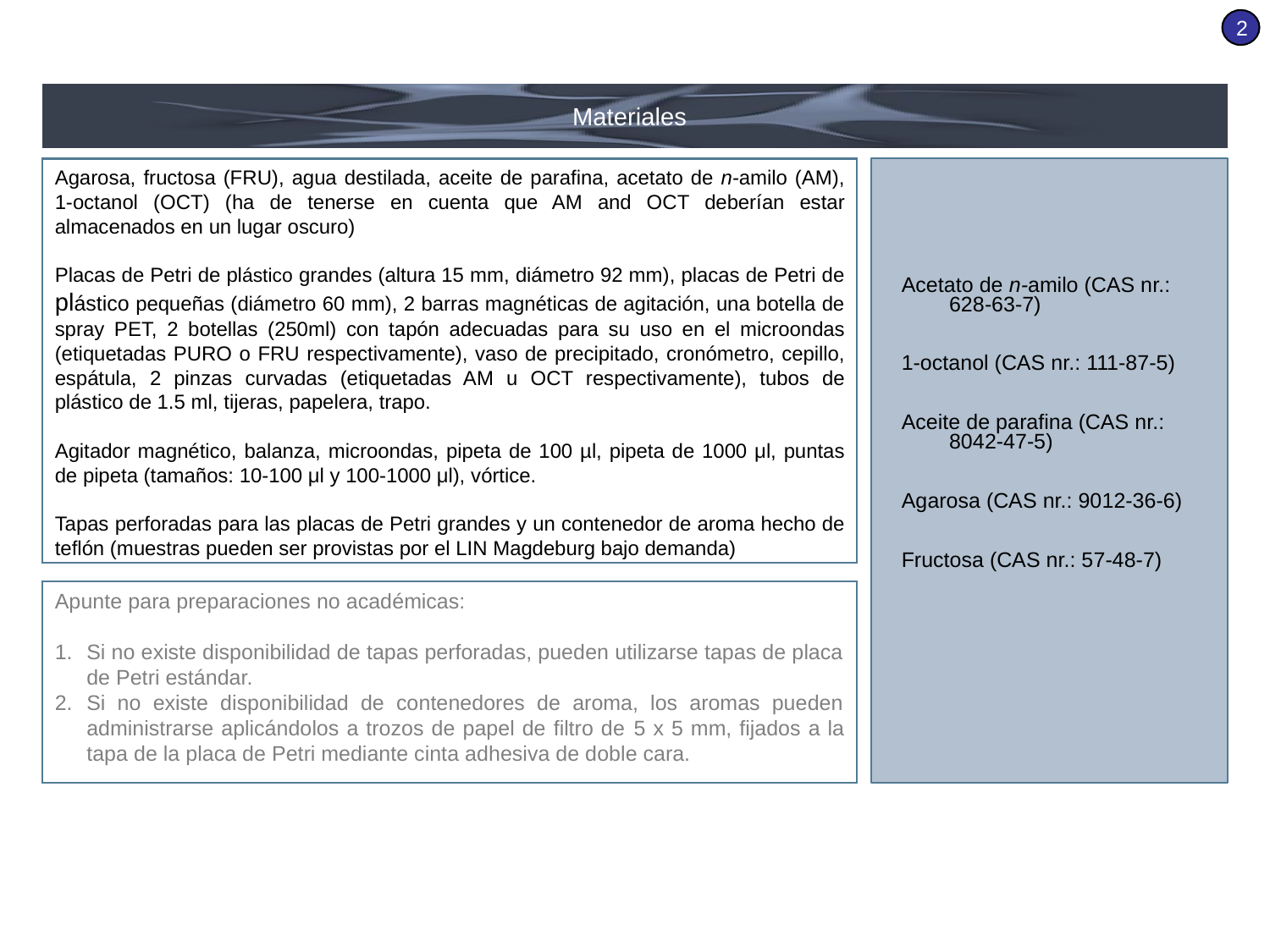

2
Materiales
Agarosa, fructosa (FRU), agua destilada, aceite de parafina, acetato de n-amilo (AM), 1-octanol (OCT) (ha de tenerse en cuenta que AM and OCT deberían estar almacenados en un lugar oscuro)
Placas de Petri de plástico grandes (altura 15 mm, diámetro 92 mm), placas de Petri de plástico pequeñas (diámetro 60 mm), 2 barras magnéticas de agitación, una botella de spray PET, 2 botellas (250ml) con tapón adecuadas para su uso en el microondas (etiquetadas PURO o FRU respectivamente), vaso de precipitado, cronómetro, cepillo, espátula, 2 pinzas curvadas (etiquetadas AM u OCT respectivamente), tubos de plástico de 1.5 ml, tijeras, papelera, trapo.
Agitador magnético, balanza, microondas, pipeta de 100 µl, pipeta de 1000 μl, puntas de pipeta (tamaños: 10-100 μl y 100-1000 μl), vórtice.
Tapas perforadas para las placas de Petri grandes y un contenedor de aroma hecho de teflón (muestras pueden ser provistas por el LIN Magdeburg bajo demanda)
Acetato de n-amilo (CAS nr.: 628-63-7)
1-octanol (CAS nr.: 111-87-5)
Aceite de parafina (CAS nr.: 8042-47-5)
Agarosa (CAS nr.: 9012-36-6)
Fructosa (CAS nr.: 57-48-7)
Apunte para preparaciones no académicas:
Si no existe disponibilidad de tapas perforadas, pueden utilizarse tapas de placa de Petri estándar.
Si no existe disponibilidad de contenedores de aroma, los aromas pueden administrarse aplicándolos a trozos de papel de filtro de 5 x 5 mm, fijados a la tapa de la placa de Petri mediante cinta adhesiva de doble cara.

## Slide 3
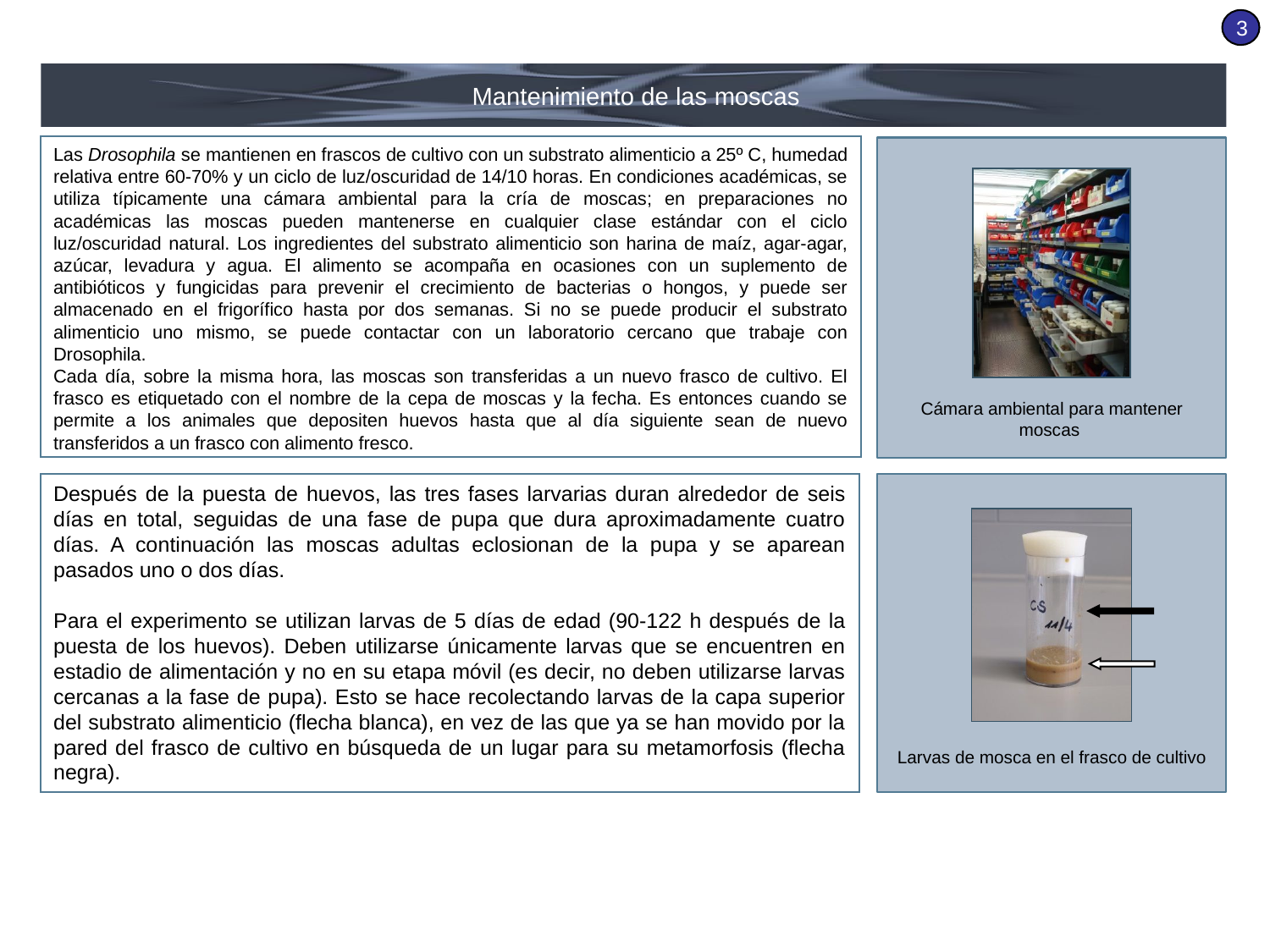

3
# Mantenimiento de las moscas
Las Drosophila se mantienen en frascos de cultivo con un substrato alimenticio a 25º C, humedad relativa entre 60-70% y un ciclo de luz/oscuridad de 14/10 horas. En condiciones académicas, se utiliza típicamente una cámara ambiental para la cría de moscas; en preparaciones no académicas las moscas pueden mantenerse en cualquier clase estándar con el ciclo luz/oscuridad natural. Los ingredientes del substrato alimenticio son harina de maíz, agar-agar, azúcar, levadura y agua. El alimento se acompaña en ocasiones con un suplemento de antibióticos y fungicidas para prevenir el crecimiento de bacterias o hongos, y puede ser almacenado en el frigorífico hasta por dos semanas. Si no se puede producir el substrato alimenticio uno mismo, se puede contactar con un laboratorio cercano que trabaje con Drosophila.
Cada día, sobre la misma hora, las moscas son transferidas a un nuevo frasco de cultivo. El frasco es etiquetado con el nombre de la cepa de moscas y la fecha. Es entonces cuando se permite a los animales que depositen huevos hasta que al día siguiente sean de nuevo transferidos a un frasco con alimento fresco.
Cámara ambiental para mantener moscas
Después de la puesta de huevos, las tres fases larvarias duran alrededor de seis días en total, seguidas de una fase de pupa que dura aproximadamente cuatro días. A continuación las moscas adultas eclosionan de la pupa y se aparean pasados uno o dos días.
Para el experimento se utilizan larvas de 5 días de edad (90-122 h después de la puesta de los huevos). Deben utilizarse únicamente larvas que se encuentren en estadio de alimentación y no en su etapa móvil (es decir, no deben utilizarse larvas cercanas a la fase de pupa). Esto se hace recolectando larvas de la capa superior del substrato alimenticio (flecha blanca), en vez de las que ya se han movido por la pared del frasco de cultivo en búsqueda de un lugar para su metamorfosis (flecha negra).
Larvas de mosca en el frasco de cultivo

## Slide 4
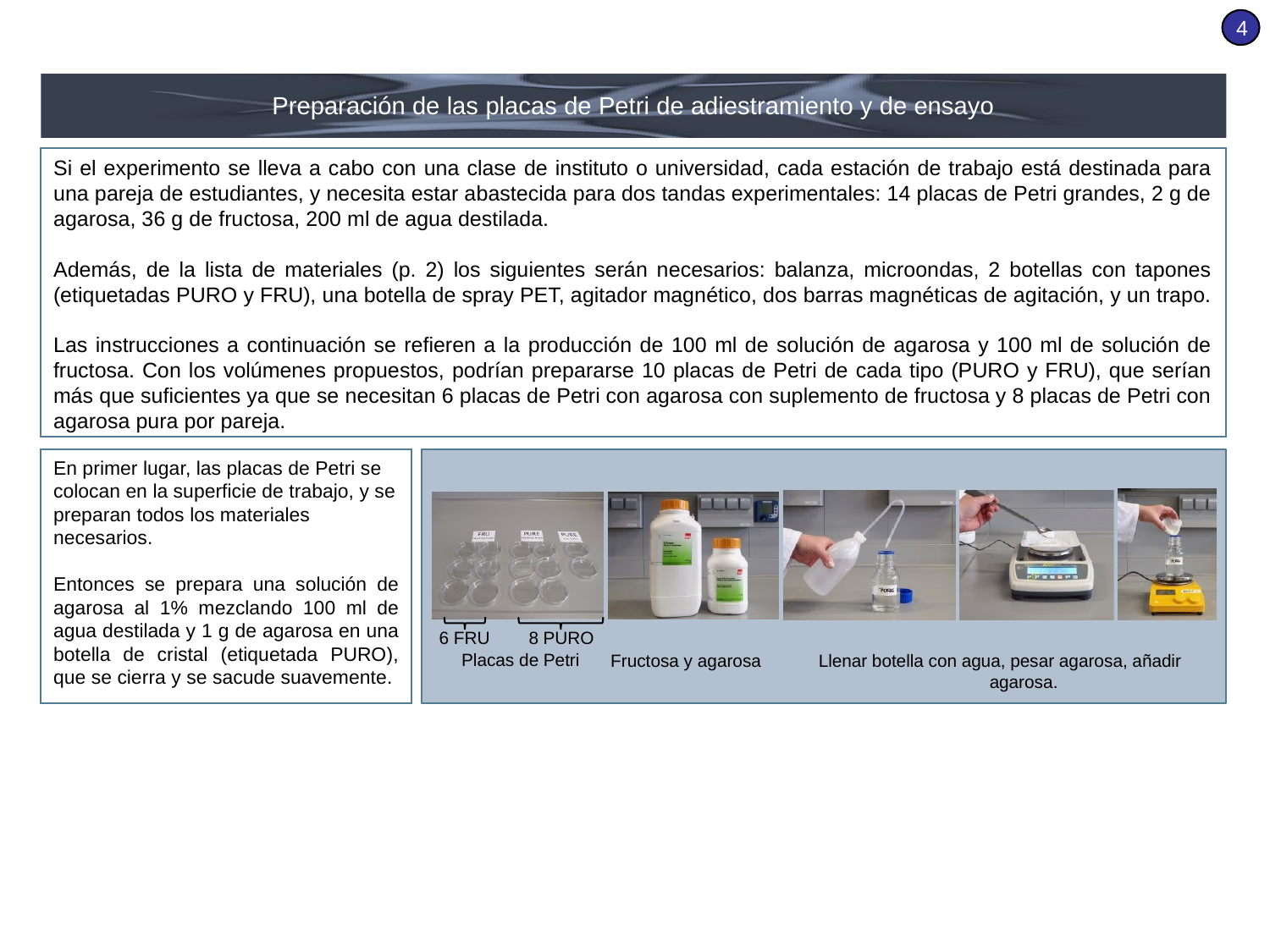

4
# Preparación de las placas de Petri de adiestramiento y de ensayo
Si el experimento se lleva a cabo con una clase de instituto o universidad, cada estación de trabajo está destinada para una pareja de estudiantes, y necesita estar abastecida para dos tandas experimentales: 14 placas de Petri grandes, 2 g de agarosa, 36 g de fructosa, 200 ml de agua destilada.
Además, de la lista de materiales (p. 2) los siguientes serán necesarios: balanza, microondas, 2 botellas con tapones (etiquetadas PURO y FRU), una botella de spray PET, agitador magnético, dos barras magnéticas de agitación, y un trapo.
Las instrucciones a continuación se refieren a la producción de 100 ml de solución de agarosa y 100 ml de solución de fructosa. Con los volúmenes propuestos, podrían prepararse 10 placas de Petri de cada tipo (PURO y FRU), que serían más que suficientes ya que se necesitan 6 placas de Petri con agarosa con suplemento de fructosa y 8 placas de Petri con agarosa pura por pareja.
En primer lugar, las placas de Petri se colocan en la superficie de trabajo, y se preparan todos los materiales necesarios.
Entonces se prepara una solución de agarosa al 1% mezclando 100 ml de agua destilada y 1 g de agarosa en una botella de cristal (etiquetada PURO), que se cierra y se sacude suavemente.
6 FRU 8 PURO
Fructosa y agarosa
Placas de Petri
Llenar botella con agua, pesar agarosa, añadir agarosa.

## Slide 5
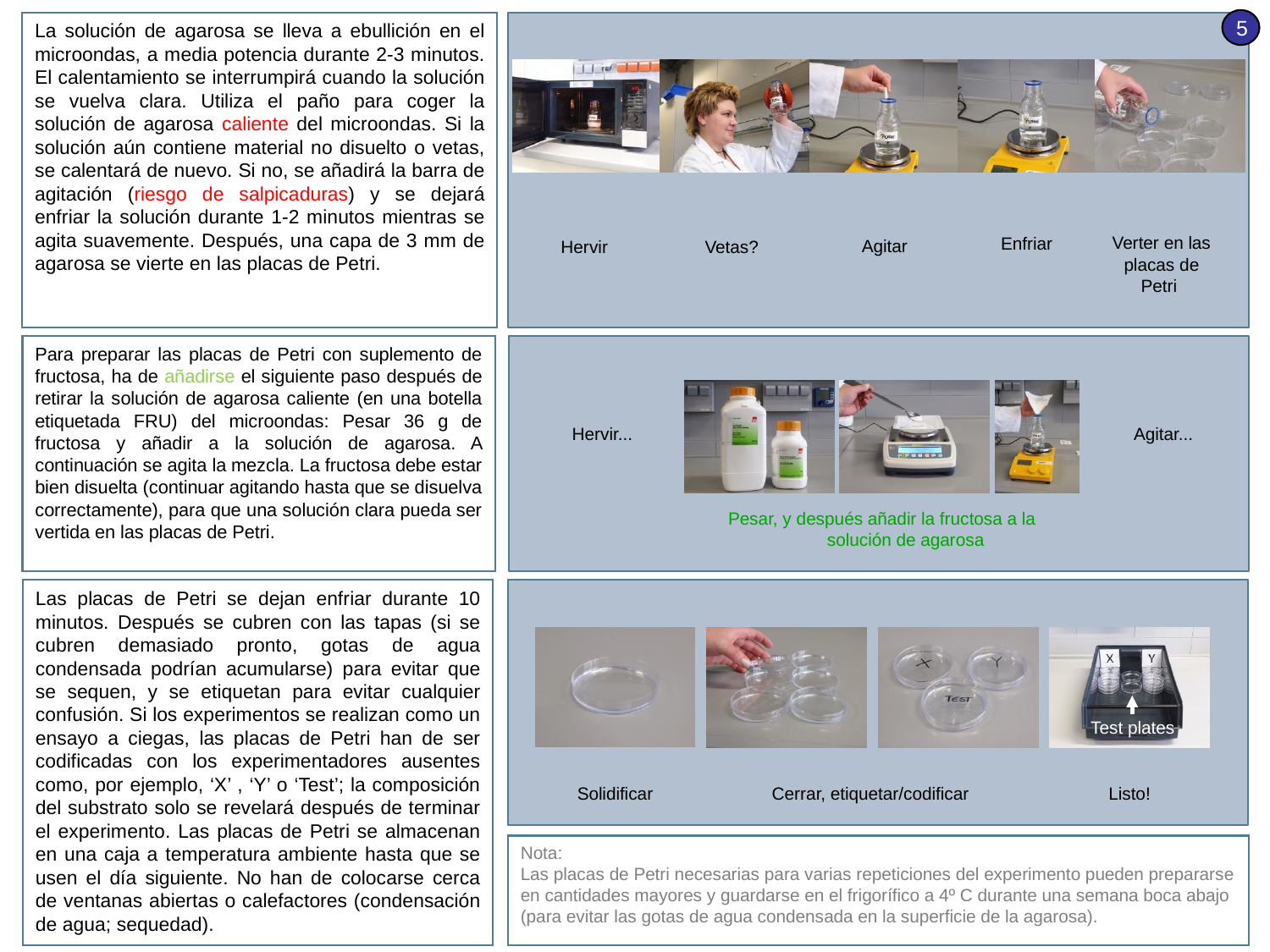

5
La solución de agarosa se lleva a ebullición en el microondas, a media potencia durante 2-3 minutos. El calentamiento se interrumpirá cuando la solución se vuelva clara. Utiliza el paño para coger la solución de agarosa caliente del microondas. Si la solución aún contiene material no disuelto o vetas, se calentará de nuevo. Si no, se añadirá la barra de agitación (riesgo de salpicaduras) y se dejará enfriar la solución durante 1-2 minutos mientras se agita suavemente. Después, una capa de 3 mm de agarosa se vierte en las placas de Petri.
Verter en las placas de Petri
Vetas?
Enfriar
Agitar
Hervir
Para preparar las placas de Petri con suplemento de fructosa, ha de añadirse el siguiente paso después de retirar la solución de agarosa caliente (en una botella etiquetada FRU) del microondas: Pesar 36 g de fructosa y añadir a la solución de agarosa. A continuación se agita la mezcla. La fructosa debe estar bien disuelta (continuar agitando hasta que se disuelva correctamente), para que una solución clara pueda ser vertida en las placas de Petri.
Hervir...
Agitar...
Pesar, y después añadir la fructosa a la solución de agarosa
Las placas de Petri se dejan enfriar durante 10 minutos. Después se cubren con las tapas (si se cubren demasiado pronto, gotas de agua condensada podrían acumularse) para evitar que se sequen, y se etiquetan para evitar cualquier confusión. Si los experimentos se realizan como un ensayo a ciegas, las placas de Petri han de ser codificadas con los experimentadores ausentes como, por ejemplo, ‘X’ , ‘Y’ o ‘Test’; la composición del substrato solo se revelará después de terminar el experimento. Las placas de Petri se almacenan en una caja a temperatura ambiente hasta que se usen el día siguiente. No han de colocarse cerca de ventanas abiertas o calefactores (condensación de agua; sequedad).
Test plates
Solidificar
Cerrar, etiquetar/codificar
Listo!
Nota:
Las placas de Petri necesarias para varias repeticiones del experimento pueden prepararse en cantidades mayores y guardarse en el frigorífico a 4º C durante una semana boca abajo (para evitar las gotas de agua condensada en la superficie de la agarosa).

## Slide 6
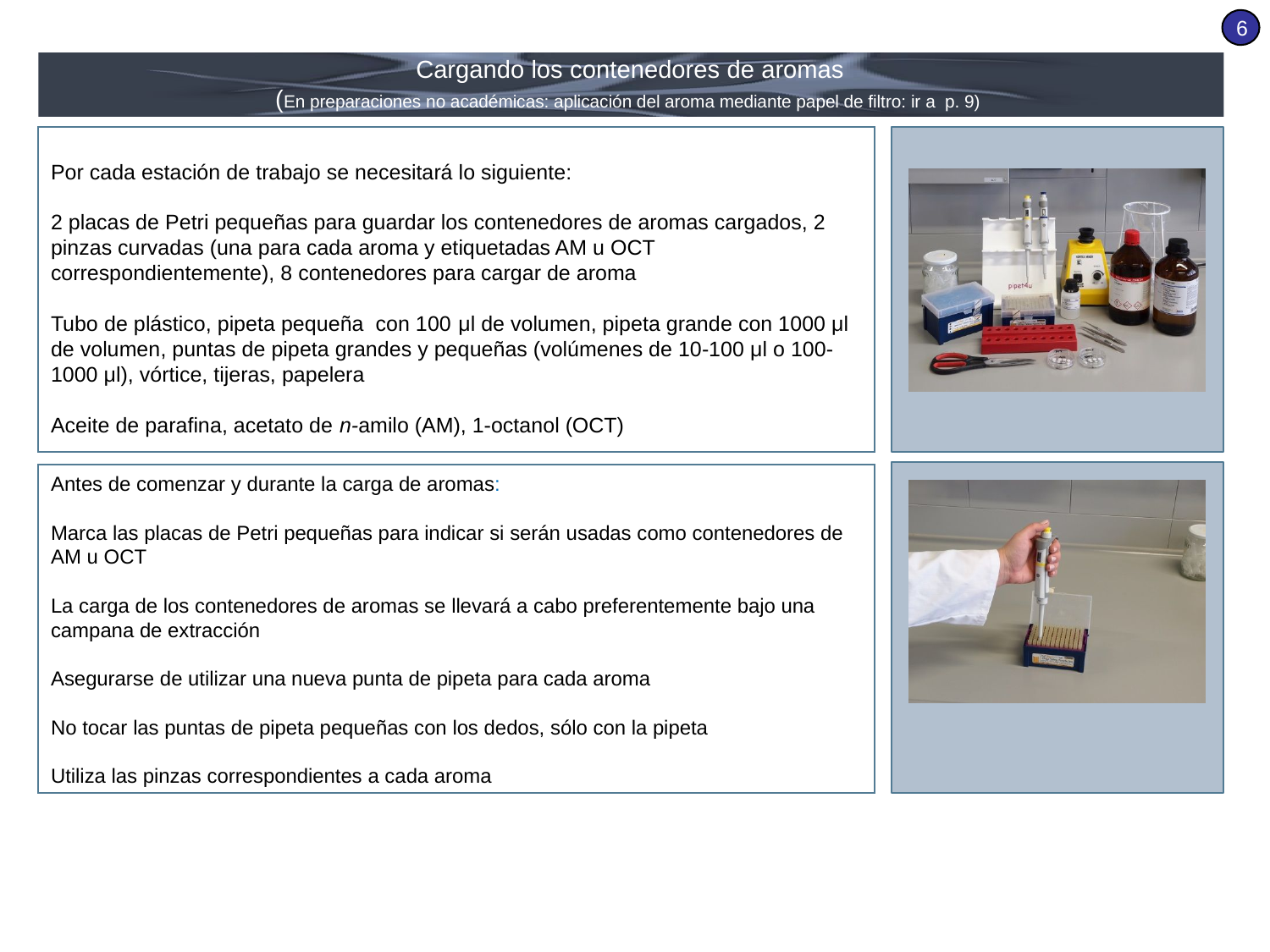

6
# Cargando los contenedores de aromas (En preparaciones no académicas: aplicación del aroma mediante papel de filtro: ir a p. 9)
Por cada estación de trabajo se necesitará lo siguiente:
2 placas de Petri pequeñas para guardar los contenedores de aromas cargados, 2 pinzas curvadas (una para cada aroma y etiquetadas AM u OCT correspondientemente), 8 contenedores para cargar de aroma
Tubo de plástico, pipeta pequeña con 100 μl de volumen, pipeta grande con 1000 μl de volumen, puntas de pipeta grandes y pequeñas (volúmenes de 10-100 μl o 100-1000 μl), vórtice, tijeras, papelera
 
Aceite de parafina, acetato de n-amilo (AM), 1-octanol (OCT)
Antes de comenzar y durante la carga de aromas:
Marca las placas de Petri pequeñas para indicar si serán usadas como contenedores de AM u OCT
La carga de los contenedores de aromas se llevará a cabo preferentemente bajo una campana de extracción
Asegurarse de utilizar una nueva punta de pipeta para cada aroma
No tocar las puntas de pipeta pequeñas con los dedos, sólo con la pipeta
Utiliza las pinzas correspondientes a cada aroma

## Slide 7
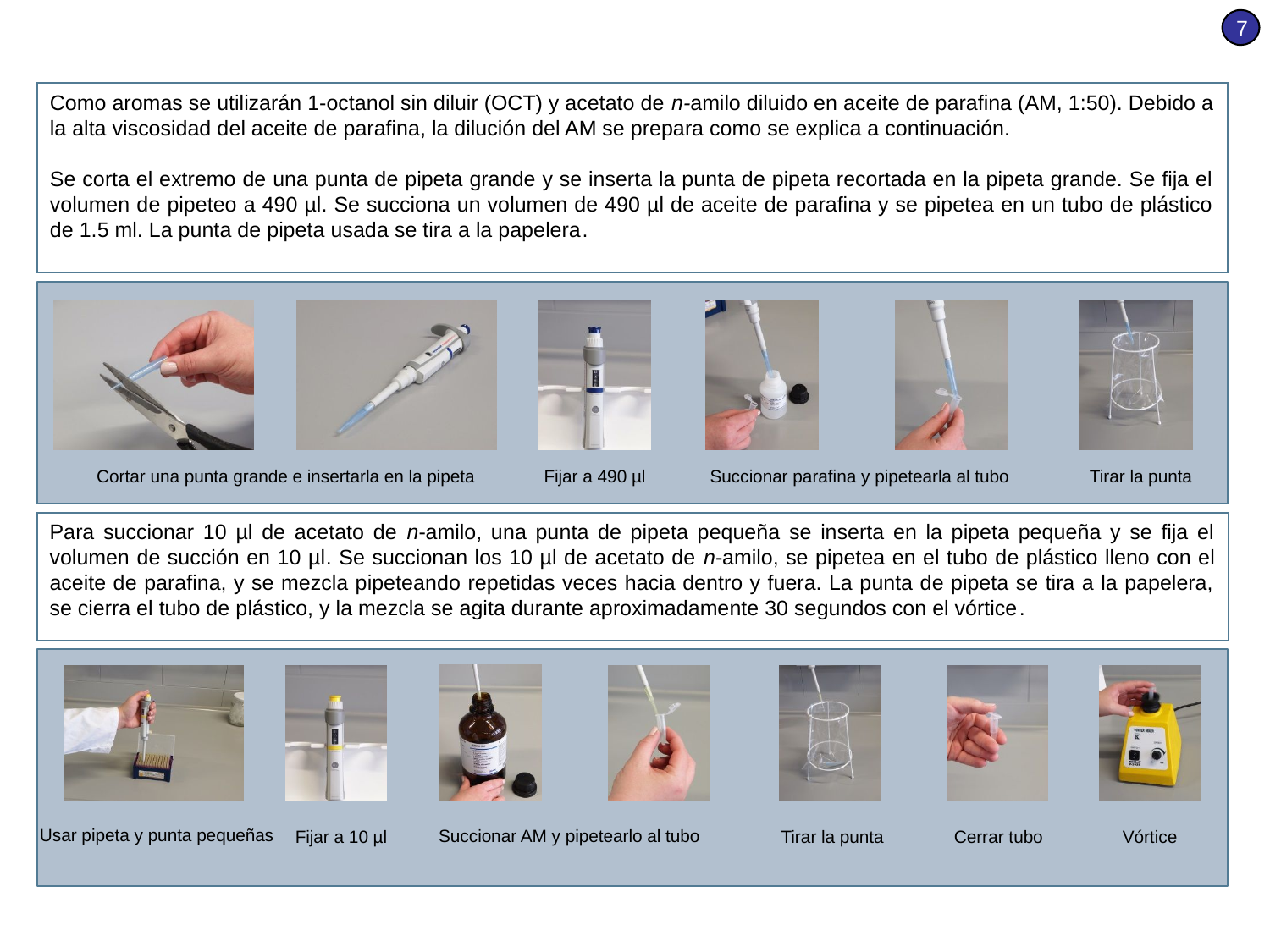

7
Como aromas se utilizarán 1-octanol sin diluir (OCT) y acetato de n-amilo diluido en aceite de parafina (AM, 1:50). Debido a la alta viscosidad del aceite de parafina, la dilución del AM se prepara como se explica a continuación.
Se corta el extremo de una punta de pipeta grande y se inserta la punta de pipeta recortada en la pipeta grande. Se fija el volumen de pipeteo a 490 µl. Se succiona un volumen de 490 µl de aceite de parafina y se pipetea en un tubo de plástico de 1.5 ml. La punta de pipeta usada se tira a la papelera.
Cortar una punta grande e insertarla en la pipeta
Fijar a 490 µl
Succionar parafina y pipetearla al tubo
Tirar la punta
Para succionar 10 µl de acetato de n-amilo, una punta de pipeta pequeña se inserta en la pipeta pequeña y se fija el volumen de succión en 10 µl. Se succionan los 10 µl de acetato de n-amilo, se pipetea en el tubo de plástico lleno con el aceite de parafina, y se mezcla pipeteando repetidas veces hacia dentro y fuera. La punta de pipeta se tira a la papelera, se cierra el tubo de plástico, y la mezcla se agita durante aproximadamente 30 segundos con el vórtice.
 Usar pipeta y punta pequeñas
Fijar a 10 µl
Succionar AM y pipetearlo al tubo
Tirar la punta
Cerrar tubo
Vórtice

## Slide 8
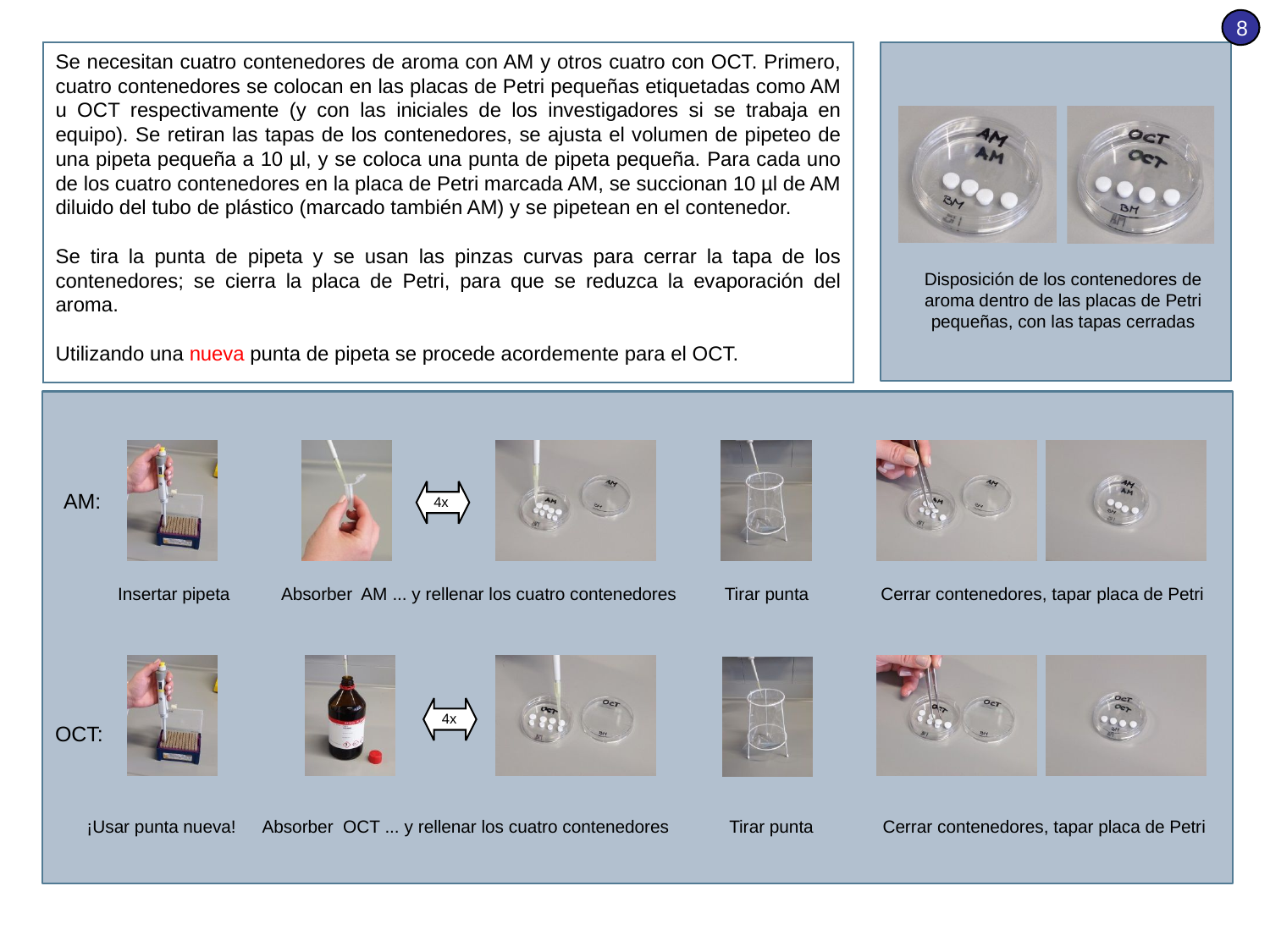

8
Se necesitan cuatro contenedores de aroma con AM y otros cuatro con OCT. Primero, cuatro contenedores se colocan en las placas de Petri pequeñas etiquetadas como AM u OCT respectivamente (y con las iniciales de los investigadores si se trabaja en equipo). Se retiran las tapas de los contenedores, se ajusta el volumen de pipeteo de una pipeta pequeña a 10 µl, y se coloca una punta de pipeta pequeña. Para cada uno de los cuatro contenedores en la placa de Petri marcada AM, se succionan 10 µl de AM diluido del tubo de plástico (marcado también AM) y se pipetean en el contenedor.
Se tira la punta de pipeta y se usan las pinzas curvas para cerrar la tapa de los contenedores; se cierra la placa de Petri, para que se reduzca la evaporación del aroma.
Utilizando una nueva punta de pipeta se procede acordemente para el OCT.
Disposición de los contenedores de aroma dentro de las placas de Petri pequeñas, con las tapas cerradas
4x
AM:
Insertar pipeta
Absorber AM ... y rellenar los cuatro contenedores
Tirar punta
Cerrar contenedores, tapar placa de Petri
4x
OCT:
¡Usar punta nueva!
Absorber OCT ... y rellenar los cuatro contenedores
Tirar punta
Cerrar contenedores, tapar placa de Petri

## Slide 9
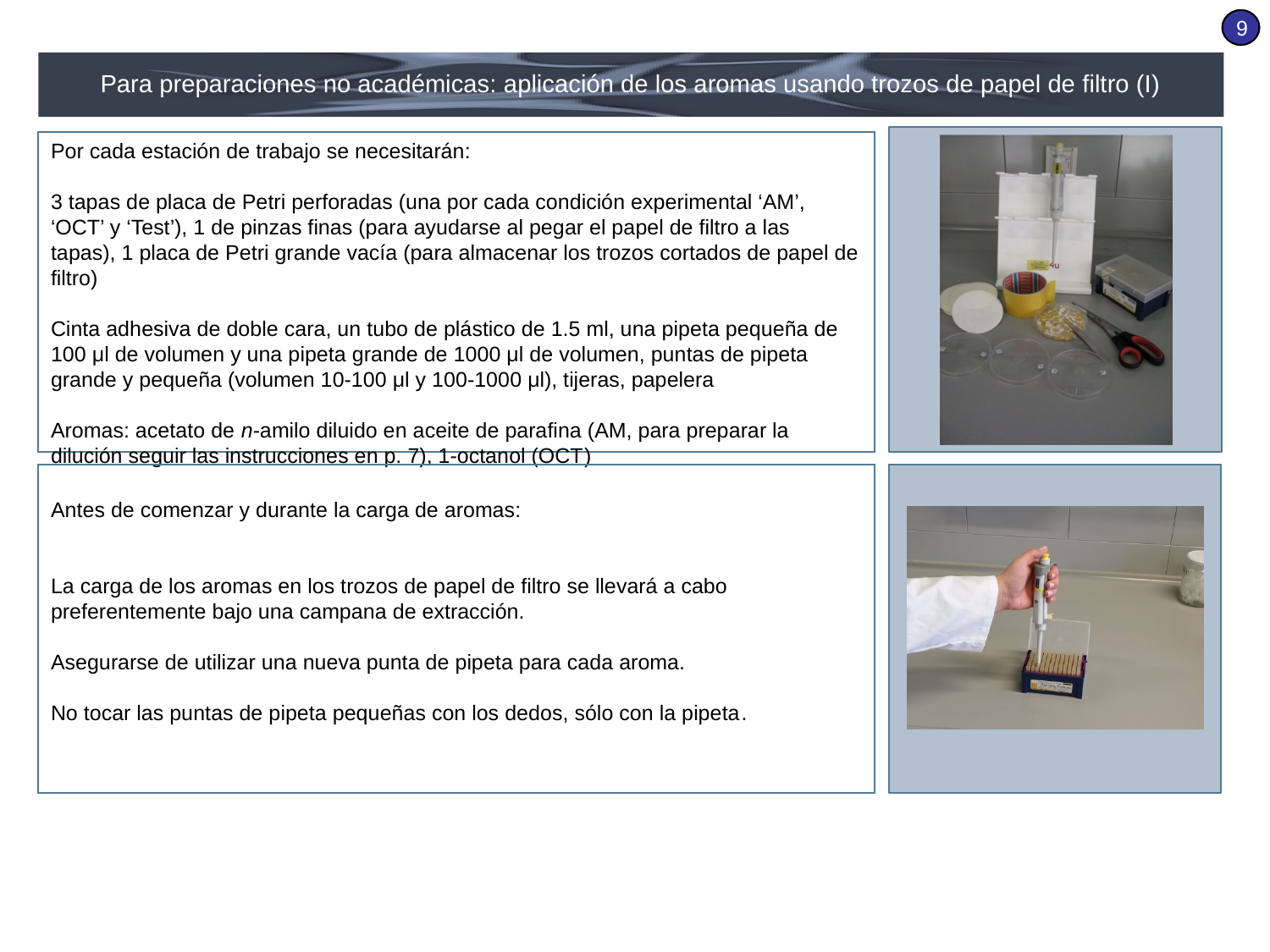

9
# Para preparaciones no académicas: aplicación de los aromas usando trozos de papel de filtro (I)
Por cada estación de trabajo se necesitarán:
3 tapas de placa de Petri perforadas (una por cada condición experimental ‘AM’, ‘OCT’ y ‘Test’), 1 de pinzas finas (para ayudarse al pegar el papel de filtro a las tapas), 1 placa de Petri grande vacía (para almacenar los trozos cortados de papel de filtro)
Cinta adhesiva de doble cara, un tubo de plástico de 1.5 ml, una pipeta pequeña de 100 μl de volumen y una pipeta grande de 1000 μl de volumen, puntas de pipeta grande y pequeña (volumen 10-100 μl y 100-1000 μl), tijeras, papelera
 
Aromas: acetato de n-amilo diluido en aceite de parafina (AM, para preparar la dilución seguir las instrucciones en p. 7), 1-octanol (OCT)
Antes de comenzar y durante la carga de aromas:
La carga de los aromas en los trozos de papel de filtro se llevará a cabo preferentemente bajo una campana de extracción.
Asegurarse de utilizar una nueva punta de pipeta para cada aroma.
No tocar las puntas de pipeta pequeñas con los dedos, sólo con la pipeta.

## Slide 10
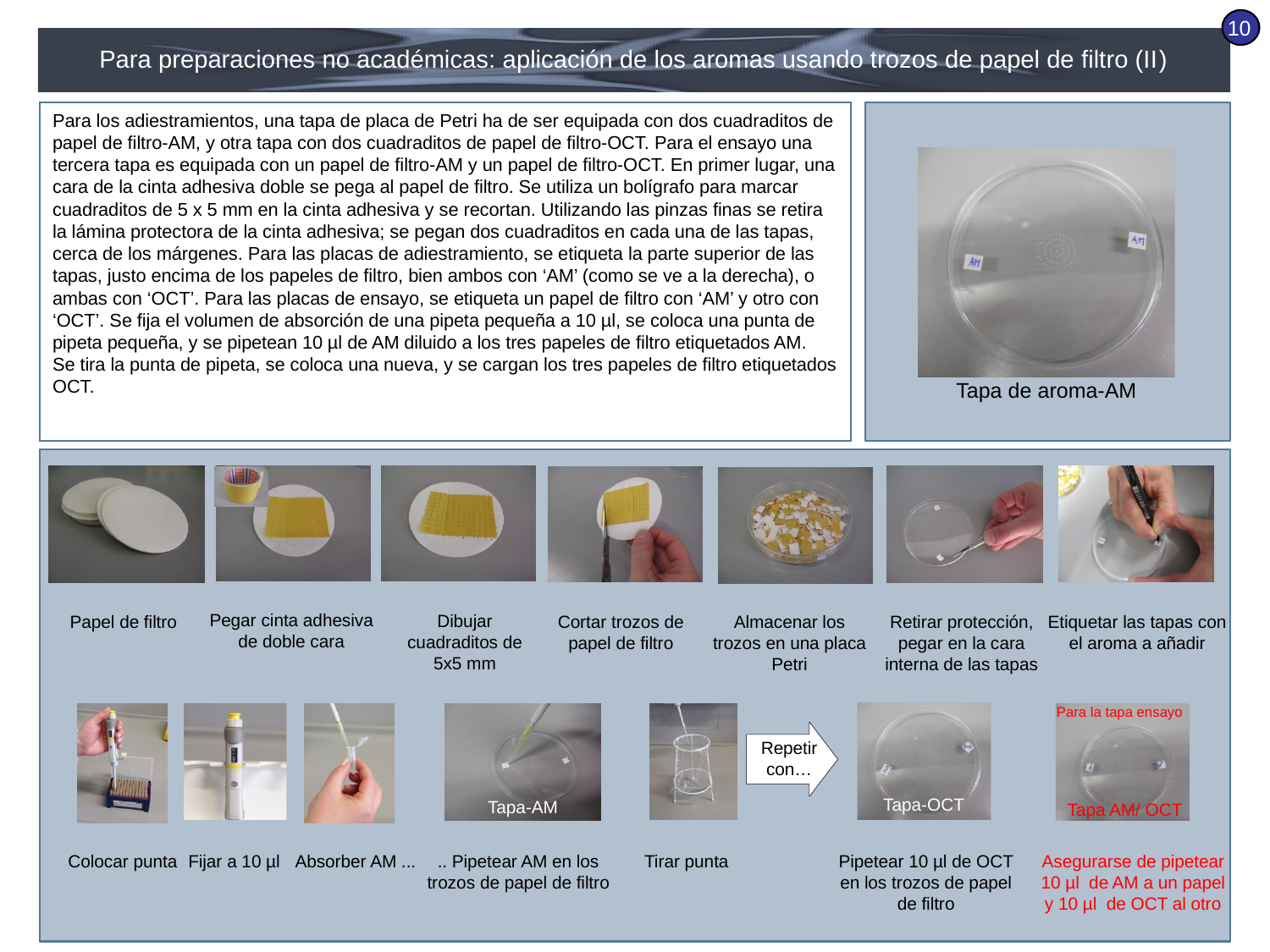

10
# Para preparaciones no académicas: aplicación de los aromas usando trozos de papel de filtro (II)
Para los adiestramientos, una tapa de placa de Petri ha de ser equipada con dos cuadraditos de papel de filtro-AM, y otra tapa con dos cuadraditos de papel de filtro-OCT. Para el ensayo una tercera tapa es equipada con un papel de filtro-AM y un papel de filtro-OCT. En primer lugar, una cara de la cinta adhesiva doble se pega al papel de filtro. Se utiliza un bolígrafo para marcar cuadraditos de 5 x 5 mm en la cinta adhesiva y se recortan. Utilizando las pinzas finas se retira la lámina protectora de la cinta adhesiva; se pegan dos cuadraditos en cada una de las tapas, cerca de los márgenes. Para las placas de adiestramiento, se etiqueta la parte superior de las tapas, justo encima de los papeles de filtro, bien ambos con ‘AM’ (como se ve a la derecha), o ambas con ‘OCT’. Para las placas de ensayo, se etiqueta un papel de filtro con ‘AM’ y otro con ‘OCT’. Se fija el volumen de absorción de una pipeta pequeña a 10 µl, se coloca una punta de pipeta pequeña, y se pipetean 10 µl de AM diluido a los tres papeles de filtro etiquetados AM.
Se tira la punta de pipeta, se coloca una nueva, y se cargan los tres papeles de filtro etiquetados OCT.
Tapa de aroma-AM
Pegar cinta adhesiva de doble cara
Dibujar cuadraditos de 5x5 mm
Papel de filtro
Cortar trozos de papel de filtro
Almacenar los trozos en una placa Petri
Retirar protección, pegar en la cara interna de las tapas
Etiquetar las tapas con el aroma a añadir
Para la tapa ensayo
Repetir con…
Tapa-OCT
Tapa-AM
Tapa AM/ OCT
Colocar punta
Fijar a 10 µl
Absorber AM ...
.. Pipetear AM en los trozos de papel de filtro
Tirar punta
Pipetear 10 µl de OCT en los trozos de papel de filtro
Asegurarse de pipetear 10 µl de AM a un papel y 10 µl de OCT al otro

## Slide 11
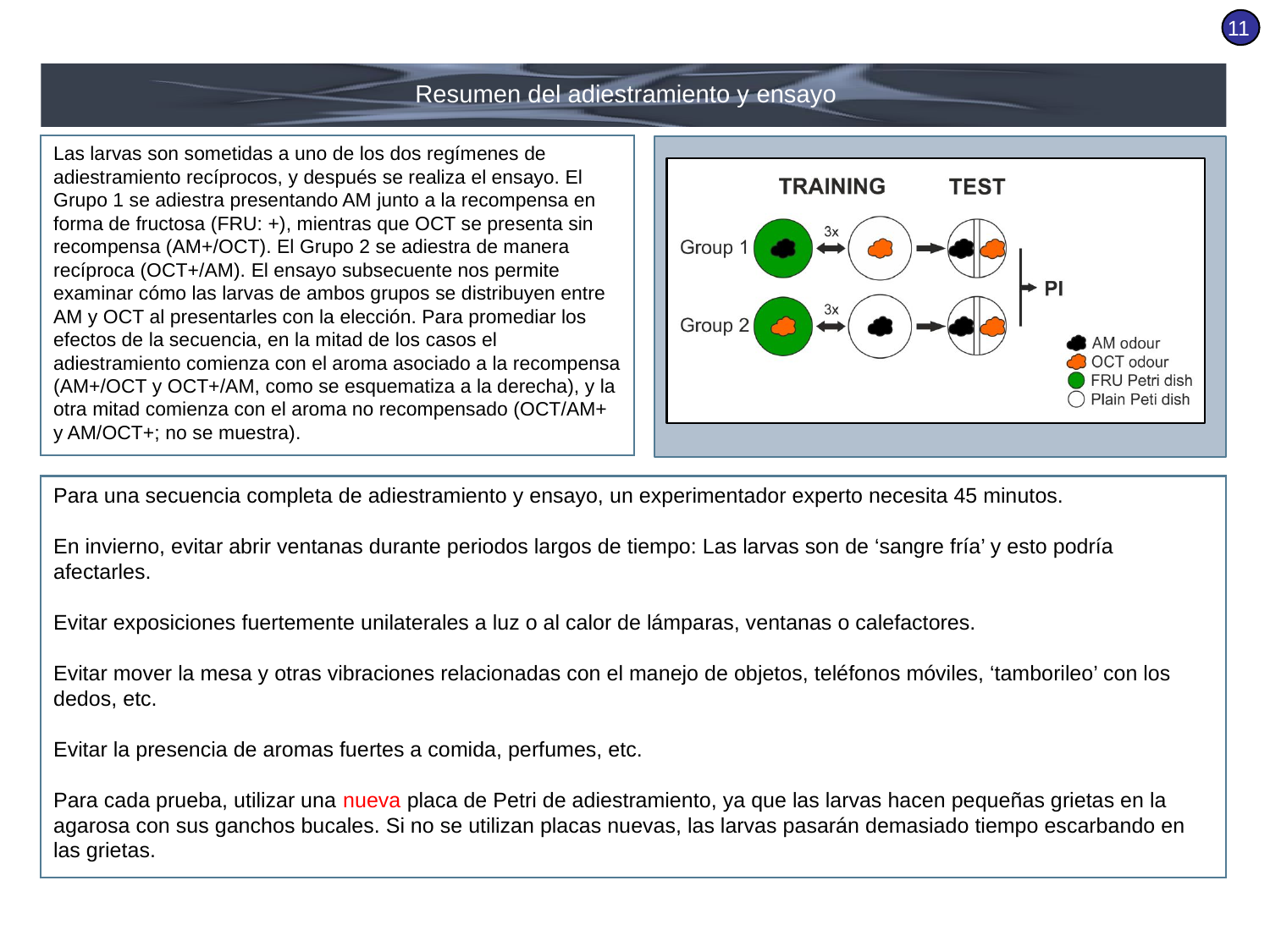

11
Resumen del adiestramiento y ensayo
Las larvas son sometidas a uno de los dos regímenes de adiestramiento recíprocos, y después se realiza el ensayo. El Grupo 1 se adiestra presentando AM junto a la recompensa en forma de fructosa (FRU: +), mientras que OCT se presenta sin recompensa (AM+/OCT). El Grupo 2 se adiestra de manera recíproca (OCT+/AM). El ensayo subsecuente nos permite examinar cómo las larvas de ambos grupos se distribuyen entre AM y OCT al presentarles con la elección. Para promediar los efectos de la secuencia, en la mitad de los casos el adiestramiento comienza con el aroma asociado a la recompensa (AM+/OCT y OCT+/AM, como se esquematiza a la derecha), y la otra mitad comienza con el aroma no recompensado (OCT/AM+ y AM/OCT+; no se muestra).
Para una secuencia completa de adiestramiento y ensayo, un experimentador experto necesita 45 minutos.
En invierno, evitar abrir ventanas durante periodos largos de tiempo: Las larvas son de ‘sangre fría’ y esto podría afectarles.
Evitar exposiciones fuertemente unilaterales a luz o al calor de lámparas, ventanas o calefactores.
Evitar mover la mesa y otras vibraciones relacionadas con el manejo de objetos, teléfonos móviles, ‘tamborileo’ con los dedos, etc.
Evitar la presencia de aromas fuertes a comida, perfumes, etc.
Para cada prueba, utilizar una nueva placa de Petri de adiestramiento, ya que las larvas hacen pequeñas grietas en la agarosa con sus ganchos bucales. Si no se utilizan placas nuevas, las larvas pasarán demasiado tiempo escarbando en las grietas.

## Slide 12
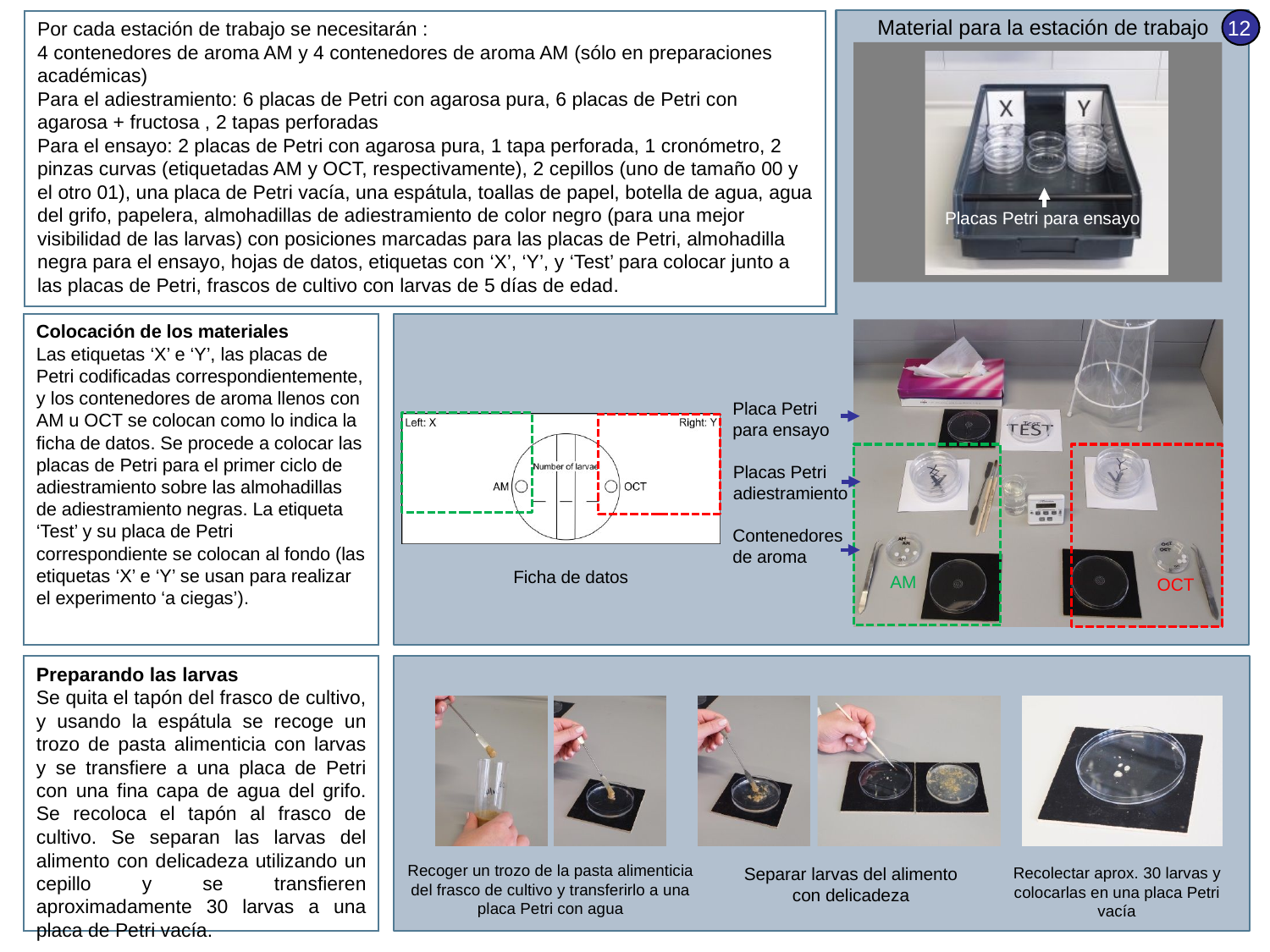

Material para la estación de trabajo
12
Por cada estación de trabajo se necesitarán :
4 contenedores de aroma AM y 4 contenedores de aroma AM (sólo en preparaciones académicas)
Para el adiestramiento: 6 placas de Petri con agarosa pura, 6 placas de Petri con agarosa + fructosa , 2 tapas perforadas
Para el ensayo: 2 placas de Petri con agarosa pura, 1 tapa perforada, 1 cronómetro, 2 pinzas curvas (etiquetadas AM y OCT, respectivamente), 2 cepillos (uno de tamaño 00 y el otro 01), una placa de Petri vacía, una espátula, toallas de papel, botella de agua, agua del grifo, papelera, almohadillas de adiestramiento de color negro (para una mejor visibilidad de las larvas) con posiciones marcadas para las placas de Petri, almohadilla negra para el ensayo, hojas de datos, etiquetas con ‘X’, ‘Y’, y ‘Test’ para colocar junto a las placas de Petri, frascos de cultivo con larvas de 5 días de edad.
Placas Petri para ensayo
Colocación de los materiales
Las etiquetas ‘X’ e ‘Y’, las placas de Petri codificadas correspondientemente, y los contenedores de aroma llenos con AM u OCT se colocan como lo indica la ficha de datos. Se procede a colocar las placas de Petri para el primer ciclo de adiestramiento sobre las almohadillas de adiestramiento negras. La etiqueta ‘Test’ y su placa de Petri correspondiente se colocan al fondo (las etiquetas ‘X’ e ‘Y’ se usan para realizar el experimento ‘a ciegas’).
Placa Petri
para ensayo
OCT
Y
Placas Petri
adiestramiento
Contenedores
de aroma
Ficha de datos
AM
OCT
Preparando las larvas
Se quita el tapón del frasco de cultivo, y usando la espátula se recoge un trozo de pasta alimenticia con larvas y se transfiere a una placa de Petri con una fina capa de agua del grifo. Se recoloca el tapón al frasco de cultivo. Se separan las larvas del alimento con delicadeza utilizando un cepillo y se transfieren aproximadamente 30 larvas a una placa de Petri vacía.
Recoger un trozo de la pasta alimenticia del frasco de cultivo y transferirlo a una placa Petri con agua
Separar larvas del alimento con delicadeza
Recolectar aprox. 30 larvas y colocarlas en una placa Petri vacía

## Slide 13
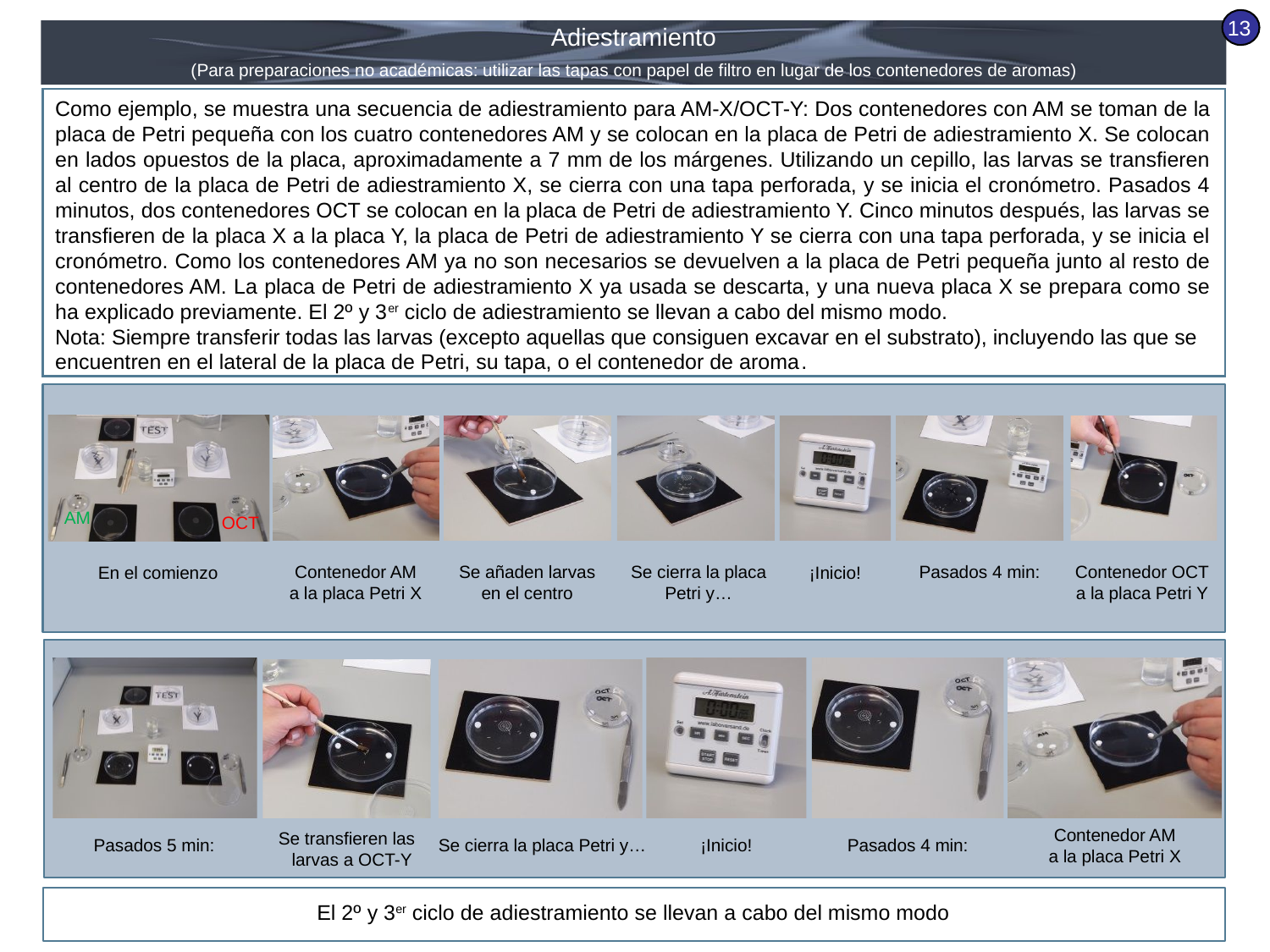

13
# Adiestramiento
(Para preparaciones no académicas: utilizar las tapas con papel de filtro en lugar de los contenedores de aromas)
Como ejemplo, se muestra una secuencia de adiestramiento para AM-X/OCT-Y: Dos contenedores con AM se toman de la placa de Petri pequeña con los cuatro contenedores AM y se colocan en la placa de Petri de adiestramiento X. Se colocan en lados opuestos de la placa, aproximadamente a 7 mm de los márgenes. Utilizando un cepillo, las larvas se transfieren al centro de la placa de Petri de adiestramiento X, se cierra con una tapa perforada, y se inicia el cronómetro. Pasados 4 minutos, dos contenedores OCT se colocan en la placa de Petri de adiestramiento Y. Cinco minutos después, las larvas se transfieren de la placa X a la placa Y, la placa de Petri de adiestramiento Y se cierra con una tapa perforada, y se inicia el cronómetro. Como los contenedores AM ya no son necesarios se devuelven a la placa de Petri pequeña junto al resto de contenedores AM. La placa de Petri de adiestramiento X ya usada se descarta, y una nueva placa X se prepara como se ha explicado previamente. El 2º y 3er ciclo de adiestramiento se llevan a cabo del mismo modo.
Nota: Siempre transferir todas las larvas (excepto aquellas que consiguen excavar en el substrato), incluyendo las que se encuentren en el lateral de la placa de Petri, su tapa, o el contenedor de aroma.
AM
OCT
Contenedor AM
a la placa Petri X
Pasados 4 min:
Se añaden larvas en el centro
Se cierra la placa Petri y…
Contenedor OCT
a la placa Petri Y
En el comienzo
¡Inicio!
Contenedor AM
a la placa Petri X
Se transfieren las larvas a OCT-Y
Se cierra la placa Petri y…
Pasados 5 min:
¡Inicio!
Pasados 4 min:
El 2º y 3er ciclo de adiestramiento se llevan a cabo del mismo modo

## Slide 14
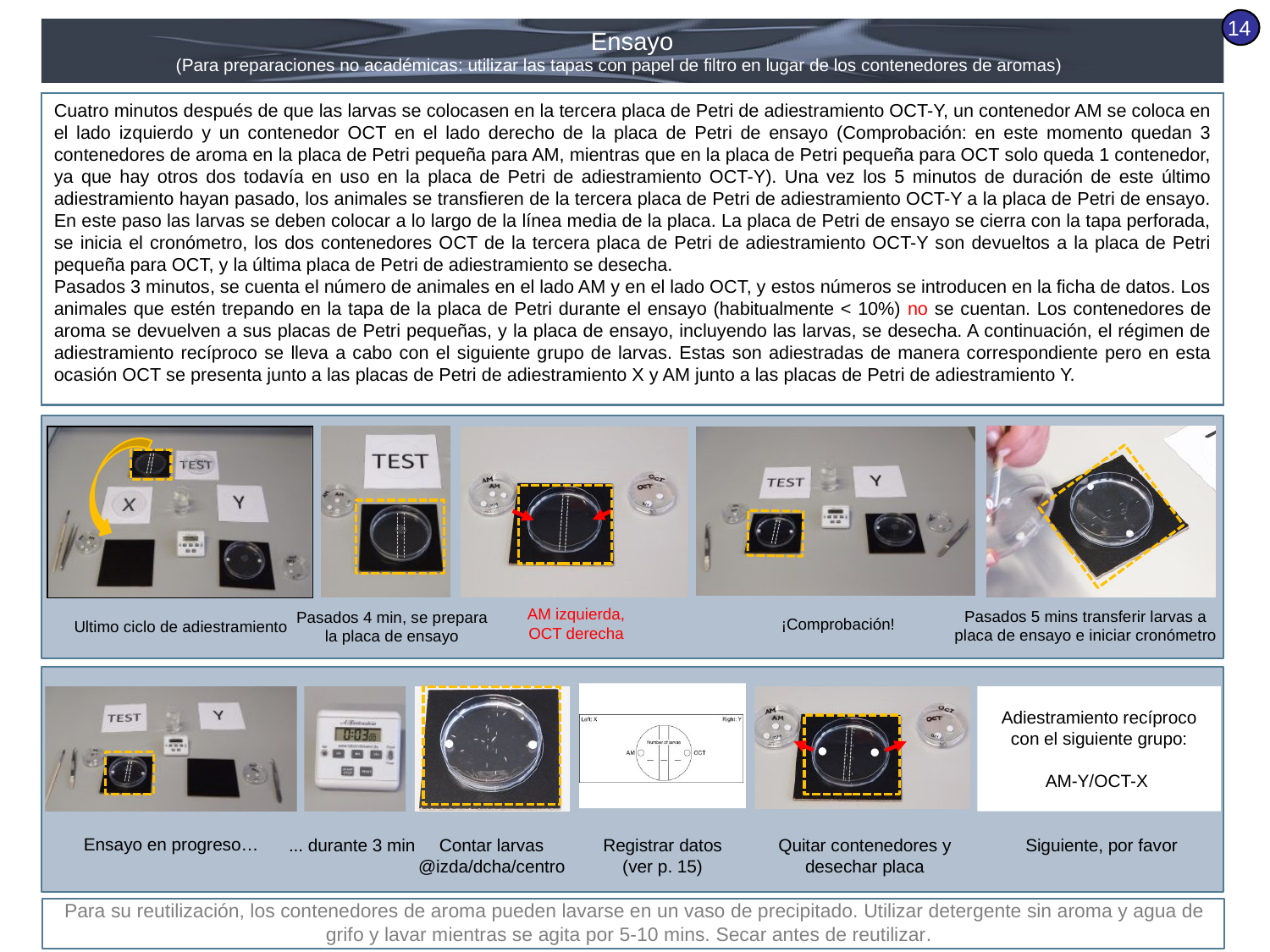

# Ensayo
14
(Para preparaciones no académicas: utilizar las tapas con papel de filtro en lugar de los contenedores de aromas)
Cuatro minutos después de que las larvas se colocasen en la tercera placa de Petri de adiestramiento OCT-Y, un contenedor AM se coloca en el lado izquierdo y un contenedor OCT en el lado derecho de la placa de Petri de ensayo (Comprobación: en este momento quedan 3 contenedores de aroma en la placa de Petri pequeña para AM, mientras que en la placa de Petri pequeña para OCT solo queda 1 contenedor, ya que hay otros dos todavía en uso en la placa de Petri de adiestramiento OCT-Y). Una vez los 5 minutos de duración de este último adiestramiento hayan pasado, los animales se transfieren de la tercera placa de Petri de adiestramiento OCT-Y a la placa de Petri de ensayo. En este paso las larvas se deben colocar a lo largo de la línea media de la placa. La placa de Petri de ensayo se cierra con la tapa perforada, se inicia el cronómetro, los dos contenedores OCT de la tercera placa de Petri de adiestramiento OCT-Y son devueltos a la placa de Petri pequeña para OCT, y la última placa de Petri de adiestramiento se desecha.
Pasados 3 minutos, se cuenta el número de animales en el lado AM y en el lado OCT, y estos números se introducen en la ficha de datos. Los animales que estén trepando en la tapa de la placa de Petri durante el ensayo (habitualmente < 10%) no se cuentan. Los contenedores de aroma se devuelven a sus placas de Petri pequeñas, y la placa de ensayo, incluyendo las larvas, se desecha. A continuación, el régimen de adiestramiento recíproco se lleva a cabo con el siguiente grupo de larvas. Estas son adiestradas de manera correspondiente pero en esta ocasión OCT se presenta junto a las placas de Petri de adiestramiento X y AM junto a las placas de Petri de adiestramiento Y.
AM izquierda,
OCT derecha
Pasados 5 mins transferir larvas a
placa de ensayo e iniciar cronómetro
Pasados 4 min, se prepara
la placa de ensayo
¡Comprobación!
Ultimo ciclo de adiestramiento
Adiestramiento recíproco con el siguiente grupo:
AM-Y/OCT-X
Ensayo en progreso…
Contar larvas
@izda/dcha/centro
Siguiente, por favor
... durante 3 min
Registrar datos
(ver p. 15)
Quitar contenedores y desechar placa
Para su reutilización, los contenedores de aroma pueden lavarse en un vaso de precipitado. Utilizar detergente sin aroma y agua de grifo y lavar mientras se agita por 5-10 mins. Secar antes de reutilizar.

## Slide 15
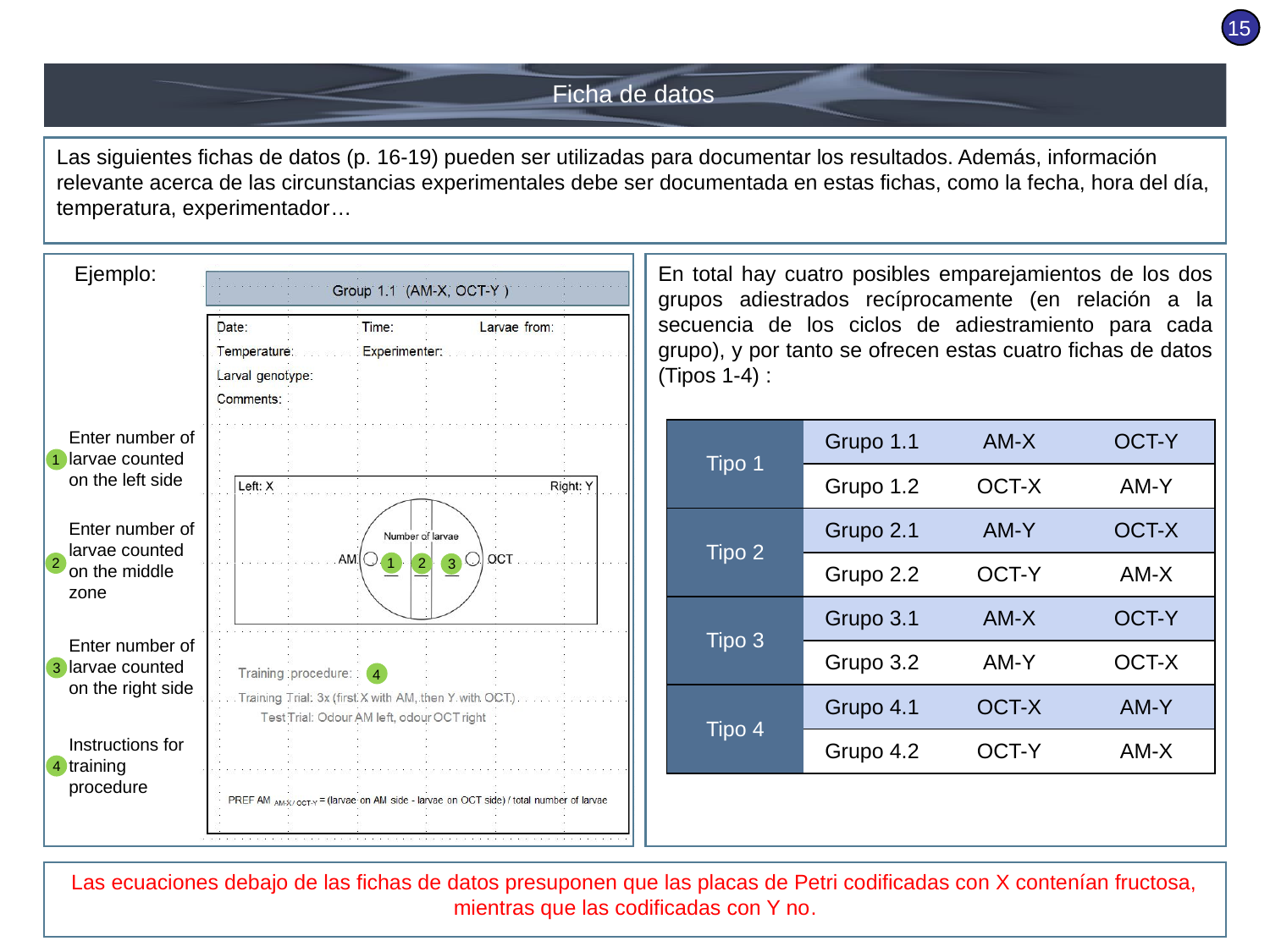

15
Ficha de datos
Las siguientes fichas de datos (p. 16-19) pueden ser utilizadas para documentar los resultados. Además, información relevante acerca de las circunstancias experimentales debe ser documentada en estas fichas, como la fecha, hora del día, temperatura, experimentador…
 Ejemplo:
En total hay cuatro posibles emparejamientos de los dos grupos adiestrados recíprocamente (en relación a la secuencia de los ciclos de adiestramiento para cada grupo), y por tanto se ofrecen estas cuatro fichas de datos (Tipos 1-4) :
| Tipo 1 | Grupo 1.1 | AM-X | OCT-Y |
| --- | --- | --- | --- |
| | Grupo 1.2 | OCT-X | AM-Y |
| Tipo 2 | Grupo 2.1 | AM-Y | OCT-X |
| | Grupo 2.2 | OCT-Y | AM-X |
| Tipo 3 | Grupo 3.1 | AM-X | OCT-Y |
| | Grupo 3.2 | AM-Y | OCT-X |
| Tipo 4 | Grupo 4.1 | OCT-X | AM-Y |
| | Grupo 4.2 | OCT-Y | AM-X |
Enter number of larvae counted on the left side
1
Enter number of larvae counted on the middle zone
1
2
2
3
Enter number of larvae counted on the right side
3
4
Instructions for training procedure
4
Las ecuaciones debajo de las fichas de datos presuponen que las placas de Petri codificadas con X contenían fructosa, mientras que las codificadas con Y no.

## Slide 16
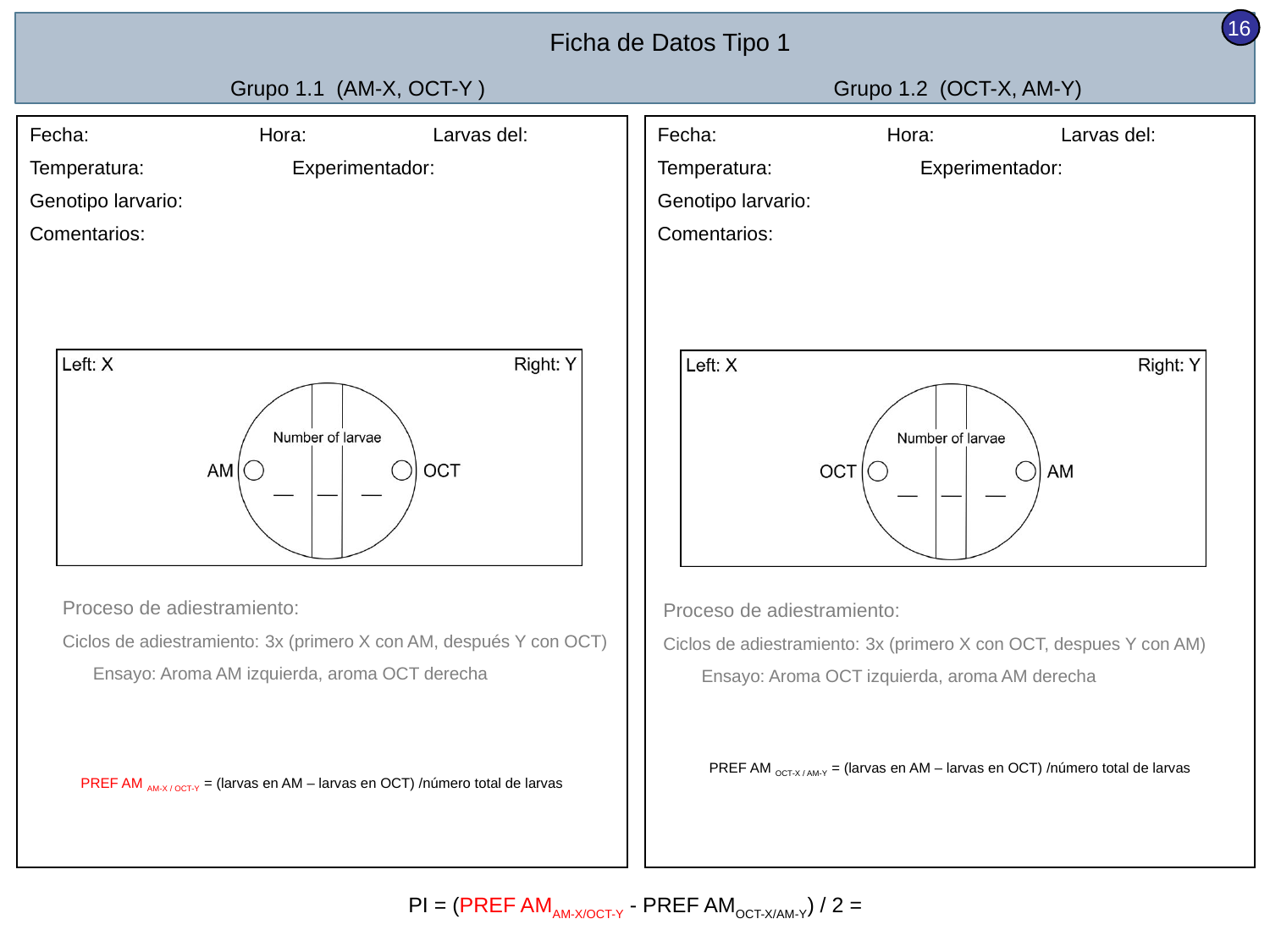

16
# Ficha de Datos Tipo 1 Grupo 1.1 (AM-X, OCT-Y ) Grupo 1.2 (OCT-X, AM-Y)
Fecha: Hora: Larvas del:
Temperatura: 	 Experimentador:
Genotipo larvario:
Comentarios:
 Proceso de adiestramiento:
 Ciclos de adiestramiento: 3x (primero X con AM, después Y con OCT)
 Ensayo: Aroma AM izquierda, aroma OCT derecha
PREF AM AM-X / OCT-Y = (larvas en AM – larvas en OCT) /número total de larvas
Fecha: Hora: Larvas del:
Temperatura: 	 Experimentador:
Genotipo larvario:
Comentarios:
 Proceso de adiestramiento:
 Ciclos de adiestramiento: 3x (primero X con OCT, despues Y con AM)
 Ensayo: Aroma OCT izquierda, aroma AM derecha
PREF AM OCT-X / AM-Y = (larvas en AM – larvas en OCT) /número total de larvas
PI = (PREF AMAM-X/OCT-Y - PREF AMOCT-X/AM-Y) / 2 =

## Slide 17
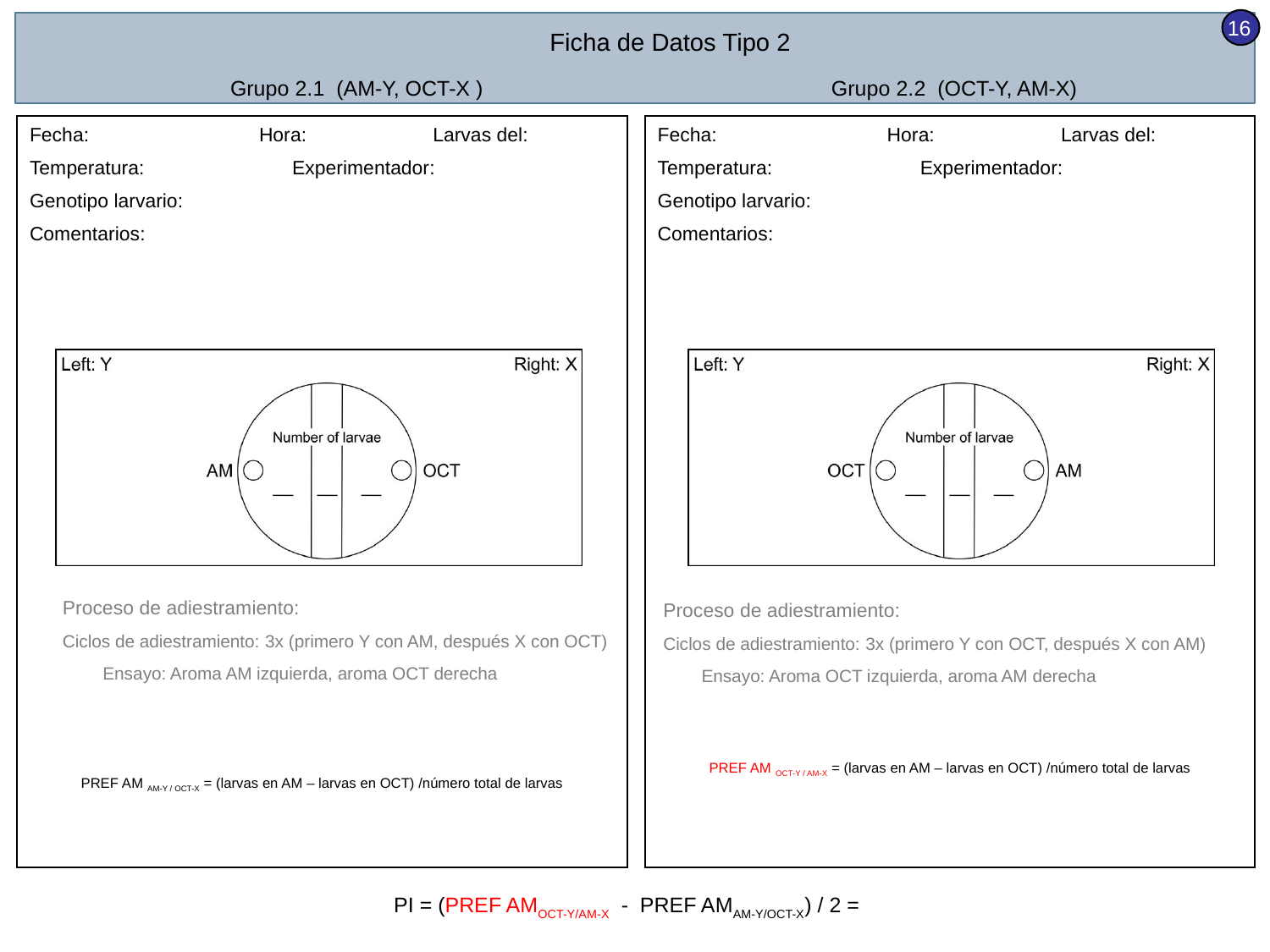

16
# Ficha de Datos Tipo 2 Grupo 2.1 (AM-Y, OCT-X ) Grupo 2.2 (OCT-Y, AM-X)
Fecha: Hora: Larvas del:
Temperatura: 	 Experimentador:
Genotipo larvario:
Comentarios:
 Proceso de adiestramiento:
 Ciclos de adiestramiento: 3x (primero Y con AM, después X con OCT)
 Ensayo: Aroma AM izquierda, aroma OCT derecha
PREF AM AM-Y / OCT-X = (larvas en AM – larvas en OCT) /número total de larvas
Fecha: Hora: Larvas del:
Temperatura: 	 Experimentador:
Genotipo larvario:
Comentarios:
 Proceso de adiestramiento:
 Ciclos de adiestramiento: 3x (primero Y con OCT, después X con AM)
 Ensayo: Aroma OCT izquierda, aroma AM derecha
PREF AM OCT-Y / AM-X = (larvas en AM – larvas en OCT) /número total de larvas
PI = (PREF AMOCT-Y/AM-X - PREF AMAM-Y/OCT-X) / 2 =

## Slide 18
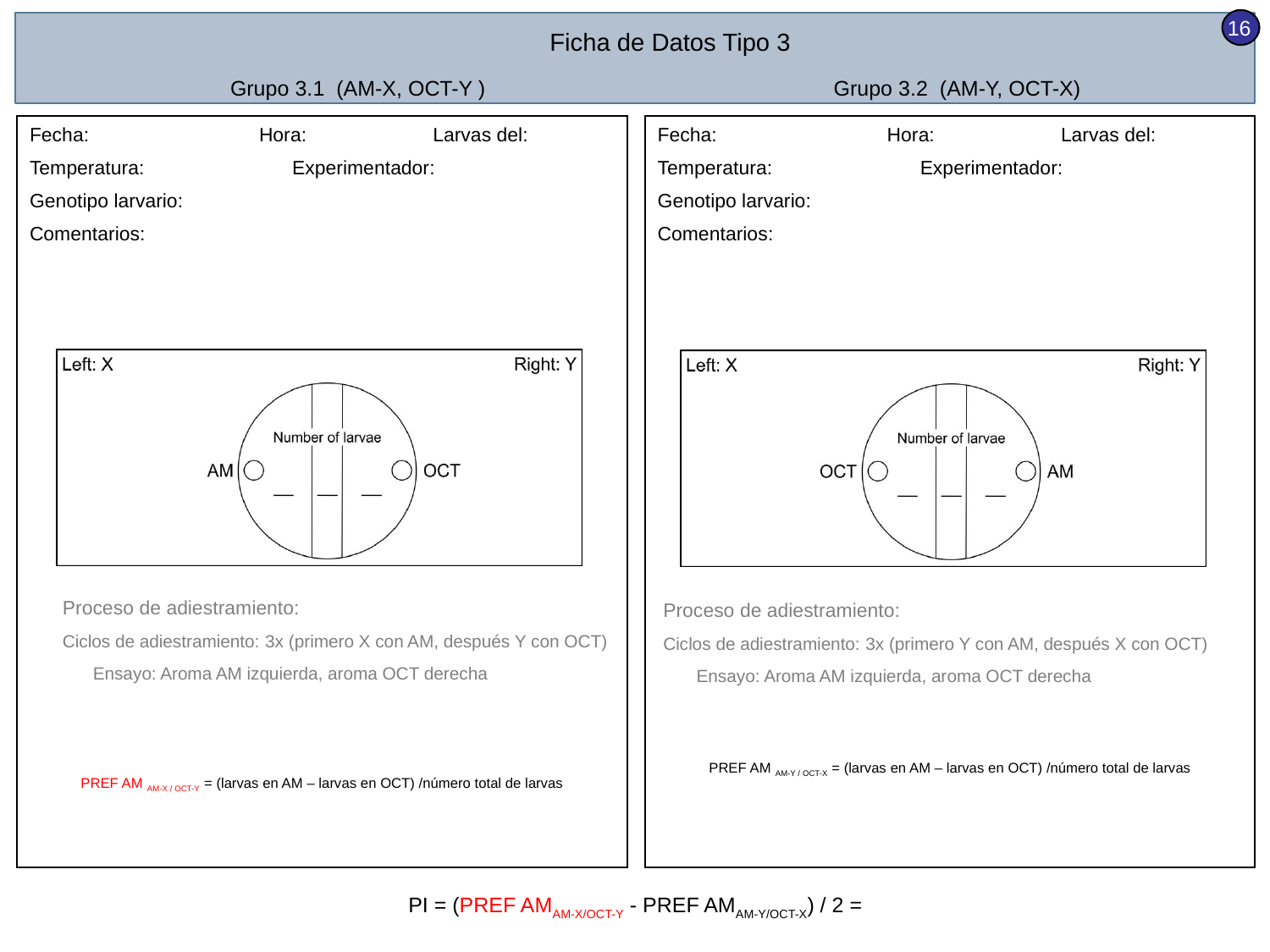

16
# Ficha de Datos Tipo 3 Grupo 3.1 (AM-X, OCT-Y ) Grupo 3.2 (AM-Y, OCT-X)
Fecha: Hora: Larvas del:
Temperatura: 	 Experimentador:
Genotipo larvario:
Comentarios:
 Proceso de adiestramiento:
 Ciclos de adiestramiento: 3x (primero X con AM, después Y con OCT)
 Ensayo: Aroma AM izquierda, aroma OCT derecha
PREF AM AM-X / OCT-Y = (larvas en AM – larvas en OCT) /número total de larvas
Fecha: Hora: Larvas del:
Temperatura: 	 Experimentador:
Genotipo larvario:
Comentarios:
 Proceso de adiestramiento:
 Ciclos de adiestramiento: 3x (primero Y con AM, después X con OCT)
 Ensayo: Aroma AM izquierda, aroma OCT derecha
PREF AM AM-Y / OCT-X = (larvas en AM – larvas en OCT) /número total de larvas
PI = (PREF AMAM-X/OCT-Y - PREF AMAM-Y/OCT-X) / 2 =

## Slide 19
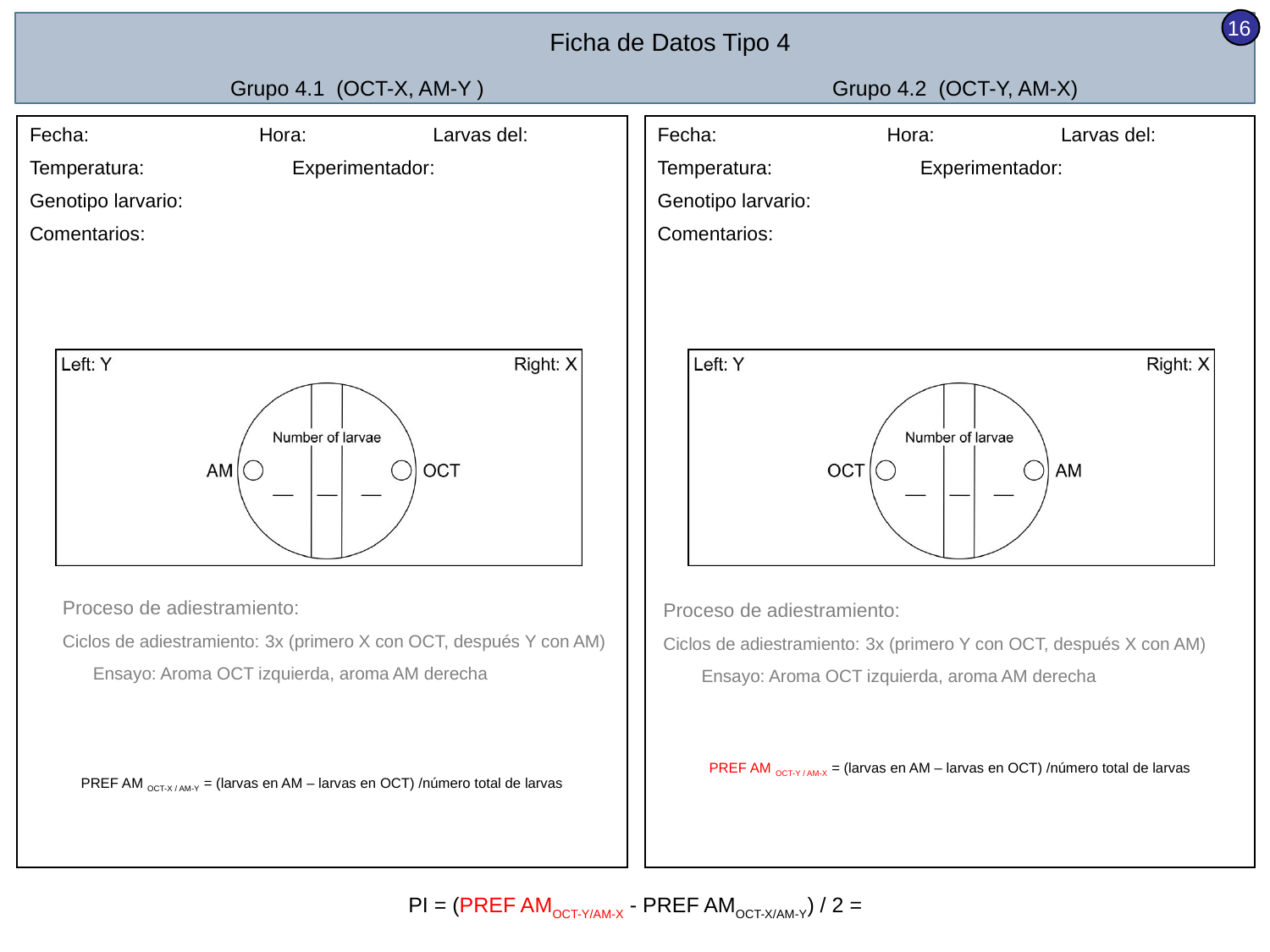

16
# Ficha de Datos Tipo 4 Grupo 4.1 (OCT-X, AM-Y ) Grupo 4.2 (OCT-Y, AM-X)
Fecha: Hora: Larvas del:
Temperatura: 	 Experimentador:
Genotipo larvario:
Comentarios:
 Proceso de adiestramiento:
 Ciclos de adiestramiento: 3x (primero X con OCT, después Y con AM)
 Ensayo: Aroma OCT izquierda, aroma AM derecha
PREF AM OCT-X / AM-Y = (larvas en AM – larvas en OCT) /número total de larvas
Fecha: Hora: Larvas del:
Temperatura: 	 Experimentador:
Genotipo larvario:
Comentarios:
 Proceso de adiestramiento:
 Ciclos de adiestramiento: 3x (primero Y con OCT, después X con AM)
 Ensayo: Aroma OCT izquierda, aroma AM derecha
PREF AM OCT-Y / AM-X = (larvas en AM – larvas en OCT) /número total de larvas
PI = (PREF AMOCT-Y/AM-X - PREF AMOCT-X/AM-Y) / 2 =

## Slide 20
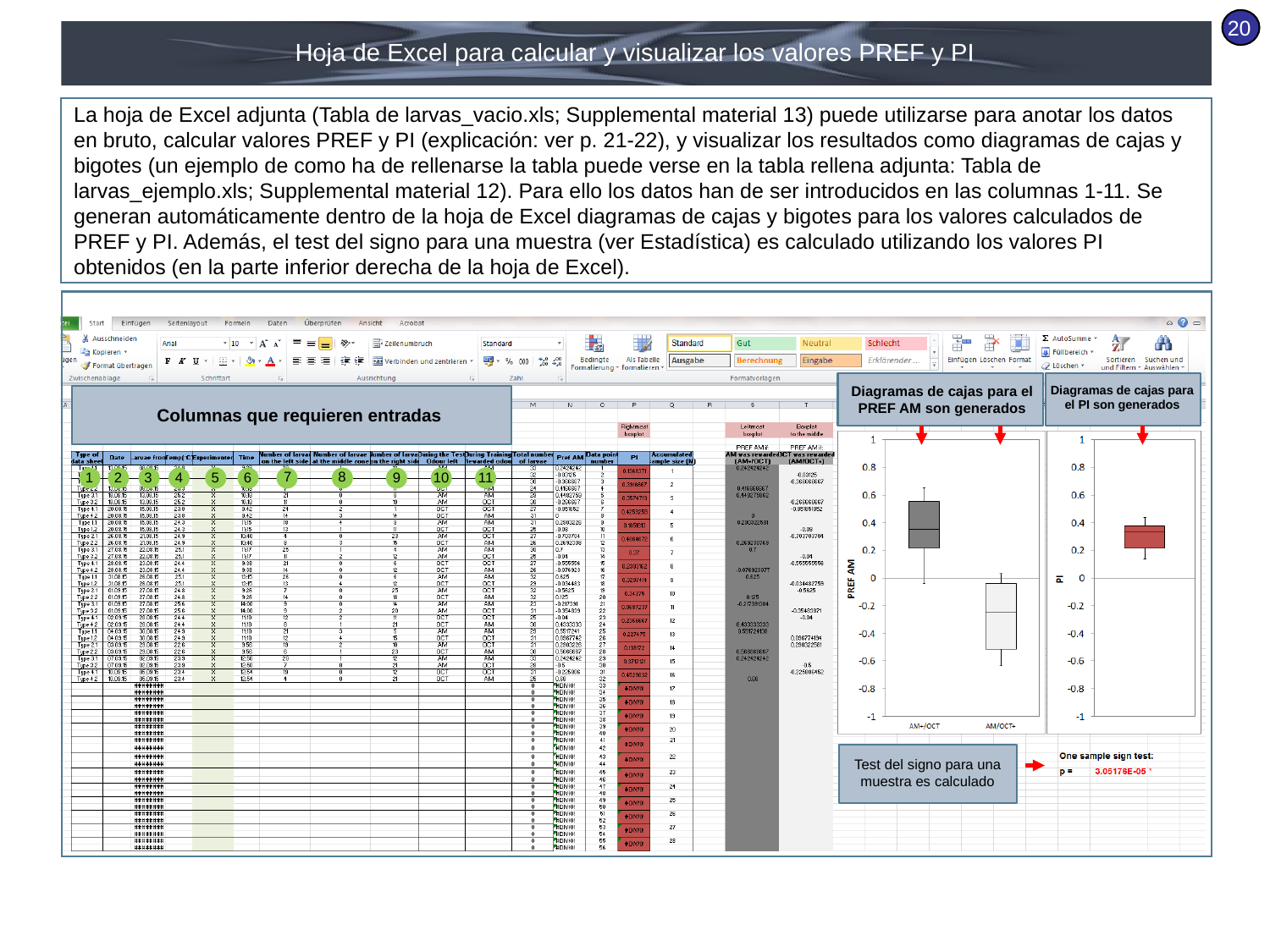

20
Hoja de Excel para calcular y visualizar los valores PREF y PI
La hoja de Excel adjunta (Tabla de larvas_vacio.xls; Supplemental material 13) puede utilizarse para anotar los datos en bruto, calcular valores PREF y PI (explicación: ver p. 21-22), y visualizar los resultados como diagramas de cajas y bigotes (un ejemplo de como ha de rellenarse la tabla puede verse en la tabla rellena adjunta: Tabla de larvas_ejemplo.xls; Supplemental material 12). Para ello los datos han de ser introducidos en las columnas 1-11. Se generan automáticamente dentro de la hoja de Excel diagramas de cajas y bigotes para los valores calculados de PREF y PI. Además, el test del signo para una muestra (ver Estadística) es calculado utilizando los valores PI obtenidos (en la parte inferior derecha de la hoja de Excel).
Diagramas de cajas para el PREF AM son generados
Diagramas de cajas para el PI son generados
Columnas que requieren entradas
7
8
3
4
2
5
6
9
10
1
11
Test del signo para una muestra es calculado

## Slide 21
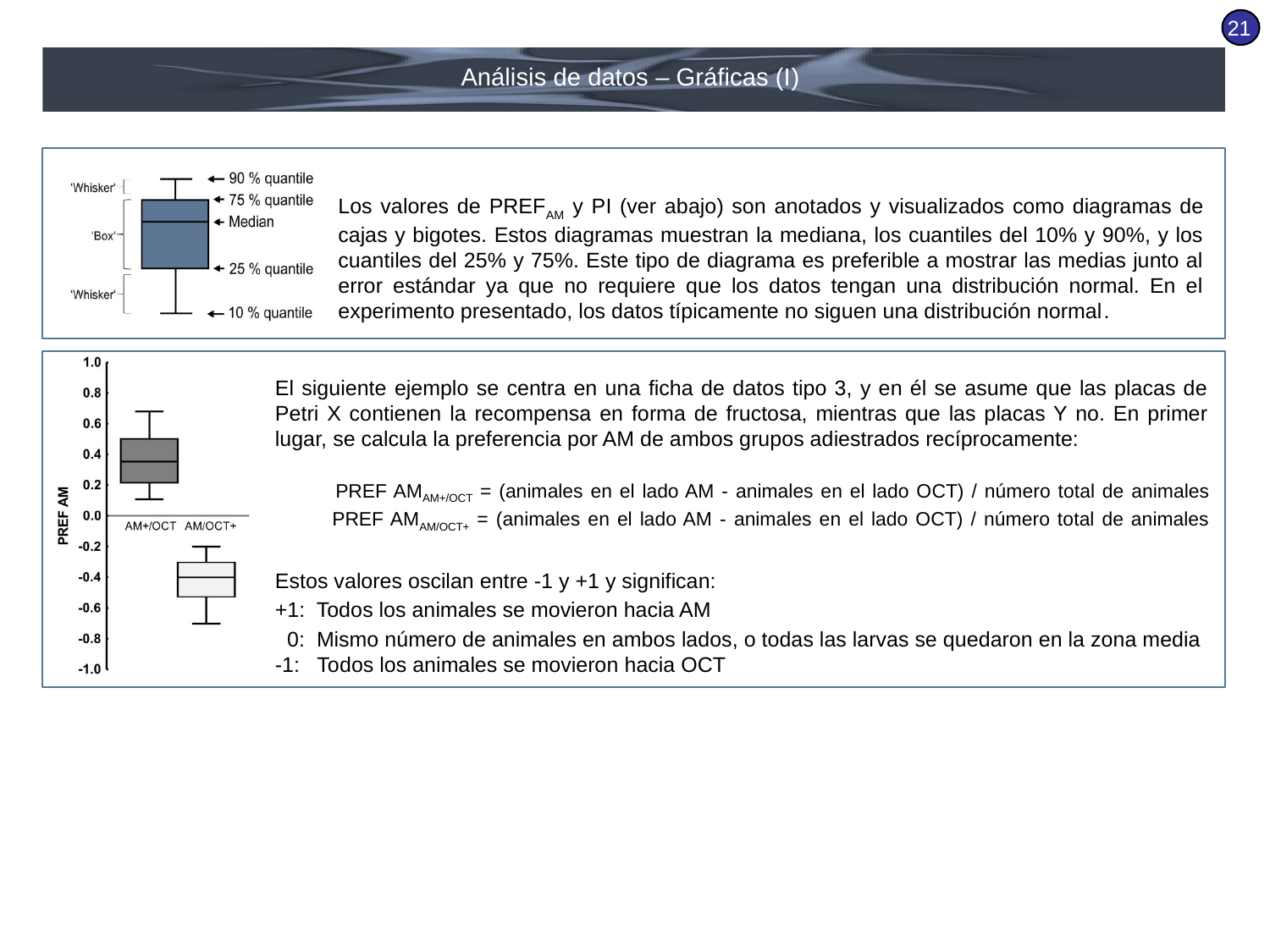

21
Análisis de datos – Gráficas (I)
Los valores de PREFAM y PI (ver abajo) son anotados y visualizados como diagramas de cajas y bigotes. Estos diagramas muestran la mediana, los cuantiles del 10% y 90%, y los cuantiles del 25% y 75%. Este tipo de diagrama es preferible a mostrar las medias junto al error estándar ya que no requiere que los datos tengan una distribución normal. En el experimento presentado, los datos típicamente no siguen una distribución normal.
El siguiente ejemplo se centra en una ficha de datos tipo 3, y en él se asume que las placas de Petri X contienen la recompensa en forma de fructosa, mientras que las placas Y no. En primer lugar, se calcula la preferencia por AM de ambos grupos adiestrados recíprocamente:
 PREF AMAM+/OCT = (animales en el lado AM - animales en el lado OCT) / número total de animales
 PREF AMAM/OCT+ = (animales en el lado AM - animales en el lado OCT) / número total de animales
Estos valores oscilan entre -1 y +1 y significan:
+1: Todos los animales se movieron hacia AM
 0: Mismo número de animales en ambos lados, o todas las larvas se quedaron en la zona media
-1: Todos los animales se movieron hacia OCT

## Slide 22
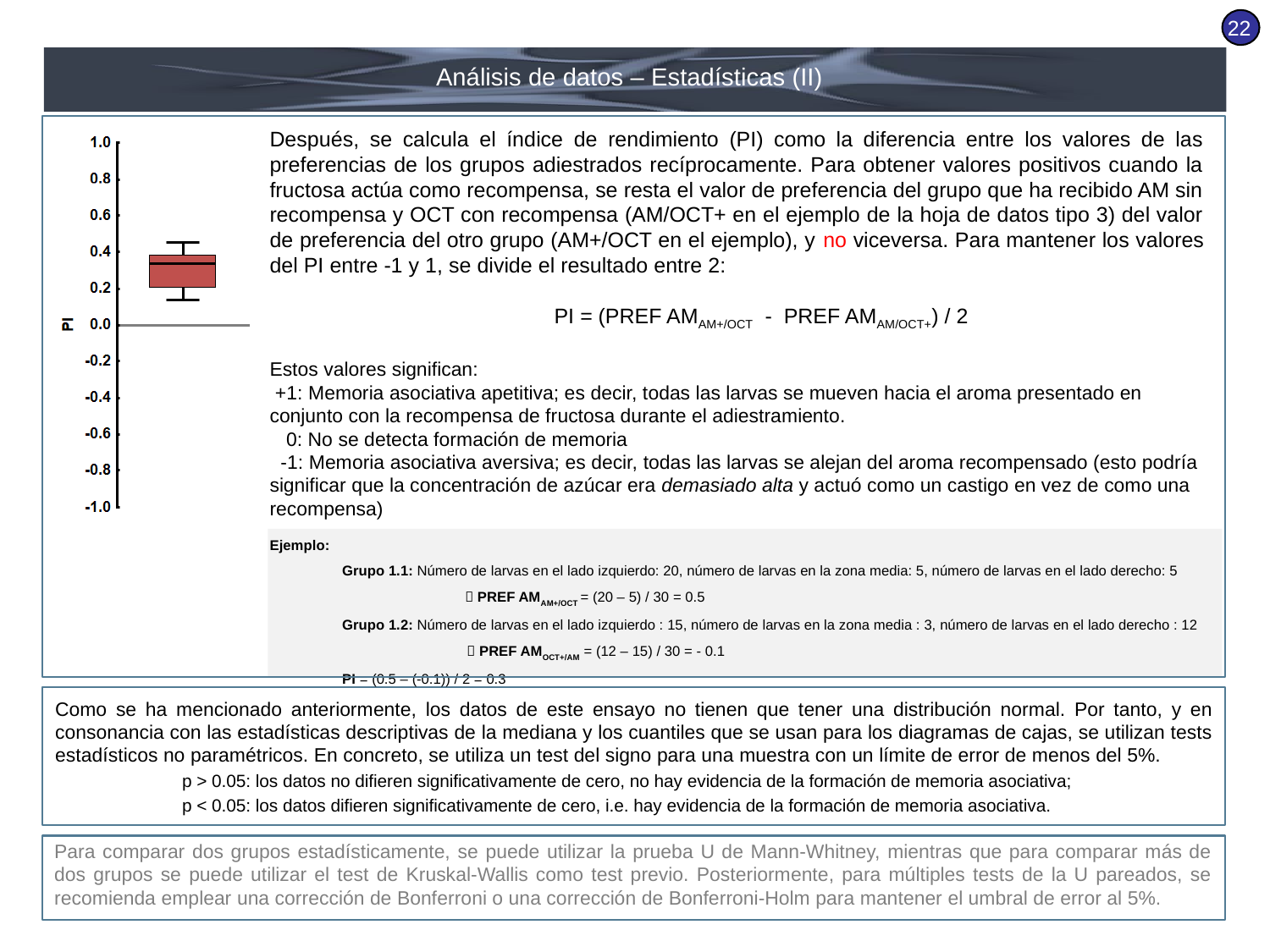

22
Análisis de datos – Estadísticas (II)
Después, se calcula el índice de rendimiento (PI) como la diferencia entre los valores de las preferencias de los grupos adiestrados recíprocamente. Para obtener valores positivos cuando la fructosa actúa como recompensa, se resta el valor de preferencia del grupo que ha recibido AM sin recompensa y OCT con recompensa (AM/OCT+ en el ejemplo de la hoja de datos tipo 3) del valor de preferencia del otro grupo (AM+/OCT en el ejemplo), y no viceversa. Para mantener los valores del PI entre -1 y 1, se divide el resultado entre 2:
 PI = (PREF AMAM+/OCT - PREF AMAM/OCT+) / 2
Estos valores significan:
 +1: Memoria asociativa apetitiva; es decir, todas las larvas se mueven hacia el aroma presentado en conjunto con la recompensa de fructosa durante el adiestramiento.
 0: No se detecta formación de memoria
 -1: Memoria asociativa aversiva; es decir, todas las larvas se alejan del aroma recompensado (esto podría significar que la concentración de azúcar era demasiado alta y actuó como un castigo en vez de como una recompensa)
Ejemplo:
 Grupo 1.1: Número de larvas en el lado izquierdo: 20, número de larvas en la zona media: 5, número de larvas en el lado derecho: 5
	  PREF AMAM+/OCT = (20 – 5) / 30 = 0.5
 Grupo 1.2: Número de larvas en el lado izquierdo : 15, número de larvas en la zona media : 3, número de larvas en el lado derecho : 12
  PREF AMOCT+/AM = (12 – 15) / 30 = - 0.1
 PI = (0.5 – (-0.1)) / 2 = 0.3
Como se ha mencionado anteriormente, los datos de este ensayo no tienen que tener una distribución normal. Por tanto, y en consonancia con las estadísticas descriptivas de la mediana y los cuantiles que se usan para los diagramas de cajas, se utilizan tests estadísticos no paramétricos. En concreto, se utiliza un test del signo para una muestra con un límite de error de menos del 5%.
	p > 0.05: los datos no difieren significativamente de cero, no hay evidencia de la formación de memoria asociativa;
	p < 0.05: los datos difieren significativamente de cero, i.e. hay evidencia de la formación de memoria asociativa.
Para comparar dos grupos estadísticamente, se puede utilizar la prueba U de Mann-Whitney, mientras que para comparar más de dos grupos se puede utilizar el test de Kruskal-Wallis como test previo. Posteriormente, para múltiples tests de la U pareados, se recomienda emplear una corrección de Bonferroni o una corrección de Bonferroni-Holm para mantener el umbral de error al 5%.

## Slide 23
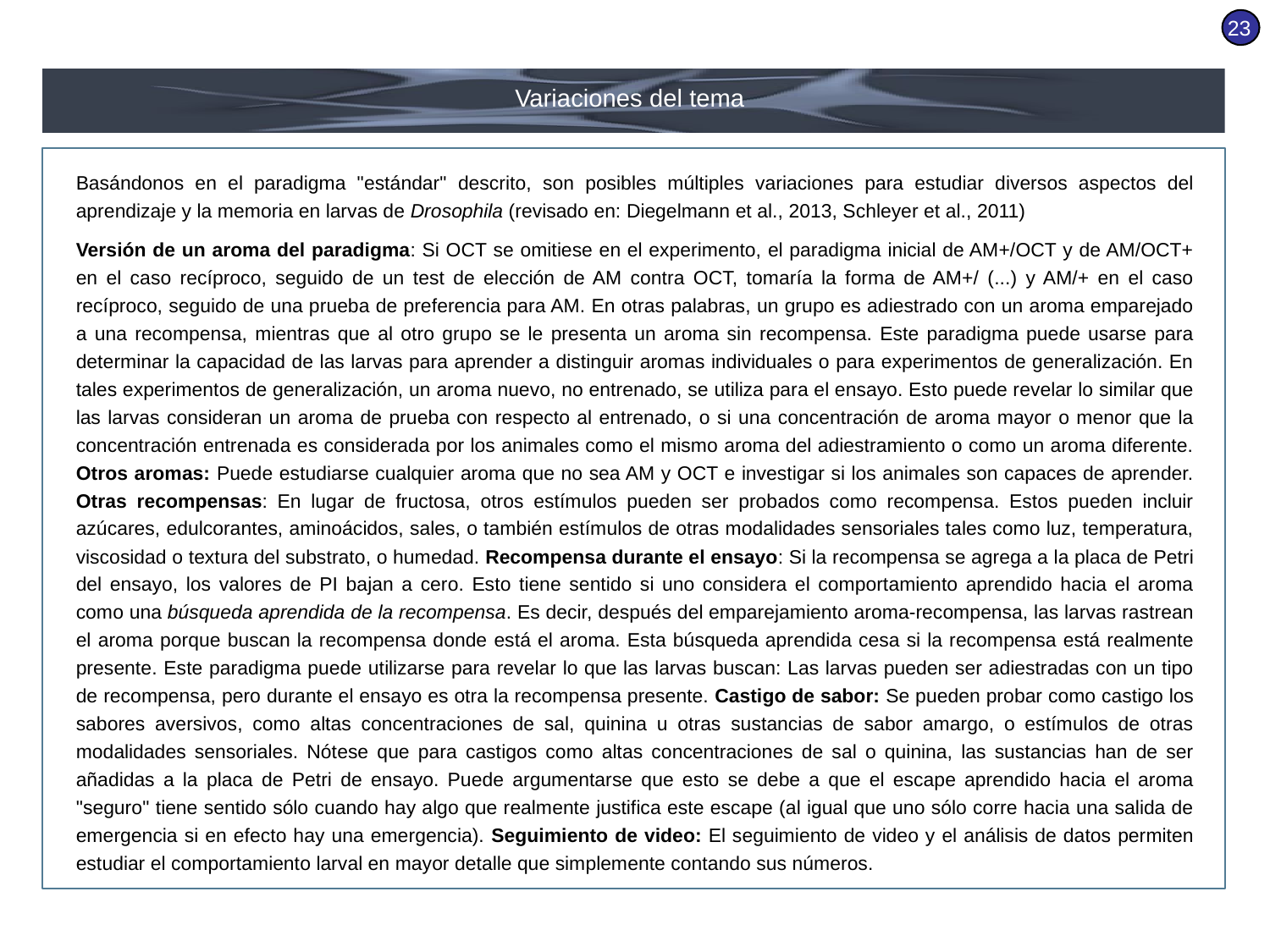

23
Variaciones del tema
Basándonos en el paradigma "estándar" descrito, son posibles múltiples variaciones para estudiar diversos aspectos del aprendizaje y la memoria en larvas de Drosophila (revisado en: Diegelmann et al., 2013, Schleyer et al., 2011)
Versión de un aroma del paradigma: Si OCT se omitiese en el experimento, el paradigma inicial de AM+/OCT y de AM/OCT+ en el caso recíproco, seguido de un test de elección de AM contra OCT, tomaría la forma de AM+/ (...) y AM/+ en el caso recíproco, seguido de una prueba de preferencia para AM. En otras palabras, un grupo es adiestrado con un aroma emparejado a una recompensa, mientras que al otro grupo se le presenta un aroma sin recompensa. Este paradigma puede usarse para determinar la capacidad de las larvas para aprender a distinguir aromas individuales o para experimentos de generalización. En tales experimentos de generalización, un aroma nuevo, no entrenado, se utiliza para el ensayo. Esto puede revelar lo similar que las larvas consideran un aroma de prueba con respecto al entrenado, o si una concentración de aroma mayor o menor que la concentración entrenada es considerada por los animales como el mismo aroma del adiestramiento o como un aroma diferente. Otros aromas: Puede estudiarse cualquier aroma que no sea AM y OCT e investigar si los animales son capaces de aprender. Otras recompensas: En lugar de fructosa, otros estímulos pueden ser probados como recompensa. Estos pueden incluir azúcares, edulcorantes, aminoácidos, sales, o también estímulos de otras modalidades sensoriales tales como luz, temperatura, viscosidad o textura del substrato, o humedad. Recompensa durante el ensayo: Si la recompensa se agrega a la placa de Petri del ensayo, los valores de PI bajan a cero. Esto tiene sentido si uno considera el comportamiento aprendido hacia el aroma como una búsqueda aprendida de la recompensa. Es decir, después del emparejamiento aroma-recompensa, las larvas rastrean el aroma porque buscan la recompensa donde está el aroma. Esta búsqueda aprendida cesa si la recompensa está realmente presente. Este paradigma puede utilizarse para revelar lo que las larvas buscan: Las larvas pueden ser adiestradas con un tipo de recompensa, pero durante el ensayo es otra la recompensa presente. Castigo de sabor: Se pueden probar como castigo los sabores aversivos, como altas concentraciones de sal, quinina u otras sustancias de sabor amargo, o estímulos de otras modalidades sensoriales. Nótese que para castigos como altas concentraciones de sal o quinina, las sustancias han de ser añadidas a la placa de Petri de ensayo. Puede argumentarse que esto se debe a que el escape aprendido hacia el aroma "seguro" tiene sentido sólo cuando hay algo que realmente justifica este escape (al igual que uno sólo corre hacia una salida de emergencia si en efecto hay una emergencia). Seguimiento de video: El seguimiento de video y el análisis de datos permiten estudiar el comportamiento larval en mayor detalle que simplemente contando sus números.

## Slide 24
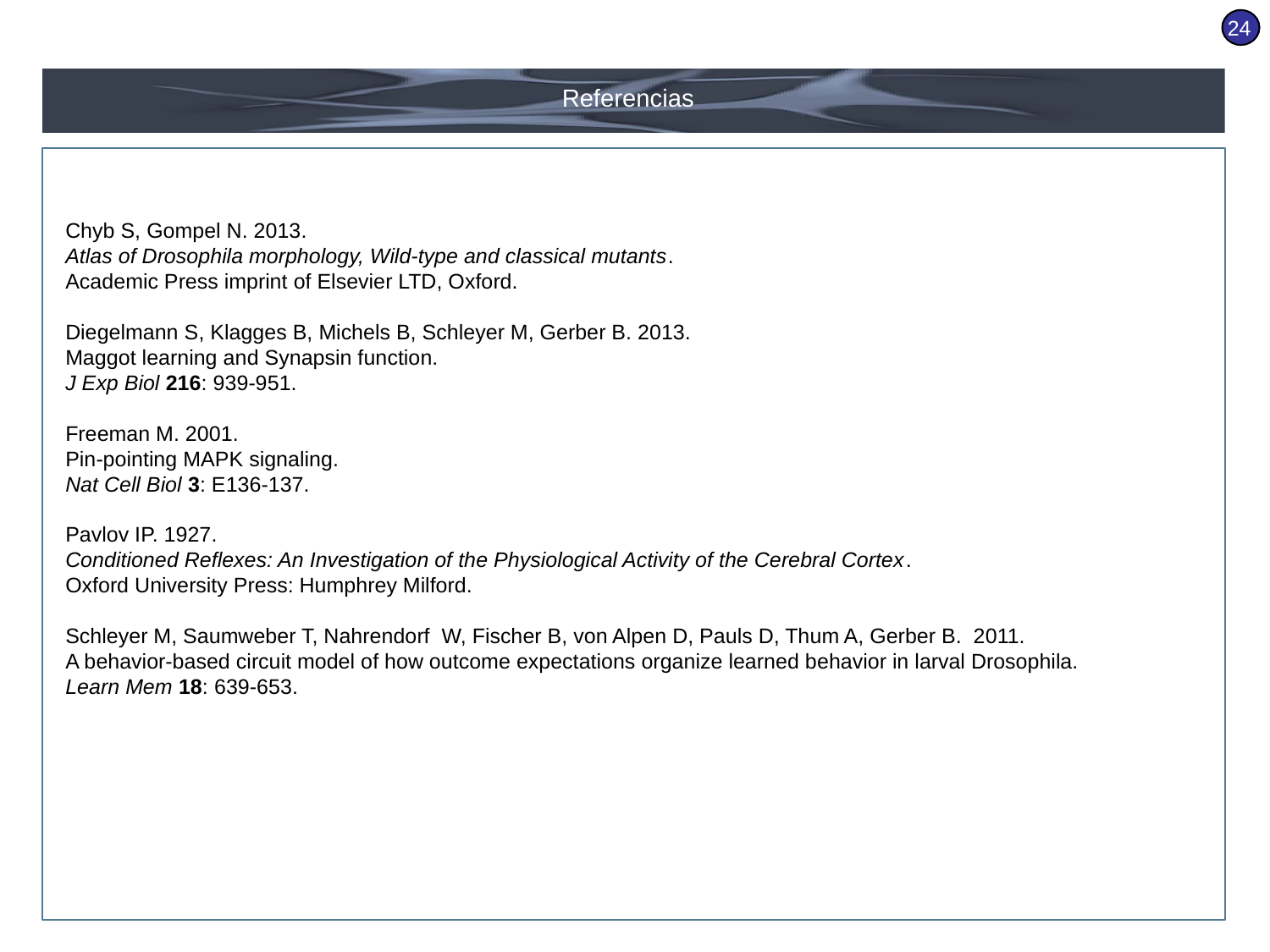

24
Referencias
Chyb S, Gompel N. 2013.
Atlas of Drosophila morphology, Wild-type and classical mutants.
Academic Press imprint of Elsevier LTD, Oxford.
Diegelmann S, Klagges B, Michels B, Schleyer M, Gerber B. 2013.
Maggot learning and Synapsin function.
J Exp Biol 216: 939-951.
Freeman M. 2001.
Pin-pointing MAPK signaling.
Nat Cell Biol 3: E136-137.
Pavlov IP. 1927.
Conditioned Reflexes: An Investigation of the Physiological Activity of the Cerebral Cortex.
Oxford University Press: Humphrey Milford.
Schleyer M, Saumweber T, Nahrendorf W, Fischer B, von Alpen D, Pauls D, Thum A, Gerber B. 2011.
A behavior-based circuit model of how outcome expectations organize learned behavior in larval Drosophila.
Learn Mem 18: 639-653.
