# Supplementary material for: Pavlovian Conditioning of Larval Drosophila: An Illustrated, Multilingual, Hands-On Manual for Odor-Taste Associative Learning in Maggots
Source: Front Behav Neurosci. 2017 Apr 19;11:45. doi: 10.3389/fnbeh.2017.00045 (PMC5395560; doi:10.3389/fnbeh.2017.00045)
Supplement: Supplemental Materials 14–16 — A manual for odor-reward learning in larval Drosophila (Supplemental Material 14), example of a table for data analysis (Supplemental Material 15), and an empty table for entering and analyzing one's own data (Supplemental Material 16), in the Italian language. Versions of this manual in the English, German, French, Japanese, and Spanish languages can be found in Supplemental Materials 1–3, 4–6, 7–9, 10, and 11–13, respectively. [file SupplementalMaterial14.pptx]

## Slide 1
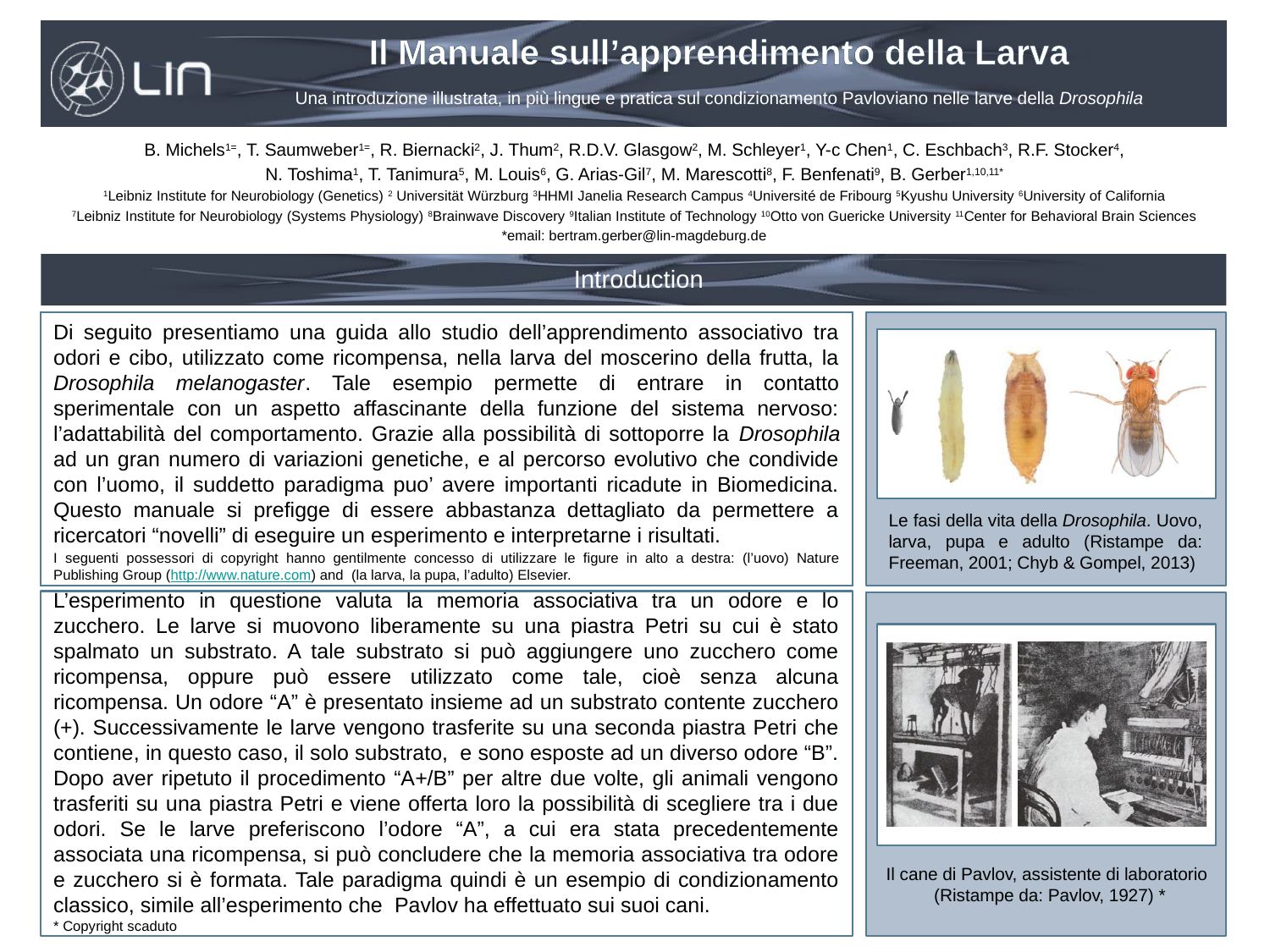

# Una introduzione illustrata, in più lingue e pratica sul condizionamento Pavloviano nelle larve della Drosophila
Il Manuale sull’apprendimento della Larva
B. Michels1=, T. Saumweber1=, R. Biernacki2, J. Thum2, R.D.V. Glasgow2, M. Schleyer1, Y-c Chen1, C. Eschbach3, R.F. Stocker4,
N. Toshima1, T. Tanimura5, M. Louis6, G. Arias-Gil7, M. Marescotti8, F. Benfenati9, B. Gerber1,10,11*
1Leibniz Institute for Neurobiology (Genetics) 2 Universität Würzburg 3HHMI Janelia Research Campus 4Université de Fribourg 5Kyushu University 6University of California
7Leibniz Institute for Neurobiology (Systems Physiology) 8Brainwave Discovery 9Italian Institute of Technology 10Otto von Guericke University 11Center for Behavioral Brain Sciences
*email: bertram.gerber@lin-magdeburg.de
Introduction
Di seguito presentiamo una guida allo studio dell’apprendimento associativo tra odori e cibo, utilizzato come ricompensa, nella larva del moscerino della frutta, la Drosophila melanogaster. Tale esempio permette di entrare in contatto sperimentale con un aspetto affascinante della funzione del sistema nervoso: l’adattabilità del comportamento. Grazie alla possibilità di sottoporre la Drosophila ad un gran numero di variazioni genetiche, e al percorso evolutivo che condivide con l’uomo, il suddetto paradigma puo’ avere importanti ricadute in Biomedicina. Questo manuale si prefigge di essere abbastanza dettagliato da permettere a ricercatori “novelli” di eseguire un esperimento e interpretarne i risultati.
I seguenti possessori di copyright hanno gentilmente concesso di utilizzare le figure in alto a destra: (l’uovo) Nature Publishing Group (http://www.nature.com) and (la larva, la pupa, l’adulto) Elsevier.
Le fasi della vita della Drosophila. Uovo, larva, pupa e adulto (Ristampe da: Freeman, 2001; Chyb & Gompel, 2013)
L’esperimento in questione valuta la memoria associativa tra un odore e lo zucchero. Le larve si muovono liberamente su una piastra Petri su cui è stato spalmato un substrato. A tale substrato si può aggiungere uno zucchero come ricompensa, oppure può essere utilizzato come tale, cioè senza alcuna ricompensa. Un odore “A” è presentato insieme ad un substrato contente zucchero (+). Successivamente le larve vengono trasferite su una seconda piastra Petri che contiene, in questo caso, il solo substrato, e sono esposte ad un diverso odore “B”. Dopo aver ripetuto il procedimento “A+/B” per altre due volte, gli animali vengono trasferiti su una piastra Petri e viene offerta loro la possibilità di scegliere tra i due odori. Se le larve preferiscono l’odore “A”, a cui era stata precedentemente associata una ricompensa, si può concludere che la memoria associativa tra odore e zucchero si è formata. Tale paradigma quindi è un esempio di condizionamento classico, simile all’esperimento che Pavlov ha effettuato sui suoi cani.
* Copyright scaduto
Il cane di Pavlov, assistente di laboratorio (Ristampe da: Pavlov, 1927) *

## Slide 2
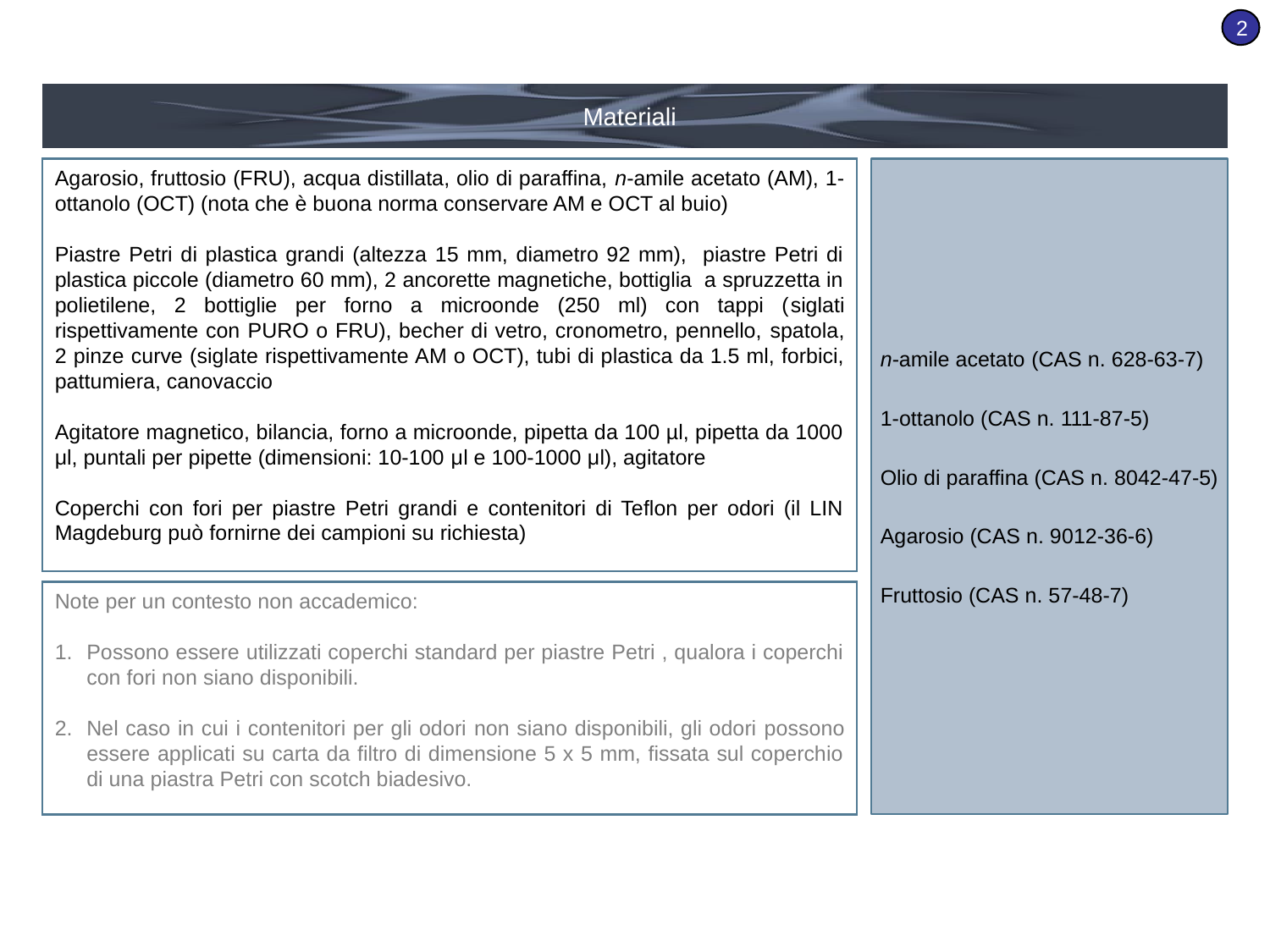

2
Materiali
Agarosio, fruttosio (FRU), acqua distillata, olio di paraffina, n-amile acetato (AM), 1-ottanolo (OCT) (nota che è buona norma conservare AM e OCT al buio)
Piastre Petri di plastica grandi (altezza 15 mm, diametro 92 mm), piastre Petri di plastica piccole (diametro 60 mm), 2 ancorette magnetiche, bottiglia a spruzzetta in polietilene, 2 bottiglie per forno a microonde (250 ml) con tappi (siglati rispettivamente con PURO o FRU), becher di vetro, cronometro, pennello, spatola, 2 pinze curve (siglate rispettivamente AM o OCT), tubi di plastica da 1.5 ml, forbici, pattumiera, canovaccio
Agitatore magnetico, bilancia, forno a microonde, pipetta da 100 µl, pipetta da 1000 μl, puntali per pipette (dimensioni: 10-100 μl e 100-1000 μl), agitatore
Coperchi con fori per piastre Petri grandi e contenitori di Teflon per odori (il LIN Magdeburg può fornirne dei campioni su richiesta)
n-amile acetato (CAS n. 628-63-7)
1-ottanolo (CAS n. 111-87-5)
Olio di paraffina (CAS n. 8042-47-5)
Agarosio (CAS n. 9012-36-6)
Fruttosio (CAS n. 57-48-7)
Note per un contesto non accademico:
Possono essere utilizzati coperchi standard per piastre Petri , qualora i coperchi con fori non siano disponibili.
Nel caso in cui i contenitori per gli odori non siano disponibili, gli odori possono essere applicati su carta da filtro di dimensione 5 x 5 mm, fissata sul coperchio di una piastra Petri con scotch biadesivo.

## Slide 3
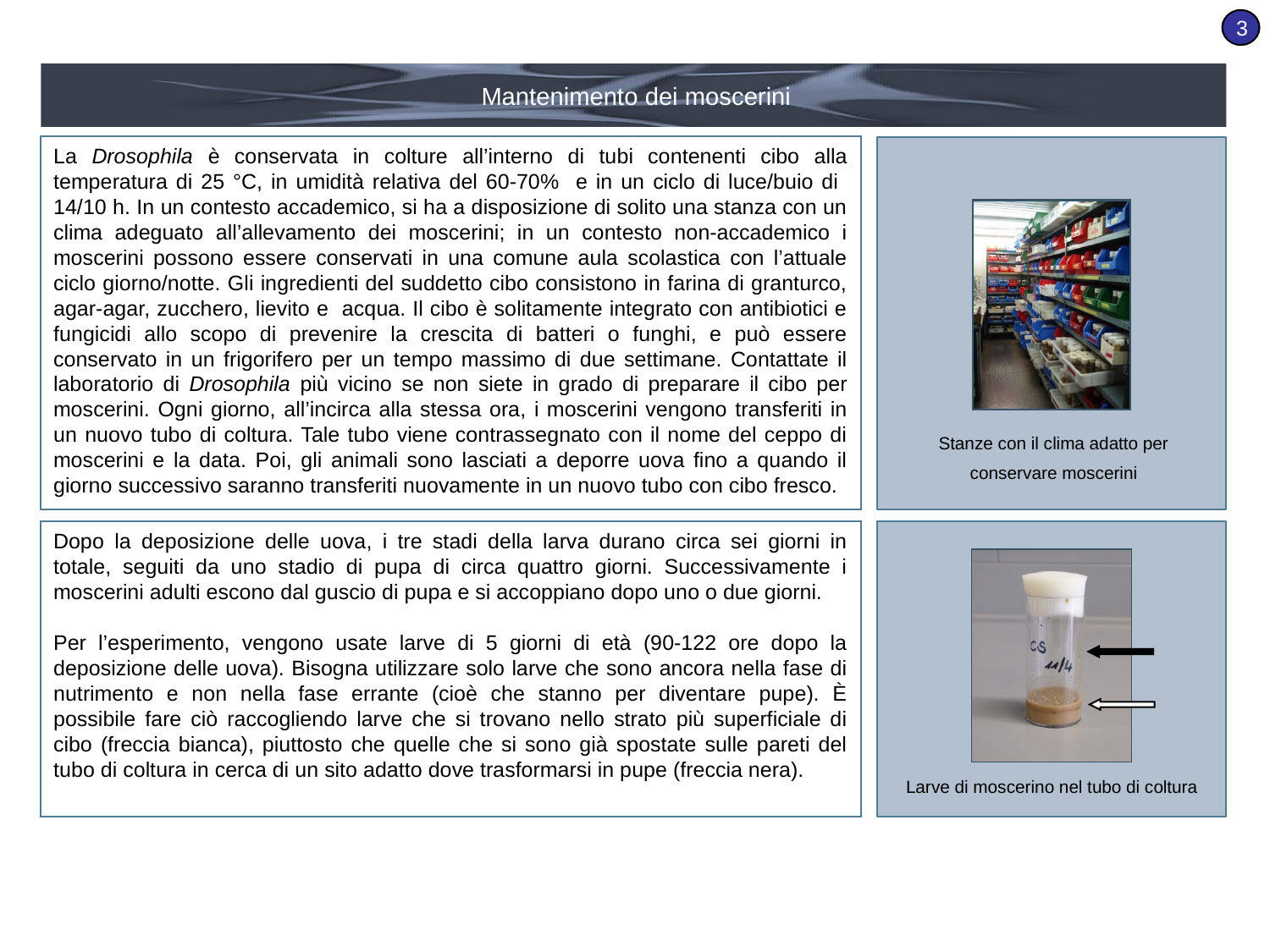

3
# Mantenimento dei moscerini
La Drosophila è conservata in colture all’interno di tubi contenenti cibo alla temperatura di 25 °C, in umidità relativa del 60-70% e in un ciclo di luce/buio di 14/10 h. In un contesto accademico, si ha a disposizione di solito una stanza con un clima adeguato all’allevamento dei moscerini; in un contesto non-accademico i moscerini possono essere conservati in una comune aula scolastica con l’attuale ciclo giorno/notte. Gli ingredienti del suddetto cibo consistono in farina di granturco, agar-agar, zucchero, lievito e acqua. Il cibo è solitamente integrato con antibiotici e fungicidi allo scopo di prevenire la crescita di batteri o funghi, e può essere conservato in un frigorifero per un tempo massimo di due settimane. Contattate il laboratorio di Drosophila più vicino se non siete in grado di preparare il cibo per moscerini. Ogni giorno, all’incirca alla stessa ora, i moscerini vengono transferiti in un nuovo tubo di coltura. Tale tubo viene contrassegnato con il nome del ceppo di moscerini e la data. Poi, gli animali sono lasciati a deporre uova fino a quando il giorno successivo saranno transferiti nuovamente in un nuovo tubo con cibo fresco.
Stanze con il clima adatto per
conservare moscerini
Dopo la deposizione delle uova, i tre stadi della larva durano circa sei giorni in totale, seguiti da uno stadio di pupa di circa quattro giorni. Successivamente i moscerini adulti escono dal guscio di pupa e si accoppiano dopo uno o due giorni.
Per l’esperimento, vengono usate larve di 5 giorni di età (90-122 ore dopo la deposizione delle uova). Bisogna utilizzare solo larve che sono ancora nella fase di nutrimento e non nella fase errante (cioè che stanno per diventare pupe). È possibile fare ciò raccogliendo larve che si trovano nello strato più superficiale di cibo (freccia bianca), piuttosto che quelle che si sono già spostate sulle pareti del tubo di coltura in cerca di un sito adatto dove trasformarsi in pupe (freccia nera).
Larve di moscerino nel tubo di coltura

## Slide 4
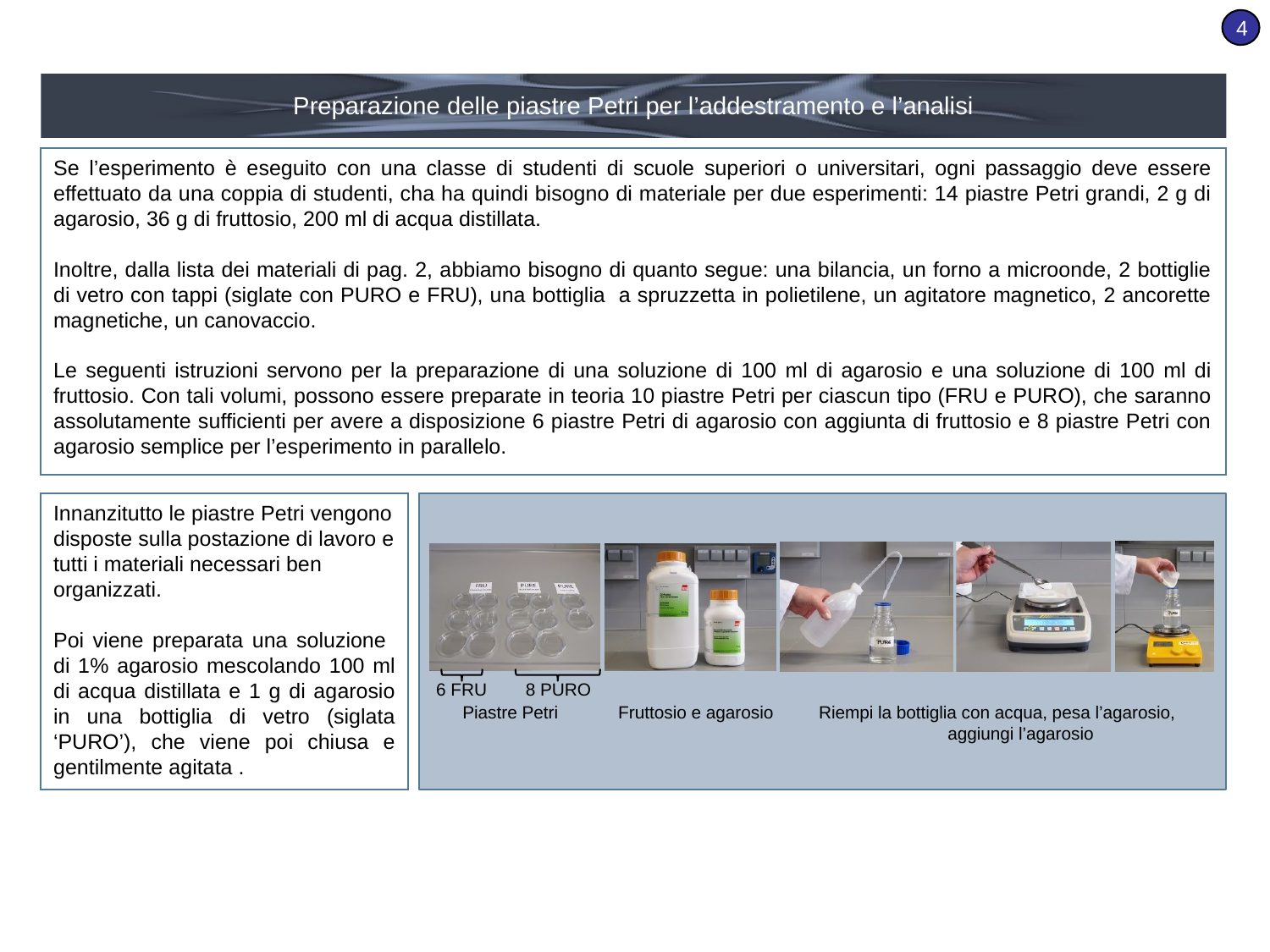

4
# Preparazione delle piastre Petri per l’addestramento e l’analisi
Se l’esperimento è eseguito con una classe di studenti di scuole superiori o universitari, ogni passaggio deve essere effettuato da una coppia di studenti, cha ha quindi bisogno di materiale per due esperimenti: 14 piastre Petri grandi, 2 g di agarosio, 36 g di fruttosio, 200 ml di acqua distillata.
Inoltre, dalla lista dei materiali di pag. 2, abbiamo bisogno di quanto segue: una bilancia, un forno a microonde, 2 bottiglie di vetro con tappi (siglate con PURO e FRU), una bottiglia a spruzzetta in polietilene, un agitatore magnetico, 2 ancorette magnetiche, un canovaccio.
Le seguenti istruzioni servono per la preparazione di una soluzione di 100 ml di agarosio e una soluzione di 100 ml di fruttosio. Con tali volumi, possono essere preparate in teoria 10 piastre Petri per ciascun tipo (FRU e PURO), che saranno assolutamente sufficienti per avere a disposizione 6 piastre Petri di agarosio con aggiunta di fruttosio e 8 piastre Petri con agarosio semplice per l’esperimento in parallelo.
Innanzitutto le piastre Petri vengono disposte sulla postazione di lavoro e tutti i materiali necessari ben organizzati.
Poi viene preparata una soluzione di 1% agarosio mescolando 100 ml di acqua distillata e 1 g di agarosio in una bottiglia di vetro (siglata ‘PURO’), che viene poi chiusa e gentilmente agitata .
6 FRU 8 PURO
Fruttosio e agarosio
Piastre Petri
Riempi la bottiglia con acqua, pesa l’agarosio, aggiungi l’agarosio

## Slide 5
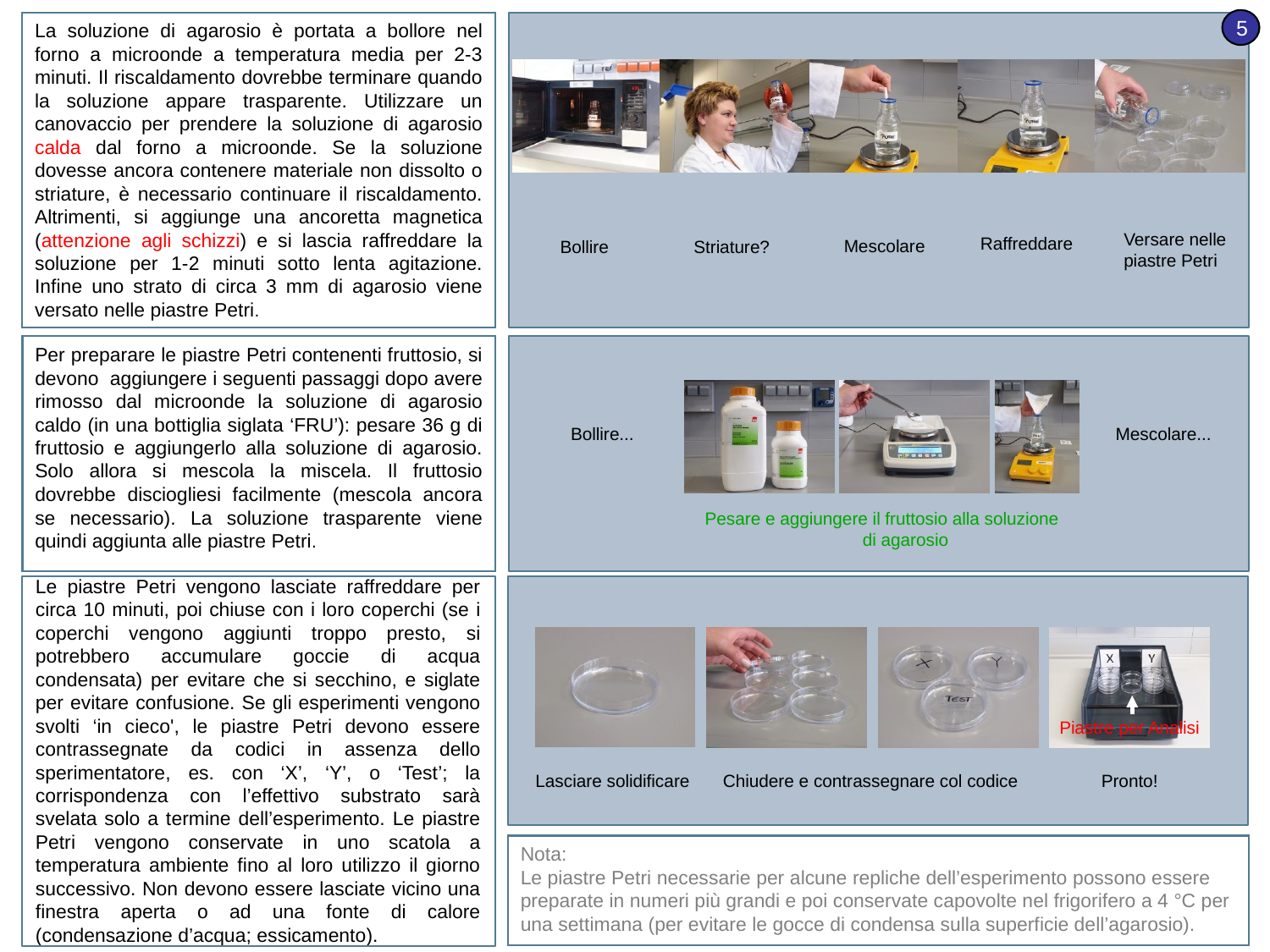

5
La soluzione di agarosio è portata a bollore nel forno a microonde a temperatura media per 2-3 minuti. Il riscaldamento dovrebbe terminare quando la soluzione appare trasparente. Utilizzare un canovaccio per prendere la soluzione di agarosio calda dal forno a microonde. Se la soluzione dovesse ancora contenere materiale non dissolto o striature, è necessario continuare il riscaldamento. Altrimenti, si aggiunge una ancoretta magnetica (attenzione agli schizzi) e si lascia raffreddare la soluzione per 1-2 minuti sotto lenta agitazione. Infine uno strato di circa 3 mm di agarosio viene versato nelle piastre Petri.
Versare nelle piastre Petri
Striature?
Raffreddare
Mescolare
Bollire
Per preparare le piastre Petri contenenti fruttosio, si devono aggiungere i seguenti passaggi dopo avere rimosso dal microonde la soluzione di agarosio caldo (in una bottiglia siglata ‘FRU’): pesare 36 g di fruttosio e aggiungerlo alla soluzione di agarosio. Solo allora si mescola la miscela. Il fruttosio dovrebbe disciogliesi facilmente (mescola ancora se necessario). La soluzione trasparente viene quindi aggiunta alle piastre Petri.
Bollire...
Mescolare...
Pesare e aggiungere il fruttosio alla soluzione di agarosio
Le piastre Petri vengono lasciate raffreddare per circa 10 minuti, poi chiuse con i loro coperchi (se i coperchi vengono aggiunti troppo presto, si potrebbero accumulare goccie di acqua condensata) per evitare che si secchino, e siglate per evitare confusione. Se gli esperimenti vengono svolti ‘in cieco', le piastre Petri devono essere contrassegnate da codici in assenza dello sperimentatore, es. con ‘X’, ‘Y’, o ‘Test’; la corrispondenza con l’effettivo substrato sarà svelata solo a termine dell’esperimento. Le piastre Petri vengono conservate in uno scatola a temperatura ambiente fino al loro utilizzo il giorno successivo. Non devono essere lasciate vicino una finestra aperta o ad una fonte di calore (condensazione d’acqua; essicamento).
Piastre per Analisi
Lasciare solidificare
Chiudere e contrassegnare col codice
Pronto!
Nota:
Le piastre Petri necessarie per alcune repliche dell’esperimento possono essere preparate in numeri più grandi e poi conservate capovolte nel frigorifero a 4 °C per una settimana (per evitare le gocce di condensa sulla superficie dell’agarosio).

## Slide 6
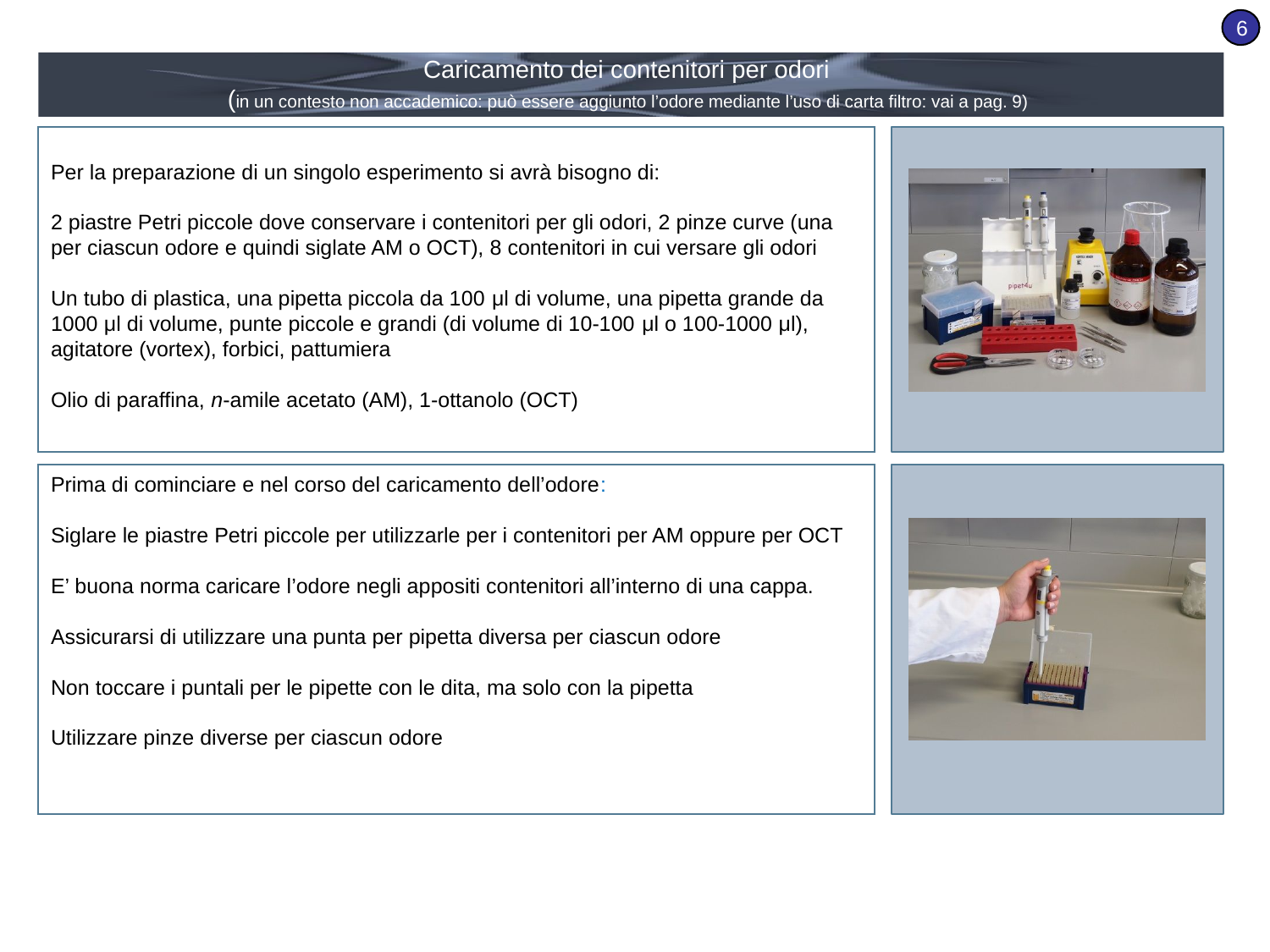

6
# Caricamento dei contenitori per odori (in un contesto non accademico: può essere aggiunto l’odore mediante l’uso di carta filtro: vai a pag. 9)
Per la preparazione di un singolo esperimento si avrà bisogno di:
2 piastre Petri piccole dove conservare i contenitori per gli odori, 2 pinze curve (una per ciascun odore e quindi siglate AM o OCT), 8 contenitori in cui versare gli odori
Un tubo di plastica, una pipetta piccola da 100 μl di volume, una pipetta grande da 1000 μl di volume, punte piccole e grandi (di volume di 10-100 μl o 100-1000 μl), agitatore (vortex), forbici, pattumiera
 
Olio di paraffina, n-amile acetato (AM), 1-ottanolo (OCT)
Prima di cominciare e nel corso del caricamento dell’odore:
Siglare le piastre Petri piccole per utilizzarle per i contenitori per AM oppure per OCT
E’ buona norma caricare l’odore negli appositi contenitori all’interno di una cappa.
Assicurarsi di utilizzare una punta per pipetta diversa per ciascun odore
Non toccare i puntali per le pipette con le dita, ma solo con la pipetta
Utilizzare pinze diverse per ciascun odore

## Slide 7
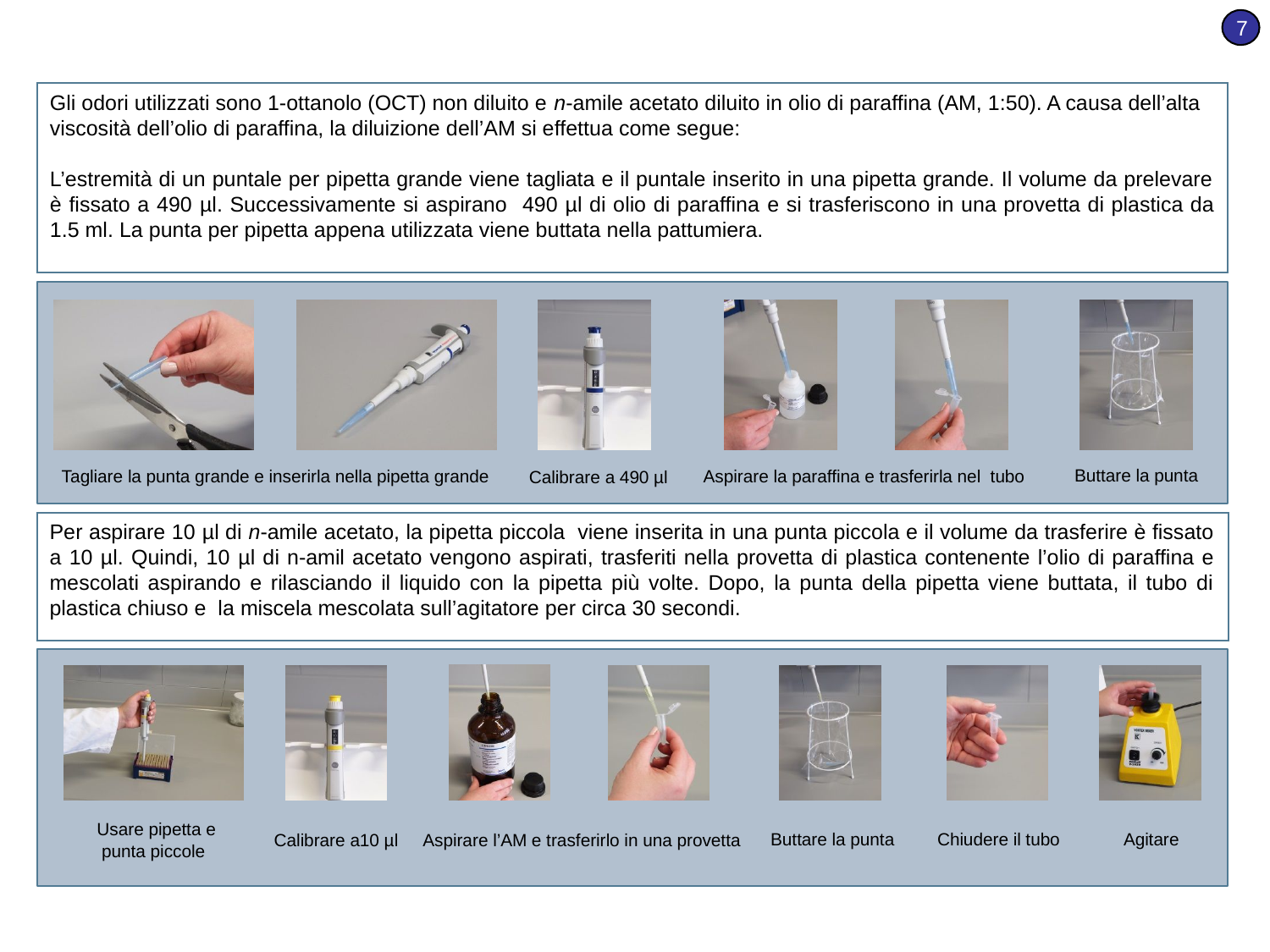

7
Gli odori utilizzati sono 1-ottanolo (OCT) non diluito e n-amile acetato diluito in olio di paraffina (AM, 1:50). A causa dell’alta viscosità dell’olio di paraffina, la diluizione dell’AM si effettua come segue:
L’estremità di un puntale per pipetta grande viene tagliata e il puntale inserito in una pipetta grande. Il volume da prelevare è fissato a 490 µl. Successivamente si aspirano 490 µl di olio di paraffina e si trasferiscono in una provetta di plastica da 1.5 ml. La punta per pipetta appena utilizzata viene buttata nella pattumiera.
Buttare la punta
Tagliare la punta grande e inserirla nella pipetta grande
Aspirare la paraffina e trasferirla nel tubo
Calibrare a 490 µl
Per aspirare 10 µl di n-amile acetato, la pipetta piccola viene inserita in una punta piccola e il volume da trasferire è fissato a 10 µl. Quindi, 10 µl di n-amil acetato vengono aspirati, trasferiti nella provetta di plastica contenente l’olio di paraffina e mescolati aspirando e rilasciando il liquido con la pipetta più volte. Dopo, la punta della pipetta viene buttata, il tubo di plastica chiuso e la miscela mescolata sull’agitatore per circa 30 secondi.
 Usare pipetta e
punta piccole
Buttare la punta
Chiudere il tubo
Agitare
Calibrare a10 µl
Aspirare l’AM e trasferirlo in una provetta

## Slide 8
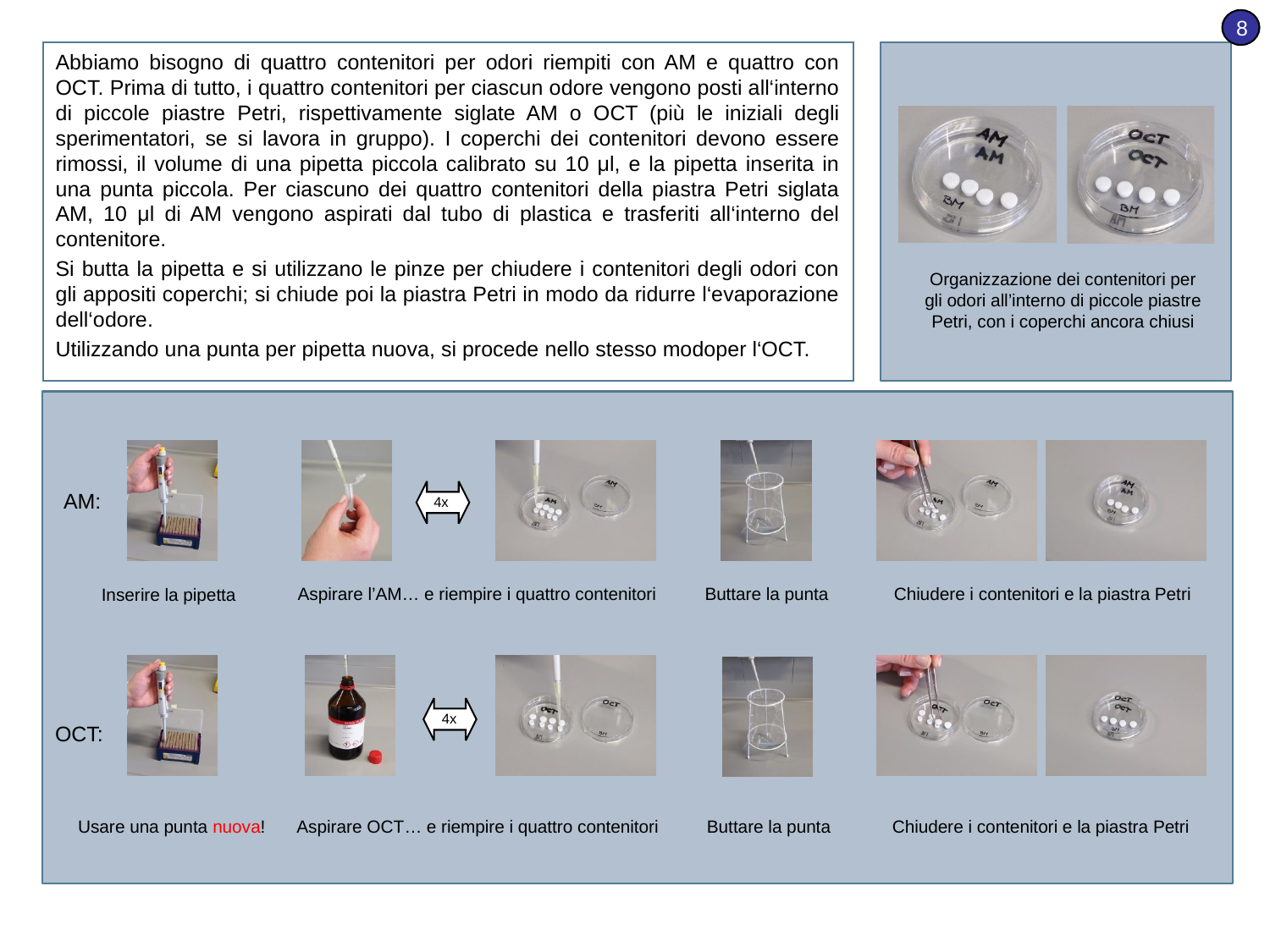

8
Abbiamo bisogno di quattro contenitori per odori riempiti con AM e quattro con OCT. Prima di tutto, i quattro contenitori per ciascun odore vengono posti all‘interno di piccole piastre Petri, rispettivamente siglate AM o OCT (più le iniziali degli sperimentatori, se si lavora in gruppo). I coperchi dei contenitori devono essere rimossi, il volume di una pipetta piccola calibrato su 10 μl, e la pipetta inserita in una punta piccola. Per ciascuno dei quattro contenitori della piastra Petri siglata AM, 10 μl di AM vengono aspirati dal tubo di plastica e trasferiti all‘interno del contenitore.
Si butta la pipetta e si utilizzano le pinze per chiudere i contenitori degli odori con gli appositi coperchi; si chiude poi la piastra Petri in modo da ridurre l‘evaporazione dell‘odore.
Utilizzando una punta per pipetta nuova, si procede nello stesso modoper l‘OCT.
Organizzazione dei contenitori per gli odori all’interno di piccole piastre Petri, con i coperchi ancora chiusi
4x
AM:
Aspirare l’AM… e riempire i quattro contenitori
Buttare la punta
Chiudere i contenitori e la piastra Petri
Inserire la pipetta
4x
OCT:
Usare una punta nuova!
Aspirare OCT… e riempire i quattro contenitori
Buttare la punta
Chiudere i contenitori e la piastra Petri

## Slide 9
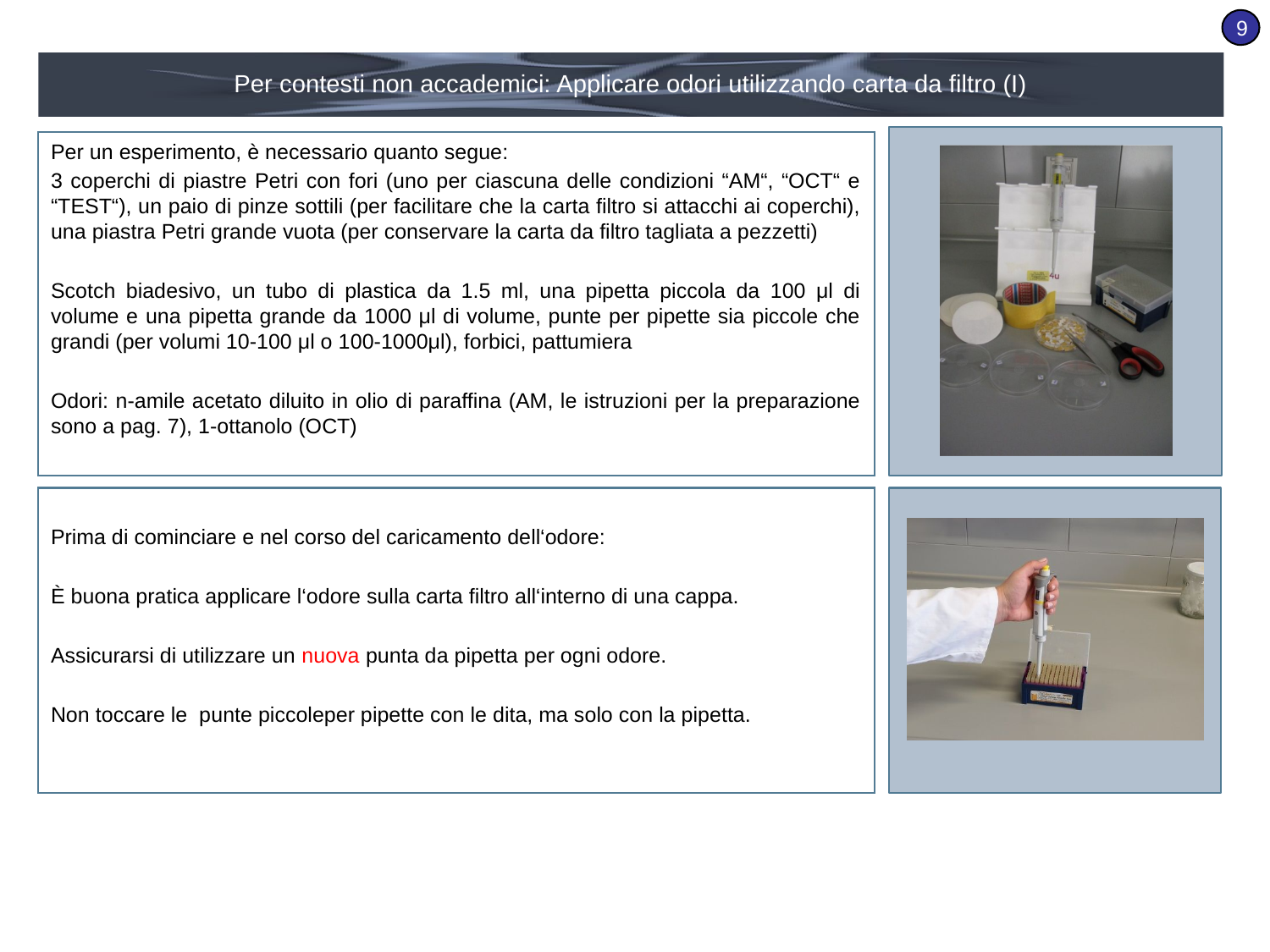

9
# Per contesti non accademici: Applicare odori utilizzando carta da filtro (I)
Per un esperimento, è necessario quanto segue:
3 coperchi di piastre Petri con fori (uno per ciascuna delle condizioni “AM“, “OCT“ e “TEST“), un paio di pinze sottili (per facilitare che la carta filtro si attacchi ai coperchi), una piastra Petri grande vuota (per conservare la carta da filtro tagliata a pezzetti)
Scotch biadesivo, un tubo di plastica da 1.5 ml, una pipetta piccola da 100 μl di volume e una pipetta grande da 1000 μl di volume, punte per pipette sia piccole che grandi (per volumi 10-100 μl o 100-1000μl), forbici, pattumiera
Odori: n-amile acetato diluito in olio di paraffina (AM, le istruzioni per la preparazione sono a pag. 7), 1-ottanolo (OCT)
Prima di cominciare e nel corso del caricamento dell‘odore:
È buona pratica applicare l‘odore sulla carta filtro all‘interno di una cappa.
Assicurarsi di utilizzare un nuova punta da pipetta per ogni odore.
Non toccare le punte piccoleper pipette con le dita, ma solo con la pipetta.

## Slide 10
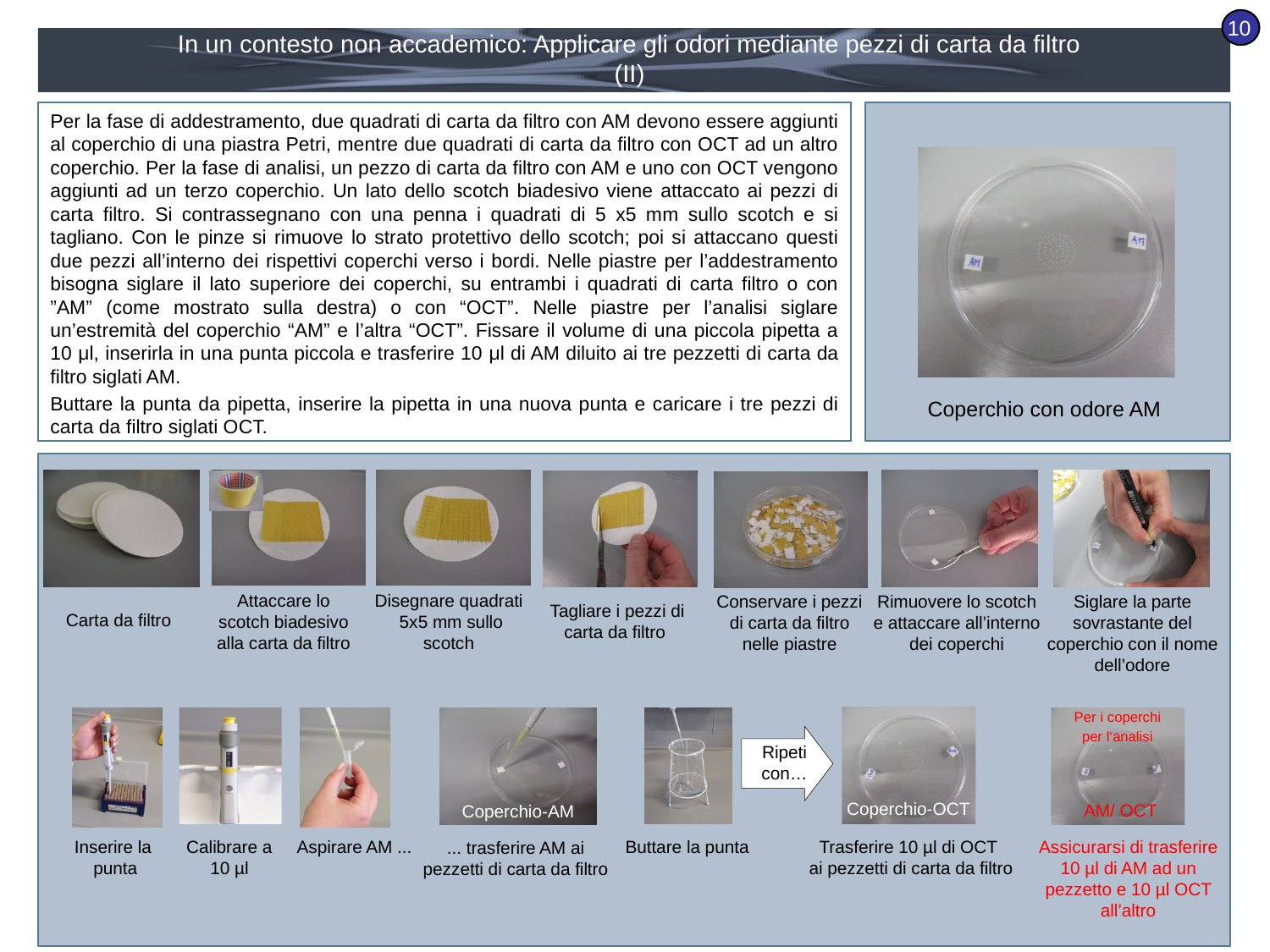

10
# In un contesto non accademico: Applicare gli odori mediante pezzi di carta da filtro (II)
Per la fase di addestramento, due quadrati di carta da filtro con AM devono essere aggiunti al coperchio di una piastra Petri, mentre due quadrati di carta da filtro con OCT ad un altro coperchio. Per la fase di analisi, un pezzo di carta da filtro con AM e uno con OCT vengono aggiunti ad un terzo coperchio. Un lato dello scotch biadesivo viene attaccato ai pezzi di carta filtro. Si contrassegnano con una penna i quadrati di 5 x5 mm sullo scotch e si tagliano. Con le pinze si rimuove lo strato protettivo dello scotch; poi si attaccano questi due pezzi all’interno dei rispettivi coperchi verso i bordi. Nelle piastre per l’addestramento bisogna siglare il lato superiore dei coperchi, su entrambi i quadrati di carta filtro o con ”AM” (come mostrato sulla destra) o con “OCT”. Nelle piastre per l’analisi siglare un’estremità del coperchio “AM” e l’altra “OCT”. Fissare il volume di una piccola pipetta a 10 μl, inserirla in una punta piccola e trasferire 10 μl di AM diluito ai tre pezzetti di carta da filtro siglati AM.
Buttare la punta da pipetta, inserire la pipetta in una nuova punta e caricare i tre pezzi di carta da filtro siglati OCT.
Coperchio con odore AM
Attaccare lo scotch biadesivo alla carta da filtro
Disegnare quadrati 5x5 mm sullo scotch
Conservare i pezzi di carta da filtro nelle piastre
Rimuovere lo scotch
e attaccare all’interno dei coperchi
Siglare la parte sovrastante del coperchio con il nome dell’odore
Tagliare i pezzi di carta da filtro
Carta da filtro
Per i coperchi
per l‘analisi
Ripeti
con…
Coperchio-OCT
AM/ OCT
Coperchio-AM
Buttare la punta
Inserire la
punta
Calibrare a 10 µl
Aspirare AM ...
Trasferire 10 µl di OCT
 ai pezzetti di carta da filtro
Assicurarsi di trasferire 10 µl di AM ad un pezzetto e 10 µl OCT all’altro
... trasferire AM ai
pezzetti di carta da filtro

## Slide 11
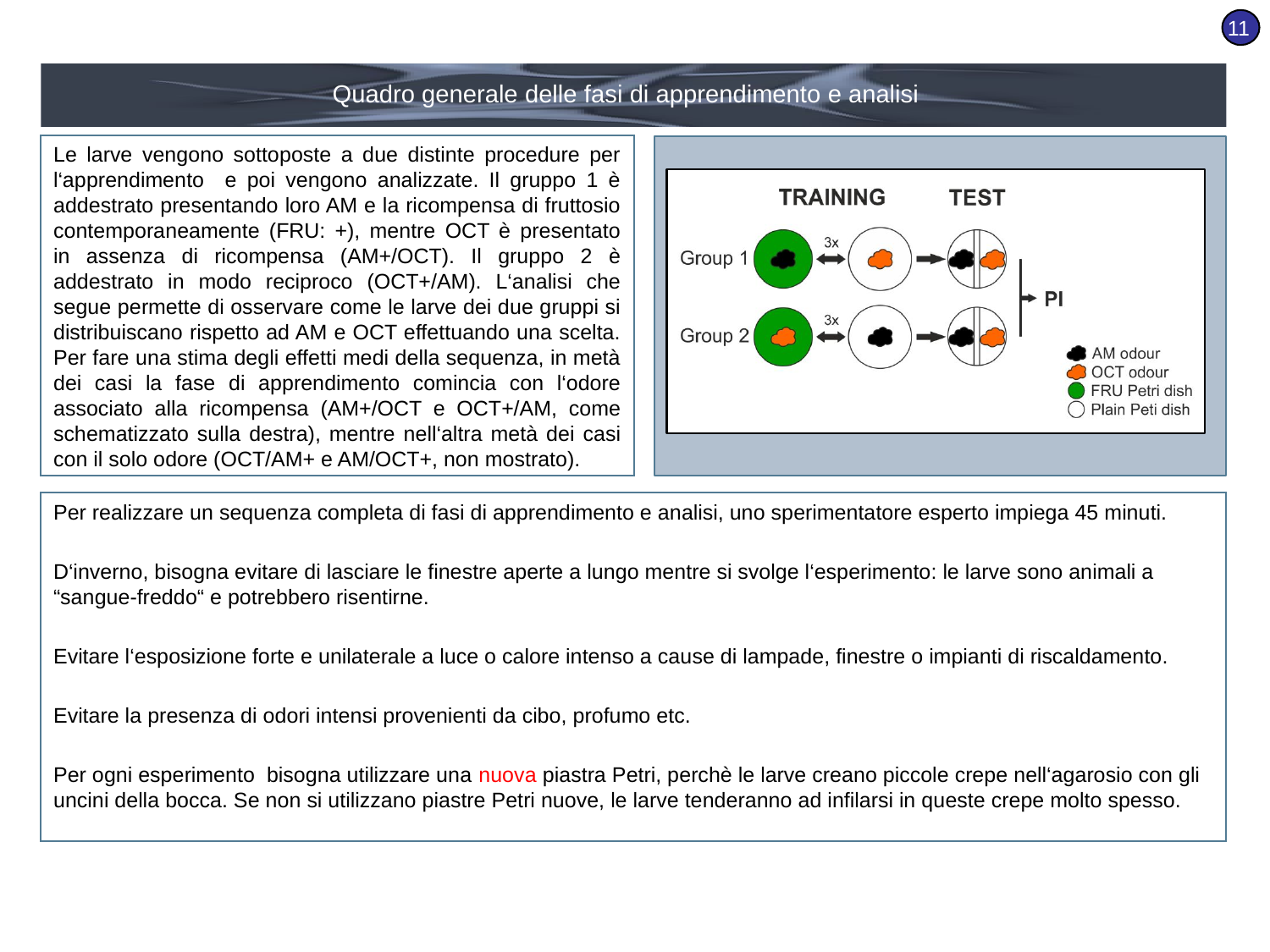

11
Quadro generale delle fasi di apprendimento e analisi
Le larve vengono sottoposte a due distinte procedure per l‘apprendimento e poi vengono analizzate. Il gruppo 1 è addestrato presentando loro AM e la ricompensa di fruttosio contemporaneamente (FRU: +), mentre OCT è presentato in assenza di ricompensa (AM+/OCT). Il gruppo 2 è addestrato in modo reciproco (OCT+/AM). L‘analisi che segue permette di osservare come le larve dei due gruppi si distribuiscano rispetto ad AM e OCT effettuando una scelta. Per fare una stima degli effetti medi della sequenza, in metà dei casi la fase di apprendimento comincia con l‘odore associato alla ricompensa (AM+/OCT e OCT+/AM, come schematizzato sulla destra), mentre nell‘altra metà dei casi con il solo odore (OCT/AM+ e AM/OCT+, non mostrato).
Per realizzare un sequenza completa di fasi di apprendimento e analisi, uno sperimentatore esperto impiega 45 minuti.
D‘inverno, bisogna evitare di lasciare le finestre aperte a lungo mentre si svolge l‘esperimento: le larve sono animali a “sangue-freddo“ e potrebbero risentirne.
Evitare l‘esposizione forte e unilaterale a luce o calore intenso a cause di lampade, finestre o impianti di riscaldamento.
Evitare la presenza di odori intensi provenienti da cibo, profumo etc.
Per ogni esperimento bisogna utilizzare una nuova piastra Petri, perchè le larve creano piccole crepe nell‘agarosio con gli uncini della bocca. Se non si utilizzano piastre Petri nuove, le larve tenderanno ad infilarsi in queste crepe molto spesso.

## Slide 12
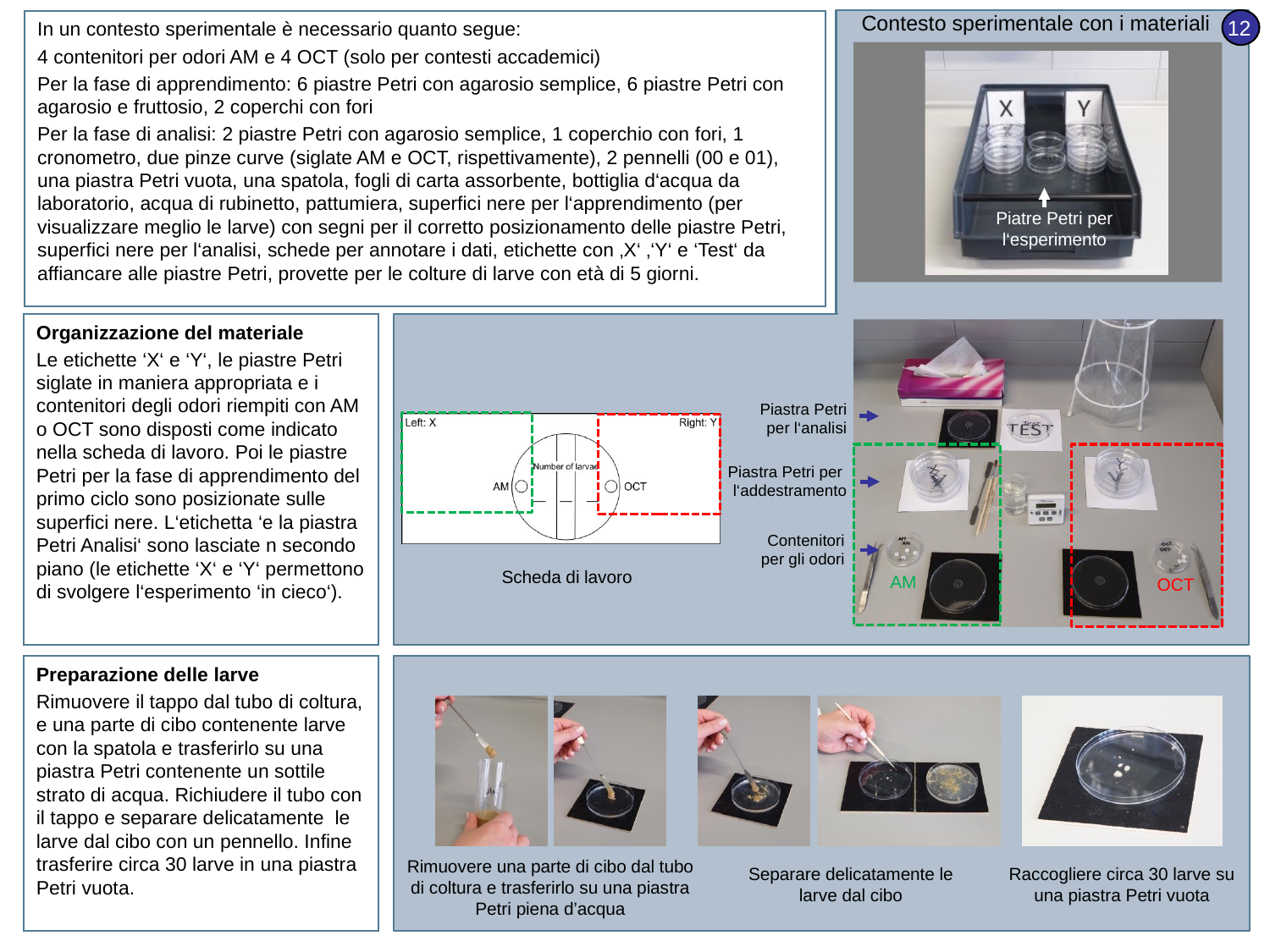

Contesto sperimentale con i materiali
12
In un contesto sperimentale è necessario quanto segue:
4 contenitori per odori AM e 4 OCT (solo per contesti accademici)
Per la fase di apprendimento: 6 piastre Petri con agarosio semplice, 6 piastre Petri con agarosio e fruttosio, 2 coperchi con fori
Per la fase di analisi: 2 piastre Petri con agarosio semplice, 1 coperchio con fori, 1 cronometro, due pinze curve (siglate AM e OCT, rispettivamente), 2 pennelli (00 e 01), una piastra Petri vuota, una spatola, fogli di carta assorbente, bottiglia d‘acqua da laboratorio, acqua di rubinetto, pattumiera, superfici nere per l‘apprendimento (per visualizzare meglio le larve) con segni per il corretto posizionamento delle piastre Petri, superfici nere per l‘analisi, schede per annotare i dati, etichette con ‚X‘ ‚‘Y‘ e ‘Test‘ da affiancare alle piastre Petri, provette per le colture di larve con età di 5 giorni.
Piatre Petri per l‘esperimento
Organizzazione del materiale
Le etichette ‘X‘ e ‘Y‘, le piastre Petri siglate in maniera appropriata e i contenitori degli odori riempiti con AM o OCT sono disposti come indicato nella scheda di lavoro. Poi le piastre Petri per la fase di apprendimento del primo ciclo sono posizionate sulle superfici nere. L‘etichetta ‘e la piastra Petri Analisi‘ sono lasciate n secondo piano (le etichette ‘X‘ e ‘Y‘ permettono di svolgere l‘esperimento ‘in cieco‘).
Piastra Petri
per l‘analisi
OCT
Y
Piastra Petri per
l‘addestramento
Contenitori
per gli odori
Scheda di lavoro
AM
OCT
Preparazione delle larve
Rimuovere il tappo dal tubo di coltura, e una parte di cibo contenente larve con la spatola e trasferirlo su una piastra Petri contenente un sottile strato di acqua. Richiudere il tubo con il tappo e separare delicatamente le larve dal cibo con un pennello. Infine trasferire circa 30 larve in una piastra Petri vuota.
Rimuovere una parte di cibo dal tubo di coltura e trasferirlo su una piastra Petri piena d’acqua
Separare delicatamente le larve dal cibo
Raccogliere circa 30 larve su una piastra Petri vuota

## Slide 13
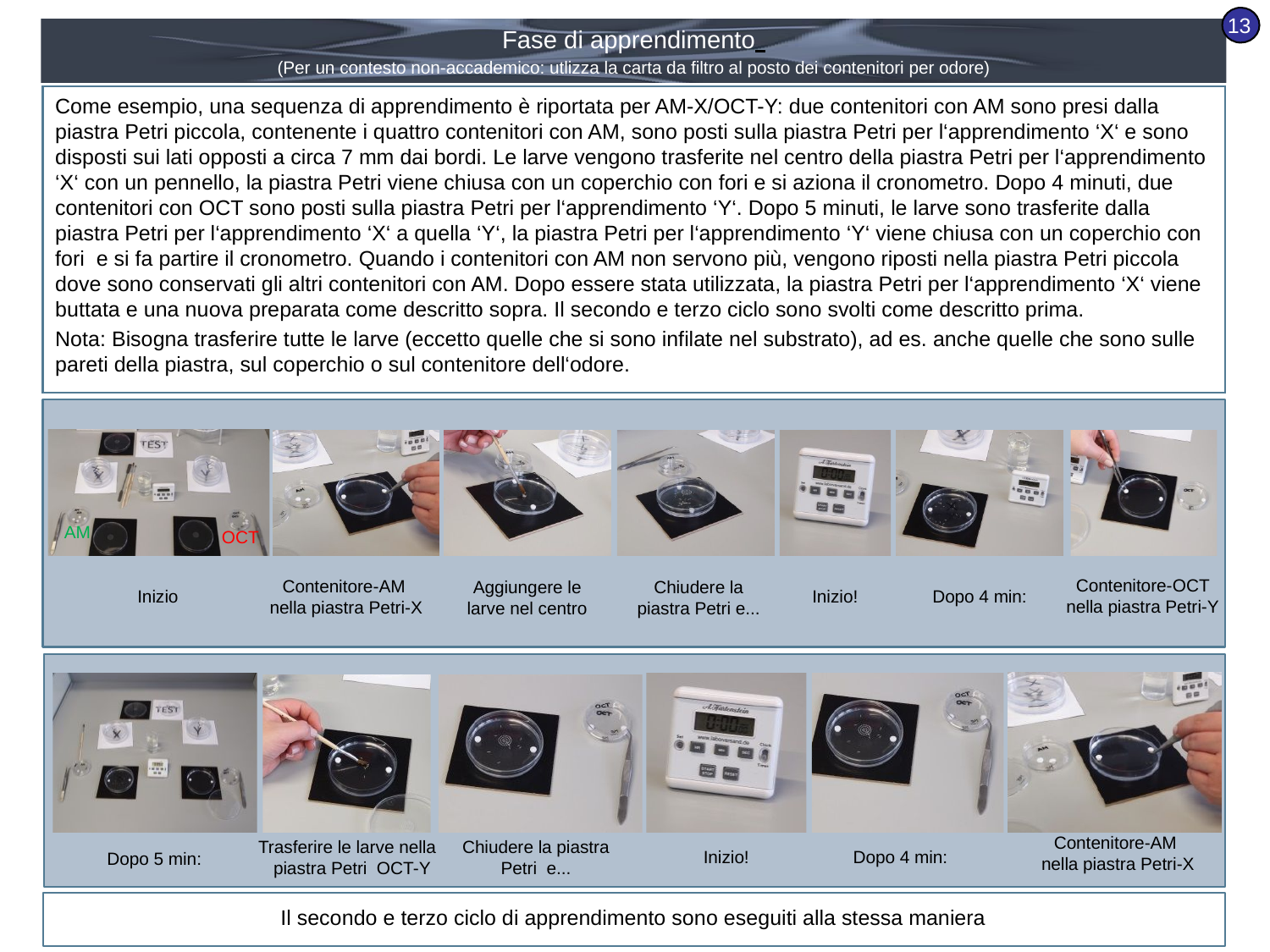

13
# Fase di apprendimento
(Per un contesto non-accademico: utlizza la carta da filtro al posto dei contenitori per odore)
Come esempio, una sequenza di apprendimento è riportata per AM-X/OCT-Y: due contenitori con AM sono presi dalla piastra Petri piccola, contenente i quattro contenitori con AM, sono posti sulla piastra Petri per l‘apprendimento ‘X‘ e sono disposti sui lati opposti a circa 7 mm dai bordi. Le larve vengono trasferite nel centro della piastra Petri per l‘apprendimento ‘X‘ con un pennello, la piastra Petri viene chiusa con un coperchio con fori e si aziona il cronometro. Dopo 4 minuti, due contenitori con OCT sono posti sulla piastra Petri per l‘apprendimento ‘Y‘. Dopo 5 minuti, le larve sono trasferite dalla piastra Petri per l‘apprendimento ‘X‘ a quella ‘Y‘, la piastra Petri per l‘apprendimento ‘Y‘ viene chiusa con un coperchio con fori e si fa partire il cronometro. Quando i contenitori con AM non servono più, vengono riposti nella piastra Petri piccola dove sono conservati gli altri contenitori con AM. Dopo essere stata utilizzata, la piastra Petri per l‘apprendimento ‘X‘ viene buttata e una nuova preparata come descritto sopra. Il secondo e terzo ciclo sono svolti come descritto prima.
Nota: Bisogna trasferire tutte le larve (eccetto quelle che si sono infilate nel substrato), ad es. anche quelle che sono sulle pareti della piastra, sul coperchio o sul contenitore dell‘odore.
AM
OCT
Contenitore-OCT nella piastra Petri-Y
Contenitore-AM
nella piastra Petri-X
Aggiungere le larve nel centro
Chiudere la piastra Petri e...
Dopo 4 min:
Inizio
Inizio!
Contenitore-AM
nella piastra Petri-X
Chiudere la piastra Petri e...
Trasferire le larve nella piastra Petri OCT-Y
Inizio!
Dopo 4 min:
Dopo 5 min:
Il secondo e terzo ciclo di apprendimento sono eseguiti alla stessa maniera

## Slide 14
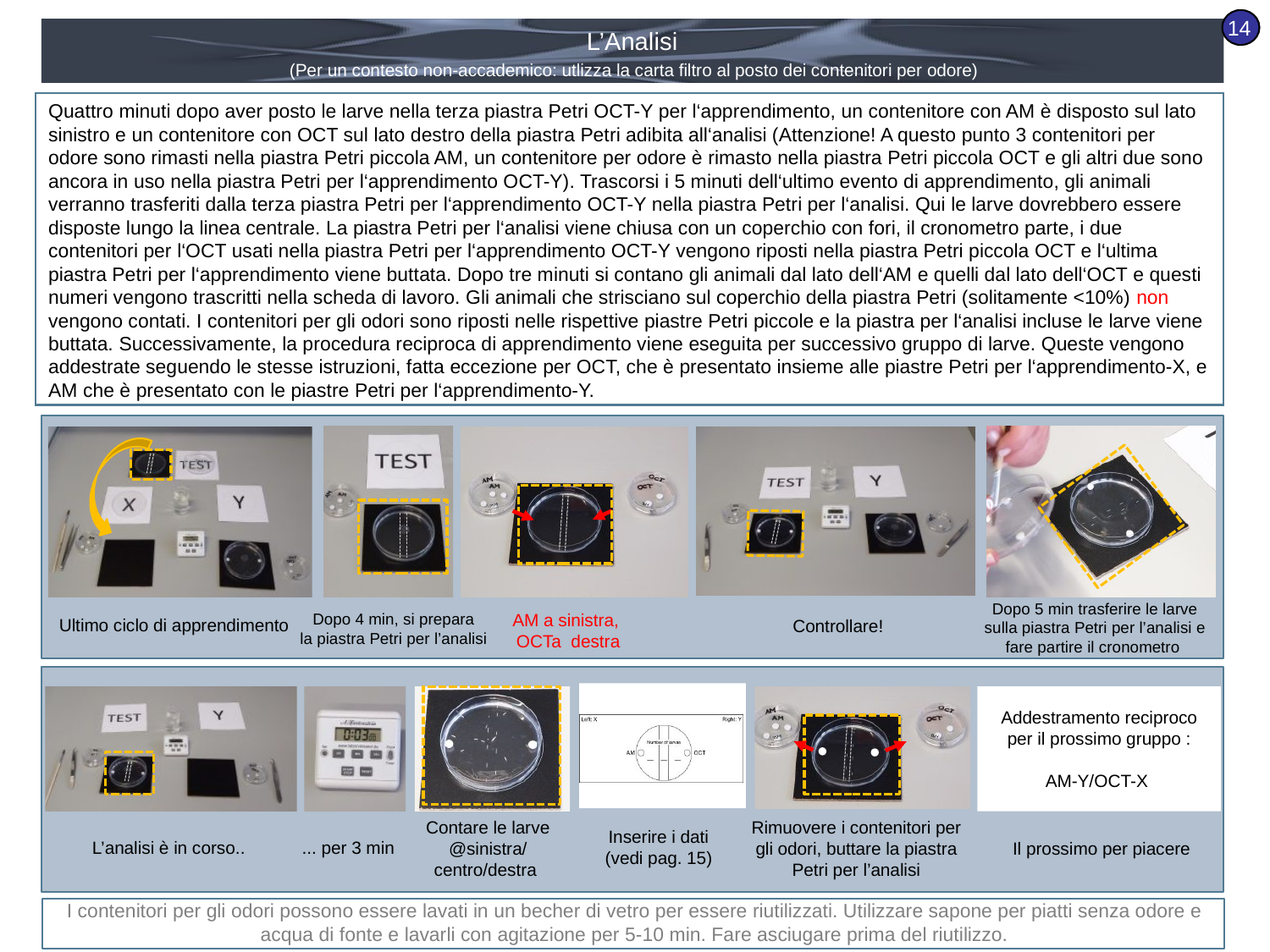

# L’Analisi
14
(Per un contesto non-accademico: utlizza la carta filtro al posto dei contenitori per odore)
Quattro minuti dopo aver posto le larve nella terza piastra Petri OCT-Y per l‘apprendimento, un contenitore con AM è disposto sul lato sinistro e un contenitore con OCT sul lato destro della piastra Petri adibita all‘analisi (Attenzione! A questo punto 3 contenitori per odore sono rimasti nella piastra Petri piccola AM, un contenitore per odore è rimasto nella piastra Petri piccola OCT e gli altri due sono ancora in uso nella piastra Petri per l‘apprendimento OCT-Y). Trascorsi i 5 minuti dell‘ultimo evento di apprendimento, gli animali verranno trasferiti dalla terza piastra Petri per l‘apprendimento OCT-Y nella piastra Petri per l‘analisi. Qui le larve dovrebbero essere disposte lungo la linea centrale. La piastra Petri per l‘analisi viene chiusa con un coperchio con fori, il cronometro parte, i due contenitori per l‘OCT usati nella piastra Petri per l‘apprendimento OCT-Y vengono riposti nella piastra Petri piccola OCT e l‘ultima piastra Petri per l‘apprendimento viene buttata. Dopo tre minuti si contano gli animali dal lato dell‘AM e quelli dal lato dell‘OCT e questi numeri vengono trascritti nella scheda di lavoro. Gli animali che strisciano sul coperchio della piastra Petri (solitamente <10%) non vengono contati. I contenitori per gli odori sono riposti nelle rispettive piastre Petri piccole e la piastra per l‘analisi incluse le larve viene buttata. Successivamente, la procedura reciproca di apprendimento viene eseguita per successivo gruppo di larve. Queste vengono addestrate seguendo le stesse istruzioni, fatta eccezione per OCT, che è presentato insieme alle piastre Petri per l‘apprendimento-X, e AM che è presentato con le piastre Petri per l‘apprendimento-Y.
Dopo 5 min trasferire le larve sulla piastra Petri per l’analisi e fare partire il cronometro
Dopo 4 min, si prepara
la piastra Petri per l’analisi
AM a sinistra,
OCTa destra
Ultimo ciclo di apprendimento
Controllare!
Addestramento reciproco per il prossimo gruppo :
AM-Y/OCT-X
Contare le larve
@sinistra/centro/destra
Rimuovere i contenitori per gli odori, buttare la piastra Petri per l’analisi
Inserire i dati
(vedi pag. 15)
L’analisi è in corso..
... per 3 min
Il prossimo per piacere
I contenitori per gli odori possono essere lavati in un becher di vetro per essere riutilizzati. Utilizzare sapone per piatti senza odore e acqua di fonte e lavarli con agitazione per 5-10 min. Fare asciugare prima del riutilizzo.

## Slide 15
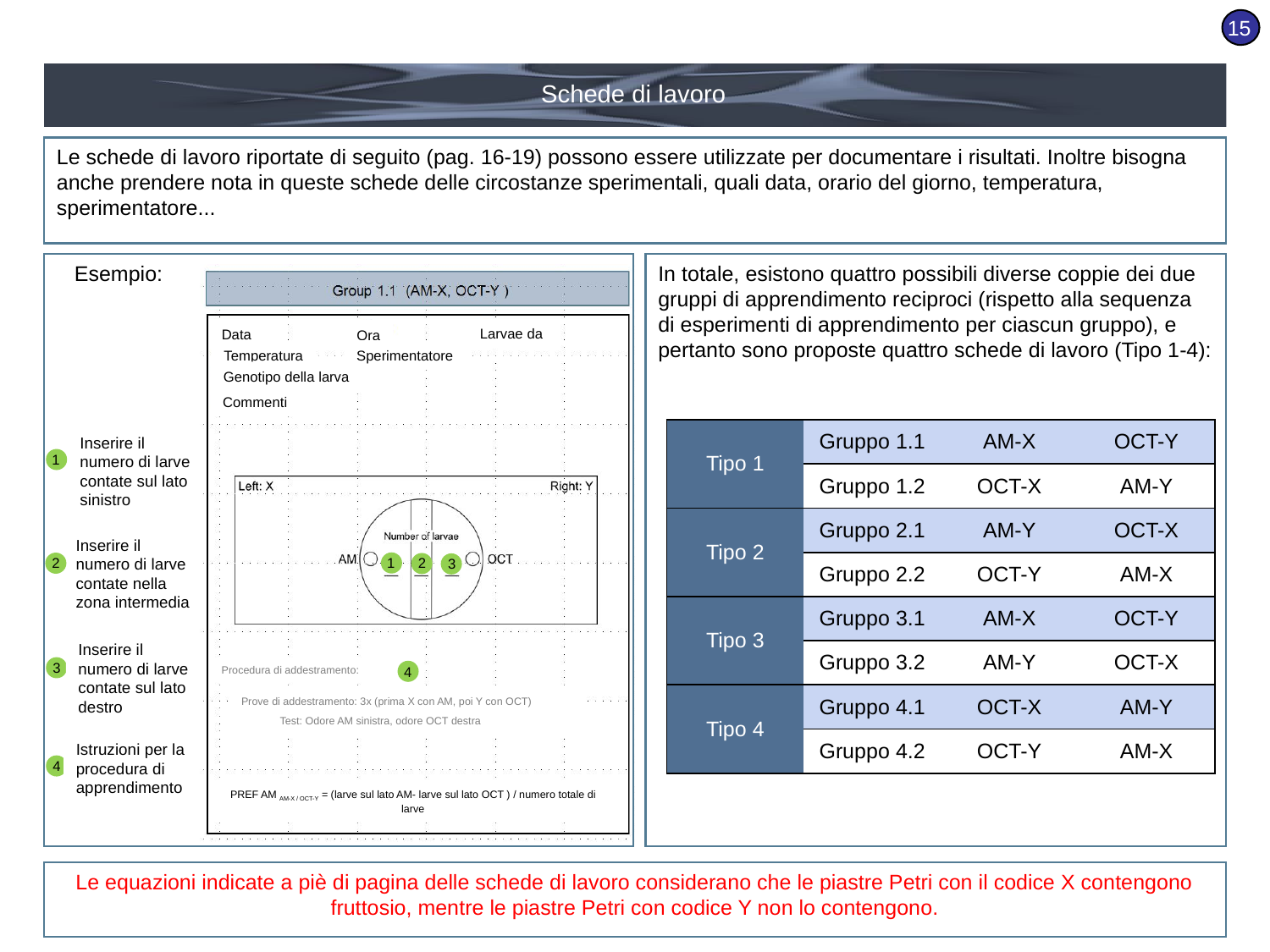

15
Schede di lavoro
Le schede di lavoro riportate di seguito (pag. 16-19) possono essere utilizzate per documentare i risultati. Inoltre bisogna anche prendere nota in queste schede delle circostanze sperimentali, quali data, orario del giorno, temperatura, sperimentatore...
 Esempio:
In totale, esistono quattro possibili diverse coppie dei due gruppi di apprendimento reciproci (rispetto alla sequenza di esperimenti di apprendimento per ciascun gruppo), e pertanto sono proposte quattro schede di lavoro (Tipo 1-4):
Larvae da
Data
Ora
Temperatura
Sperimentatore
Genotipo della larva
Commenti
| Tipo 1 | Gruppo 1.1 | AM-X | OCT-Y |
| --- | --- | --- | --- |
| | Gruppo 1.2 | OCT-X | AM-Y |
| Tipo 2 | Gruppo 2.1 | AM-Y | OCT-X |
| | Gruppo 2.2 | OCT-Y | AM-X |
| Tipo 3 | Gruppo 3.1 | AM-X | OCT-Y |
| | Gruppo 3.2 | AM-Y | OCT-X |
| Tipo 4 | Gruppo 4.1 | OCT-X | AM-Y |
| | Gruppo 4.2 | OCT-Y | AM-X |
Enter number of larve counted on the left side
Inserire il numero di larve contate sul lato sinistro
1
Inserire il numero di larve contate nella zona intermedia
1
2
2
3
Enter number of larve counted on the right side
Inserire il numero di larve contate sul lato destro
3
4
Procedura di addestramento:
Prove di addestramento: 3x (prima X con AM, poi Y con OCT)
 Test: Odore AM sinistra, odore OCT destra
Instructions for training procedure
Istruzioni per la procedura di apprendimento
4
PREF AM AM-X / OCT-Y = (larve sul lato AM- larve sul lato OCT ) / numero totale di larve
Le equazioni indicate a piè di pagina delle schede di lavoro considerano che le piastre Petri con il codice X contengono fruttosio, mentre le piastre Petri con codice Y non lo contengono.

## Slide 16
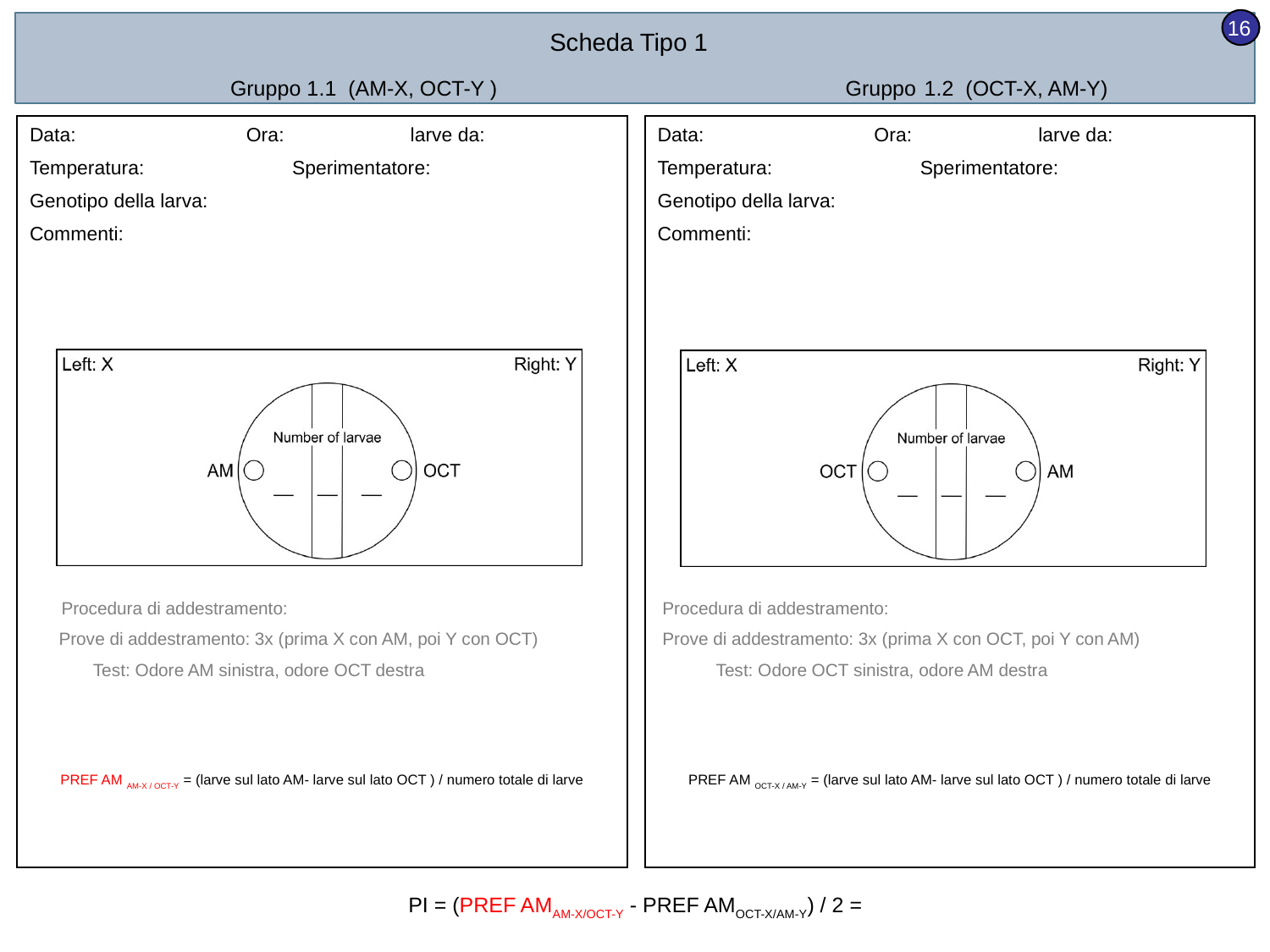

16
# Scheda Tipo 1 Gruppo 1.1 (AM-X, OCT-Y ) Gruppo 1.2 (OCT-X, AM-Y)
Data: Ora: larve da:
Temperatura: 	 Sperimentatore:
Genotipo della larva:
Commenti:
 Procedura di addestramento:
 Prove di addestramento: 3x (prima X con AM, poi Y con OCT)
 Test: Odore AM sinistra, odore OCT destra
PREF AM AM-X / OCT-Y = (larve sul lato AM- larve sul lato OCT ) / numero totale di larve
Data: Ora: larve da:
Temperatura: 	 Sperimentatore:
Genotipo della larva:
Commenti:
 Procedura di addestramento:
 Prove di addestramento: 3x (prima X con OCT, poi Y con AM)
 Test: Odore OCT sinistra, odore AM destra
PREF AM OCT-X / AM-Y = (larve sul lato AM- larve sul lato OCT ) / numero totale di larve
PI = (PREF AMAM-X/OCT-Y - PREF AMOCT-X/AM-Y) / 2 =

## Slide 17
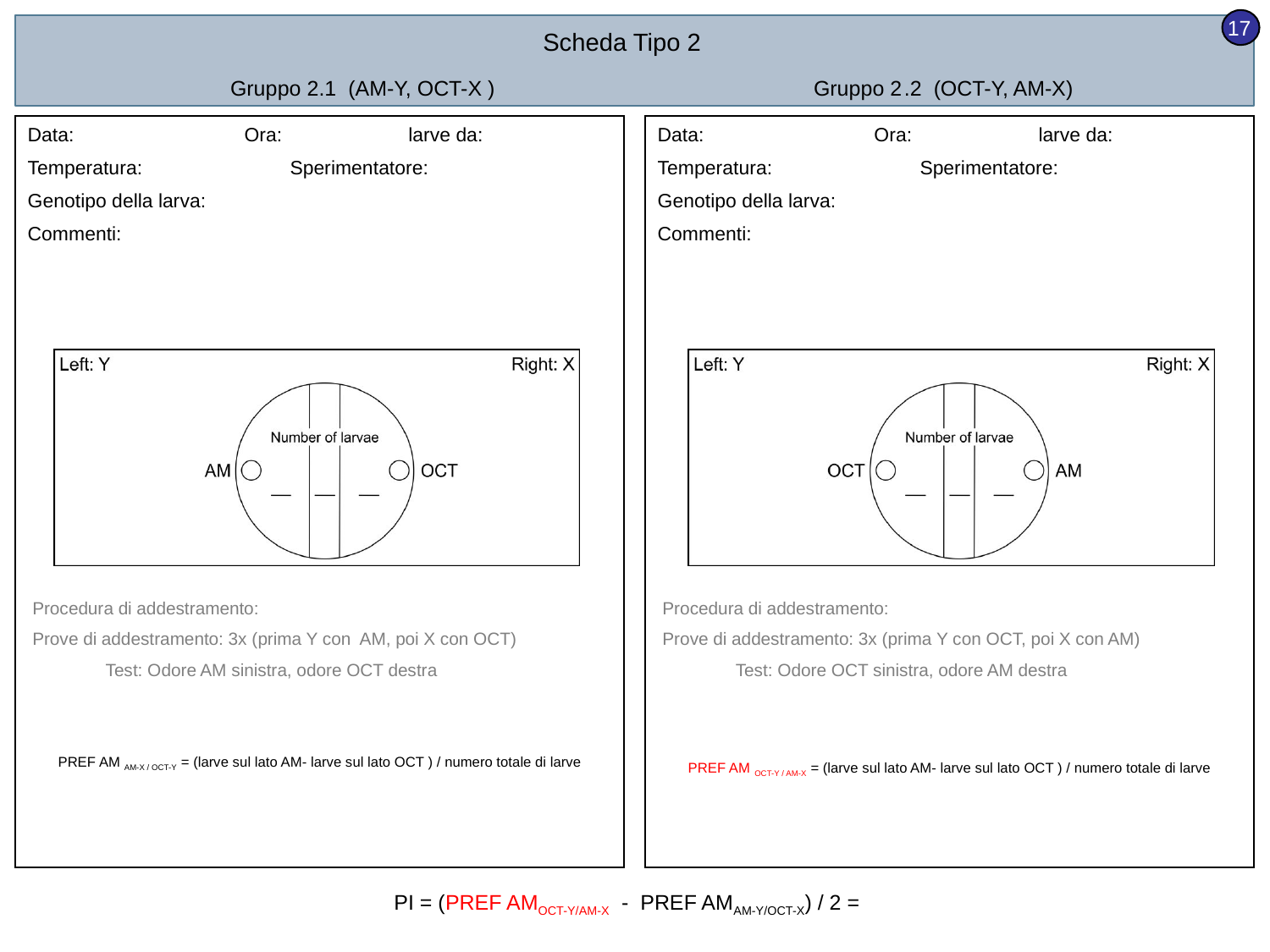

17
				 Scheda Tipo 2
 Gruppo 2.1 (AM-Y, OCT-X ) Gruppo 2.2 (OCT-Y, AM-X)
Data: Ora: larve da:
Temperatura: 	 Sperimentatore:
Genotipo della larva:
Commenti:
 Procedura di addestramento:
 Prove di addestramento: 3x (prima Y con AM, poi X con OCT)
 Test: Odore AM sinistra, odore OCT destra
PREF AM AM-X / OCT-Y = (larve sul lato AM- larve sul lato OCT ) / numero totale di larve
Data: Ora: larve da:
Temperatura: 	 Sperimentatore:
Genotipo della larva:
Commenti:
 Procedura di addestramento:
 Prove di addestramento: 3x (prima Y con OCT, poi X con AM)
 Test: Odore OCT sinistra, odore AM destra
PREF AM OCT-Y / AM-X = (larve sul lato AM- larve sul lato OCT ) / numero totale di larve
PI = (PREF AMOCT-Y/AM-X - PREF AMAM-Y/OCT-X) / 2 =

## Slide 18
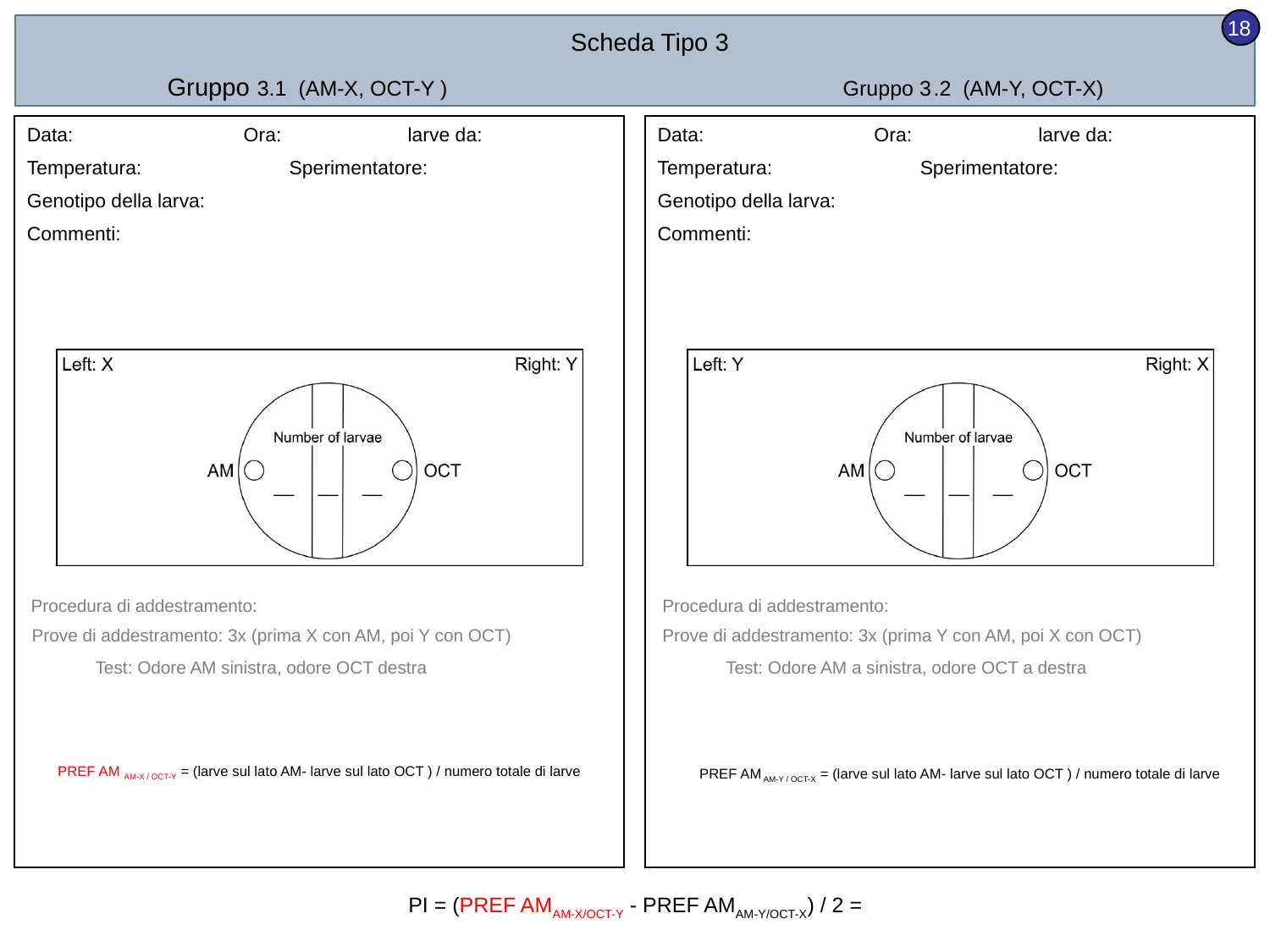

18
				 Scheda Tipo 3
 Gruppo 3.1 (AM-X, OCT-Y ) Gruppo 3.2 (AM-Y, OCT-X)
Data: Ora: larve da:
Temperatura: 	 Sperimentatore:
Genotipo della larva:
Commenti:
 Procedura di addestramento:
 Prove di addestramento: 3x (prima X con AM, poi Y con OCT)
 Test: Odore AM sinistra, odore OCT destra
PREF AM AM-X / OCT-Y = (larve sul lato AM- larve sul lato OCT ) / numero totale di larve
Data: Ora: larve da:
Temperatura: 	 Sperimentatore:
Genotipo della larva:
Commenti:
 Procedura di addestramento:
 Prove di addestramento: 3x (prima Y con AM, poi X con OCT)
 Test: Odore AM a sinistra, odore OCT a destra
 PREF AM AM-Y / OCT-X = (larve sul lato AM- larve sul lato OCT ) / numero totale di larve
PI = (PREF AMAM-X/OCT-Y - PREF AMAM-Y/OCT-X) / 2 =

## Slide 19
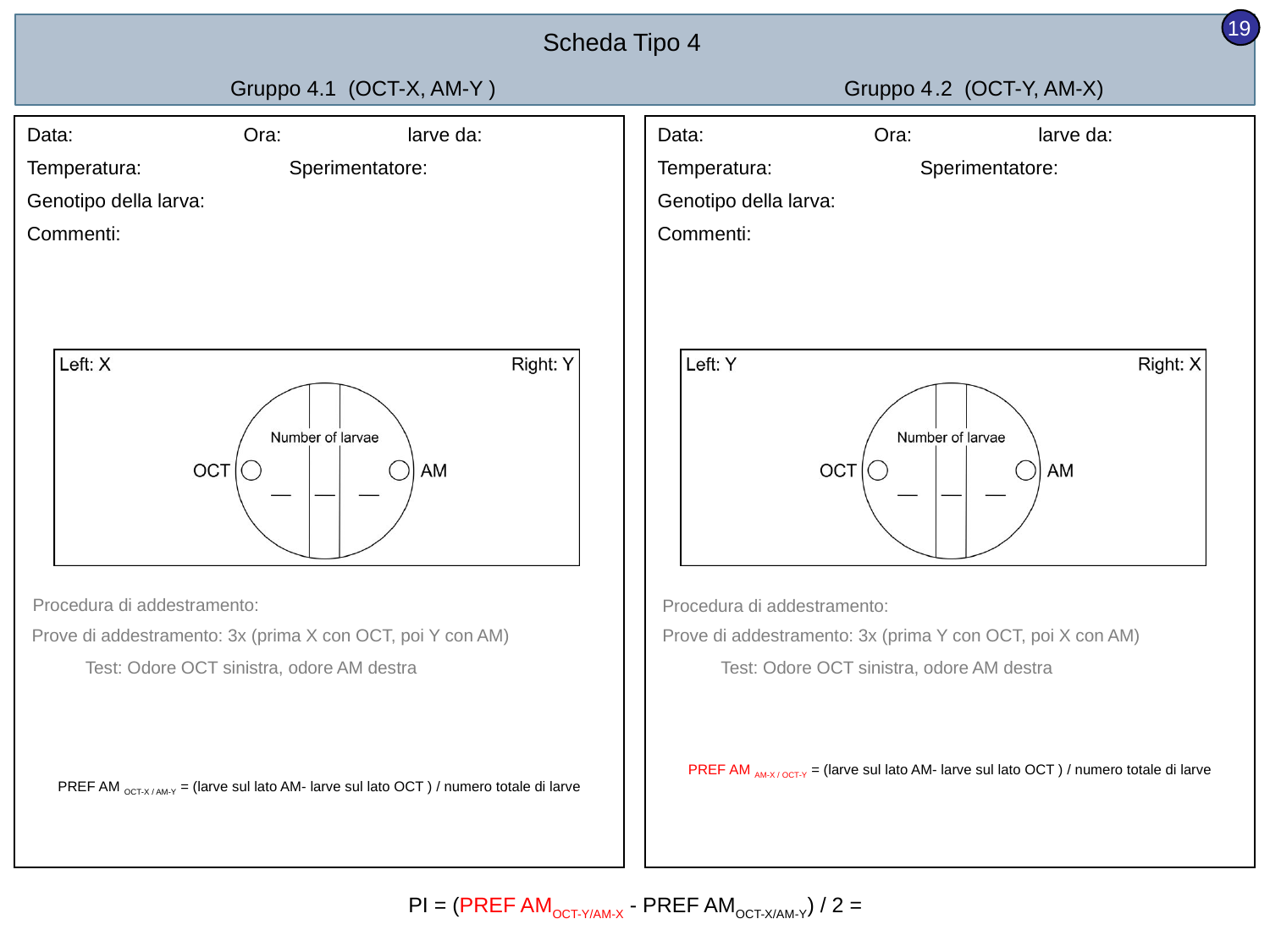

19
				 Scheda Tipo 4
 Gruppo 4.1 (OCT-X, AM-Y ) Gruppo 4.2 (OCT-Y, AM-X)
Data: Ora: larve da:
Temperatura: 	 Sperimentatore:
Genotipo della larva:
Commenti:
 Procedura di addestramento:
 Prove di addestramento: 3x (prima X con OCT, poi Y con AM)
 Test: Odore OCT sinistra, odore AM destra
PREF AM OCT-X / AM-Y = (larve sul lato AM- larve sul lato OCT ) / numero totale di larve
Data: Ora: larve da:
Temperatura: 	 Sperimentatore:
Genotipo della larva:
Commenti:
 Procedura di addestramento:
 Prove di addestramento: 3x (prima Y con OCT, poi X con AM)
 Test: Odore OCT sinistra, odore AM destra
PREF AM AM-X / OCT-Y = (larve sul lato AM- larve sul lato OCT ) / numero totale di larve
PI = (PREF AMOCT-Y/AM-X - PREF AMOCT-X/AM-Y) / 2 =

## Slide 20
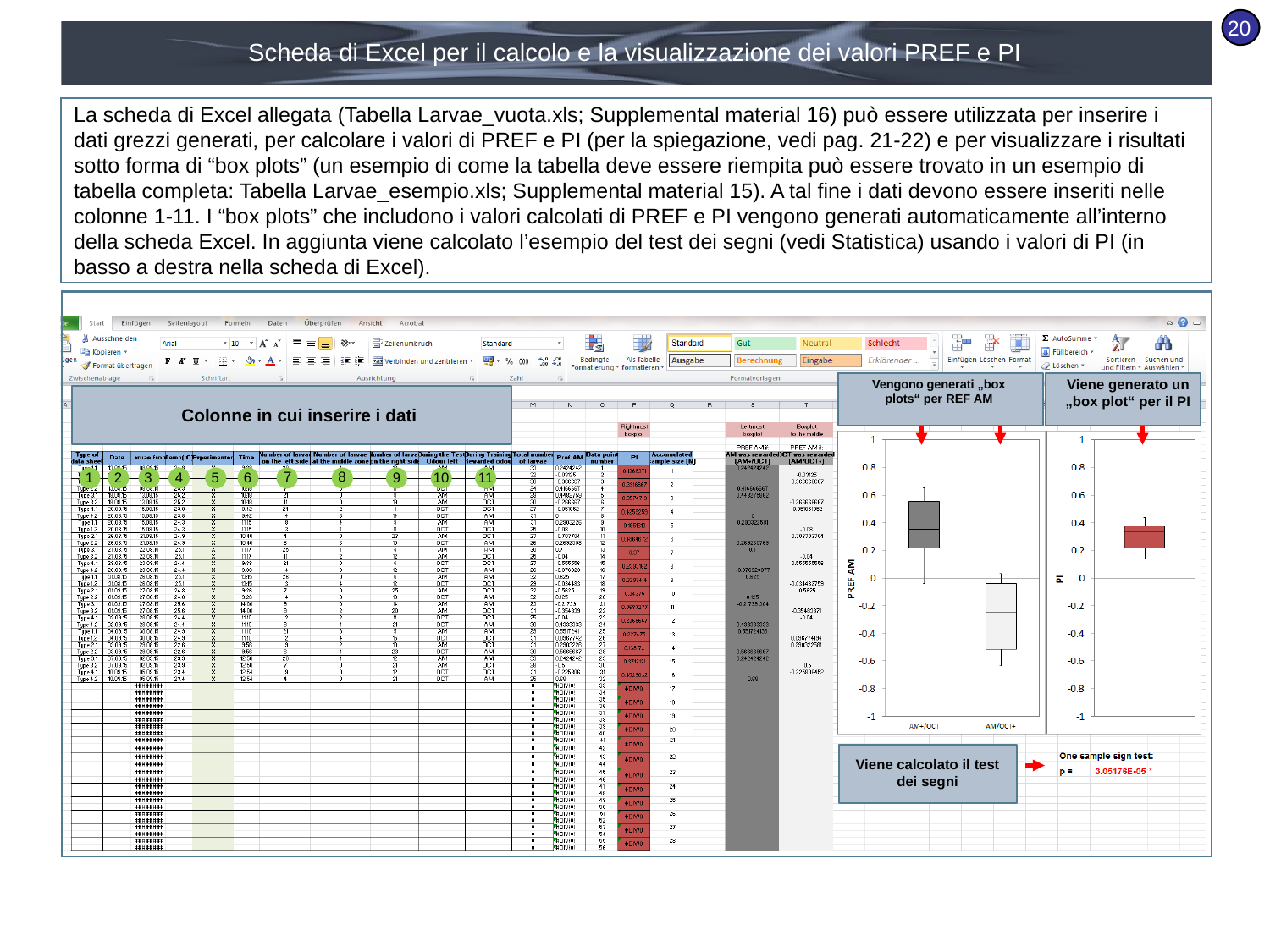

20
Scheda di Excel per il calcolo e la visualizzazione dei valori PREF e PI
La scheda di Excel allegata (Tabella Larvae_vuota.xls; Supplemental material 16) può essere utilizzata per inserire i dati grezzi generati, per calcolare i valori di PREF e PI (per la spiegazione, vedi pag. 21-22) e per visualizzare i risultati sotto forma di “box plots” (un esempio di come la tabella deve essere riempita può essere trovato in un esempio di tabella completa: Tabella Larvae_esempio.xls; Supplemental material 15). A tal fine i dati devono essere inseriti nelle colonne 1-11. I “box plots” che includono i valori calcolati di PREF e PI vengono generati automaticamente all’interno della scheda Excel. In aggiunta viene calcolato l’esempio del test dei segni (vedi Statistica) usando i valori di PI (in basso a destra nella scheda di Excel).
Viene generato un „box plot“ per il PI
Vengono generati „box plots“ per REF AM
Colonne in cui inserire i dati
7
8
3
4
2
5
6
9
10
1
11
Viene calcolato il test dei segni

## Slide 21
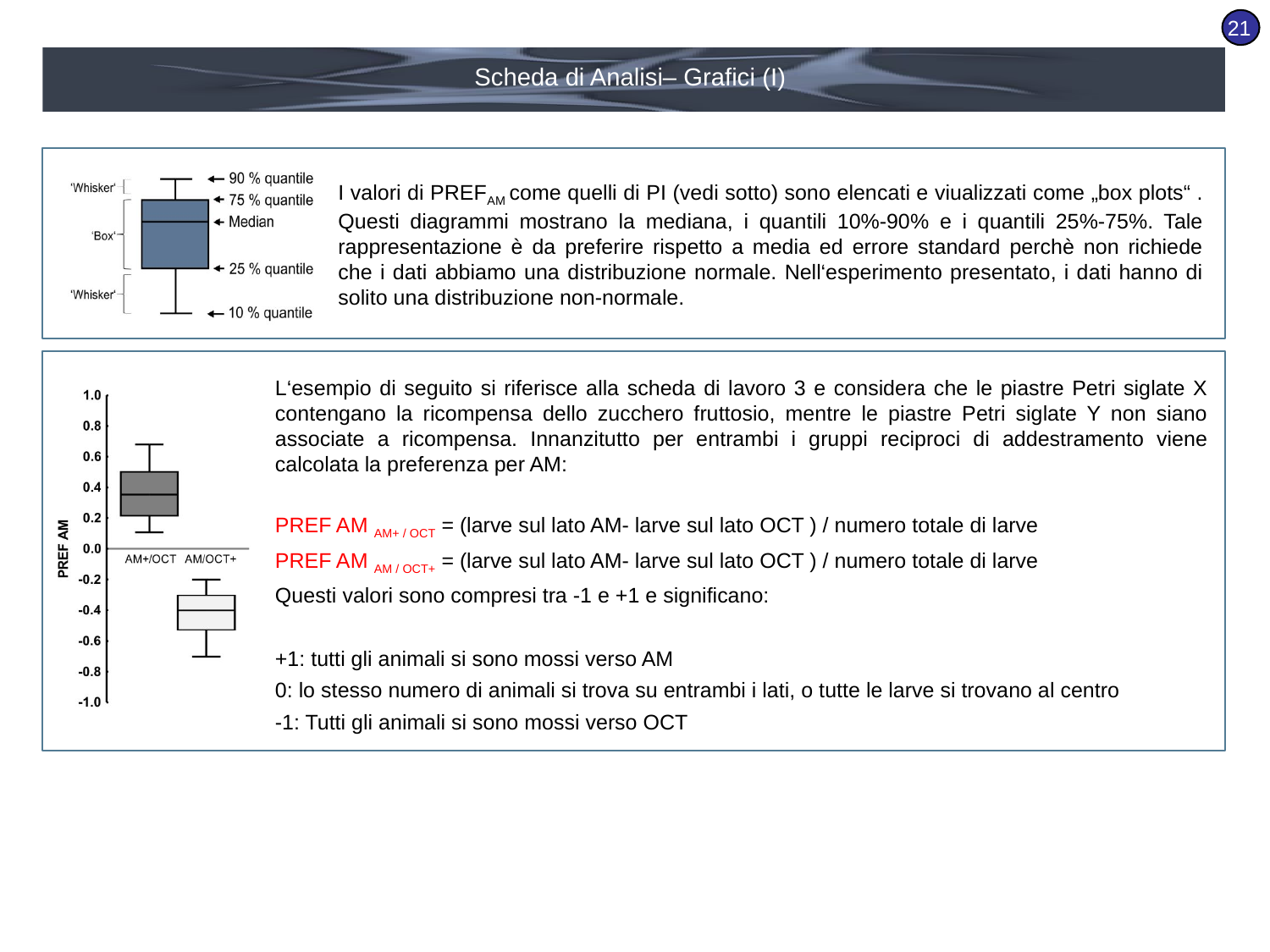

21
Scheda di Analisi– Grafici (I)
I valori di PREFAM come quelli di PI (vedi sotto) sono elencati e viualizzati come „box plots“ . Questi diagrammi mostrano la mediana, i quantili 10%-90% e i quantili 25%-75%. Tale rappresentazione è da preferire rispetto a media ed errore standard perchè non richiede che i dati abbiamo una distribuzione normale. Nell‘esperimento presentato, i dati hanno di solito una distribuzione non-normale.
L‘esempio di seguito si riferisce alla scheda di lavoro 3 e considera che le piastre Petri siglate X contengano la ricompensa dello zucchero fruttosio, mentre le piastre Petri siglate Y non siano associate a ricompensa. Innanzitutto per entrambi i gruppi reciproci di addestramento viene calcolata la preferenza per AM:
PREF AM AM+ / OCT = (larve sul lato AM- larve sul lato OCT ) / numero totale di larve
PREF AM AM / OCT+ = (larve sul lato AM- larve sul lato OCT ) / numero totale di larve
Questi valori sono compresi tra -1 e +1 e significano:
+1: tutti gli animali si sono mossi verso AM
0: lo stesso numero di animali si trova su entrambi i lati, o tutte le larve si trovano al centro
-1: Tutti gli animali si sono mossi verso OCT

## Slide 22
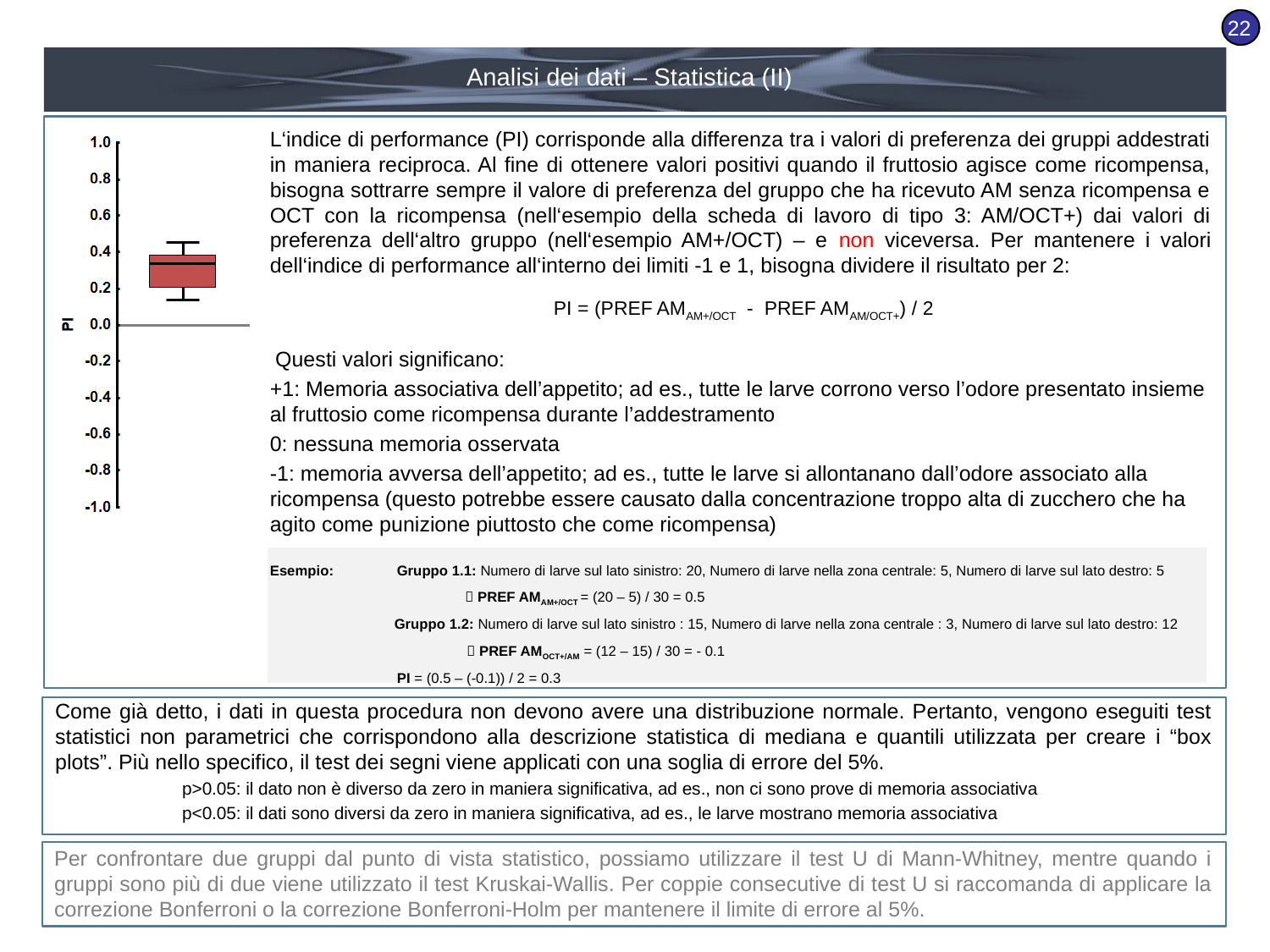

22
Analisi dei dati – Statistica (II)
L‘indice di performance (PI) corrisponde alla differenza tra i valori di preferenza dei gruppi addestrati in maniera reciproca. Al fine di ottenere valori positivi quando il fruttosio agisce come ricompensa, bisogna sottrarre sempre il valore di preferenza del gruppo che ha ricevuto AM senza ricompensa e OCT con la ricompensa (nell‘esempio della scheda di lavoro di tipo 3: AM/OCT+) dai valori di preferenza dell‘altro gruppo (nell‘esempio AM+/OCT) – e non viceversa. Per mantenere i valori dell‘indice di performance all‘interno dei limiti -1 e 1, bisogna dividere il risultato per 2:
 PI = (PREF AMAM+/OCT - PREF AMAM/OCT+) / 2
 Questi valori significano:
+1: Memoria associativa dell’appetito; ad es., tutte le larve corrono verso l’odore presentato insieme al fruttosio come ricompensa durante l’addestramento
0: nessuna memoria osservata
-1: memoria avversa dell’appetito; ad es., tutte le larve si allontanano dall’odore associato alla ricompensa (questo potrebbe essere causato dalla concentrazione troppo alta di zucchero che ha agito come punizione piuttosto che come ricompensa)
Esempio: 	Gruppo 1.1: Numero di larve sul lato sinistro: 20, Numero di larve nella zona centrale: 5, Numero di larve sul lato destro: 5
	  PREF AMAM+/OCT = (20 – 5) / 30 = 0.5
 Gruppo 1.2: Numero di larve sul lato sinistro : 15, Numero di larve nella zona centrale : 3, Numero di larve sul lato destro: 12
  PREF AMOCT+/AM = (12 – 15) / 30 = - 0.1
	PI = (0.5 – (-0.1)) / 2 = 0.3
Come già detto, i dati in questa procedura non devono avere una distribuzione normale. Pertanto, vengono eseguiti test statistici non parametrici che corrispondono alla descrizione statistica di mediana e quantili utilizzata per creare i “box plots”. Più nello specifico, il test dei segni viene applicati con una soglia di errore del 5%.
	p>0.05: il dato non è diverso da zero in maniera significativa, ad es., non ci sono prove di memoria associativa
	p<0.05: il dati sono diversi da zero in maniera significativa, ad es., le larve mostrano memoria associativa
Per confrontare due gruppi dal punto di vista statistico, possiamo utilizzare il test U di Mann-Whitney, mentre quando i gruppi sono più di due viene utilizzato il test Kruskai-Wallis. Per coppie consecutive di test U si raccomanda di applicare la correzione Bonferroni o la correzione Bonferroni-Holm per mantenere il limite di errore al 5%.

## Slide 23
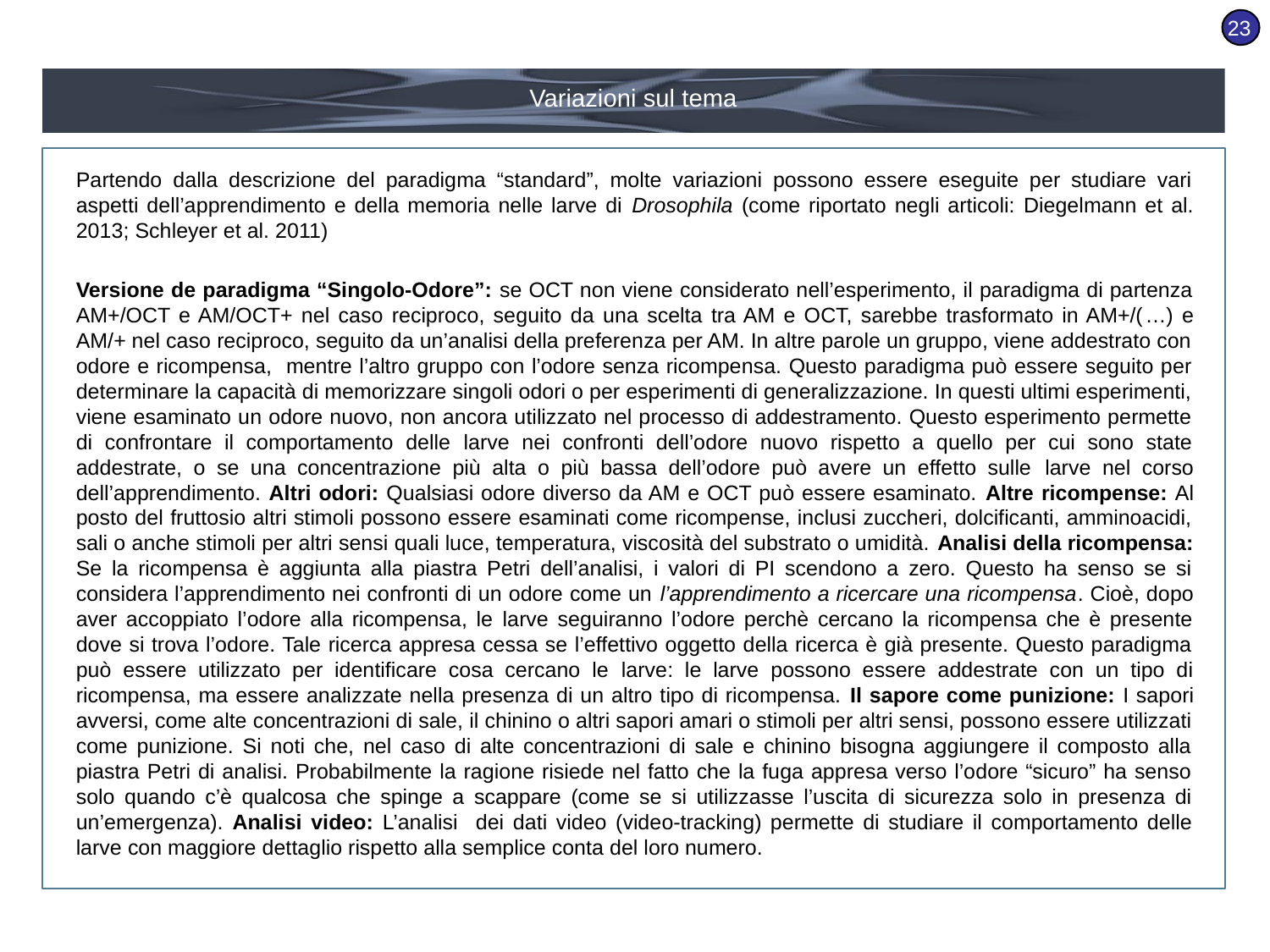

23
Variazioni sul tema
Partendo dalla descrizione del paradigma “standard”, molte variazioni possono essere eseguite per studiare vari aspetti dell’apprendimento e della memoria nelle larve di Drosophila (come riportato negli articoli: Diegelmann et al. 2013; Schleyer et al. 2011)
Versione de paradigma “Singolo-Odore”: se OCT non viene considerato nell’esperimento, il paradigma di partenza AM+/OCT e AM/OCT+ nel caso reciproco, seguito da una scelta tra AM e OCT, sarebbe trasformato in AM+/(…) e AM/+ nel caso reciproco, seguito da un’analisi della preferenza per AM. In altre parole un gruppo, viene addestrato con odore e ricompensa, mentre l’altro gruppo con l’odore senza ricompensa. Questo paradigma può essere seguito per determinare la capacità di memorizzare singoli odori o per esperimenti di generalizzazione. In questi ultimi esperimenti, viene esaminato un odore nuovo, non ancora utilizzato nel processo di addestramento. Questo esperimento permette di confrontare il comportamento delle larve nei confronti dell’odore nuovo rispetto a quello per cui sono state addestrate, o se una concentrazione più alta o più bassa dell’odore può avere un effetto sulle larve nel corso dell’apprendimento. Altri odori: Qualsiasi odore diverso da AM e OCT può essere esaminato. Altre ricompense: Al posto del fruttosio altri stimoli possono essere esaminati come ricompense, inclusi zuccheri, dolcificanti, amminoacidi, sali o anche stimoli per altri sensi quali luce, temperatura, viscosità del substrato o umidità. Analisi della ricompensa: Se la ricompensa è aggiunta alla piastra Petri dell’analisi, i valori di PI scendono a zero. Questo ha senso se si considera l’apprendimento nei confronti di un odore come un l’apprendimento a ricercare una ricompensa. Cioè, dopo aver accoppiato l’odore alla ricompensa, le larve seguiranno l’odore perchè cercano la ricompensa che è presente dove si trova l’odore. Tale ricerca appresa cessa se l’effettivo oggetto della ricerca è già presente. Questo paradigma può essere utilizzato per identificare cosa cercano le larve: le larve possono essere addestrate con un tipo di ricompensa, ma essere analizzate nella presenza di un altro tipo di ricompensa. Il sapore come punizione: I sapori avversi, come alte concentrazioni di sale, il chinino o altri sapori amari o stimoli per altri sensi, possono essere utilizzati come punizione. Si noti che, nel caso di alte concentrazioni di sale e chinino bisogna aggiungere il composto alla piastra Petri di analisi. Probabilmente la ragione risiede nel fatto che la fuga appresa verso l’odore “sicuro” ha senso solo quando c’è qualcosa che spinge a scappare (come se si utilizzasse l’uscita di sicurezza solo in presenza di un’emergenza). Analisi video: L’analisi dei dati video (video-tracking) permette di studiare il comportamento delle larve con maggiore dettaglio rispetto alla semplice conta del loro numero.

## Slide 24
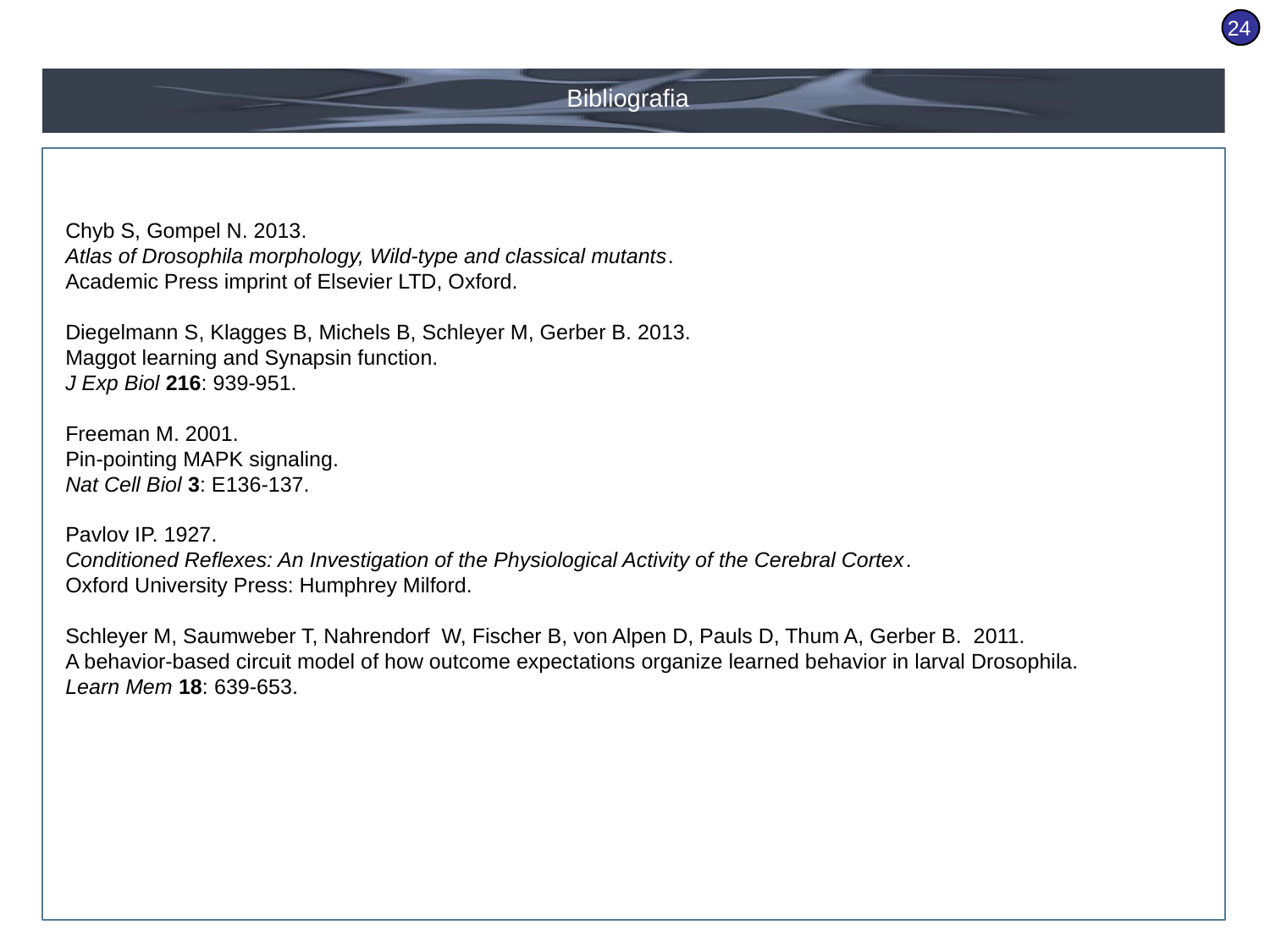

24
Bibliografia
Chyb S, Gompel N. 2013.
Atlas of Drosophila morphology, Wild-type and classical mutants.
Academic Press imprint of Elsevier LTD, Oxford.
Diegelmann S, Klagges B, Michels B, Schleyer M, Gerber B. 2013.
Maggot learning and Synapsin function.
J Exp Biol 216: 939-951.
Freeman M. 2001.
Pin-pointing MAPK signaling.
Nat Cell Biol 3: E136-137.
Pavlov IP. 1927.
Conditioned Reflexes: An Investigation of the Physiological Activity of the Cerebral Cortex.
Oxford University Press: Humphrey Milford.
Schleyer M, Saumweber T, Nahrendorf W, Fischer B, von Alpen D, Pauls D, Thum A, Gerber B. 2011.
A behavior-based circuit model of how outcome expectations organize learned behavior in larval Drosophila.
Learn Mem 18: 639-653.
